# The m6A reader IGF2BP3 promotes acute myeloid leukemia progression by enhancing RCC2 stability

**DOI:** 10.1038/s12276-022-00735-x

**Published:** 2022-02-25

**Authors:** Nan Zhang, Yan Shen, Huan Li, Ying Chen, Ping Zhang, Shifeng Lou, Jianchuan Deng

**Affiliations:** Department of Hematology, The Second Affiliated Hospital of Chongqing Medical University, Chongqing, 400010 China

**Keywords:** Myeloma, Epigenetics

## Abstract

N6-methyladenosine (m6A) is the most abundant posttranscriptional modification of mRNA in eukaryotes. Recent evidence suggests that dysregulated m6A-associated proteins and m6A modifications play a pivotal role in the initiation and progression of diseases such as cancer. Here, we identified that IGF2BP3 is specifically overexpressed in acute myeloid leukemia (AML), a subtype of leukemia associated with poor prognosis and high genetic risk. IGF2BP3 is required for maintaining AML cell survival in an m6A-dependent manner, and knockdown of IGF2BP3 dramatically suppresses the apoptosis, reduces the proliferation, and impairs the leukemic capacity of AML cells in vitro and in vivo. Mechanistically, IGF2BP3 interacts with RCC2 mRNA and stabilizes the expression of m6A-modified RNA. Thus, we provided compelling evidence demonstrating that the m6A reader IGF2BP3 contributes to tumorigenesis and poor prognosis in AML and can serve as a target for the development of cancer therapeutics.

## Leukemia: Key component of disease progression identified

Inhibiting a protein that is overexpressed in the bone marrow of acute myeloid leukemia patients may prove valuable in treating the disease. Recent research has demonstrated the important role played by epigenetics in cancers – for example, disruption to a common mRNA modification known as m6A can result in cancer initiation and progression. Jianchuan Deng and co-workers at Chongqing Medical Universit0y, China, examined the role of an m6A-related protein called IGF2BP3 in mice models and samples from leukemia patients. IGF2BP3 was overexpressed in patients’ bone marrows, the levels of the protein correlating with extent of proliferation of leukemia cells and poor prognosis. IGF2BP3 stabilises the activity of a known cancer-related protein, promoting leukemia progression. Blocking IGF2BP3 expression reduced cell proliferation and impaired activity of leukemic cells, suggesting the protein may be a useful therapeutic target.

## Introduction

Leukemia is caused by genetic mutations and chromosomal aberrations that alter the growth and differentiation program of hematopoietic cells^1^. In the last few decades, epigenetic modifications (such as DNA methylation and histone modifications) have been found to play an important role in this process, and they are currently therapeutic targets in acute myeloid leukemia (AML) and other hematologic malignancies^2^. We previously reported the complex regulation of some genetic mechanisms, such as noncoding RNAs and alternative splicing in AML, and provided some insights into personalized medicine^3–6^. Currently, more in-depth investigation is essential for clarifying the pathogenesis of AML.

RNA methylation, especially with N6-methyladenosine (m6A), is the most predominant internal modification of eukaryotic mRNAs and has been reported to be involved in the regulation of RNA splicing, stability, translation, and localization^7,8^. The process of m6A methylation is dynamically and reversibly regulated by methyltransferases, functional effectors, and demethylases, which are also jargonized as writers, readers, and erasers, respectively^9^. The sites of methylation are conserved DRACH (D = G, A, or U; R = G or A; H = A, C, or U) motifs and are highly dynamic^10^. Evidence has recently emerged that dysregulated m6A-associated proteins and m6A modifications play a pivotal role in the initiation and progression of diseases such as cancer^9^. For instance, METTL3, an m6A methyltransferase, is highly expressed in AML. It not only activates the oncogene c-MYC by enhancing m6A modification of SP1 but also promotes the occurrence of AML by regulating the translation of BCL2 and PTEN mRNAs through m6A modification^11,12^. FTO and ALKBH5, m6A demethylases, are abnormally expressed in AML and promote tumorigenesis and self-renewal of leukemia stem cells through m6A-dependent regulation of their target mRNAs^13–15^. Moreover, the fate of m6A-modified mRNAs is dependent on selective binding proteins^16^. To date, the role of various m6A reader proteins in the progression of leukemia remains largely unexplored.

Here, we aim to address these questions first by determining the expression patterns of the typical m6A-associated writers, erasers, and readers in AML patient samples. Consequently, IGF2BP3 emerged as the most highly expressed m6A reader gene in high-risk AML patients. We then confirmed the overexpression of IGF2BP3 in clinical bone marrow samples of patients with AML compared with those of patients with iron deficiency anemia (IDA), and higher IGF2BP3 expression was indeed correlated with the proliferation and apoptosis of leukemic cells. Furthermore, reduced IGF2BP3 expression inhibited the progression of AML by changing the stability of RCC2 mRNA in an m6A-dependent manner. In summary, our results reveal that upregulation of IGF2BP3, an important m6A regulator promoting tumor progression, is most likely the carcinogenic mechanism in the great majority of AML cases.

## Materials and methods

### Clinical specimens and cell lines

Bone marrow aspirates were obtained from patients with AML who were initially diagnosed in the Hematology Department of the Second Affiliated Hospital of Chongqing Medical University between August 2019 and January 2021. Before starting the research, written informed consent was obtained from all the participants. This study was approved by the Ethics in Research Committee of Chongqing Medical University in Chongqing, China.

The HL-60 cell line was purchased from Procell Life Science & Technology Co., Ltd. (Wuhan, China). The KG-1 cell line was purchased from Chuanqiu Science & Technology Co., Ltd. (Shanghai, China). The SUP-B15 cell line was obtained from the Institute of Hematology at Army Medical University. The THP-1 cell line was obtained from the Institute of Life Sciences at Chongqing Medical University. The RS4;11, Raji, and K562 cell lines were maintained in our lab. KG-1, SUP-B15, K562, and HL-60 cells were grown in IMDM supplemented with 20% fetal bovine serum and penicillin/streptomycin. RS4;11, Raji and THP-1 cells were grown in RPMI-1640 medium supplemented with 10% fetal bovine serum and 1% penicillin/streptomycin, and additional 0.05 mM beta-mercaptoethanol was added to the medium for THP-1 cells.

### Quantitative RT–PCR

Total RNA was extracted from cells using TRIzol reagent (Invitrogen, Carlsbad, CA, USA) according to the manufacturer’s instructions. Subsequently, complementary DNA (cDNA) was synthesized using a PrimeScript^TM^ RT Master Mix Kit (TaKaRa, Dalian, China). Real-time PCR analysis was performed using SYBR Premix Ex Taq^TM^ (Tli RNaseH Plus) (TaKaRa, Dalian, China). Expression was normalized to the housekeeping control GAPDH or ACTIN. The amplification primers are shown in Supplementary Table 1.

### Cell transduction and transfection

Lentiviral vectors for IGF2BP3 knockdown and overexpression (NM_006547) were purchased from GeneChem (Shanghai, China), and empty vector was used as the negative control (NC). Green fluorescent protein (GFP) was used to assess the transduction efficiency, and puromycin was used to select stably transduced cells. The siRNA sequences targeting RCC2 were designed and synthesized by GenePharma Company (Shanghai, China). The relevant sequences are listed in Supplementary Table 1. Each comparison was performed using pairs of samples from the same batch, thus eliminating batch-to-batch variation. All transfections were performed according to the manufacturers’ instructions.

### Cell proliferation, apoptosis, and cell cycle assays

Cell proliferation assays were performed using a Cell Counting Kit-8 (CCK-8, APExBio, USA). Proliferation rates were determined 0, 24, 48, 72, and 96 h after infection, and the absorbance was measured at 450 nm following the manufacturer-recommended protocol. The apoptosis rate was confirmed by flow cytometry (FCM) using an Annexin V-FITC/PI apoptosis detection kit (Elabscience Biotechnology Co., Ltd., Wuhan, China). Cell cycle analysis was performed using a Cell Cycle Analysis Kit (Sigma, St. Louis, MO, USA).

### Immunoblot assay

Total protein was extracted from cultured cells with a Protein Extraction Kit (Kaiji, Nanjing, China). Proteins were separated on SDS–PAGE gels and transferred onto PVDF membranes (0.45-μM pore, Millipore). Blots were probed with anti-IGF2BP3 (14642-1-AP, Proteintech), anti-RCC2 (16755-1-AP, Proteintech), anti-BCL2 (BA0412, Boster Bio), anti-BAX (BA0315-2, Boster Bio), anti-cleaved Caspase3 (29034, Signalway Antibody), and anti-GAPDH (HRP-60004, Proteintech) antibodies. Anti-rabbit or anti-mouse HRP-conjugated secondary antibodies were used prior to ECL detection.

### RNA sequencing analysis

Total RNA was isolated from IGF2BP3 knockdown and control HL-60 cells using TRIzol Reagent. RNA sequencing (RNA-seq) analysis was performed at LC Biotech Inc. (Hangzhou, China).

### RNA immunoprecipitation (RIP) assay

The RIP assay was performed according to the instructions of the Geneseed RIP Kit (Guangzhou, China). Briefly, magnetic beads were mixed with anti-m6A/IGF2BP3/IgG antibodies before the addition of cell lysates. Then, the bound complexes were thoroughly washed, eluted, purified, and analyzed by RT–qPCR. Enrichment of precipitated RNAs was normalized relative to input controls.

### mRNA stability assay

Cells were seeded in 6-well plates and were then treated with 5 μg/mL actinomycin D for 0 h, 3 h, or 6 h prior to RNA extraction. The transcript level of RCC2 mRNA was estimated as the half-life of the mRNA and normalized to ACTIN as the standard.

### Tumor xenograft model

All animal experimental procedures used in this study were approved by the Animal Ethics Committee of Chongqing Medical University. Male BALB/c nude mice (4–6 weeks, 18–22 g) purchased from Vital River (Beijing, China) were maintained under specific pathogen-free conditions. Treated HL-60 cell suspensions (1 × 10^7^ cells) were mixed 1:1 with Matrigel (BD356234, Corning, USA) and injected subcutaneously into the right axillae of nude mice. Quantification of immunohistochemical staining was performed using Image-Pro Plus 6.0 software. Tumor volume was calculated as follows: (longest diameter) × (shortest diameter)^2^ × (π/6).

### Statistical analysis

Statistical analyses were performed as indicated using R language (version 3.5.2) or GraphPad Prism (version 9.0.0). Two-tailed Student’s *t* tests were performed for comparisons between two groups. Categorical data were assessed by the chi-square test. Survival curves were constructed using the Kaplan–Meier method. Survival data were evaluated by univariate and multivariate Cox regression analyses. Bioinformatic approaches of Gene Ontology (GO) analysis, Kyoto Encyclopedia of Genes and Genomes (KEGG) analysis, and gene set enrichment analysis (GSEA) were performed using the OmicStudio tools (https://www.omicstudio.cn/tool). The potential m6A sites were predicted using an online tool, SRAMP (http://www.cuilab.cn/sramp/). *P* < 0.05 was considered statistically significant.

## Results

### High-throughput library screening identifies IGF2BP3 as a core m6A regulator in AML

**Fig. 1:**
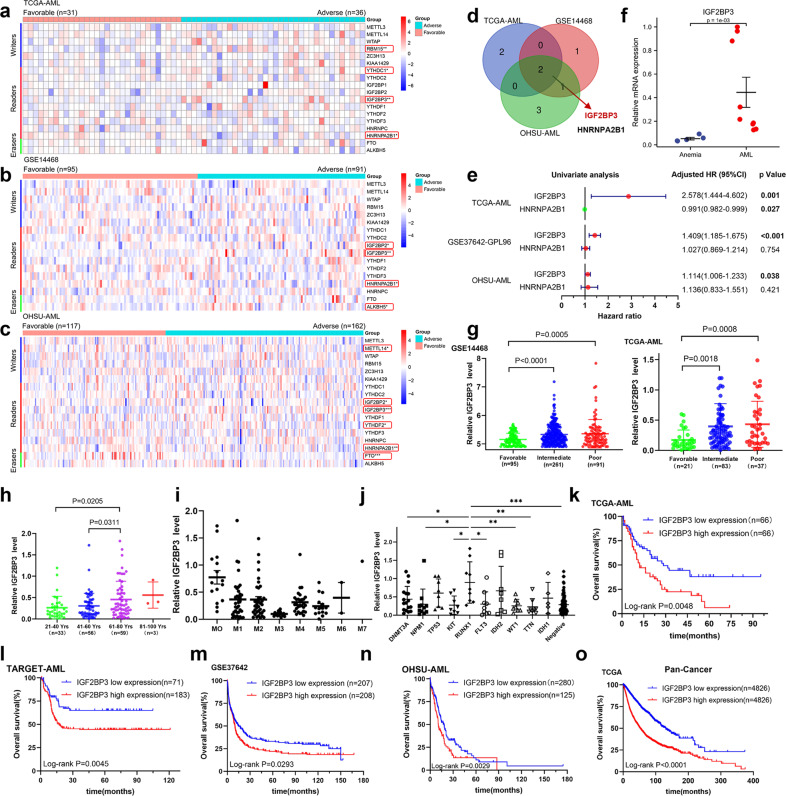
High-throughput library screening identifies IGF2BP3 as a core m6A regulator in AML. **a**–**c** Heatmap representation of transcriptome array data for the expression levels of m6A-associated regulators in AML from the TCGA-AML, GSE14468, and OHSU-AML datasets. **d** Both IGF2BP3 and HNRNPA2B1 are significantly differentially regulated m6A functional effectors in different datasets. **e** A forest plot showing the HR and 95% CI for the association between IGF2BP3 and HNRNPA2B1 candidate genes and overall survival times in patients with AML calculated by univariate Cox regression analysis. **f** Real-time PCR was used to determine the expression of IGF2BP3 in bone marrow specimens from patients with AML. **g** The aberrantly high expression of IGF2BP3 in AML patients was significantly correlated with more unfavorable clinical characteristics on measures such as cytogenetic risk stratification. **h** Expression of IGF2BP3 mRNA with increasing age. **i**, **j** IGF2BP3 expression was lowest in patients with the AML-M3 subtype (French-American-British classification) and higher in patients with RUNX1 mutation. **k**–**n** Kaplan–Meier survival analysis showed that AML patients with high IGF2BP3 expression exhibited worse overall survival, based on analysis of TCGA, TARGET, GSE37642, and OHSU-AML datasets. **o** Pancancer analysis of 9652 tumor patients in the TCGA cohort showed that high expression of IGF2BP3 was strongly associated with poor prognosis. *P < 0.05; **P < 0.01; ***P < 0.001.

To comprehensively investigate the roles of the m6A-associated genes in AML development, we first analyzed the expression levels of these genes by surveying publicly available TCGA-AML, GSE14468, and OHSU-AML datasets (Fig. 1a–c). To eliminate the effects of tissue specificity, a Venn diagram was generated, and the intersection shows the number of overlapping genes. IGF2BP3 and HNRNPA2B1 were significantly differentially regulated m6A factors in different datasets (Fig. 1d). To further filter candidate molecules, a forest plot showing the results of univariate Cox regression analysis for IGF2BP3 and HNRNPA2B1 was generated (Fig. 1e). In the TCGA-AML cohort, high expression of IGF2BP3 was associated with shorter overall survival times in patients with AML (HR = 2.578, 95% CI = 1.444–4.602, *P* = 0.001), and high expression of HNRNPA2B1 was associated with longer overall survival times in patients with AML (HR = 0.991, 95% CI = 0.982–0.999, *P* = 0.027). In the GSE37642 cohort, high expression of IGF2BP3 was a risk factor for AML (HR = 1.409, 95% CI = 1.185–1.675, *P* < 0.001), whereas HNRNPA2B1 was not (*P* = 0.754). In the OHSU-AML cohort, high expression of IGF2BP3 was associated with poor overall prognosis of AML (HR = 1.114, 95% CI = 1.006–1.233, *P* = 0.038), and HNRNPA2B1 expression was not associated with overall survival in patients with AML (*P* = 0.421). Kaplan–Meier survival analysis showed that HNRNPA2B1 was not associated with prognosis in patients with AML (Supplementary Fig. 1a–f). We then verified these in silico findings in bone marrow specimens from patients with AML by RT–qPCR. Indeed, IGF2BP3 mRNA expression was significantly increased in AML patients (*P* = 0.001) (Fig. 1f). The clinical characteristics of the patients included in the study are shown in Supplementary Table 2. The relationships between the clinical and molecular characteristics with the IGF2BP3 expression level in patients with AML are listed in Table 1. Additionally, we used the external database Oncomine to analyze the expression of IGF2BP3 in AML patients/samples, and the expression of IGF2BP3 was observed to be upregulated compared with that in the normal controls in the Andersson Leukemia and Haferlach Leukemia cohorts (Supplementary Fig. 2a–c).

**Table 1:** Comparison of clinical and molecular characteristics with the IGF2BP3 expression status in patients with AML.

Characteristic	Total	IGF2BP3-low (*n* = 75)	IGF2BP3-high (*n* = 76)	*P* value
**Age/years, median (range)**		53 (21–78)	59 (21–88)	0.0296^*^
**Age group/*n*** **(%)**				0.1611^§^
<60 years	84	46 (61.3%)	38 (50%)	
≥60 years	67	29 (38.7%)	38 (50%)	
**Sex/*n*** **(%)**				0.1628^§^
Male	82	45 (60%)	37 (48.7%)	
Female	69	30 (40%)	39 (51.3%)	
**WBC/×10**^**9**^**/L, median (range)**		16.0 (0.4–223.8)	18.8 (0.6–202.7)	0.6689^*^
**BM blasts/%, median (range)**		72 (30–100)	69.5 (32–98)	0.3832^*^
**PB blasts/%, median (range)**		39 (097)	45 (0–97)	0.7409^*^
**FAB subtype/*n*** **(%)**				
M0	15	2 (2.7%)	13 (17.1%)	**0.0030**^**§**^
M1	36	18 (24%)	18 (23.7%)	0.9637^§^
M2	37	17 (22.7%)	20 (26.3%)	0.6022^§^
M3	15	15 (20%)	0 (0%)	**0.0000**^**§**^
M4	29	11 (14.7%)	18 (23.7%)	0.1596^§^
M5	15	10 (13.3%)	5 (6.6%)	0.1653^§^
M6	2	1 (1.3%)	1 (1.3%)	0.9925^§^
M7	1	0 (0%)	1 (1.3%)	0.3189^§^
Other	1	1 (1.3%)	0 (0%)	0.3125^§^
**Cytogenetics/*n*** **(%)**				
Normal	60	24 (32%)	36 (47.4%)	0.0537^§^
Complex karyotype	18	8 (10.7%)	10 (13.2%)	0.6367^§^
PML-RARA	15	15 (20%)	0 (0%)	**0.0000**^**§**^
RUNX1-RUNX1T1	7	7 (9.3%)	0 (0%)	**0.0064**^**§**^
CBFB-MYH11	10	3 (4%)	7 (9.2%)	0.1980^§^
MLL translocation	8	6 (8%)	2 (2.6%)	0.1409^§^
Other	33	12 (16%)	21 (27.6%)	0.0838^§^
**Risk_Cyto/*n*** **(%)**				
Favorable	31	24 (32%)	7 (9.2%)	**0.0005**^**§**^
Intermediate	81	31 (41.3%)	50 (65.8%)	**0.0026**^**§**^
Poor	36	19 (25.3%)	17 (22.4%)	0.6690^§^
Other	3	1 (1.3%)	2 (2.6%)	0.5676^§^
**Survival status/*n*** **(%)**				**0.0055**^**§**^
Deceased	97	40 (53.3%)	57 (75%)	
Living	54	35 (46.7%)	19 (25%)	

Bold values identify statistical significance (*p* < 0.05)

*WBC* white blood cell, *BM* bone marrow, *PB* peripheral blood, *FAB* French-American-British.

*Mann–Whitney *U* test; ‘§’ denotes chi-square test. “Complex karyotype” is defined as the presence of more than or equal to 3 chromosomal abnormalities.

Subsequently, the associations between IGF2BP3 expression and clinical parameters were assessed, and aberrantly high expression of IGF2BP3 in AML patients was significantly correlated with more unfavorable clinical characteristics on measures such as cytogenetic risk stratification (Fig. 1g), age (Fig. 1h), FAB (French-American-British) subtype (Fig. 1i) and molecular subtype (Fig. 1j). Of note, IGF2BP3 expression was the lowest in patients with the AML-M3 subtype and higher in patients with RUNX1 mutation. Kaplan–Meier survival analysis also showed that AML patients with high IGF2BP3 expression exhibited worse overall survival, and we obtained consistent results with multiple datasets (Fig. 1k–n). In parallel with this study, a pancancer analysis of 9652 tumor patients in the TCGA cohort showed that high expression of IGF2BP3 was strongly associated with poor prognosis (*P* < 0.001, Fig. 1o). The clinical variables used for the univariate and multivariate analyses are listed in Table 2. The results identified the expression of IGF2BP3 and risk stratification as independent prognostic factors of AML. Intriguingly, high expression of IGF2BP3 was associated with a poor prognosis not only in hematologic malignances but also in other solid tumors, such as colon cancer, uveal melanoma, mesothelioma, lung adenocarcinoma, glioma, renal papillary cell carcinoma, and renal clear cell carcinoma (Supplementary Fig. 3a–h). Taken together, these data further shed light on the oncogenic role of IGF2BP3 in tumor progression.

**Table 2:** Univariate and multivariate analysis of the relationship between IGF2BP3 expression and overall survival in patients with AML.

	Univariate analysis			Multivariate analysis		
Variable	HR	95% CI	*P* value	HR	95% CI	*P* value
**Sex** (male vs. female)	1.01	0.68–1.51	0.9465			
**WBC** ( > 50*109/L vs.<=50*10^9^/L)	1.47	0.94–2.32	0.0916	1.39	0.84–2.31	0.1956
**BM_blast** (>90% vs.<=90%)	0.89	0.46–1.72	0.7345			
**PB_blast** (>50% vs.<=50%)	1.22	0.82–1.84	0.3273			
**Risk_Cyto** (poor vs. intermediate/good)	3.43	1.78–6.63	**0.0002**	2.72	1.37–5.4	**0.0043**
**Complex_Cyto** (yes vs. no)	1.91	1.08–3.37	**0.0254**	1.71	0.9–3.22	0.0992
**IGF2BP3_expression** (high vs. low)	1.89	1.25–2.83	**0.0023**	1.58	1.04–2.4	**0.0319**

Bold values identify statistical significance (*p* < 0.05)

*WBC* white blood cell, *BM* bone marrow, *PB* peripheral blood, *HR* hazard ratio, *CI* confidence interval

Variables with *P* < 0.1 in the univariate analysis were included in the multivariate analysis.

### Knockdown of IGF2BP3 significantly inhibits AML progression in vitro

**Fig. 2:**
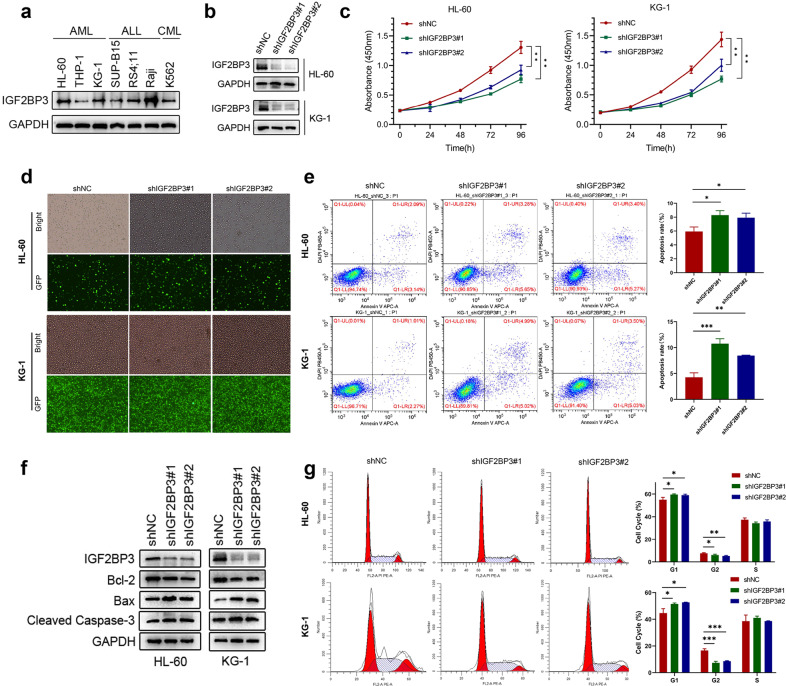
Knockdown of IGF2BP3 significantly inhibits AML progression in vitro. **a** The protein expression level of IGF2BP3 in various hematologic tumor cell lines was measured by western blotting. **b** The knockdown efficiency of IGF2BP3 shRNAs (shIGF2BP3#1 and shIGF2BP3#2) delivered via lentiviral vectors in HL-60 and KG-1 cell lines was confirmed by western blotting. GAPDH was used as the internal reference. **c** Cell proliferation was measured by a CCK-8 assay at different time points (0, 24, 48, 72, and 96 h) in HL-60 and KG-1 cells after shRNA transduction. **d** The transduction efficiency after puromycin selection was evaluated by GFP fluorescence imaging in both cell lines. **e** Flow cytometry (representative images are presented) was used to confirm the induction of apoptosis by IGF2BP3 knockdown. **f** Western blotting was used to explore apoptosis-related protein levels. The levels of cleaved caspase-3 and Bax were increased but the level of Bcl-2 was decreased under shIGF2BP3 treatment compared with control treatment. **g** Flow cytometry (representative images are presented) was used to analyze the cell cycle distribution. *P < 0.05; **P < 0.01; ***P < 0.001.

To explore the potential role of IGF2BP3 in AML, we measured the protein expression level of IGF2BP3 in various hematologic tumor cell lines (Fig. 2a). First, we investigated the effects of knocking down IGF2BP3 by generating two shRNA-expressing stable human AML cell lines, HL-60 and KG-1, which displayed relatively high IGF2BP3 expression among all tested AML cell lines. Both shRNAs (shIGF2BP3#1/#2) markedly suppressed IGF2BP3 expression at the protein level (Fig. 2b). GFP fluorescence imaging was used to verify the transduction efficiency after puromycin selection (Fig. 2d). IGF2BP3 knockdown indeed decreased the proliferation of AML cells (Fig. 2c). Additionally, IGF2BP3 knockdown significantly promoted apoptosis in leukemia cells, as evidenced by the higher percentage of Annexin V + cells (Fig. 2e). Immunoblot analysis showed that the level of the antiapoptotic protein Bcl-2 was slightly decreased and the levels of proapoptotic proteins (Bax and cleaved Caspase 3) were significantly increased (Fig. 2f). Moreover, cell cycle analysis showed that inhibition of IGF2BP3 in AML cells increased the proportion of G1-phase cells and decreased the proportion of G2-phase cells (Fig. 2g). These observations indicate a potential role for IGF2BP3 in the pathogenesis of AML.

### Knockdown of IGF2BP3 decreases AML cell viability in vivo

**Fig. 3:**
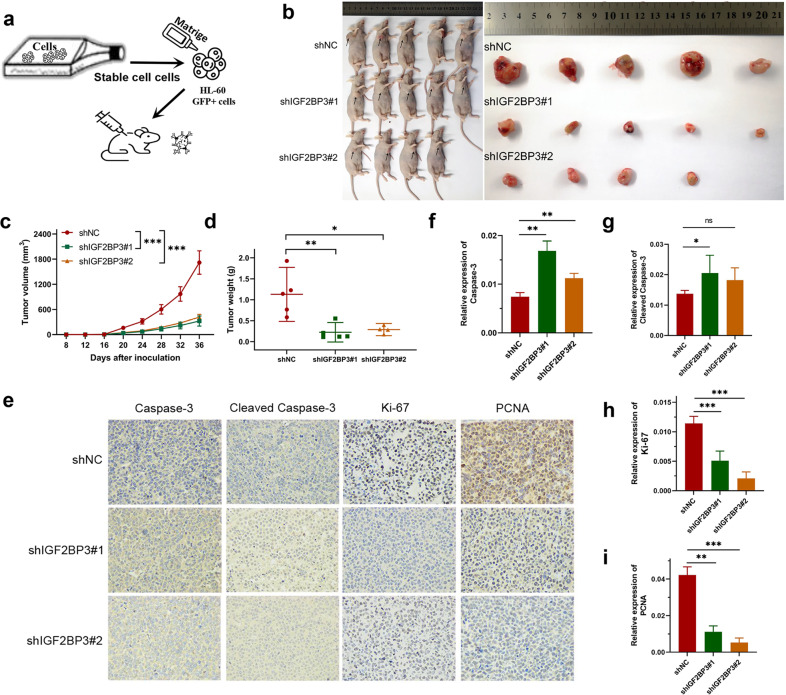
Knockdown of IGF2BP3 decreases AML cell viability in vivo. **a** Schematic diagram showing the schedule of nude mouse xenograft assays. **b** Subcutaneous tumors were observed at 36 days in two different groups (the black arrows indicate xenograft tumors). **c** The xenograft growth curves for the shIGF2BP3#1, shIGF2BP3#2, and shNC groups were plotted by measuring the tumor size (width^2^ × length × π/6) with a Vernier caliper every four days. **d** Nude mice were sacrificed, and xenografts were harvested and weighed. **e**–**i** Representative images of immunohistochemical staining for Caspase-3, cleaved Caspase-3, Ki-67, and PCNA in tumors excised from xenograft model mice. *P < 0.05; **P < 0.01; ***P < 0.001; ns, not significant.

The HL-60 cell line shIGF2BP3 generated via a lentivirus-based shRNA technique exhibited stable knockdown of IGF2BP3. Matrigel and cell suspension mixtures were used to establish an in vivo xenograft model (Fig. 3a). During the observation of flank xenografts in BALB/c nude mice for 36 days, a reduced IGF2BP3 level was observed to result in delayed progression of engrafted HL-60 tumors, as the volume of IGF2BP3-deficient tumors was significantly decreased compared with that of control tumors (Fig. 3b, c). The nude mice were sacrificed, and the xenografts were harvested and weighed. The tumor weights in the two groups were significantly different (Fig. 3d). Accordingly, the proliferation markers Ki-67 and PCNA were downregulated and the apoptotic marker Caspase-3 was upregulated in IGF2BP3-deficient tumors (Fig. 3e–i). In summary, IGF2BP3 knockdown also dramatically decreased AML cell viability in vivo.

### Overexpression of IGF2BP3 promoted the proliferation and tumorigenesis of AML

**Fig. 4:**
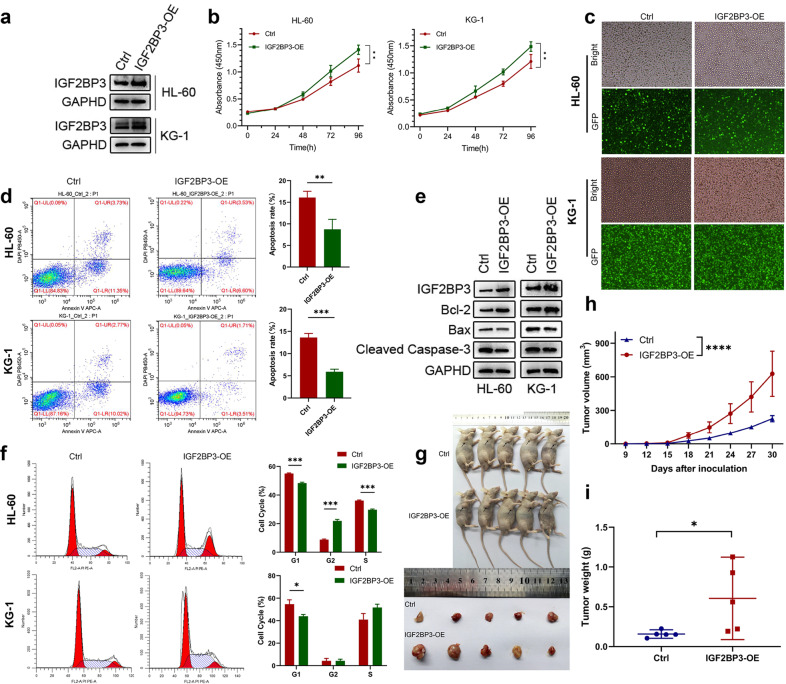
Overexpression of IGF2BP3 promoted the proliferation and tumorigenesis of AML cells. **a** IGF2BP3 overexpression with lentiviral constructs in HL-60 and KG-1 cell lines was confirmed by western blotting. GAPDH was used as the internal reference. **b** Cell proliferation was measured by a CCK-8 assay at different time points (0, 24, 48, 72, and 96 h) in HL-60 and KG-1 cells. **c** The transduction efficiency after puromycin selection was evaluated by GFP fluorescence imaging in both cell lines. **d** Flow cytometry (representative images are presented) was used to confirm the induction of apoptosis by overexpression of IGF2BP3. **e** Western blotting was used to explore apoptosis-related protein levels. **f** Flow cytometry (representative images are presented) was used to analyze the cell cycle distribution. **g** Subcutaneous tumors were observed at 30 days in two different groups (the black arrows indicate xenograft tumors), and the excised tumors were photographed and weighed. **h** The xenograft growth curves for the IGF2BP3-OE and Ctrl groups were plotted by measuring the tumor size with a Vernier caliper every three days. **i** Nude mice were sacrificed, and xenografts were harvested and weighed. *P < 0.05; **P < 0.01; ***P < 0.001; ****P < 0.0001.

To further confirm the function of IGF2BP3 in AML, we determined the effect of IGF2BP3 overexpression on the proliferation of these AML cells both in vitro and in vivo (Fig. 4a). GFP fluorescence imaging was used to verify the transfection efficiency (Fig. 4c). The CCK-8 assay results indicated that overexpression of IGF2BP3 significantly promoted cell growth compared with that in the control group (Fig. 4b). Furthermore, flow cytometric analysis showed that IGF2BP3 overexpression decreased the apoptosis of leukemia cells and that the percentage of Annexin V + cells was reduced (Fig. 4d). Consistent with these observations, the expression of antiapoptotic Bcl-2 protein was significantly increased, and the protein levels of Bax and cleaved Caspase 3 were greatly decreased (Fig. 4e). Cell cycle analysis was conducted by FCM and showed a reduction in the G1 phase population of cells overexpressing IGF2BP3 (Fig. 4f). Similar results were obtained in a xenograft mouse model of AML, in which stable IGF2BP3-overexpressing (IGF2BP3-OE) cells and control (Ctrl) cells were injected into the flanks of male nude mice. Tumor size was measured every three days. All mice were sacrificed 30 days after tumor inoculation, and the excised tumors were photographed and weighed (Fig. 4g). The growth curve of the xenograft tumors showed that overexpression of IGF2BP3 accelerated tumor growth (Fig. 4h), and there were significant differences in the xenograft weights after excision (Fig. 4i). Representative images of immunohistochemical staining for Caspase-3, cleaved Caspase-3, Ki-67, and PCNA in the excised tumors are provided in Supplementary Fig. 4. Taken together, these results suggest that IGF2BP3 plays an important role in the proliferation and apoptosis of AML cells.

### Identification of the IGF2BP3 targets in AML

**Fig. 5:**
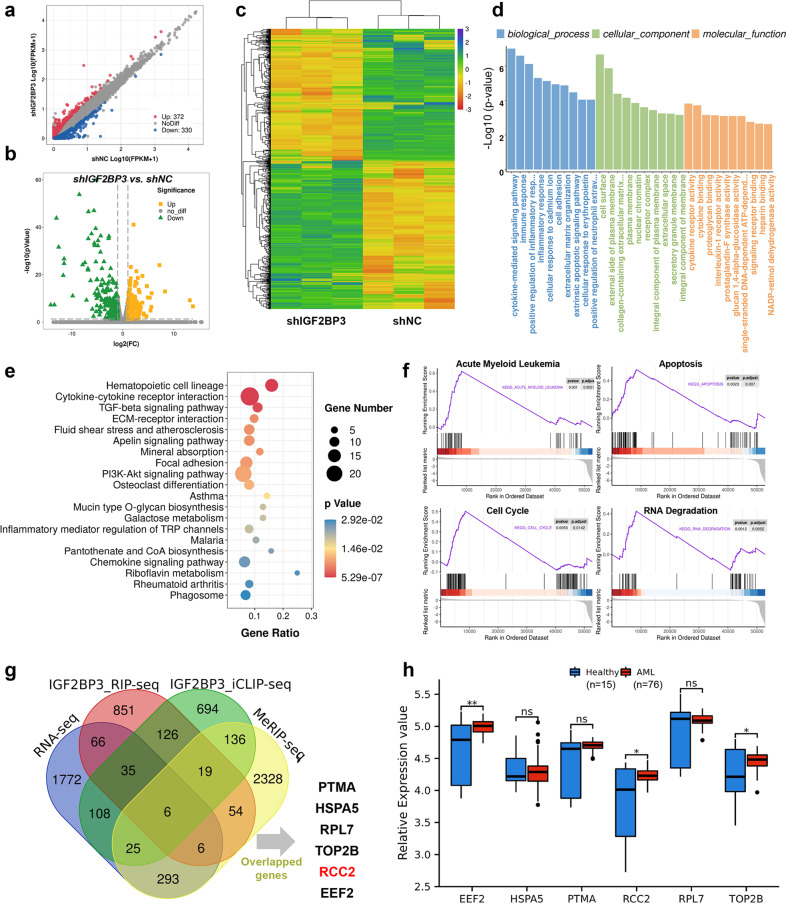
Identification of the potential IGF2BP3 targets in AML. **a** Scatter plot of differentially expressed genes. The values on the X and Y axes in the scatter plot are the average FPKM values of each group (log10 scale). The dots above the top line (372 red dots, upregulated in the shIGF2BP3 group) or below the bottom line (330 blue dots, upregulated in the shNC group) indicate genes with a change in expression of more than 2-fold between the two comparison groups. **b** Volcano plot of differentially expressed genes. The values on the X and Y axes in the volcano plot are the fold change (log2 transformed) values and *P* values (−log10 transformed) between the two groups, respectively. The green/yellow dots indicate differentially expressed genes with a statistically significant change in expression of greater than 2-fold. **c** Heatmap generated from the RNA-seq data showing the representative genes after IGF2BP3 knockdown. **d**, **e** Gene Ontology (GO) and Kyoto Encyclopedia of Genes and Genomes (KEGG) analyses revealed the potential roles of differentially expressed genes following IGF2BP3 knockdown in HL-60 cells. **f** Representative GSEA results showed that apoptosis, the cell cycle, and RNA degradation were closely correlated with IGF2BP3 expression. **g** Venn diagram showing that 6 candidate genes—PTMA, HSPA5, RPL7, TOP2B, RCC2, and EEF2—overlapped with the predicted direct targets of IGF2BP3. **h** The expression differences in candidate genes were analyzed from GSE12662, and EEF2, RCC2, and TOP2B were verified to be highly expressed. *P < 0.05; **P < 0.01; ***P < 0.001.

To explore the underlying mechanisms of IGF2BP3 in AML development, we first performed RNA-seq analysis with IGF2BP3 knockdown and control HL-60 cells, each group consisting of three biological replicates. The scatter plot, volcano plot and heatmap indicated that IGF2BP3 depletion resulted in 702 genes being altered globally, specifically, 372 genes were upregulated and 330 were downregulated (Fig. 5a–c). More details are provided in Supplementary Table 3. GO analysis indicated that those differentially expressed genes were enriched in the terms cytokine-mediated signaling pathway, immune response, extrinsic apoptotic signaling pathway, and cytokine binding (Fig. 5d and Supplementary Table 4). The KEGG results elucidated the potential biological functions of IGF2BP3 in AML. The top 20 significantly enriched pathways are shown in Fig. 5e. Among these pathways, the hematopoietic cell lineage, TGF-beta signaling pathway, PI3K-Akt signaling pathway, and chemokine signaling pathway were linked with the progression of AML^17–19^. Full results from this analysis are presented in Supplementary Table 5. To identify pathways that may be altered by IGF2BP3 knockout, our RNA-seq data were subjected to GSEA (Supplementary Table 6). Representative results showed that apoptosis, the cell cycle, and RNA degradation were closely correlated with IGF2BP3 expression (Fig. 5f). This is consistent with the previous finding that IGF2BP3 mainly regulates apoptosis.

Since IGF2BP3 is a well-known m6A-specific reader protein^20^, we then intended to identify potential transcripts with m6A modification that are regulated by IGF2BP3 using MeRIP-seq data from GSE87515^21^. We next screened for IGF2BP3-binding genes by using RIP-seq data from the previously published dataset GSE90639^20^. Furthermore, iCLIP-seq data from GSE76931 were combined with our RNA-seq data to identify reliable target candidate genes^22^. The Venn diagram showed that 6 candidate genes—PTMA, HSPA5, RPL7, TOP2B, RCC2, and EEF2—overlapped with the predicted direct targets of IGF2BP3 (Fig. 5g). The expression differences in the candidate genes were analyzed in GSE12662, and EEF2, RCC2, and TOP2B were verified to be highly expressed in AML (Fig. 5h). By reviewing the literature, we selected RCC2 as the target gene of IGF2BP3 for subsequent analysis.

### IGF2BP3 regulates RCC2 expression in an m6A-dependent manner

**Fig. 6:**
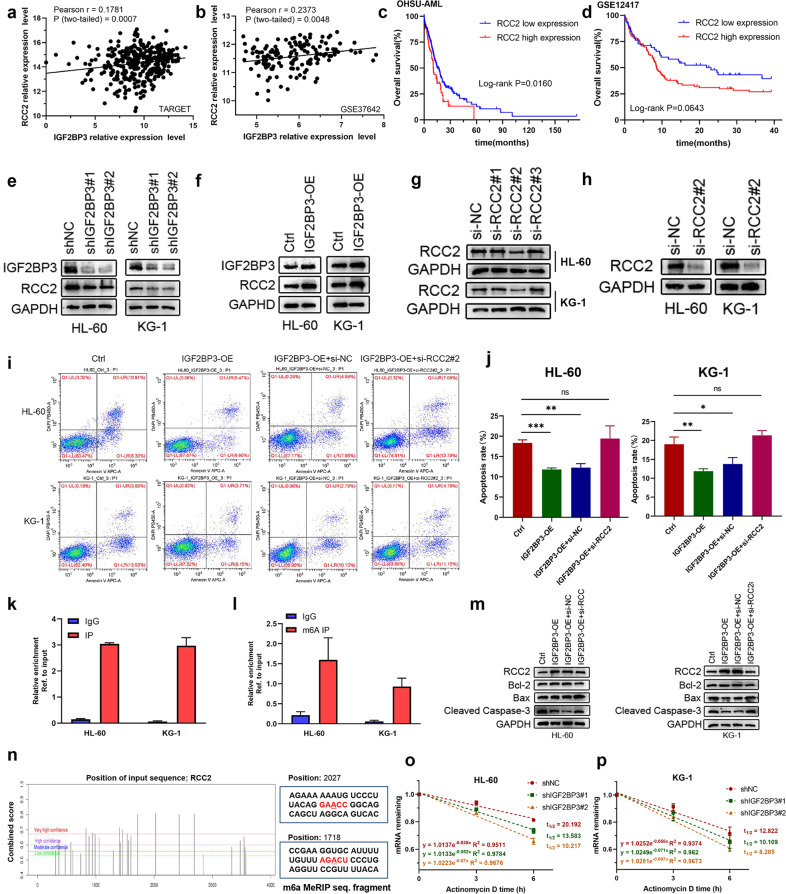
IGF2BP3 regulates RCC2 expression in an m6A-dependent manner. **a**, **b** RCC2 expression was positively correlated with IGF2BP3 expression in the GSE37642 and TARGET datasets. **c**, **d** Kaplan–Meier survival analysis revealed that high RCC2 expression indicated a poor prognosis in AML patients. **e**, **f** Protein expression level of RCC2 following knockdown or overexpression of IGF2BP3 in HL-60 and KG-1 cells. **g**, **h** The interference efficiency of the siRNAs was evaluated to confirm the feasibility of the siRNAs, and si-RCC2#2 was found to be effective in reducing RCC2 expression. **i**, **j** Apoptosis was detected by flow cytometry. RCC2 deficiency promoted the induction of apoptosis by IGF2BP3 overexpression. **k** The mRNA of RCC2 was enriched by the anti-IGF2BP3 antibody compared to IgG in the HL-60 and KG-1 cell lines. **l** The mRNA of RCC2 was enriched by the m6A-specific antibody compared to IgG in the HL-60 and KG-1 cell lines. **m** Overexpression of IGF2BP3 restored the increases in the levels of proapoptotic proteins (Bax and cleaved Caspase 3) caused by silencing RCC2, and the level of the antiapoptotic protein Bcl-2 was slightly decreased. **n** The potential m6A sites in RCC2 were predicted by SRAMP. The different colored lines indicate different confidence levels. **o**, **p** Loss of IGF2BP3 reduced RCC2 stability in HL-60 and KG-1 cells. Transfected cells were treated with 5 µg/ml actinomycin D for 0 h, 3 h, or 6 hours prior to RNA extraction. *P < 0.05; **P < 0.01; ***P < 0.001.

To investigate the regulatory relationship between IGF2BP3 and RCC2 and the specific mechanisms of RCC2 in AML, we first detected the correlation between these genes in the GSE37642 and TARGET databases, and the results suggested that RCC2 expression was positively correlated with IGF2BP3 expression (Fig. 6a, b). Additionally, Oncomine database analysis showed high expression of RCC2 in AML (Supplementary Fig. 2d, e). Interestingly, we analyzed the significance of RCC2 in terms of clinical prognosis by Kaplan–Meier survival analysis, and the results revealed that high RCC2 expression indicated poor prognosis in AML patients (Fig. 6c, d). We found that RCC2 was significantly downregulated after IGF2BP3 knockdown in both the HL-60 and KG-1 cell lines and, similarly, overexpression of IGF2BP3 enhanced the expression of RCC2 (Fig. 6e, f, Supplementary Fig. 5c). The interference efficiency of the siRNAs was first evaluated to confirm the feasibility of the siRNAs (Fig. 6g), and si-RCC2#2 was found to be effective in reducing RCC2 expression (Fig. 6h). Notably, we observed an increase in the proportion of apoptotic cells in the si-RCC2#2 groups compared to the control groups, which confirmed that depletion of RCC2 enhanced apoptosis in HL-60 and KG-1 cells (Supplementary Fig. 5a, b). We next explored the role of the IGF2BP3/RCC2 axis in AML and performed rescue experiments. RCC2 deficiency promoted the induction of apoptosis by IGF2BP3 overexpression (Fig. 6i–j). Concomitantly, overexpression of IGF2BP3 restored the increases in the levels of the proapoptotic proteins (Bax and cleaved Caspase 3) caused by silencing RCC2, and the level of the antiapoptotic protein Bcl-2 was slightly decreased (Fig. 6m). RNA enrichment was evaluated through RIP and RT–qPCR, and the results validated that the mRNA of RCC2 was enriched by the anti-IGF2BP3 antibody compared with IgG in the HL-60 and KG-1 cell lines (Fig. 6k), which confirmed the direct interaction between IGF2BP3 and RCC2. Moreover, the m6A site prediction tool SRAMP was used^23^, and distinct m6A sites in RCC2 at single-base resolution were identified (Fig. 6n). To investigate whether gene expression affects m6A modification, MeRIP-RT–qPCR was performed, and the results indicated RCC2 mRNA enrichment in the m6A-specific antibody precipitate (Fig. 6l). We investigated whether IGF2BP3 regulates RCC2 expression by modulating its mRNA stability in leukemia cells. Indeed, the loss of IGF2BP3 caused an obvious decrease in the half-life of RCC2 mRNA (Fig. 6o–p). Collectively, these findings indicate that IGF2BP3 mediates the degradation of RCC2 mRNA by reading m6A-modified sites and that this regulation is m6A-dependent.

## Discussion

As the most abundant RNA epigenetic modification, the dynamic and reversible m6A has been considered to play a crucial role in regulating various stages of diverse aspects of RNA function^24^. In addition, the most recent research studies have shown that dysregulation of m6A can affect normal hematopoiesis, leukemia cell proliferation, leukemia cell apoptosis, and leukemia stem/initiating cell (LSC/LIC) self-renewal^12,15,25,26^. RNA-binding proteins have a variety of functions, and their posttranscriptional regulation of gene expression is a major factor determining cancer progression and the therapeutic response^27^. IGF2BP3 is a carcinoembryonic protein that is usually expressed in fetal/embryonic tissue, but it is abnormally expressed in malignancies^28,29^. Although many studies have shown that IGF2BP3 is an oncogenic factor in various solid tumors, the role of IGF2BP3 and its paralogs in AML is not well understood. Several reports indicate that IGF2BPs are associated with leukemia. Elcheva et al. reported that inhibition of IGF2BP1 reduced the tumorigenicity of leukemia cells and promoted leukemia cell death and differentiation by regulating HOXB4, MYB, and ALDH1A1^30^. Studies have shown that IGF2BP3 overexpression in mouse progenitor cells directs hematopoietic cells toward the myeloid lineage^22^. Thus, IGF2BPs may have a unique role in stem and precursor lineage specification as well as in the myeloid lineage.

**Fig. 7:**
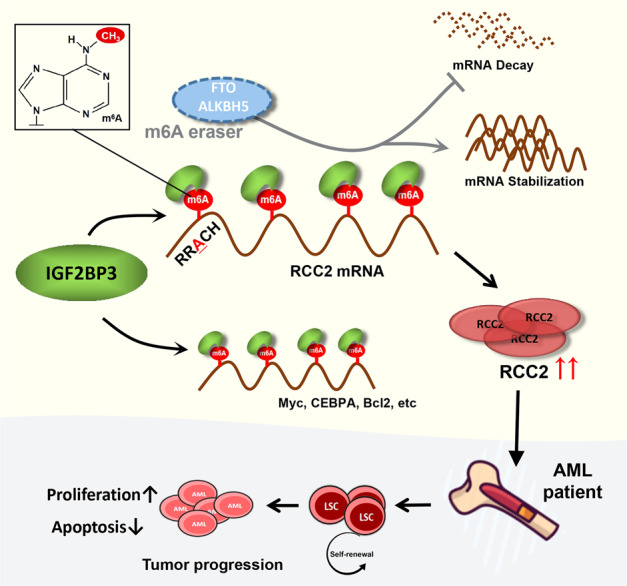
Schematic diagram showing the mechanism by which IGF2BP3 contributes to tumorigenesis and poor prognosis in AML through m6A RNA methylation. Model showing mechanism by which pro-oncogenic trigger may impair the cross-talk among m6A reader IGF2BP3, stabilize m6A-labeled genes and promote the abnormal accumulation of carcinogens (such as Myc, CEBPA, Bcl2, RCC2, etc.), thereby regulating the survival of leukemia cells and leading to AML progression.

In this study, we first analyzed AML high-throughput sequencing data and identified two potential m6A regulators, IGF2BP3 and HNRNPA2B1. Subsequently, univariate Cox regression analysis and survival analysis were performed in combination with clinical follow-up data, and IGF2BP3 expression was verified by analysis of bone marrow aspirate samples from patients; the findings preliminarily confirmed that IGF2BP3 plays a key role in the occurrence and development of AML. We then showed that IGF2BP3 is overexpressed in AML and plays an essential role in regulating leukemia cell proliferation in vivo and ex vivo. Previous studies have reported abnormal overexpression of the demethylases FTO and ALKBH5 in AML, and their increased expression has been associated with poor prognosis in AML patients^15,31^. One study showed that ALKBH5 selectively maintained the self-renewal of LSC/LIC but was not essential for normal hematopoiesis^15^. However, current studies on m6A reader proteins in AML predominantly focus on YTHDF2^32–34^, which mediates phenotypes by influencing target genes. To our knowledge, our study provides the first indication that IGF2BP3 is a tumorigenic RNA-binding protein that mediates the stabilization of RCC2 in an m6A-dependent manner in AML (Fig. 7). However, the detailed mechanisms of IGF2BP3 and other m6A regulators need to be investigated in the future.

Previous studies have suggested that knockdown of METTL14, FTO, ALKBH5, or YTHDF2 has a stronger inhibitory effect on leukemogenesis than on normal hematopoiesis and that m6A RNA methylation is important in the occurrence and progression of AML, suggesting that m6A regulators may serve as potential therapeutic targets for the eradication of leukemia cells. Through a literature review, we found that RCC2 has been reported to be an oncogene and that overexpression of RCC2 induced epithelial-mesenchymal transition to promote the proliferation, invasion, and migration of lung adenocarcinoma cells; RCC2 can also be driven by BRD4 to promote the growth of esophageal squamous cell carcinoma, although this has not been reported in leukemia^35–38^. Recently, FB23 and FB23-2, novel small molecule FTO inhibitors, were identified to selectively inhibit m6A demethylase activity and significantly inhibit the development of human AML cell lines and primary cells in xenograft mice^14^. In addition, CS1 and CS2 were shown to have more effective antileukemia effects with fewer side effects than the FTO inhibitors reported above^39^. Mechanistically, FTO targets the expression of immune checkpoint genes in an m6A-dependent manner via mechanisms such as affecting the stability of LILRB4 mRNA and being recognized by the reader protein YTHDF2, which significantly enhances AML cell sensitivity to T-cell cytotoxicity and thus overcomes immune escape^39^. Subsequently, we reviewed the RNA-seq data and found that LILRB4 was significantly downregulated after IGF2BP3 knockdown (*P* = 2.78E−45, log2FC = −6.76), which provided us with a deeper understanding of the mechanisms underlying RNA-mediated AML immune escape. Moreover, we described the increased expression of IGF2BP3 in patients with RUNX1 mutation, which may help us focus on targeted therapeutic strategies for patients with RUNX1 mutation.

However, this study has several limitations. First, the sample size of AML patients needs to be expanded for broader research. Second, m6A methylation site prediction and functional verification should be performed at multiple levels to confirm the IGF2BP3-dependent epigenetic regulatory mechanism of RCC2. Ultimately, these are important questions that should be addressed by future studies, especially in more animal studies, as the roles of IGF2BP3 and RCC2 in AML require further clarification.

In summary, we provided compelling in vitro and in vivo evidence demonstrating that the m6A reader IGF2BP3 contributes to the tumorigenesis and poor prognosis of AML by triggering downstream signaling cascades. The m6A binding proteins, such as IGF2BP3, should be further explored as biomarkers for leukemia type, as prognostic factors and, ultimately, as therapeutic targets in hematological malignancies.

## Supplementary information

Supplementary Information

## Data Availability

The datasets produced in this study are available in the Supplementary files. In addition, several user-friendly AML databases or tools were used to download, analyze, or reference data. TCGA (The Cancer Genome Atlas, https://portal.gdc.cancer.gov/), TARGET (Therapeutically Applicable Research to Generate Effective Treatment, https://ocg.cancer.gov/), OHSU (Oregon Health and Science University, accessed through https://www.cbioportal.org/) were used, and GEO (Gene Expression Omnibus, https://www.ncbi.nlm.nih.gov/geo/) data are accessible under accession numbers GSE37642, GSE14468, GSE12262, GSE71014, and GSE12662. Mutation data were downloaded from the TCGA website.
