# Supplementary material for: The m6A reader IGF2BP3 promotes acute myeloid leukemia progression by enhancing RCC2 stability
Source: Exp Mol Med. 2022 Feb 25;54(2):194–205. doi: 10.1038/s12276-022-00735-x (PMC8894383; doi:10.1038/s12276-022-00735-x)
Supplement: Supplementary file 1 — Supplementary Information [file 12276_2022_735_MOESM1_ESM.pdf]

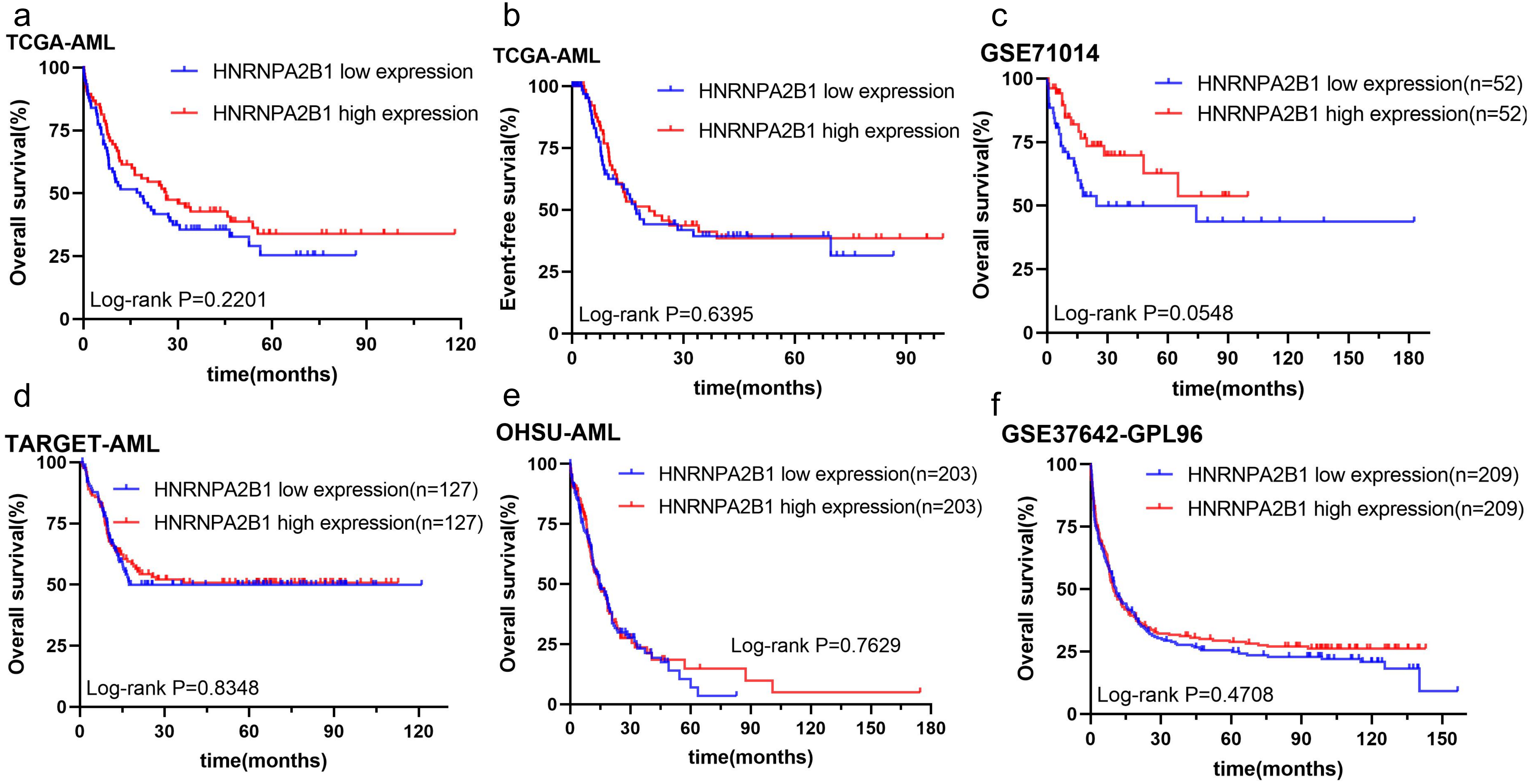

Supplementary Fig. 1. Kaplan-Meier survival analysis across multiple datasets showed that HNRNPA2B1 was not associated with prognosis in patients with AML. **(a-b)** TCGA database. **(c)** GSE71014 database. **(d)** TARGET database. **(e)** OHSU database. **(f)** GSE37642 database.

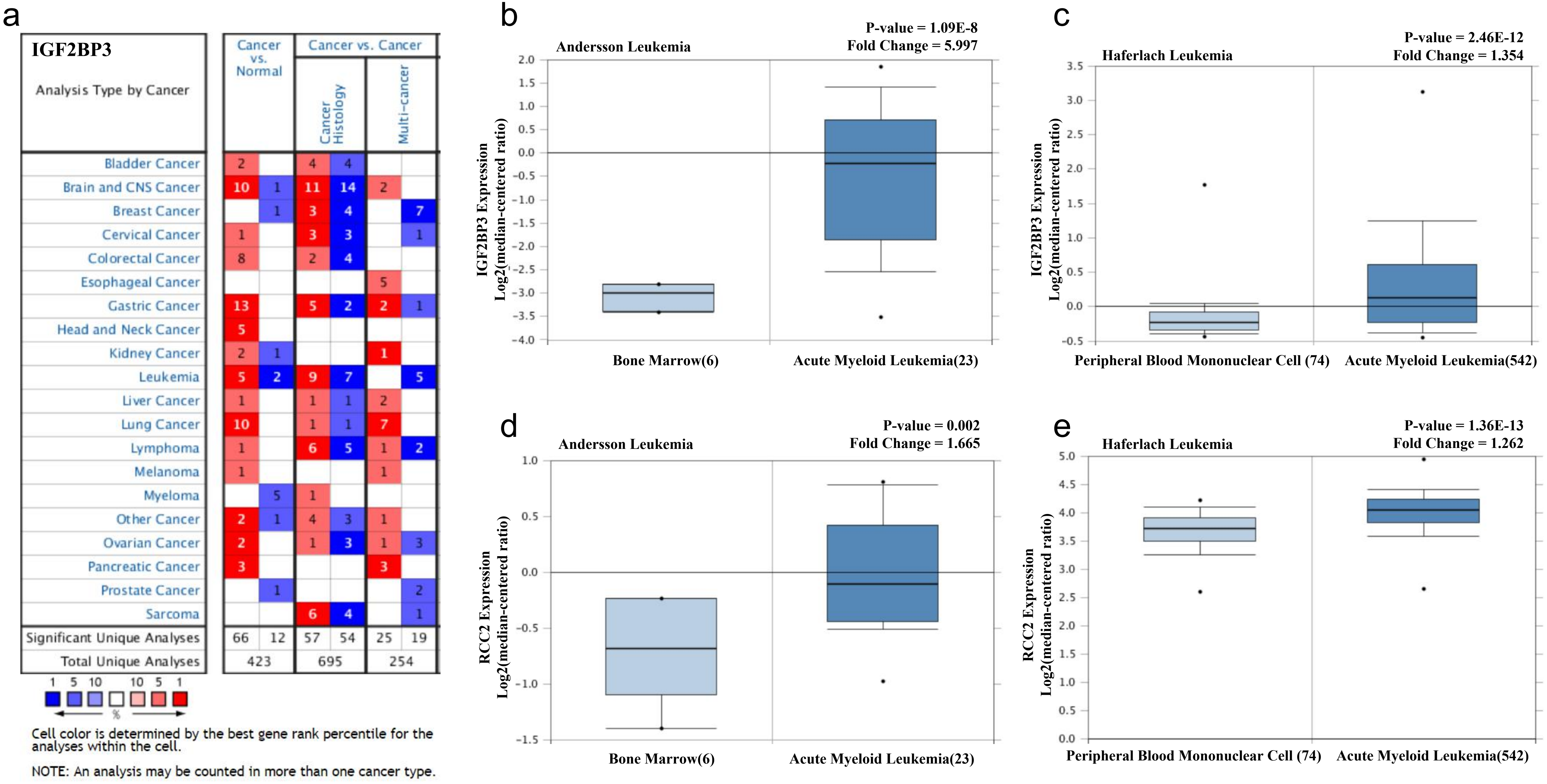

Supplementary Fig. 2. The expression of IGF2BP3 and RCC2 was analyzed in Oncomine database. **(a)** The Oncomine database was used to compare YAP1 expression in pan-tumors and corresponding normal tissues. **(b-c)** Upregulation of IGF2BP3 was observed compared with the normal controls in Andersson Leukemia and Haferlach Leukemia cohorts. **(d-e)** RCC2 high expression in Andersson Leukemia and Haferlach Leukemia cohorts.

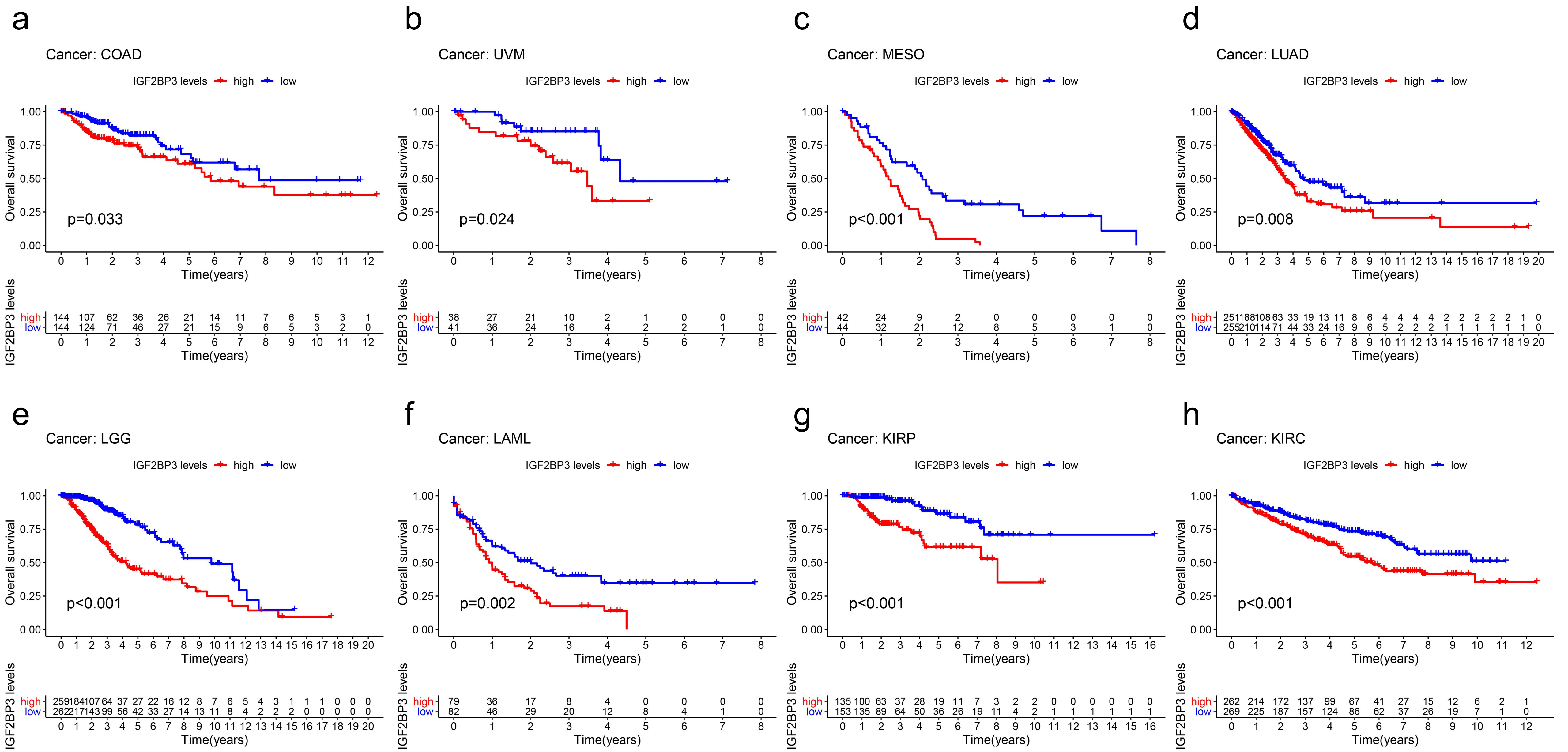

Supplementary Fig. 3. Survival analysis of IGF2BP3 in various tumors from the TCGA database. **(a)** Colon adenocarcinoma, COAD. **(b)** Uveal Melanoma, UVM. **(c)** Mesothelioma, MESO. **(d)** Lung adenocarcinoma, LUAD. **(e)** Brain Lower Grade Glioma, LGG. **(f)** Acute Myeloid Leukemia, LAML. **(g)** Kidney renal papillary cell carcinoma, KIRP. **(h)** Kidney renal clear cell carcinoma, KIRC.

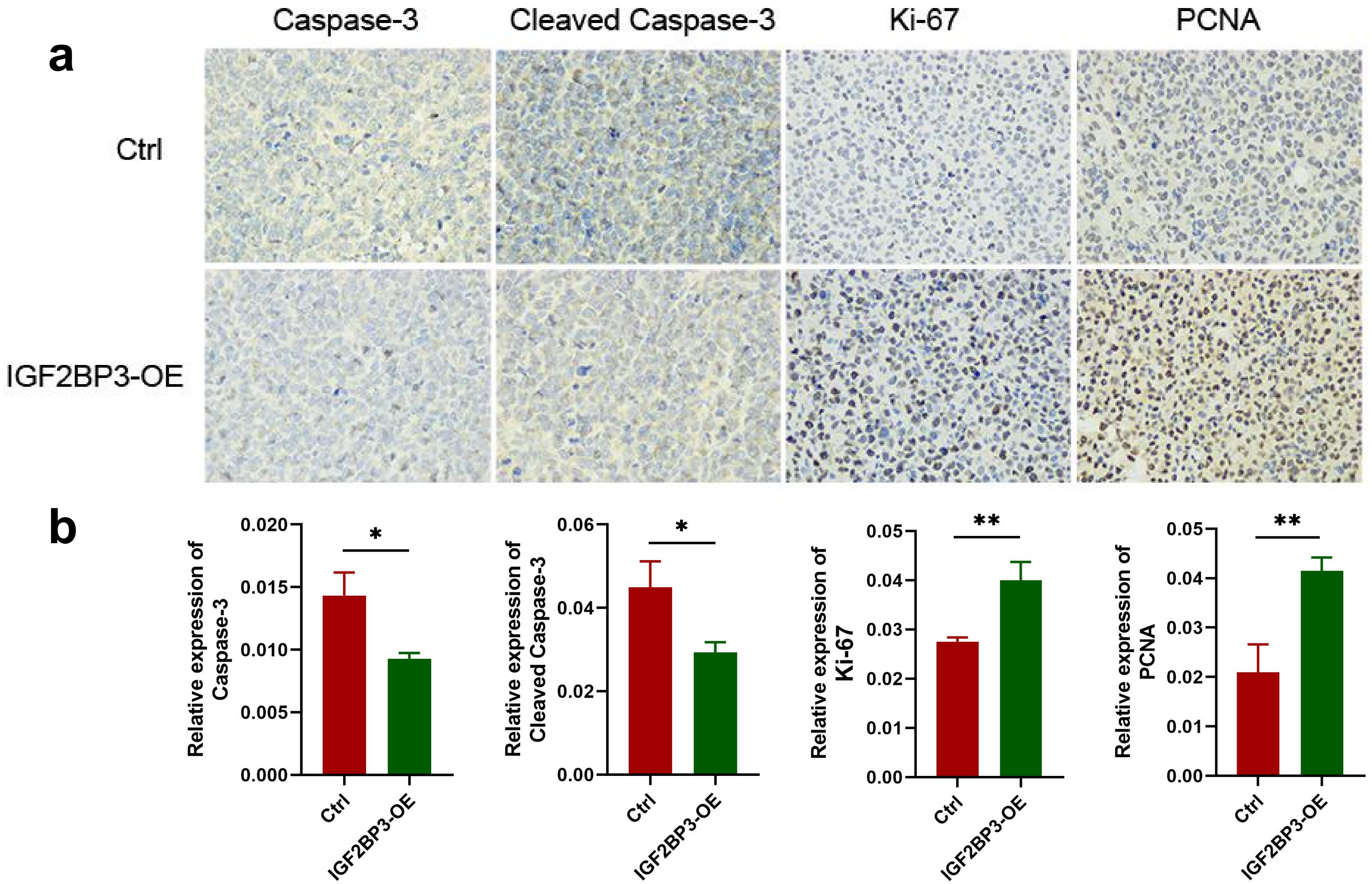

Supplementary Fig. 4. Representative immunohistochemical images of IGF2BP3 overexpression were provided **(a)**, and immunohistochemical intensity was scored **(b)**. *, $P < 0.05$; **, $P < 0.01$.

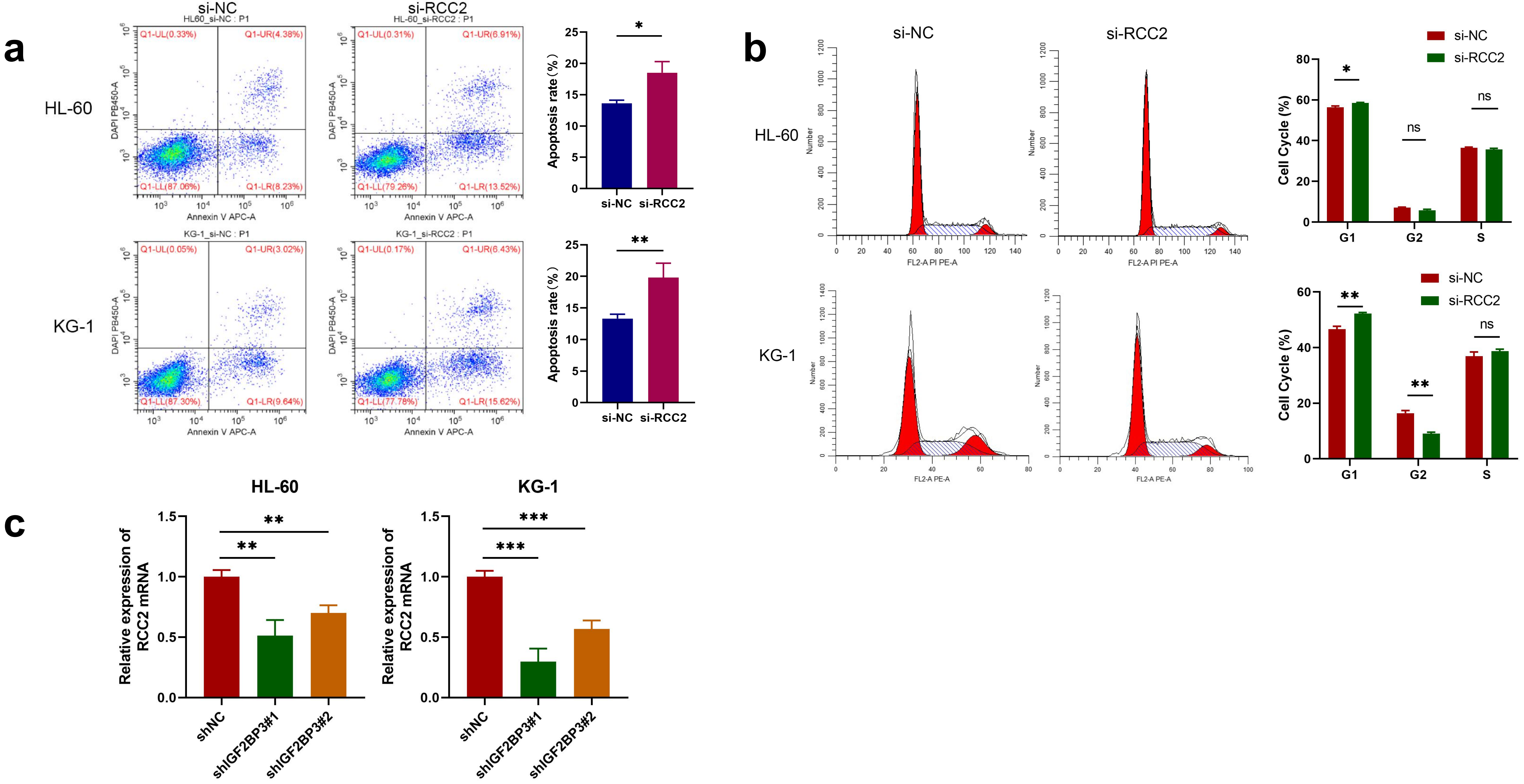

Supplementary Fig. 5. Effect of RCC2-siRNA on apoptosis of HL-60 and KG-1 cells. **(a)** RCC2 siRNA promotes apoptosis of AML cells. **(b)** Effect of RCC2 siRNA on AML cell cycle. **(c)** RCC2 mRNA expression levels following knockdown of IGF2BP3 in HL-60 and KG-1 cells. *, P < 0.05; **, P < 0.01; ***, P < 0.001.

Supplementary Table 1. Primers and sequences used in this research (5’-3’).

**shRNA target oligo**	shIGF2BP3#1	gcACATTTAATTCCTGGATTA
	shIGF2BP3#2	GCAGGAATTGACGCTGTAT
	si-RCC2#1	GGGACUUGAUUGGUCGAAATT
	si-RCC2#2	GGGCUGAAUUCAGUAUGAUTT
	si-RCC2#3	GGUGAUAGCAAGAGAUGAATT
**Primers used in PCR**	IGF2BP3 forward	GGGAGGTGCTGGATAGTTTAC
	IGF2BP3 reverse	CTAGCTTGGTCCTTACTGGAATAG
	RCC2 forward	ACACCTCCCGTGAATCTACC
	RCC2 reverse	ATGCCATCCAGAGTCTTTACCT
	GAPDH forward	CAGGAGGCATTGCTGATGAT
	GAPDH reverse	GAAGGCTGGGGCTCATTT
	ACTIN forward	CGTGGACATCCGCAAAG
	ACTIN reverse	AAGGTGGACAGCGAGGC

Supplementary Table 2. Clinical characteristics of Acute Myeloid Leukaemia patients in our cohort.

Patient_ID	Sample_ID	Age	Sex	Diagnosis	Category	Cytogenetic_Code	WBC /×109/L	Blast Percentage /%	Admission time	IGF2BP3 Relative Expression
Liu**	2019FG1077	54	Male	Acute Myeloid Leukaemia	AML-M2	NPM1, IDH2mut	8.6	85.16	12-Aug-19	1
Dong**	2019FG1877	78	Male	Acute Myeloid Leukaemia	AML-M2	AML1/ETO positive	62.4	74	20-Oct-19	0.2161193
Li**	2020FG0111	56	Female	Acute Myeloid Leukaemia	AML with myelodysplasia-related changes	TET2	21.47	56	03-Jan-20	0.8803533
Peng**	2020FG0112	65	Male	Acute Myeloid Leukaemia	AML-M1	na	299.5	90	06-Jan-20	0.1855181
Rao**	2020FG1452	34	Female	Acute Promyelocytic Leukemia	AML-M3	PML/RARA positive	0.34	73	18-Apr-20	0.1756742
Ding**	2020FG2336	64	Female	Acute Myeloid Leukaemia	AML with myelodysplasia-related	na	0.6	28	11-Jun-20	0.1335005
Tian**	2020FG3767	26	Female	Acute Myeloid Leukaemia	AML-M4	SF3B1 positive	3.91	22	03-Sep-20	0.9634103
Liao**	2020FG3882	61	Male	Acute Myeloid Leukaemia	AML-M5	CEBPA-double-mutated，IDH	85	88	10-Sep-20	0.320684
Xie*	2020FG5424	12	Female	Acute Myeloid Leukaemia	AML-M2	CEBPA-double-mutated	199.4	12	28-Nov-20	0.1263313
Qiu*	2020FG5697	16	Female	Iron deficiency anemia	na	na	8.13	na	14-Dec-20	0.0322063
Zhang*	2020FG5745	48	Female	Iron deficiency anemia	na	na	na	na	16-Dec-20	0.0578761
Tang**	2020FG5882	47	Female	Iron deficiency anemia	na	na	na	na	25-Dec-20	0.0439013
Qian**	2020FG5880	35	Female	Iron deficiency anemia	na	na	na	na	25-Dec-20	0.0341697
Wang**	2020FG5881	47	Male	Iron deficiency anemia	na	na	na	na	25-Dec-20	0.0921058

Supplementary Table 3. DESeq results from comparison of IGF2BP3-depleted and control cells

Gene_Symbol	Gene_ID	Trans_Type	BaseMean_shTreat	BaseMean_shNC	FoldChange	Log2FoldChange	Pval	Direction
MS4A6A	ENSG0000011007	retained_intron;pro	0.1383	4.9434	0.03	-5.16	1.21E-60	down
TIFAB	ENSG0000025583	protein_coding	0.0047	1.7223	0.00	-8.52	1.71E-54	down
CD180	ENSG0000013406	protein_coding;ret	0.0351	1.7733	0.02	-5.66	1.03E-47	down
MTUS1	ENSG0000012942	protein_coding;pro	0.0060	0.6473	0.01	-6.74	3.09E-47	down
IL31RA	ENSG0000016450	protein_coding;pro	0.0001	0.8280	0.00	-13.02	1.54E-44	down
ARL8B	ENSG0000013410	protein_coding;ret	55.2349	12.7999	4.32	2.11	8.15E-42	up
MYOF	ENSG0000013811	protein_coding;pro	0.0290	1.1479	0.03	-5.31	3.52E-40	down
FCER2	ENSG0000010492	protein_coding;ret	0.0948	6.6250	0.01	-6.13	6.04E-38	down
IRF8	ENSG0000014096	protein_coding;ret	0.1451	2.9010	0.05	-4.32	4.63E-37	down
BEX1	ENSG0000013316	protein_coding	8.0294	52.6597	0.15	-2.71	6.51E-37	down
ZNF445	ENSG0000018521	protein_coding;ret	0.3083	2.1067	0.15	-2.77	5.71E-36	down
LILRB4	ENSG0000018681	protein_coding;no	0.0103	1.1171	0.01	-6.77	6.80E-36	down
METTL7B	ENSG0000017043	protein_coding	0.2629	7.5841	0.03	-4.85	4.38E-35	down
GALM	ENSG0000014389	protein_coding;pro	0.0499	2.3990	0.02	-5.59	5.86E-34	down
XAF1	ENSG0000013253	protein_coding;ret	0.0193	0.8939	0.02	-5.53	5.35E-33	down
CD109	ENSG0000015653	protein_coding;pro	0.0669	1.9969	0.03	-4.90	5.95E-33	down
INHBA-AS1	ENSG0000022411	antisense	0.0001	0.6101	0.00	-12.57	2.41E-32	down
ADGRA2	ENSG0000002018	protein_coding	0.1440	2.9955	0.05	-4.38	2.83E-30	down
TRPM8	ENSG0000014448	protein_coding;pro	0.0001	0.2915	0.00	-11.51	2.17E-27	down
IVD	ENSG0000012892	protein_coding;ret	0.0978	1.2644	0.08	-3.69	3.36E-26	down
C1orf21	ENSG0000011666	protein_coding;pro	0.0109	0.4222	0.03	-5.28	3.91E-26	down
ADAM23	ENSG0000011494	protein_coding	0.0023	0.4014	0.01	-7.42	8.73E-26	down
NPHP3-ACA	ENSG0000027481	nonsense_mediate	0.0001	0.3042	0.00	-11.51	1.94E-25	down
AL365361	ENSG0000025983	lincRNA	0.0027	0.6325	0.00	-7.88	6.76E-25	down
IGFBP2	ENSG0000011545	protein_coding;ret	0.5579	0.0038	146.01	7.19	1.02E-24	up
CLC	ENSG0000010520	protein_coding	601.4046	200.1856	3.00	1.59	5.92E-24	up
FGL2	ENSG0000012795	protein_coding	1.2718	6.6004	0.19	-2.38	2.38E-23	down
ITGA7	ENSG0000013542	protein_coding;no	0.0469	0.7913	0.06	-4.08	2.40E-23	down
AFF3	ENSG0000014421	protein_coding;no	0.0025	0.2805	0.01	-6.83	2.95E-23	down
SASH1	ENSG0000011196	protein_coding;ret	0.0024	0.2708	0.01	-6.80	1.07E-22	down
ALDH5A1	ENSG0000011229	protein_coding;ret	3.1297	0.4924	6.36	2.67	3.62E-22	up
WDR49	ENSG0000017477	protein_coding;pro	0.0001	0.3276	0.00	-11.68	2.12E-20	down
PTPN3	ENSG0000007015	protein_coding;pro	0.6590	0.0453	14.56	3.86	3.67E-20	up
GPNMB	ENSG0000013623	protein_coding;ret	0.5483	3.3193	0.17	-2.60	3.77E-20	down
MT1G	ENSG0000012514	protein_coding	12.3818	1.6129	7.68	2.94	2.20E-19	up
EPX	ENSG0000012105	protein_coding	137.2264	46.7979	2.93	1.55	3.84E-19	up
PDPK1	ENSG0000014099	protein_coding;ret	0.6975	2.8548	0.24	-2.03	9.09E-19	down
MS4A7	ENSG0000016692	protein_coding;ret	0.0329	1.0826	0.03	-5.04	9.23E-19	down
RXFP1	ENSG0000017150	protein_coding;pro	0.0116	0.4050	0.03	-5.13	1.54E-18	down
CADM1	ENSG0000018298	protein_coding;ret	0.0252	0.4096	0.06	-4.02	1.58E-18	down
INHBA	ENSG0000012264	protein_coding;pro	0.0034	0.2746	0.01	-6.32	3.19E-18	down
CD52	ENSG0000016944	protein_coding;pro	63.4170	22.5819	2.81	1.49	8.44E-18	up
SPP1	ENSG0000011878	protein_coding;pro	5.8676	1.0433	5.62	2.49	1.04E-17	up
GNG10	ENSG0000024261	protein_coding	2.1559	14.2025	0.15	-2.72	1.14E-17	down

HGF	ENSG0000001999	protein_coding;ret	0.9287	3.6591	0.25	-1.98	1.75E-17	down
NAV3	ENSG0000006779	protein_coding;ret	1.2691	4.0974	0.31	-1.69	3.61E-17	down
PADI4	ENSG0000015933	protein_coding;pro	21.2445	7.7026	2.76	1.46	3.83E-17	up
DTNA	ENSG0000013476	protein_coding;pro	0.0522	0.6517	0.08	-3.64	4.26E-17	down
DTX4	ENSG0000011004	protein_coding;ret	1.4265	0.2165	6.59	2.72	4.56E-17	up
CERS6	ENSG0000017229	protein_coding	7.1225	17.3761	0.41	-1.29	5.95E-17	down
ADA2	ENSG0000009307	protein_coding;pro	0.1209	0.9528	0.13	-2.98	8.13E-17	down
S100A9	ENSG0000016322	protein_coding	2672.9503	1053.2792	2.54	1.34	8.87E-17	up
HDAC9	ENSG0000004805	protein_coding;ret	0.0178	0.3542	0.05	-4.31	1.81E-16	down
PRG2	ENSG0000018665	retained_intron;pro	4121.7804	1553.5761	2.65	1.41	2.37E-16	up
FYN	ENSG0000001081	protein_coding;ret	0.0324	0.5918	0.05	-4.19	3.66E-16	down
STEAP4	ENSG0000012795	protein_coding	1.3128	0.2526	5.20	2.38	5.42E-16	up
AL359762	ENSG0000028585	processed_transcri	2.5687	0.0960	26.77	4.74	6.69E-16	up
PHF10	ENSG0000013002	protein_coding;ret	7.1470	16.9495	0.42	-1.25	2.08E-15	down
NAPSB	ENSG0000013140	transcribed_unpro	0.2915	2.3088	0.13	-2.99	4.04E-15	down
HLA-DRA	ENSG0000020428	protein_coding	0.0621	1.9986	0.03	-5.01	1.10E-14	down
CR1	ENSG0000020371	protein_coding;ret	9.7457	3.6594	2.66	1.41	1.53E-14	up
EMP1	ENSG0000013453	protein_coding;pro	0.0482	0.4651	0.10	-3.27	2.09E-14	down
CPA3	ENSG0000016375	protein_coding;ret	3.2682	0.3794	8.61	3.11	2.62E-14	up
LOXHD1	ENSG0000016721	protein_coding;ret	0.0205	0.3048	0.07	-3.89	3.62E-14	down
LY86	ENSG0000011279	protein_coding	1.5430	7.6808	0.20	-2.32	3.74E-14	down
SEPT3	ENSG0000010016	protein_coding;ret	0.0860	0.6336	0.14	-2.88	6.48E-14	down
NCF1	ENSG0000015851	protein_coding;ret	22.3825	8.9779	2.49	1.32	7.60E-14	up
ADD2	ENSG0000007534	protein_coding;pro	0.0137	0.1903	0.07	-3.79	9.71E-14	down
POC1B-GAI	ENSG0000025907	protein_coding	1.1298	0.0036	313.45	8.29	1.22E-13	up
CDC42BPA	ENSG0000014377	protein_coding;pro	0.4128	0.0426	9.68	3.28	2.55E-13	up
PRLR	ENSG0000011349	protein_coding;no	0.0248	0.2615	0.09	-3.40	3.59E-13	down
S100A8	ENSG0000014354	protein_coding;pro	491.6053	225.3193	2.18	1.13	3.86E-13	up
ZDBF2	ENSG0000020418	protein_coding;no	0.0059	0.1581	0.04	-4.74	6.92E-13	down
WASF3	ENSG0000013297	protein_coding;pro	0.1830	0.0036	50.55	5.66	6.92E-13	up
CLSTN2	ENSG0000015825	protein_coding;ret	0.0029	0.1304	0.02	-5.48	7.25E-13	down
NCF1C	ENSG0000016517	unprocessed_pseu	33.3546	13.3115	2.51	1.33	1.16E-12	up
SMIM12	ENSG0000016386	protein_coding;no	2.7584	6.3501	0.43	-1.20	1.21E-12	down
YTHDF2	ENSG0000019849	protein_coding;pro	9.0895	22.8858	0.40	-1.33	1.28E-12	down
MGAM	ENSG0000025733	protein_coding;ret	9.5572	4.3963	2.17	1.12	1.50E-12	up
LSM5	ENSG0000010635	protein_coding;no	6.6853	14.8633	0.45	-1.15	3.19E-12	down
KCNH2	ENSG0000005511	protein_coding;ret	3.0862	0.9194	3.36	1.75	3.27E-12	up
SLC41A1	ENSG0000013306	protein_coding;pro	0.0062	0.2269	0.03	-5.20	4.23E-12	down
HLA-B	ENSG0000023474	retained_intron;pro	125.4714	270.5205	0.46	-1.11	4.94E-12	down
CLIP4	ENSG0000011529	protein_coding;ret	0.0074	0.1806	0.04	-4.60	5.84E-12	down
ABCB4	ENSG0000000547	protein_coding;ret	0.0001	0.0902	0.00	-9.82	8.77E-12	down
MSH6	ENSG0000011606	protein_coding;no	7.7162	15.7190	0.49	-1.03	8.77E-12	down
IREB2	ENSG0000013638	protein_coding;no	3.9083	8.1572	0.48	-1.06	9.86E-12	down
TNS1	ENSG0000007930	protein_coding;ret	0.0027	0.0688	0.04	-4.69	1.05E-11	down
RHEX	ENSG0000026396	protein_coding;ret	14.2290	4.5701	3.11	1.64	1.47E-11	up
TRGC1	ENSG0000021168	TR_C_gene	15.5792	32.4249	0.48	-1.06	1.62E-11	down
IL9R	ENSG0000012433	protein_coding;pro	2.4561	0.4876	5.04	2.33	1.69E-11	up
TGM5	ENSG0000010405	protein_coding;ret	5.0594	12.1859	0.42	-1.27	1.71E-11	down

HTR7	ENSG0000014868	protein_coding	0.0165	0.4355	0.04	-4.72	1.96E-11	down
OAT	ENSG0000006515	protein_coding;pro	1.7515	0.3626	4.83	2.27	2.05E-11	up
SEL1L3	ENSG0000009149	protein_coding;ret	1.8274	4.8746	0.37	-1.42	2.06E-11	down
HPGD	ENSG0000016412	protein_coding;ret	2.0883	0.5589	3.74	1.90	2.27E-11	up
MT2A	ENSG0000012514	protein_coding;ret	88.8864	39.3008	2.26	1.18	2.84E-11	up
ITGAX	ENSG0000014067	protein_coding;ret	5.8394	2.2802	2.56	1.36	3.24E-11	up
H2AFY2	ENSG0000009928	protein_coding	0.5632	0.0185	30.45	4.93	4.32E-11	up
MRTFB	ENSG0000018626	protein_coding;pro	0.3024	0.0555	5.44	2.44	4.76E-11	up
PRSS33	ENSG0000010335	protein_coding	0.4150	0.0106	39.04	5.29	4.91E-11	up
DLX1	ENSG0000014435	retained_intron;pro	0.9835	3.5139	0.28	-1.84	7.46E-11	down
RBM15	ENSG0000016277	protein_coding	0.9399	3.3398	0.28	-1.83	8.38E-11	down
AOC1	ENSG0000000272	protein_coding;pro	6.8944	2.0249	3.40	1.77	8.93E-11	up
RECK	ENSG0000012270	protein_coding;pro	0.1269	0.7323	0.17	-2.53	9.32E-11	down
PLCB4	ENSG0000010133	processed_transcri	0.0650	0.5026	0.13	-2.95	9.74E-11	down
STARD8	ENSG0000013005	protein_coding;pro	0.0098	0.3434	0.03	-5.12	1.07E-10	down
C11orf45	ENSG0000017437	protein_coding;pro	0.0231	0.4043	0.06	-4.13	2.06E-10	down
APOC1	ENSG0000013020	protein_coding;no	0.1170	1.1765	0.10	-3.33	2.25E-10	down
VNN2	ENSG0000011230	nonsense_mediate	18.1123	7.4215	2.44	1.29	2.41E-10	up
KYNU	ENSG0000011591	protein_coding;ret	0.0089	0.0814	0.11	-3.20	3.10E-10	down
GNAI2	ENSG0000011435	protein_coding;ret	24.0469	46.6122	0.52	-0.95	3.31E-10	down
TNS3	ENSG0000013620	protein_coding;pro	0.0107	0.1259	0.09	-3.55	3.62E-10	down
TIMP1	ENSG0000010226	protein_coding	48.1324	23.7837	2.02	1.02	4.52E-10	up
AC006011	ENSG0000024456	processed_pseudo	0.1690	23.5245	0.01	-7.12	5.30E-10	down
H1F0	ENSG0000018906	protein_coding	1.4268	5.1734	0.28	-1.86	5.55E-10	down
VPS4A	ENSG0000013261	protein_coding;pro	6.6146	12.9607	0.51	-0.97	5.99E-10	down
LINC01670	ENSG0000027909	lincRNA	0.9002	0.0001	9002.15	13.14	6.19E-10	up
ACOT2	ENSG0000011967	nonsense_mediate	0.0514	0.8639	0.06	-4.07	6.55E-10	down
EPAS1	ENSG0000011601	retained_intron;pro	0.5073	0.0955	5.31	2.41	6.60E-10	up
ANAPC13	ENSG0000012905	protein_coding;ret	4.7574	14.5220	0.33	-1.61	6.68E-10	down
NOB1	ENSG0000014110	protein_coding;no	29.8780	56.5100	0.53	-0.92	8.51E-10	down
TMEM121B	ENSG0000018330	protein_coding	0.0576	0.4710	0.12	-3.03	8.54E-10	down
FPR1	ENSG0000017105	protein_coding	30.9062	14.9025	2.07	1.05	8.57E-10	up
SLC16A1	ENSG0000015538	protein_coding;pro	11.6687	22.5796	0.52	-0.95	8.60E-10	down
NCF1B	ENSG0000018248	processed_transcri	21.8829	9.1454	2.39	1.26	1.05E-09	up
MIR155HG	ENSG0000023488	lincRNA	0.3595	1.9911	0.18	-2.47	1.17E-09	down
NOG	ENSG0000018369	protein_coding	0.1128	1.0327	0.11	-3.19	1.28E-09	down
DPYSL2	ENSG0000009296	protein_coding;ret	0.1228	0.5966	0.21	-2.28	1.43E-09	down
HDC	ENSG0000014028	protein_coding;ret	1.9853	0.4848	4.09	2.03	1.58E-09	up
DKK2	ENSG0000015501	protein_coding;ret	0.0365	0.3122	0.12	-3.10	1.62E-09	down
HERC5	ENSG0000013864	protein_coding;ret	4.1784	1.4449	2.89	1.53	1.91E-09	up
TNFSF15	ENSG0000018163	protein_coding	0.3585	0.0558	6.42	2.68	2.26E-09	up
NCOA1	ENSG0000008467	protein_coding;ret	1.8863	0.6086	3.10	1.63	2.44E-09	up
FCN1	ENSG0000008526	protein_coding	2.9873	1.1305	2.64	1.40	2.78E-09	up
CPNE8	ENSG0000013911	protein_coding;pro	0.0396	0.3693	0.11	-3.22	3.26E-09	down
LRRK2	ENSG0000018890	protein_coding;ret	1.7605	0.6666	2.64	1.40	3.57E-09	up
COCH	ENSG0000010047	protein_coding;pro	24.7352	11.9732	2.07	1.05	3.61E-09	up
RNF114	ENSG0000012422	protein_coding;ret	11.6927	24.3220	0.48	-1.06	3.68E-09	down
SMAD3	ENSG0000016694	protein_coding;ret	0.9474	2.4974	0.38	-1.40	3.84E-09	down

PDZD7	ENSG0000018686	protein_coding;no	0.4334	1.4393	0.30	-1.73	4.15E-09	down
ZNRD1	ENSG0000006637	protein_coding;ret	4.6683	10.7492	0.43	-1.20	4.28E-09	down
CCR2	ENSG0000012180	protein_coding;pro	1.1847	3.6322	0.33	-1.62	4.56E-09	down
NRP2	ENSG0000011825	protein_coding;pro	0.0085	0.0993	0.09	-3.55	5.96E-09	down
THSD7A	ENSG0000000510	protein_coding;pro	0.0867	0.0060	14.34	3.84	6.71E-09	up
CLEC2B	ENSG0000011085	protein_coding;ret	0.7702	2.9031	0.27	-1.91	7.30E-09	down
RNASE2	ENSG0000016938	protein_coding	281.7874	150.1965	1.88	0.91	7.44E-09	up
OGFRL1	ENSG0000011990	protein_coding;pro	7.2733	13.3491	0.54	-0.88	7.51E-09	down
AFF1	ENSG0000017249	protein_coding;ret	1.3094	3.6550	0.36	-1.48	7.60E-09	down
IKZF3	ENSG0000016140	protein_coding;no	0.3276	0.0616	5.32	2.41	7.90E-09	up
IFI30	ENSG0000021649	protein_coding;ret	111.8283	61.1478	1.83	0.87	8.46E-09	up
DFFB	ENSG0000016959	protein_coding;no	1.6556	4.5092	0.37	-1.45	9.66E-09	down
TATDN1	ENSG0000014768	protein_coding;no	3.6212	8.0119	0.45	-1.15	1.00E-08	down
LILRA5	ENSG0000018711	protein_coding;ret	7.9305	2.5516	3.11	1.64	1.07E-08	up
LY9	ENSG0000012222	protein_coding;pro	0.0577	0.3157	0.18	-2.45	1.08E-08	down
VWDE	ENSG0000014653	nonsense_mediate	0.3848	0.0529	7.27	2.86	1.32E-08	up
STC2	ENSG0000011373	protein_coding;pro	0.3239	1.1568	0.28	-1.84	1.37E-08	down
RGS1	ENSG0000009010	protein_coding;ret	0.4164	1.5415	0.27	-1.89	1.48E-08	down
MEF2D	ENSG0000011660	retained_intron;pro	3.6306	7.5913	0.48	-1.06	1.52E-08	down
PLEKHA1	ENSG0000010767	protein_coding;pro	0.4803	0.1574	3.05	1.61	2.07E-08	up
PDIA4	ENSG0000015566	protein_coding;ret	82.0390	42.7898	1.92	0.94	2.17E-08	up
RET	ENSG0000016573	protein_coding;ret	0.4398	1.4231	0.31	-1.69	2.21E-08	down
AC114786	ENSG0000024976	processed_pseudo;	0.5927	0.0625	9.48	3.24	2.39E-08	up
ATP1A1	ENSG0000016339	protein_coding;pro	23.2201	41.8795	0.55	-0.85	2.79E-08	down
THBS1	ENSG0000013780	protein_coding;ret	3.3072	1.4308	2.31	1.21	3.59E-08	up
ZNF320	ENSG0000018298	protein_coding;pro	0.0298	0.1806	0.17	-2.60	3.75E-08	down
ROR2	ENSG0000016907	protein_coding;pro	0.0837	0.5672	0.15	-2.76	3.77E-08	down
HNRNPA1P	ENSG0000022093	processed_pseudo;	0.0001	1.0907	0.00	-13.41	5.18E-08	down
CRIM1	ENSG0000015093	protein_coding;pro	1.7033	0.5857	2.91	1.54	5.71E-08	up
GAS5	ENSG0000023474	lincRNA;retained_	31.5385	55.8085	0.57	-0.82	5.74E-08	down
GATA2	ENSG0000017934	protein_coding;pro	7.8981	3.5589	2.22	1.15	5.87E-08	up
SYS1-DBNI	ENSG0000025480	nonsense_mediate	0.0001	1.7994	0.00	-14.14	5.89E-08	down
GPR174	ENSG0000014713	protein_coding	0.3811	0.0621	6.14	2.62	6.45E-08	up
SAMHD1	ENSG0000010134	retained_intron;no	1.7872	3.9511	0.45	-1.14	7.23E-08	down
C2	ENSG0000016627	protein_coding;no	0.0304	0.2896	0.10	-3.25	7.59E-08	down
FRMD4B	ENSG0000011454	protein_coding;pro	0.3113	0.0617	5.04	2.33	7.93E-08	up
SRGN	ENSG0000012286	processed_transcri	2605.4933	4518.8461	0.58	-0.79	8.76E-08	down
APOC4-APO	ENSG0000022491	nonsense_mediate	6.7868	0.9018	7.53	2.91	9.27E-08	up
ITGA2B	ENSG0000000596	protein_coding;ret	1.3691	0.3580	3.82	1.93	9.31E-08	up
TNF	ENSG0000023281	protein_coding	7.9897	19.6047	0.41	-1.29	9.67E-08	down
LINC00996	ENSG0000024225	lincRNA	0.2244	1.2318	0.18	-2.46	1.02E-07	down
GALNT14	ENSG0000015808	protein_coding;pro	1.0469	0.2870	3.65	1.87	1.07E-07	up
AC006001	ENSG0000027540	processed_pseudo;	0.2102	7.1323	0.03	-5.08	1.35E-07	down
PDIA6	ENSG0000014387	protein_coding;pro	143.3468	76.5909	1.87	0.90	1.39E-07	up
SNX27	ENSG0000014337	protein_coding;ret	0.5858	1.3997	0.42	-1.26	1.39E-07	down
AC023906	ENSG0000025971	antisense	0.3725	4.1770	0.09	-3.49	1.39E-07	down
IL1R1	ENSG0000011559	protein_coding;no	2.4604	0.9216	2.67	1.42	1.68E-07	up
AL356417	ENSG0000028549	lincRNA	0.0170	0.1074	0.16	-2.66	1.68E-07	down

RGS2	ENSG0000011674	protein_coding;pr	8.5789	3.3493	2.56	1.36	1.71E-07	up
DDIT4L	ENSG0000014535	protein_coding	6.7591	2.8379	2.38	1.25	1.75E-07	up
PFN2	ENSG0000007008	protein_coding;no	0.7493	0.1553	4.82	2.27	1.91E-07	up
ZNF501	ENSG0000018644	protein_coding;pr	0.0106	0.2223	0.05	-4.39	1.94E-07	down
PRDX2	ENSG0000016781	protein_coding;ret	9.3058	3.8574	2.41	1.27	1.98E-07	up
AC068533	ENSG0000024931	protein_coding	1.3763	0.0001	######	13.75	2.22E-07	up
DLD	ENSG0000009114	retained_intron;pr	14.8230	25.5839	0.58	-0.79	2.26E-07	down
CKB	ENSG0000016616	retained_intron;pr	3.0144	0.9787	3.08	1.62	2.51E-07	up
NDRG2	ENSG0000016579	protein_coding;ret	0.2113	0.7009	0.30	-1.73	2.69E-07	down
FKBP5	ENSG0000009606	protein_coding	13.0336	7.5787	1.72	0.78	2.70E-07	up
GALNT10	ENSG0000016457	protein_coding;no	1.2886	0.5053	2.55	1.35	2.81E-07	up
JAML	ENSG0000016059	nonsense_mediate	8.1448	4.3281	1.88	0.91	2.81E-07	up
TNFAIP6	ENSG0000012361	protein_coding;ret	5.3122	1.8734	2.84	1.50	3.05E-07	up
TMEM263	ENSG0000015113	protein_coding;pr	1.4000	0.3975	3.52	1.82	3.05E-07	up
CAPN2	ENSG0000016290	processed_transcri	0.8139	1.9643	0.41	-1.27	3.25E-07	down
NTRK1	ENSG0000019840	protein_coding;pr	0.5460	0.0857	6.37	2.67	3.46E-07	up
GAL	ENSG0000006948	protein_coding;pr	31.2365	14.4034	2.17	1.12	3.59E-07	up
UAP1L1	ENSG0000019735	protein_coding;ret	0.2815	0.9039	0.31	-1.68	3.88E-07	down
AL591806	ENSG0000027014	nonsense_mediate	1.3231	0.0274	48.37	5.60	3.91E-07	up
ITPR3	ENSG0000009643	protein_coding	0.1096	0.0093	11.77	3.56	4.39E-07	up
CDKN2B	ENSG0000014788	protein_coding;ret	1.5183	0.4216	3.60	1.85	4.87E-07	up
MLF1	ENSG0000017805	protein_coding;no	1.3922	0.4429	3.14	1.65	5.57E-07	up
FUCA1	ENSG0000017916	protein_coding	34.2490	19.6391	1.74	0.80	5.60E-07	up
STK33	ENSG0000013041	processed_transcri	1.7303	0.6600	2.62	1.39	5.70E-07	up
NLRP3	ENSG0000016271	protein_coding;no	2.4122	5.2341	0.46	-1.12	5.86E-07	down
CDRT4	ENSG0000023970	protein_coding;pr	0.0819	1.5321	0.05	-4.23	6.06E-07	down
RTN2	ENSG0000012574	protein_coding;no	0.1429	0.6570	0.22	-2.20	6.24E-07	down
TSPOAP1	ENSG0000000537	protein_coding;ret	0.9502	2.1561	0.44	-1.18	6.33E-07	down
GIMAP5	ENSG0000019632	protein_coding;ret	0.1216	0.4996	0.24	-2.04	6.43E-07	down
IL1R2	ENSG0000011559	protein_coding;pr	0.9671	0.2323	4.16	2.06	6.49E-07	up
TRIQK	ENSG0000020513	protein_coding;pr	4.3525	8.0072	0.54	-0.88	6.54E-07	down
CRAT	ENSG0000009532	protein_coding;pr	0.1099	0.4985	0.22	-2.18	6.73E-07	down
FERMT1	ENSG0000010131	protein_coding;pr	0.5514	0.1210	4.56	2.19	6.87E-07	up
OAF	ENSG0000018423	protein_coding;pr	0.7465	2.1410	0.35	-1.52	6.87E-07	down
CD99	ENSG0000000258	protein_coding;ret	8.0377	3.9921	2.01	1.01	6.92E-07	up
ALDH1L2	ENSG0000013601	retained_intron;pr	0.8922	2.2457	0.40	-1.33	7.77E-07	down
GSG1	ENSG0000011130	protein_coding	0.3513	1.2362	0.28	-1.82	7.95E-07	down
GRPEL2	ENSG0000016428	protein_coding;pr	1.9601	8.1751	0.24	-2.06	8.02E-07	down
IGFBP7	ENSG0000016345	protein_coding;pr	36.6357	16.4779	2.22	1.15	8.44E-07	up
HIST2H2BE	ENSG0000018467	protein_coding	3.3057	1.1251	2.94	1.55	8.46E-07	up
FAM50B	ENSG0000014594	protein_coding	3.3614	1.1742	2.86	1.52	1.03E-06	up
AC138392	ENSG0000021920	processed_pseudo	3.3312	9.1863	0.36	-1.46	1.05E-06	down
FCGR1A	ENSG0000015033	protein_coding;pr	13.8716	23.6317	0.59	-0.77	1.11E-06	down
PROSER1	ENSG0000012068	protein_coding;ret	4.6682	10.5099	0.44	-1.17	1.13E-06	down
SNHG1	ENSG0000025571	lincRNA;retained_	21.3909	37.7086	0.57	-0.82	1.15E-06	down
MSH5-SAP(	ENSG0000025515	nonsense_mediate	0.9625	2.6189	0.37	-1.44	1.18E-06	down
OR2A9P	ENSG0000022896	transcribed_unpro	0.3573	0.0661	5.41	2.43	1.36E-06	up
EIF3M	ENSG0000014910	protein_coding;ret	24.3394	40.4194	0.60	-0.73	1.40E-06	down

APOL6	ENSG0000022196	protein_coding	0.5532	1.3878	0.40	-1.33	1.41E-06	down
HHLA2	ENSG0000011445	protein_coding	0.4323	1.3568	0.32	-1.65	1.46E-06	down
DDIT4	ENSG0000016820	protein_coding;ret	20.1515	34.5156	0.58	-0.78	1.48E-06	down
GPAT3	ENSG0000013867	protein_coding;pr	1.3264	0.4200	3.16	1.66	1.53E-06	up
MARCKSL1	ENSG0000017513	protein_coding	20.0365	9.2845	2.16	1.11	1.58E-06	up
AL121594	ENSG0000025879	nonsense_mediate	4.8125	18.1261	0.27	-1.91	1.59E-06	down
HLA-A	ENSG0000020650	retained_intron;pr	20.3606	11.4043	1.79	0.84	1.66E-06	up
PTPRCAP	ENSG0000021340	protein_coding	57.4872	30.1055	1.91	0.93	1.67E-06	up
CYP4F3	ENSG0000018652	protein_coding;ret	7.2261	3.8420	1.88	0.91	1.70E-06	up
ARHGAP21	ENSG0000010786	protein_coding;ret	0.1978	0.0391	5.06	2.34	1.78E-06	up
PRTN3	ENSG0000019641	protein_coding	762.9220	397.8789	1.92	0.94	1.83E-06	up
HMGCS1	ENSG0000011297	protein_coding;ret	18.8306	10.6853	1.76	0.82	1.84E-06	up
PIK3R6	ENSG0000027623	nonsense_mediate	1.3758	0.4522	3.04	1.61	1.85E-06	up
OTULINL	ENSG0000014556	protein_coding;pr	1.9387	3.9909	0.49	-1.04	1.95E-06	down
LINC01578	ENSG0000027288	retained_intron;lin	2.6560	5.5301	0.48	-1.06	1.96E-06	down
ARHGAP5	ENSG0000010085	protein_coding;ret	0.0910	0.0165	5.52	2.46	2.03E-06	up
MGAT3	ENSG0000012826	protein_coding	1.2729	0.4579	2.78	1.47	2.04E-06	up
LUC7L2	ENSG0000014696	retained_intron;pr	20.4120	33.5225	0.61	-0.72	2.05E-06	down
ADGRE3	ENSG0000013135	protein_coding	4.0867	1.6708	2.45	1.29	2.12E-06	up
FZD6	ENSG0000016493	protein_coding;no	1.4985	0.4706	3.18	1.67	2.20E-06	up
CALR	ENSG0000017921	protein_coding;ret	861.9144	473.2142	1.82	0.87	2.22E-06	up
TRAC	ENSG0000027773	TR_C_gene	2.4020	6.6499	0.36	-1.47	2.35E-06	down
RSL24D1	ENSG0000013787	protein_coding;no	48.0254	80.4591	0.60	-0.74	2.36E-06	down
PPP1R16B	ENSG0000010144	protein_coding;pr	0.0510	0.2521	0.20	-2.30	2.54E-06	down
MT1X	ENSG0000018719	protein_coding;ret	10.4508	4.6607	2.24	1.16	2.74E-06	up
SIGLEC15	ENSG0000019704	retained_intron;pr	0.2720	0.0442	6.15	2.62	2.83E-06	up
CD1D	ENSG0000015847	protein_coding	0.3762	1.3980	0.27	-1.89	2.87E-06	down
OXER1	ENSG0000016288	protein_coding	0.7177	0.1330	5.40	2.43	2.88E-06	up
GJB2	ENSG0000016547	protein_coding	4.1650	1.6881	2.47	1.30	2.92E-06	up
GRIK5	ENSG0000010573	nonsense_mediate	0.3084	0.0634	4.86	2.28	2.92E-06	up
TMEM106C	ENSG0000013429	protein_coding;ret	33.9286	16.6154	2.04	1.03	3.10E-06	up
SERPINA1	ENSG0000019724	protein_coding;ret	7.1365	3.4187	2.09	1.06	3.10E-06	up
DYNC2H1	ENSG0000018724	protein_coding;no	0.5217	0.1702	3.06	1.62	3.18E-06	up
RPS6KA2	ENSG0000007124	protein_coding;pr	0.0267	0.1249	0.21	-2.23	3.30E-06	down
AC010547	ENSG0000026161	nonsense_mediate	0.0219	1.3779	0.02	-5.98	3.34E-06	down
MIPOL1	ENSG0000015133	protein_coding;pr	0.0422	0.0049	8.53	3.09	3.38E-06	up
ZFAS1	ENSG0000017741	antisense	35.7446	65.1151	0.55	-0.87	3.44E-06	down
ZFP92	ENSG0000018942	protein_coding	0.4925	1.2886	0.38	-1.39	3.48E-06	down
BPI	ENSG0000010142	protein_coding;no	227.2579	136.9834	1.66	0.73	3.64E-06	up
MMP25	ENSG0000000851	protein_coding;ret	2.5402	1.0417	2.44	1.29	3.68E-06	up
TCEAL4	ENSG0000013314	protein_coding;ret	1.0258	0.2344	4.38	2.13	3.69E-06	up
RALGPS2	ENSG0000011619	protein_coding;ret	0.4668	0.1464	3.19	1.67	3.80E-06	up
SLC7A2	ENSG0000000398	protein_coding	1.3626	5.0082	0.27	-1.88	3.95E-06	down
NRP1	ENSG0000009925	protein_coding	1.7000	0.7960	2.14	1.09	3.96E-06	up
SLC45A3	ENSG0000015871	protein_coding;pr	1.0639	0.3289	3.23	1.69	4.12E-06	up
ARSB	ENSG0000011327	protein_coding;pr	5.4241	3.0343	1.79	0.84	4.18E-06	up
NCF2	ENSG0000011670	protein_coding;pr	29.0468	17.3009	1.68	0.75	4.21E-06	up
FMNL3	ENSG0000016179	protein_coding;ret	0.3467	0.8288	0.42	-1.26	4.31E-06	down

NTSR1	ENSG0000010118	protein_coding;pro	0.2657	0.9138	0.29	-1.78	4.39E-06	down
TRAM1	ENSG0000006716	protein_coding;pro	28.1950	45.7077	0.62	-0.70	4.46E-06	down
NFE2	ENSG0000012340	protein_coding	18.9219	9.0485	2.09	1.06	4.84E-06	up
COLEC12	ENSG0000015827	protein_coding;pro	0.4256	0.1241	3.43	1.78	4.96E-06	up
ATF1	ENSG0000012326	protein_coding;no	10.7535	18.4457	0.58	-0.78	5.01E-06	down
IL1RL1	ENSG0000011560	protein_coding;no	0.9383	0.3170	2.96	1.57	5.04E-06	up
AZU1	ENSG0000017223	protein_coding	160.9422	98.4149	1.64	0.71	5.32E-06	up
DYSF	ENSG0000013563	protein_coding;pro	3.2508	1.6267	2.00	1.00	5.44E-06	up
AP1S3	ENSG0000015205	protein_coding;no	0.1025	0.3914	0.26	-1.93	5.49E-06	down
HMMR	ENSG0000007257	protein_coding;ret	7.9633	14.0559	0.57	-0.82	5.54E-06	down
LRPAP1	ENSG0000016395	nonsense_mediate	20.7956	12.7713	1.63	0.70	5.74E-06	up
CREG1	ENSG0000014316	protein_coding	44.4366	71.0800	0.63	-0.68	5.85E-06	down
SF3B3	ENSG0000018909	protein_coding;ret	10.9901	17.7517	0.62	-0.69	6.05E-06	down
CDC14B	ENSG0000008137	protein_coding;no	0.0107	0.1226	0.09	-3.51	6.21E-06	down
TMEM170A	ENSG0000016682	protein_coding;no	2.8088	5.6438	0.50	-1.01	6.45E-06	down
TPSB2	ENSG0000019725	protein_coding;no	1.1879	0.1107	10.73	3.42	6.57E-06	up
AL118508	ENSG0000027916	TEC	0.1837	0.7644	0.24	-2.06	6.72E-06	down
GATA1	ENSG0000010214	protein_coding	3.6224	1.2204	2.97	1.57	6.84E-06	up
RAB11A	ENSG0000010376	protein_coding;pro	7.5647	12.3036	0.61	-0.70	6.99E-06	down
PRICKLE1	ENSG0000013917	retained_intron;pro	0.2580	0.6967	0.37	-1.43	7.26E-06	down
ARHGEF12	ENSG0000019691	protein_coding;ret	0.0324	0.1155	0.28	-1.83	7.40E-06	down
RFX3	ENSG0000008029	protein_coding	0.4471	1.0859	0.41	-1.28	7.40E-06	down
ZNF24	ENSG0000017246	protein_coding	4.1714	17.1828	0.24	-2.04	7.45E-06	down
QPRT	ENSG0000010348	protein_coding;ret	12.4391	7.1872	1.73	0.79	7.61E-06	up
RPS10-NUD	ENSG0000027080	protein_coding	0.1939	4.8506	0.04	-4.64	7.65E-06	down
FN1	ENSG0000011541	protein_coding;ret	0.2861	0.1020	2.80	1.49	8.43E-06	up
EHBP1L1	ENSG0000017344	protein_coding;ret	5.8692	3.2684	1.80	0.84	8.68E-06	up
FAM72C	ENSG0000026351	protein_coding	6.5364	13.3603	0.49	-1.03	8.77E-06	down
ICAM1	ENSG0000009033	protein_coding;pro	2.3696	0.8696	2.72	1.45	9.19E-06	up
ANKRD49	ENSG0000016887	protein_coding;pro	3.9838	10.3209	0.39	-1.37	9.90E-06	down
MMP9	ENSG0000010098	protein_coding	1.6535	0.5673	2.91	1.54	1.00E-05	up
MT1E	ENSG0000016971	protein_coding	7.5729	3.0136	2.51	1.33	1.03E-05	up
TBC1D23	ENSG0000003605	protein_coding;pro	5.4883	9.2528	0.59	-0.75	1.06E-05	down
ENDOD1	ENSG0000014921	protein_coding	0.6623	1.8003	0.37	-1.44	1.06E-05	down
PCYOX1	ENSG0000011600	protein_coding;ret	5.2295	2.7016	1.94	0.95	1.09E-05	up
EHHADH	ENSG0000011379	protein_coding;pro	0.4861	0.1269	3.83	1.94	1.12E-05	up
ZNF727	ENSG0000021465	protein_coding	0.1915	0.0210	9.11	3.19	1.12E-05	up
DENND4A	ENSG0000017448	protein_coding;ret	2.7454	4.6824	0.59	-0.77	1.17E-05	down
MPEG1	ENSG0000019762	protein_coding	6.0254	3.0871	1.95	0.96	1.25E-05	up
AC019205	ENSG0000022985	antisense	0.3751	0.0888	4.22	2.08	1.27E-05	up
AGPAT1	ENSG0000020431	protein_coding;pro	11.4085	6.3678	1.79	0.84	1.29E-05	up
ECI2	ENSG0000019872	retained_intron;pro	0.4766	1.2165	0.39	-1.35	1.32E-05	down
POLR2A	ENSG0000018122	protein_coding;pro	5.3817	3.0259	1.78	0.83	1.35E-05	up
AP002990	ENSG0000025550	nonsense_mediate	297.0574	6.8659	43.27	5.44	1.37E-05	up
PYCR1	ENSG0000018301	protein_coding	11.4788	19.1665	0.60	-0.74	1.37E-05	down
TAF1D	ENSG0000016601	nonsense_mediate	8.2558	12.9373	0.64	-0.65	1.77E-05	down
COL15A1	ENSG0000020429	protein_coding;ret	0.0558	0.2326	0.24	-2.06	1.87E-05	down
RNMT	ENSG0000010165	protein_coding;no	4.3176	12.3647	0.35	-1.52	1.93E-05	down

PPIL3	ENSG0000024034	protein_coding;ret	5.6867	10.5200	0.54	-0.89	2.06E-05	down
LANCL3	ENSG0000014703	protein_coding	0.3127	0.1023	3.06	1.61	2.12E-05	up
CD63	ENSG0000013540	protein_coding;ret	130.8291	81.6078	1.60	0.68	2.13E-05	up
ZFP62	ENSG0000019667	protein_coding;pro	3.4130	1.4385	2.37	1.25	2.13E-05	up
CRISPLD2	ENSG0000010319	protein_coding;ret	0.7796	0.2501	3.12	1.64	2.16E-05	up
ADAM8	ENSG0000015165	retained_intron;pro	3.5558	1.7317	2.05	1.04	2.29E-05	up
DNAJC25-C	ENSG0000024411	protein_coding	1.1569	5.3800	0.22	-2.22	2.30E-05	down
SNHG5	ENSG0000020387	lincRNA;retained_	8.3660	14.1987	0.59	-0.76	2.33E-05	down
BORA	ENSG0000013612	protein_coding;no	6.5539	11.9192	0.55	-0.86	2.41E-05	down
CGAS	ENSG0000016443	protein_coding;ret	3.9348	7.1514	0.55	-0.86	2.47E-05	down
NOMO2	ENSG0000018516	protein_coding;no	25.2645	16.0397	1.58	0.66	2.51E-05	up
ATF5	ENSG0000016913	protein_coding	44.2602	72.9618	0.61	-0.72	2.54E-05	down
GNLY	ENSG0000011552	protein_coding;ret	0.6561	0.1762	3.72	1.90	2.69E-05	up
DDX21	ENSG0000016573	protein_coding	55.0784	85.8132	0.64	-0.64	2.79E-05	down
EHD1	ENSG0000011004	retained_intron;pro	2.4657	4.5623	0.54	-0.89	2.81E-05	down
PHACTR3	ENSG0000008749	protein_coding;ret	1.2418	2.5129	0.49	-1.02	2.85E-05	down
ETS1	ENSG0000013495	protein_coding;ret	0.2232	0.5962	0.37	-1.42	2.87E-05	down
SMG1P7	ENSG0000026155	processed_transcri	0.8766	2.0900	0.42	-1.25	2.93E-05	down
TUBE1	ENSG0000007493	nonsense_mediate	3.0732	5.3782	0.57	-0.81	2.93E-05	down
CKAP4	ENSG0000013602	protein_coding;pro	2.8817	1.3021	2.21	1.15	3.10E-05	up
PRAF2	ENSG0000024327	protein_coding;pro	4.0823	1.4714	2.77	1.47	3.13E-05	up
AC087190	ENSG0000026324	3prime_overlappir	0.5113	3.0261	0.17	-2.57	3.17E-05	down
RIPOR2	ENSG0000011191	protein_coding;pro	0.4481	0.1648	2.72	1.44	3.38E-05	up
C18orf32	ENSG0000017757	protein_coding	6.8909	2.6108	2.64	1.40	3.49E-05	up
TDRKH	ENSG0000018213	protein_coding;no	0.5561	0.1735	3.21	1.68	3.50E-05	up
BABAM2	ENSG0000015801	protein_coding;pro	9.5936	16.2785	0.59	-0.76	3.61E-05	down
RPN2	ENSG0000011870	protein_coding;pro	221.8932	142.7608	1.55	0.64	3.76E-05	up
MTLN	ENSG0000017570	protein_coding	2.3293	5.2459	0.44	-1.17	3.85E-05	down
AC023024	ENSG0000025917	antisense	0.0066	0.2459	0.03	-5.23	3.89E-05	down
S100A12	ENSG0000016322	protein_coding	50.8410	25.4337	2.00	1.00	3.99E-05	up
P2RX7	ENSG0000008904	protein_coding;no	0.3300	0.9592	0.34	-1.54	4.08E-05	down
P2RY6	ENSG0000017163	protein_coding	1.2519	0.4873	2.57	1.36	4.22E-05	up
VILL	ENSG0000013605	protein_coding;ret	1.2395	2.4390	0.51	-0.98	4.25E-05	down
AC159540	ENSG0000027770	lincRNA	3.5389	1.4662	2.41	1.27	4.30E-05	up
CHAC1	ENSG0000012896	protein_coding	2.2608	5.1391	0.44	-1.18	4.36E-05	down
ZNF354C	ENSG0000017793	protein_coding	0.7935	0.2810	2.82	1.50	4.37E-05	up
ATP5MD	ENSG0000017391	protein_coding	62.0833	95.9012	0.65	-0.63	4.40E-05	down
PLEKHO1	ENSG0000002390	protein_coding;ret	4.5244	2.4656	1.84	0.88	4.55E-05	up
TMEM9	ENSG0000011685	protein_coding;pro	0.3328	0.9357	0.36	-1.49	4.87E-05	down
TDRD7	ENSG0000019611	protein_coding;pro	11.5865	17.8371	0.65	-0.62	5.02E-05	down
SRP14	ENSG0000014031	protein_coding;pro	27.0449	41.7582	0.65	-0.63	5.07E-05	down
ADAM22	ENSG0000000827	protein_coding;pro	0.2332	0.0770	3.03	1.60	5.20E-05	up
SDHA	ENSG0000007357	retained_intron;pro	11.2200	7.2873	1.54	0.62	5.42E-05	up
CCNA1	ENSG0000013310	protein_coding	5.8362	10.8473	0.54	-0.89	5.51E-05	down
DDX60	ENSG0000013762	protein_coding;pro	0.0414	0.1709	0.24	-2.05	5.57E-05	down
LINC00900	ENSG0000024610	lincRNA	0.0421	0.2205	0.19	-2.39	5.70E-05	down
WNT10B	ENSG0000016988	protein_coding;ret	0.1284	0.4583	0.28	-1.84	5.71E-05	down
PAFAH1B2	ENSG0000016809	protein_coding;pro	6.6603	10.3068	0.65	-0.63	5.75E-05	down

ITM2C	ENSG0000013591	protein_coding;pr	35.1328	22.1861	1.58	0.66	5.78E-05	up
PLXNC1	ENSG0000013604	protein_coding;ret	3.2650	1.8518	1.76	0.82	5.85E-05	up
AC069288	ENSG0000028619	nonsense_mediate	0.1128	2.3522	0.05	-4.38	5.98E-05	down
SIGLEC6	ENSG0000010549	protein_coding;ret	3.1726	6.0444	0.52	-0.93	6.13E-05	down
DENND6B	ENSG0000020559	protein_coding;ret	0.1733	0.5128	0.34	-1.56	6.24E-05	down
NIP7	ENSG0000013260	retained_intron;pr	11.3231	17.2407	0.66	-0.61	6.26E-05	down
AL590764	ENSG0000028517	nonsense_mediate	0.5650	0.0582	9.70	3.28	6.31E-05	up
GOLGA8R	ENSG0000018639	protein_coding;TE	0.3835	1.0571	0.36	-1.46	6.37E-05	down
ASF1A	ENSG0000011187	protein_coding;ret	20.4986	32.0578	0.64	-0.65	6.46E-05	down
HEXIM1	ENSG0000018683	protein_coding	13.6716	8.4321	1.62	0.70	6.54E-05	up
MYO1E	ENSG0000015748	protein_coding;pr	0.0557	0.2041	0.27	-1.87	6.55E-05	down
SMOX	ENSG0000008882	protein_coding;pr	1.8255	3.9028	0.47	-1.10	6.63E-05	down
ZFR	ENSG0000005609	nonsense_mediate	17.1396	26.2921	0.65	-0.62	6.72E-05	down
FBXO10	ENSG0000014791	protein_coding;no	0.4914	1.1228	0.44	-1.19	6.76E-05	down
ITGB7	ENSG0000013962	protein_coding;ret	3.2419	1.5931	2.03	1.03	6.84E-05	up
ADM2	ENSG0000012816	protein_coding	0.0539	0.2243	0.24	-2.06	7.14E-05	down
CX3CR1	ENSG0000016832	protein_coding	0.0527	0.2320	0.23	-2.14	7.46E-05	down
AL365181	ENSG0000027240	antisense	0.9510	2.2705	0.42	-1.26	7.59E-05	down
BCL6	ENSG0000011391	protein_coding;no	4.1986	2.2720	1.85	0.89	7.61E-05	up
SEPT7-AS1	ENSG0000022887	lincRNA	0.1615	0.5545	0.29	-1.78	7.69E-05	down
ACSF2	ENSG0000016710	processed_transcri	0.0725	0.2442	0.30	-1.75	7.69E-05	down
ZC3H12C	ENSG0000014928	protein_coding	0.0410	0.1803	0.23	-2.14	7.71E-05	down
COMT	ENSG0000009301	nonsense_mediate	9.5362	15.8965	0.60	-0.74	7.72E-05	down
CHI3L1	ENSG0000013304	protein_coding;ret	197.7928	128.3398	1.54	0.62	7.98E-05	up
SRPX	ENSG0000010195	protein_coding;pr	4.9108	2.2334	2.20	1.14	7.99E-05	up
FAM72D	ENSG0000021578	protein_coding	6.7268	12.3137	0.55	-0.87	8.05E-05	down
ZPR1	ENSG0000010991	protein_coding;no	6.9960	10.8369	0.65	-0.63	8.09E-05	down
HK1	ENSG0000015651	protein_coding;pr	5.7568	3.3569	1.71	0.78	8.10E-05	up
POLR2L	ENSG0000017770	protein_coding;no	50.6239	31.0454	1.63	0.71	8.25E-05	up
GANC	ENSG0000021401	retained_intron;no	2.5726	4.1052	0.63	-0.67	8.26E-05	down
RTCA	ENSG0000013799	protein_coding;pr	12.2514	19.3011	0.63	-0.66	8.62E-05	down
ADAM1A	ENSG0000022918	unitary_pseudoger	2.3073	0.2800	8.24	3.04	8.81E-05	up
CCL3	ENSG0000027763	protein_coding;ret	0.2706	1.1769	0.23	-2.12	8.87E-05	down
ZC2HC1A	ENSG0000010442	protein_coding;pr	0.9730	0.3634	2.68	1.42	9.03E-05	up
DOCK10	ENSG0000013590	protein_coding;no	2.2114	3.6391	0.61	-0.72	9.06E-05	down
DESI1	ENSG0000010041	processed_transcri	11.5104	17.4951	0.66	-0.60	9.49E-05	down
CCNE2	ENSG0000017530	protein_coding;ret	6.5090	3.6273	1.79	0.84	1.02E-04	up
GPR18	ENSG0000012524	protein_coding	0.9670	2.4607	0.39	-1.35	1.03E-04	down
CEBPE	ENSG0000009206	protein_coding	164.0261	107.7185	1.52	0.61	1.03E-04	up
GANAB	ENSG0000008959	protein_coding;pr	76.2695	50.2409	1.52	0.60	1.05E-04	up
PDIA5	ENSG0000006548	protein_coding;pr	13.7907	8.5789	1.61	0.68	1.06E-04	up
UBE2Q2	ENSG0000014036	protein_coding;no	7.2002	11.3750	0.63	-0.66	1.07E-04	down
LINC01341	ENSG0000022795	lincRNA;retained_	0.6161	0.1900	3.24	1.70	1.09E-04	up
TNNI2	ENSG0000013059	retained_intron;pr	6.6714	2.8451	2.34	1.23	1.11E-04	up
CCDC59	ENSG0000013377	protein_coding;pr	10.9180	17.1639	0.64	-0.65	1.13E-04	down
GIMAP2	ENSG0000010656	protein_coding;ret	9.7436	17.2741	0.56	-0.83	1.14E-04	down
BMPR1A	ENSG0000010777	protein_coding;no	0.1884	0.4649	0.41	-1.30	1.18E-04	down
ARHGEF3	ENSG0000016394	protein_coding;pr	0.0502	0.1809	0.28	-1.85	1.22E-04	down

CDA	ENSG0000015882	protein_coding;pr	38.9636	21.8770	1.78	0.83	1.28E-04	up
COQ2	ENSG0000017308	protein_coding;no	6.6337	11.7607	0.56	-0.83	1.30E-04	down
AKR1C3	ENSG0000019613	protein_coding;ret	1.0163	0.3917	2.59	1.38	1.31E-04	up
GXYLT1	ENSG0000015123	protein_coding	4.8488	2.8685	1.69	0.76	1.38E-04	up
REXO5	ENSG0000000518	protein_coding;no	5.6007	9.2592	0.60	-0.73	1.39E-04	down
IGF2BP3	ENSG0000013623	protein_coding;pr	4.6799	7.2370	0.65	-0.63	1.41E-04	down
ZC3H15	ENSG0000006554	protein_coding;ret	30.8048	47.4045	0.65	-0.62	1.41E-04	down
RFLNB	ENSG0000018368	protein_coding	23.2949	15.3087	1.52	0.61	1.44E-04	up
RNF13	ENSG0000008299	protein_coding;no	7.0592	3.7484	1.88	0.91	1.44E-04	up
EFHC1	ENSG0000009609	retained_intron;pr	0.3546	0.6674	0.53	-0.91	1.45E-04	down
PIGT	ENSG0000012415	protein_coding;no	5.3370	3.3710	1.58	0.66	1.46E-04	up
ITPRIPL2	ENSG0000020573	protein_coding;pr	0.0501	0.2834	0.18	-2.50	1.48E-04	down
PRKCE	ENSG0000017113	protein_coding;pr	0.0497	0.1659	0.30	-1.74	1.50E-04	down
C15orf41	ENSG0000018607	protein_coding;pr	0.5242	1.2329	0.43	-1.23	1.52E-04	down
OLR1	ENSG0000017339	protein_coding;ret	0.4831	1.2629	0.38	-1.39	1.54E-04	down
CNTNAP3	ENSG0000010671	protein_coding;no	0.2287	0.0682	3.35	1.75	1.57E-04	up
CFP	ENSG0000012675	protein_coding;ret	4.5838	2.5730	1.78	0.83	1.68E-04	up
SLC9A6	ENSG0000019868	protein_coding;ret	3.5186	1.7293	2.03	1.02	1.73E-04	up
GFI1B	ENSG0000016570	protein_coding;pr	0.1702	0.0426	4.00	2.00	1.78E-04	up
FGD6	ENSG0000018026	protein_coding;no	0.1402	0.0437	3.21	1.68	1.79E-04	up
MEIOB	ENSG0000016203	protein_coding;no	0.5577	0.1559	3.58	1.84	1.80E-04	up
NOA1	ENSG0000008409	protein_coding	13.0630	20.7021	0.63	-0.66	1.83E-04	down
EIF2A	ENSG0000014489	protein_coding;ret	16.0757	25.2046	0.64	-0.65	1.84E-04	down
SPOPL	ENSG0000014422	protein_coding;no	2.7549	4.9609	0.56	-0.85	1.84E-04	down
ADGRB1	ENSG0000018179	protein_coding;ret	0.2813	0.0709	3.97	1.99	1.85E-04	up
ZCCHC24	ENSG0000016542	protein_coding	0.1831	0.5041	0.36	-1.46	1.86E-04	down
KIF1B	ENSG0000005452	protein_coding;ret	0.7750	0.3899	1.99	0.99	1.86E-04	up
SPTAN1	ENSG0000019769	processed_transcri	4.6884	3.0731	1.53	0.61	1.90E-04	up
SEMA7A	ENSG0000013862	protein_coding;ret	0.8346	0.3133	2.66	1.41	1.91E-04	up
FST	ENSG0000013436	protein_coding;ret	0.3612	0.0552	6.54	2.71	1.93E-04	up
SETDB2	ENSG0000013616	protein_coding;pr	8.6040	12.9192	0.67	-0.59	1.93E-04	down
C6orf120	ENSG0000018512	protein_coding	10.4628	16.4869	0.63	-0.66	1.94E-04	down
CD70	ENSG0000012572	protein_coding	0.1074	0.8578	0.13	-3.00	1.98E-04	down
RABGAP1L	ENSG0000015206	protein_coding;no	1.4787	2.4832	0.60	-0.75	1.99E-04	down
TP53INP1	ENSG0000016493	protein_coding	0.8122	0.3121	2.60	1.38	2.05E-04	up
DEFA3	ENSG0000023983	protein_coding	1676.8785	2567.6765	0.65	-0.61	2.05E-04	down
PSMG2	ENSG0000012878	protein_coding;ret	31.0620	45.6757	0.68	-0.56	2.08E-04	down
CTSZ	ENSG0000010116	protein_coding;ret	63.5683	42.6474	1.49	0.58	2.09E-04	up
RPL9P9	ENSG0000023755	processed_transcri	1046.2953	1557.7401	0.67	-0.57	2.13E-04	down
F5	ENSG0000019873	protein_coding;ret	1.2617	0.5980	2.11	1.08	2.19E-04	up
C10orf105	ENSG0000021468	protein_coding	0.2332	0.0137	17.07	4.09	2.25E-04	up
RNF157	ENSG0000014157	protein_coding;ret	1.1148	0.5106	2.18	1.13	2.27E-04	up
APMAP	ENSG0000010147	protein_coding	117.2654	79.7883	1.47	0.56	2.37E-04	up
CR2	ENSG0000011732	protein_coding;pr	0.1896	0.0264	7.18	2.84	2.38E-04	up
FCGR1CP	ENSG0000026553	unprocessed_pseu	11.9028	20.9800	0.57	-0.82	2.39E-04	down
ACADSB	ENSG0000019617	protein_coding;pr	4.3551	2.4527	1.78	0.83	2.44E-04	up
DHRS3	ENSG0000016249	protein_coding;ret	0.2074	0.6331	0.33	-1.61	2.45E-04	down
KCNAB2	ENSG0000006942	protein_coding;ret	19.3226	10.4221	1.85	0.89	2.46E-04	up

FADS1	ENSG0000014948	protein_coding;pro	11.5325	7.6028	1.52	0.60	2.47E-04	up
ADAT2	ENSG0000018900	protein_coding;pro	5.3927	9.6892	0.56	-0.85	2.55E-04	down
TCN1	ENSG0000013482	protein_coding;ret	3.4550	1.0904	3.17	1.66	2.57E-04	up
ZNF277	ENSG0000019883	nonsense_mediate	6.2177	10.5915	0.59	-0.77	2.58E-04	down
GOLGA8O	ENSG0000020612	protein_coding	2.7202	5.5382	0.49	-1.03	2.59E-04	down
NLRP12	ENSG0000014240	protein_coding;ret	0.5993	0.2342	2.56	1.36	2.61E-04	up
HIGD2A	ENSG0000014606	protein_coding	58.2277	88.1143	0.66	-0.60	2.63E-04	down
SH3BGRL3	ENSG0000014266	protein_coding	189.4811	127.7871	1.48	0.57	2.68E-04	up
STOX2	ENSG0000017332	protein_coding;no	0.3253	0.9005	0.36	-1.47	2.68E-04	down
CXorf21	ENSG0000012028	protein_coding	8.8543	14.9857	0.59	-0.76	2.72E-04	down
ABHD14A	ENSG0000024848	protein_coding;ret	6.4049	3.3983	1.88	0.91	2.79E-04	up
ETFB	ENSG0000010537	protein_coding;ret	13.1503	8.8313	1.49	0.57	2.81E-04	up
MYEF2	ENSG0000010417	protein_coding;ret	0.2061	0.0624	3.30	1.72	2.82E-04	up
S100A10	ENSG0000019774	protein_coding;pro	4.2141	8.7888	0.48	-1.06	2.86E-04	down
CD300A	ENSG0000016785	protein_coding;ret	12.8301	7.7241	1.66	0.73	2.88E-04	up
APOLD1	ENSG0000017887	nonsense_mediate	0.3231	1.7579	0.18	-2.44	2.91E-04	down
GBP3	ENSG0000011722	protein_coding;ret	0.3958	0.9535	0.42	-1.27	2.92E-04	down
EEF1G	ENSG0000025477	protein_coding;ret	694.9528	1543.5836	0.45	-1.15	2.98E-04	down
TPSAB1	ENSG0000017223	protein_coding;ret	0.5326	0.0564	9.44	3.24	2.99E-04	up
UBA7	ENSG0000018217	retained_intron;pro	1.5932	3.0302	0.53	-0.93	3.03E-04	down
AC104452	ENSG0000028318	nonsense_mediate	1.1510	0.4979	2.31	1.21	3.06E-04	up
C6orf48	ENSG0000020438	lincRNA;protein_c	92.2129	136.9801	0.67	-0.57	3.09E-04	down
RBP7	ENSG0000016244	protein_coding;no	1.2526	0.1998	6.27	2.65	3.17E-04	up
DNAJA4	ENSG0000014040	protein_coding;ret	1.5914	0.7787	2.04	1.03	3.17E-04	up
ALOX5	ENSG0000001277	protein_coding;pro	13.3644	8.5946	1.55	0.64	3.18E-04	up
ASS1	ENSG0000013070	protein_coding;ret	2.3748	4.7630	0.50	-1.00	3.29E-04	down
GNG2	ENSG0000018646	protein_coding;pro	4.1148	2.3867	1.72	0.79	3.30E-04	up
PINK1-AS	ENSG0000011724	antisense	5.9776	3.4702	1.72	0.78	3.41E-04	up
COX5A	ENSG0000017874	protein_coding	52.9833	76.9931	0.69	-0.54	3.43E-04	down
CLIC4	ENSG0000016950	protein_coding;pro	12.6152	18.8682	0.67	-0.58	3.47E-04	down
PPIB	ENSG0000016679	protein_coding;pro	149.4567	95.8930	1.56	0.64	3.47E-04	up
SNRPN	ENSG0000012873	protein_coding;pro	18.5909	11.6380	1.60	0.68	3.48E-04	up
SNHG15	ENSG0000023295	lincRNA	17.1046	26.9854	0.63	-0.66	3.49E-04	down
MSRB1	ENSG0000019873	protein_coding;pro	37.9372	25.4791	1.49	0.57	3.51E-04	up
ATRN	ENSG0000008881	protein_coding	11.2690	7.6764	1.47	0.55	3.53E-04	up
PLEC	ENSG0000017820	protein_coding;ret	1.5303	0.8627	1.77	0.83	3.54E-04	up
CCDC18	ENSG0000012248	protein_coding;ret	3.2333	5.4304	0.60	-0.75	3.60E-04	down
TNFSF13B	ENSG0000010252	protein_coding;pro	5.2155	9.5552	0.55	-0.87	3.64E-04	down
CASC11	ENSG0000024937	lincRNA	0.2063	0.6222	0.33	-1.59	3.72E-04	down
S100P	ENSG0000016399	protein_coding;pro	68.4830	108.6239	0.63	-0.67	3.73E-04	down
FCRL2	ENSG0000013270	retained_intron;pro	0.3455	0.1089	3.17	1.67	3.74E-04	up
EAF2	ENSG0000014508	nonsense_mediate	24.1901	35.5218	0.68	-0.55	3.89E-04	down
LCMT1	ENSG0000020562	nonsense_mediate	7.3643	12.0723	0.61	-0.71	3.91E-04	down
PGAM4	ENSG0000022678	protein_coding	15.2348	5.8593	2.60	1.38	3.91E-04	up
AL358473	ENSG0000022919	antisense	0.6015	0.1972	3.05	1.61	3.94E-04	up
ARHGEF35	ENSG0000021321	protein_coding	0.3916	0.0630	6.22	2.64	3.96E-04	up
FCRL1	ENSG0000016353	protein_coding;pro	1.8503	0.8848	2.09	1.06	3.97E-04	up
ACSS2	ENSG0000013106	protein_coding;ret	8.2983	5.4026	1.54	0.62	4.00E-04	up

NCS1	ENSG0000010713	protein_coding;prc	0.2808	0.0666	4.22	2.08	4.00E-04	up
ARHGAP15	ENSG0000007588	retained_intron;pr	11.2809	16.5437	0.68	-0.55	4.03E-04	down
TRIM71	ENSG0000020655	protein_coding	0.2020	0.0439	4.61	2.20	4.06E-04	up
NEB	ENSG0000018309	protein_coding;prc	0.0182	0.0615	0.30	-1.76	4.07E-04	down
MEGF9	ENSG0000010678	protein_coding	9.1748	6.1280	1.50	0.58	4.15E-04	up
ACAD8	ENSG0000015149	retained_intron;pr	4.7792	2.8885	1.65	0.73	4.16E-04	up
FAM45A	ENSG0000011997	protein_coding;prc	3.4630	6.6023	0.52	-0.93	4.18E-04	down
AC034236	ENSG0000018564	processed_pseudo;	46.6504	77.8960	0.60	-0.74	4.23E-04	down
AES	ENSG0000010496	retained_intron;pr	3.8070	6.3187	0.60	-0.73	4.25E-04	down
ABTB1	ENSG0000011462	protein_coding;ret	3.3219	1.8794	1.77	0.82	4.27E-04	up
ITGAM	ENSG0000016989	protein_coding;ret	3.2463	1.8278	1.78	0.83	4.31E-04	up
PSAP	ENSG0000019774	protein_coding;ret	228.9167	157.7060	1.45	0.54	4.31E-04	up
HP	ENSG0000025701	protein_coding;ret	8.1885	4.7721	1.72	0.78	4.31E-04	up
EXOSC6	ENSG0000022349	protein_coding	3.0380	5.2130	0.58	-0.78	4.33E-04	down
B3GALNT1	ENSG0000016925	protein_coding;ret	1.1092	0.5481	2.02	1.02	4.37E-04	up
SHTN1	ENSG0000018716	protein_coding;prc	1.2460	0.6219	2.00	1.00	4.48E-04	up
MME	ENSG0000019654	protein_coding;ret	0.2349	0.0765	3.07	1.62	4.49E-04	up
FAT1	ENSG0000008385	protein_coding;ret	2.2951	1.3789	1.66	0.73	4.51E-04	up
METAP2	ENSG0000011114	protein_coding;no	20.9862	31.1889	0.67	-0.57	4.51E-04	down
MPZL1	ENSG0000019796	protein_coding;ret	1.7861	0.5836	3.06	1.61	4.64E-04	up
CXCL2	ENSG0000008104	retained_intron;pr	0.2555	0.7946	0.32	-1.64	4.65E-04	down
CYB5A	ENSG0000016634	retained_intron;pr	2.1055	1.1998	1.75	0.81	4.65E-04	up
NPM1P27	ENSG0000024935	processed_pseudo;	69.7004	101.0036	0.69	-0.54	4.66E-04	down
KCNK5	ENSG0000016462	protein_coding	3.1883	5.6288	0.57	-0.82	4.68E-04	down
CNR2	ENSG0000018882	protein_coding	6.2274	3.8250	1.63	0.70	4.68E-04	up
ABCA13	ENSG0000017986	protein_coding;no	0.1505	0.0583	2.58	1.37	4.72E-04	up
EIF3E	ENSG0000010440	protein_coding;ret	140.3594	204.6070	0.69	-0.54	4.80E-04	down
C11orf21	ENSG0000011066	protein_coding;prc	16.6809	11.2709	1.48	0.57	4.86E-04	up
HELLS	ENSG0000011996	protein_coding;ret	2.4702	1.4473	1.71	0.77	4.89E-04	up
SLC25A24	ENSG0000008549	protein_coding;no	4.0379	6.2884	0.64	-0.64	4.91E-04	down
FAM72A	ENSG0000019655	protein_coding;prc	4.8550	8.1318	0.60	-0.74	5.04E-04	down
MTMR6	ENSG0000013950	protein_coding;prc	10.0136	14.4793	0.69	-0.53	5.15E-04	down
CEACAM4	ENSG0000010535	protein_coding;ret	6.7741	3.3727	2.01	1.01	5.23E-04	up
IL17RC	ENSG0000016370	protein_coding;no	0.0492	0.1581	0.31	-1.68	5.30E-04	down
LILRB2	ENSG0000013104	protein_coding;ret	1.8243	0.8299	2.20	1.14	5.31E-04	up
GYPC	ENSG0000013673	protein_coding;prc	14.7996	21.5128	0.69	-0.54	5.32E-04	down
NEU1	ENSG0000020438	retained_intron;pr	5.8972	3.5279	1.67	0.74	5.37E-04	up
COPS2	ENSG0000016620	protein_coding;ret	7.9052	11.5547	0.68	-0.55	5.40E-04	down
HEBP2	ENSG0000005162	protein_coding;no	4.6627	6.8008	0.69	-0.54	5.41E-04	down
SLC38A2	ENSG0000013429	protein_coding;no	26.4344	38.0713	0.69	-0.53	5.41E-04	down
TMEM38A	ENSG0000007295	protein_coding;ret	1.2725	0.5136	2.48	1.31	5.44E-04	up
PSAT1	ENSG0000013506	protein_coding	82.9357	119.8023	0.69	-0.53	5.53E-04	down
NPM1	ENSG0000018116	retained_intron;pr	223.4835	320.8496	0.70	-0.52	5.54E-04	down
HVCN1	ENSG0000012298	protein_coding;no	10.7563	6.9397	1.55	0.63	5.56E-04	up
PENK	ENSG0000018119	protein_coding;prc	0.6556	0.2504	2.62	1.39	5.62E-04	up
MTFR2	ENSG0000014641	protein_coding;no	5.1208	9.0584	0.57	-0.82	5.67E-04	down
RACK1	ENSG0000020462	protein_coding;ret	125.9441	183.2429	0.69	-0.54	5.68E-04	down
HACD2	ENSG0000020652	protein_coding;prc	7.1958	3.0361	2.37	1.24	5.70E-04	up

TOMM22	ENSG0000010021	protein_coding;ret	37.7363	53.8730	0.70	-0.51	5.74E-04	down
BLVRA	ENSG0000010660	protein_coding;ret	4.5403	8.0447	0.56	-0.83	6.00E-04	down
PTGER2	ENSG0000012538	protein_coding	9.1822	14.3804	0.64	-0.65	6.04E-04	down
KDELC1	ENSG0000013490	processed_transcri	9.5982	5.6301	1.70	0.77	6.15E-04	up
FBXO4	ENSG0000015187	protein_coding;ret	2.4113	4.3673	0.55	-0.86	6.15E-04	down
FFAR4	ENSG0000018618	protein_coding	0.4463	0.1537	2.90	1.54	6.24E-04	up
COL4A1	ENSG0000018749	protein_coding;ret	0.1916	0.4082	0.47	-1.09	6.32E-04	down
FOXC1	ENSG0000005459	protein_coding	1.2261	0.5012	2.45	1.29	6.34E-04	up
WBP2	ENSG0000013247	protein_coding;ret	8.4004	5.0514	1.66	0.73	6.45E-04	up
SULF2	ENSG0000019656	protein_coding;ret	4.0908	2.3567	1.74	0.80	6.50E-04	up
CCNL2	ENSG0000022197	protein_coding;ret	8.4236	13.5679	0.62	-0.69	6.56E-04	down
APOBEC3B	ENSG0000017975	protein_coding;no	4.0568	1.7957	2.26	1.18	6.56E-04	up
VHL	ENSG0000013408	protein_coding;pro	9.1498	5.3938	1.70	0.76	6.63E-04	up
SNHG29	ENSG0000017506	antisense;retained_	105.4178	151.3970	0.70	-0.52	6.63E-04	down
SLC1A5	ENSG0000010528	protein_coding	25.1522	36.2097	0.69	-0.53	6.68E-04	down
PCGF2	ENSG0000027725	protein_coding	0.1697	0.4918	0.35	-1.54	6.68E-04	down
NKAPD1	ENSG0000015077	protein_coding;ret	4.1793	6.7832	0.62	-0.70	6.71E-04	down
CCL23	ENSG0000027473	protein_coding;no	1.8681	4.5236	0.41	-1.28	6.75E-04	down
PABPC4	ENSG0000009062	protein_coding;no	9.5783	13.9208	0.69	-0.54	6.77E-04	down
AC012321	ENSG0000026077	3prime_overlappir	3.2555	0.1860	17.50	4.13	6.77E-04	up
EXOC4	ENSG0000013155	protein_coding;pro	6.0014	9.3086	0.64	-0.63	6.86E-04	down
BORCS8	ENSG0000025490	retained_intron;pro	2.9417	1.6338	1.80	0.85	6.91E-04	up
CSF2RB	ENSG0000010036	protein_coding	15.6155	10.7790	1.45	0.53	6.92E-04	up
SOD2	ENSG0000011209	protein_coding;pro	1.0714	1.8228	0.59	-0.77	7.06E-04	down
AP1M1	ENSG0000007295	protein_coding;ret	1.7234	2.7208	0.63	-0.66	7.08E-04	down
PRR7	ENSG0000013118	protein_coding	0.7563	1.9550	0.39	-1.37	7.18E-04	down
TXNDC5	ENSG0000023926	retained_intron;pro	98.4796	68.7880	1.43	0.52	7.36E-04	up
SFXN5	ENSG0000014404	processed_transcri	2.5693	1.5501	1.66	0.73	7.40E-04	up
IL23R	ENSG0000016259	protein_coding;no	3.5574	6.0940	0.58	-0.78	7.64E-04	down
AC090517	ENSG0000028533	processed_transcri	0.2038	0.0695	2.93	1.55	7.65E-04	up
HMGCR	ENSG0000011316	retained_intron;pro	9.4976	6.0594	1.57	0.65	7.69E-04	up
CECR7	ENSG0000023743	processed_transcri	2.1971	1.0511	2.09	1.06	7.70E-04	up
SIGLEC5	ENSG0000010550	protein_coding	5.6564	3.1437	1.80	0.85	7.79E-04	up
RNF135	ENSG0000018148	protein_coding	3.4628	6.2207	0.56	-0.85	7.88E-04	down
ELANE	ENSG0000019756	protein_coding	488.5498	325.1915	1.50	0.59	7.96E-04	up
LCP2	ENSG0000004346	retained_intron;pro	10.3120	16.6645	0.62	-0.69	8.02E-04	down
CHID1	ENSG0000017783	protein_coding;no	14.1265	9.5701	1.48	0.56	8.02E-04	up
ENC1	ENSG0000017161	protein_coding;pro	0.2650	0.5810	0.46	-1.13	8.04E-04	down
GJB6	ENSG0000012174	protein_coding	1.8234	0.8366	2.18	1.12	8.07E-04	up
ITGB2	ENSG0000016025	protein_coding;ret	23.7005	16.6063	1.43	0.51	8.10E-04	up
GNAS	ENSG0000008746	processed_transcri	22.1685	15.3074	1.45	0.53	8.11E-04	up
THAP9-AS1	ENSG0000025102	antisense	15.0727	21.6352	0.70	-0.52	8.12E-04	down
NID1	ENSG0000011696	protein_coding	0.1237	0.3691	0.33	-1.58	8.25E-04	down
DHRS9	ENSG0000007373	protein_coding	3.4328	1.6637	2.06	1.04	8.25E-04	up
AL441992	ENSG0000028611	protein_coding	0.0620	0.3154	0.20	-2.35	8.33E-04	down
TBCEL	ENSG0000015411	protein_coding;no	0.6913	1.4463	0.48	-1.06	8.34E-04	down
DCAF12	ENSG0000019887	protein_coding;pro	14.1510	20.1163	0.70	-0.51	8.35E-04	down
UGGT1	ENSG0000013673	nonsense_mediate	20.0808	14.0217	1.43	0.52	8.37E-04	up

DDOST	ENSG0000024403	protein_coding;pro	140.3562	96.6557	1.45	0.54	8.44E-04	up
BEX4	ENSG0000010240	protein_coding	3.4212	1.5098	2.27	1.18	8.49E-04	up
TMEM268	ENSG0000015769	protein_coding;pro	1.6241	2.9191	0.56	-0.85	8.52E-04	down
ITM2A	ENSG0000007859	protein_coding;pro	11.6989	6.8447	1.71	0.77	8.56E-04	up
SNTB1	ENSG0000017216	protein_coding;pro	3.0809	7.0757	0.44	-1.20	8.57E-04	down
KIAA0355	ENSG0000016639	protein_coding;pro	0.8703	0.4315	2.02	1.01	8.60E-04	up
CCSER2	ENSG0000010777	protein_coding;no	1.9465	1.1185	1.74	0.80	8.65E-04	up
C8orf88	ENSG0000025325	protein_coding	2.8377	1.1237	2.53	1.34	8.75E-04	up
ZNF44	ENSG0000019785	protein_coding;no	1.2668	0.6423	1.97	0.98	8.77E-04	up
ADO	ENSG0000018191	protein_coding	7.9408	11.7451	0.68	-0.56	8.88E-04	down
AP5S1	ENSG0000012584	protein_coding	0.5207	1.0960	0.48	-1.07	9.00E-04	down
ZBTB25	ENSG0000008977	protein_coding	0.4997	0.1815	2.75	1.46	9.01E-04	up
C3orf80	ENSG0000018004	protein_coding;no	0.5953	0.1465	4.06	2.02	9.08E-04	up
SMIM19	ENSG0000017620	protein_coding;no	2.7160	4.6619	0.58	-0.78	9.17E-04	down
NABP2	ENSG0000013957	protein_coding;ret	10.8296	17.1722	0.63	-0.67	9.33E-04	down
MPC1	ENSG0000006076	processed_transcri	29.3526	19.2751	1.52	0.61	9.41E-04	up
LRRC8D	ENSG0000017149	protein_coding	7.2978	16.4183	0.44	-1.17	9.45E-04	down
RPL35A	ENSG0000018289	protein_coding;ret	263.4460	377.8221	0.70	-0.52	9.46E-04	down
LENG8	ENSG0000016761	protein_coding;ret	24.2316	16.7823	1.44	0.53	9.65E-04	up
DSE	ENSG0000011181	protein_coding;ret	0.3559	0.1739	2.05	1.03	9.66E-04	up
AC093668	ENSG0000027294	nonsense_mediate	0.0122	0.2866	0.04	-4.56	9.68E-04	down
RBMX2	ENSG0000013459	protein_coding;pro	6.5760	10.8582	0.61	-0.72	9.79E-04	down
SFXN3	ENSG0000010781	protein_coding;pro	2.7989	4.7613	0.59	-0.77	9.79E-04	down
SLC22A15	ENSG0000016339	processed_transcri	10.5348	15.1347	0.70	-0.52	1.00E-03	down
SLC25A4	ENSG0000015172	protein_coding;no	1.2215	0.5889	2.07	1.05	1.01E-03	up
ATP9B	ENSG0000016637	retained_intron;pro	2.8935	1.9431	1.49	0.57	1.01E-03	up
ZCCHC7	ENSG0000014790	protein_coding;pro	6.6005	10.1467	0.65	-0.62	1.03E-03	down
SRRM2	ENSG0000016797	protein_coding;no	23.5429	13.5014	1.74	0.80	1.03E-03	up
BSG	ENSG0000017227	protein_coding;ret	75.0378	52.4398	1.43	0.52	1.03E-03	up
ABI2	ENSG0000013844	protein_coding;no	0.2116	0.0797	2.65	1.41	1.03E-03	up
CCNB1IP1	ENSG0000010081	protein_coding;no	19.1659	28.0015	0.68	-0.55	1.03E-03	down
E2F7	ENSG0000016589	protein_coding;ret	1.8317	0.9915	1.85	0.89	1.03E-03	up
ZNF253	ENSG0000025677	protein_coding;ret	3.0696	6.6843	0.46	-1.12	1.04E-03	down
ABLIM1	ENSG0000009920	protein_coding;ret	0.7924	0.2534	3.13	1.64	1.05E-03	up
CCDC26	ENSG0000022914	lincRNA	0.4233	0.8121	0.52	-0.94	1.05E-03	down
TRAF3IP3	ENSG0000000979	protein_coding;ret	5.9567	4.0471	1.47	0.56	1.05E-03	up
AQP9	ENSG0000010356	protein_coding;pro	7.1216	4.2272	1.68	0.75	1.05E-03	up
LILRA6	ENSG0000024448	protein_coding;no	0.9404	0.3899	2.41	1.27	1.06E-03	up
UPK3A	ENSG0000010037	protein_coding	13.9129	7.7680	1.79	0.84	1.07E-03	up
MTHFD2	ENSG0000006591	protein_coding;pro	71.5322	101.8702	0.70	-0.51	1.07E-03	down
SUMO2P17	ENSG0000024827	transcribed_proces	0.2319	1.2982	0.18	-2.48	1.09E-03	down
NUDT14	ENSG0000018382	protein_coding;ret	0.7737	0.2755	2.81	1.49	1.11E-03	up
SLC9A7	ENSG0000006592	protein_coding;pro	0.0787	0.2319	0.34	-1.56	1.11E-03	down
MYH15	ENSG0000014482	retained_intron;pro	2.0591	1.1485	1.79	0.84	1.11E-03	up
OSTC	ENSG0000019885	protein_coding;pro	58.0989	86.4481	0.67	-0.57	1.11E-03	down
THG1L	ENSG0000011327	protein_coding;no	4.0800	6.9601	0.59	-0.77	1.12E-03	down
IL18R1	ENSG0000011560	protein_coding;ret	2.1525	1.1516	1.87	0.90	1.12E-03	up
AL049830	ENSG0000025852	antisense	3.3788	1.5499	2.18	1.12	1.12E-03	up

ASPM	ENSG0000006627	protein_coding	5.1422	7.3134	0.70	-0.51	1.12E-03	down
ENPP3	ENSG0000015426	protein_coding;no	0.4846	0.1678	2.89	1.53	1.12E-03	up
OAS3	ENSG0000011133	protein_coding;pro	0.2128	0.0815	2.61	1.38	1.13E-03	up
SNHG7	ENSG0000023301	antisense	8.2574	12.1734	0.68	-0.56	1.13E-03	down
TEC	ENSG0000013560	protein_coding;no	2.2500	3.8657	0.58	-0.78	1.14E-03	down
CXCL8	ENSG0000016942	protein_coding;ret	95.5405	142.2234	0.67	-0.57	1.15E-03	down
STIL	ENSG0000012347	protein_coding;ret	5.4703	3.4407	1.59	0.67	1.15E-03	up
BCAT1	ENSG0000006098	protein_coding;pro	6.1676	9.0369	0.68	-0.55	1.17E-03	down
TRAPPC11	ENSG0000016853	retained_intron;pro	7.4857	10.7826	0.69	-0.53	1.19E-03	down
RRAGD	ENSG0000002503	protein_coding;pro	11.1707	16.5187	0.68	-0.56	1.20E-03	down
POMGNT2	ENSG0000014464	protein_coding	2.5537	1.3075	1.95	0.97	1.20E-03	up
AC092045	ENSG0000023955	processed_pseudo;	0.3495	0.9572	0.37	-1.45	1.23E-03	down
BST1	ENSG0000010974	protein_coding	27.9946	19.7386	1.42	0.50	1.23E-03	up
ACVR1	ENSG0000011517	protein_coding;pro	0.0501	0.1884	0.27	-1.91	1.23E-03	down
RNF175	ENSG0000014542	protein_coding;pro	0.1659	0.4188	0.40	-1.34	1.24E-03	down
JPT2	ENSG0000020605	protein_coding;pro	11.6153	3.7580	3.09	1.63	1.24E-03	up
SESN2	ENSG0000013076	protein_coding	3.1414	5.3593	0.59	-0.77	1.25E-03	down
KIAA1586	ENSG0000016811	protein_coding;pro	8.8625	14.6303	0.61	-0.72	1.25E-03	down
SNHG8	ENSG0000026989	lincRNA	31.3276	46.3843	0.68	-0.57	1.25E-03	down
ANKRD55	ENSG0000016451	protein_coding;pro	1.3211	2.5213	0.52	-0.93	1.26E-03	down
C6orf62	ENSG0000011230	protein_coding	33.3758	46.3845	0.72	-0.47	1.26E-03	down
EPHB6	ENSG0000010612	retained_intron;no	0.5705	0.2436	2.34	1.23	1.26E-03	up
VWA5A	ENSG0000011000	protein_coding;ret	1.4248	0.6996	2.04	1.03	1.26E-03	up
AC012651	ENSG0000025846	nonsense_mediate	2.1555	3.8506	0.56	-0.84	1.27E-03	down
ETNK1	ENSG0000013916	protein_coding;ret	5.6885	8.1405	0.70	-0.52	1.28E-03	down
ALOX5AP	ENSG0000013296	protein_coding;pro	189.6469	134.7331	1.41	0.49	1.29E-03	up
ADAP1	ENSG0000010596	retained_intron;pro	2.5232	4.1967	0.60	-0.73	1.29E-03	down
GNL3LP1	ENSG0000021503	processed_pseudo;	0.1508	0.5153	0.29	-1.77	1.32E-03	down
GPR65	ENSG0000014003	protein_coding	4.2386	2.5007	1.69	0.76	1.32E-03	up
CD276	ENSG0000010385	nonsense_mediate	0.0830	0.2584	0.32	-1.64	1.32E-03	down
MYADM	ENSG0000017982	protein_coding;pro	15.1665	8.8378	1.72	0.78	1.36E-03	up
UBA3	ENSG0000014474	retained_intron;pro	11.1601	17.7977	0.63	-0.67	1.37E-03	down
KIAA0930	ENSG0000010036	retained_intron;pro	14.1414	20.0476	0.71	-0.50	1.38E-03	down
EGR1	ENSG0000012073	protein_coding	5.4871	2.9407	1.87	0.90	1.38E-03	up
RPL21P16	ENSG0000022084	processed_pseudo;	250.5644	373.5926	0.67	-0.58	1.38E-03	down
ARPC4-TTL	ENSG0000025015	nonsense_mediate	3.4732	1.6373	2.12	1.08	1.40E-03	up
DENND1A	ENSG0000011952	protein_coding;pro	1.6273	2.5910	0.63	-0.67	1.40E-03	down
MARCH7	ENSG0000013653	processed_transcri	11.7788	17.4548	0.67	-0.57	1.41E-03	down
NECAB1	ENSG0000012311	protein_coding;pro	0.1436	0.0307	4.67	2.22	1.41E-03	up
RHOXF1P1	ENSG0000023449	processed_transcri	4.0872	2.0782	1.97	0.98	1.44E-03	up
HMGB1P10	ENSG0000021370	processed_pseudo;	9.6360	21.2682	0.45	-1.14	1.44E-03	down
TK1	ENSG0000016790	protein_coding;ret	31.3981	21.8096	1.44	0.53	1.44E-03	up
RAVER2	ENSG0000016243	protein_coding;no	5.4522	3.4130	1.60	0.68	1.46E-03	up
THBS4	ENSG0000011329	processed_transcri	1.0093	1.8729	0.54	-0.89	1.48E-03	down
ST7-AS1	ENSG0000022719	antisense	0.1435	0.4647	0.31	-1.69	1.48E-03	down
FCGR1B	ENSG0000019801	processed_transcri	2.4737	4.4853	0.55	-0.86	1.49E-03	down
NCK2	ENSG0000007105	protein_coding	3.7750	7.5478	0.50	-1.00	1.49E-03	down
SNHG12	ENSG0000019798	antisense;retained_	2.3034	3.8481	0.60	-0.74	1.49E-03	down

AKR1B1	ENSG0000008566	protein_coding;ret	0.6368	1.4681	0.43	-1.21	1.50E-03	down
CBR1	ENSG0000015922	protein_coding;pro	14.3685	9.4032	1.53	0.61	1.50E-03	up
TMEM106B	ENSG0000010646	protein_coding;ret	1.2272	2.1155	0.58	-0.79	1.51E-03	down
PDE1B	ENSG0000012336	protein_coding;pro	0.1582	0.3790	0.42	-1.26	1.52E-03	down
ADCY4	ENSG0000012946	nonsense_mediate	0.6435	0.3150	2.04	1.03	1.53E-03	up
HPRT1	ENSG0000016570	protein_coding;pro	42.6530	59.8600	0.71	-0.49	1.53E-03	down
GARS	ENSG0000010610	nonsense_mediate	34.3712	47.6154	0.72	-0.47	1.55E-03	down
ZC3H6	ENSG0000018817	protein_coding;ret	0.2617	0.5948	0.44	-1.18	1.55E-03	down
CRELD2	ENSG0000018416	retained_intron;pro	3.8997	2.2704	1.72	0.78	1.55E-03	up
ATF4	ENSG0000012827	protein_coding	218.1419	304.2272	0.72	-0.48	1.56E-03	down
RSL1D1	ENSG0000017149	retained_intron;pro	37.4966	53.2898	0.70	-0.51	1.56E-03	down
SLC3A2	ENSG0000016800	protein_coding;no	23.2090	32.0506	0.72	-0.47	1.57E-03	down
TVP23C-CD	ENSG0000025902	protein_coding;no	2.0444	0.2693	7.59	2.92	1.57E-03	up
ESD	ENSG0000013968	protein_coding;pro	36.2072	50.6478	0.71	-0.48	1.57E-03	down
PTP4A3	ENSG0000018448	protein_coding	2.9648	1.4979	1.98	0.99	1.58E-03	up
ARHGAP22	ENSG0000012880	protein_coding;pro	0.1282	0.0434	2.95	1.56	1.59E-03	up
CCNC	ENSG0000011223	protein_coding;no	5.7766	8.3324	0.69	-0.53	1.62E-03	down
PTPDC1	ENSG0000015807	protein_coding;pro	0.8417	0.4060	2.07	1.05	1.62E-03	up
CCND2	ENSG0000011897	protein_coding;ret	12.3939	17.1533	0.72	-0.47	1.63E-03	down
LPIN1	ENSG0000013432	protein_coding;ret	0.6786	0.3574	1.90	0.93	1.63E-03	up
MRPL33	ENSG0000024314	processed_transcri	36.5444	51.3553	0.71	-0.49	1.64E-03	down
PREPL	ENSG0000013807	nonsense_mediate	9.2413	5.8442	1.58	0.66	1.64E-03	up
AC022148	ENSG0000018045	antisense	0.2802	0.0512	5.48	2.45	1.64E-03	up
RF02104	ENSG0000027354	misc_RNA	12.3302	22.9008	0.54	-0.89	1.65E-03	down
HAUS5	ENSG0000024911	retained_intron;pro	4.4001	2.5802	1.71	0.77	1.65E-03	up
PLXNA3	ENSG0000013082	protein_coding;ret	0.2466	0.1061	2.33	1.22	1.66E-03	up
CDK6	ENSG0000010581	protein_coding;ret	7.9658	11.1815	0.71	-0.49	1.66E-03	down
CISD2	ENSG0000014535	protein_coding;no	5.1782	2.8023	1.85	0.89	1.67E-03	up
NFKBIZ	ENSG0000014480	protein_coding;ret	0.9199	0.4040	2.28	1.19	1.71E-03	up
SNX5	ENSG0000008900	processed_transcri	14.7315	20.2678	0.73	-0.46	1.72E-03	down
RPF2	ENSG0000019749	nonsense_mediate	13.7704	19.6370	0.70	-0.51	1.73E-03	down
UBE2L6	ENSG0000015658	protein_coding;ret	7.5133	12.3345	0.61	-0.72	1.73E-03	down
RABGGTB	ENSG0000013795	processed_transcri	12.5791	17.3411	0.73	-0.46	1.75E-03	down
CALCOCO1	ENSG0000001282	protein_coding;ret	3.9472	2.5860	1.53	0.61	1.75E-03	up
CEBPB	ENSG0000017221	protein_coding	13.2681	19.9534	0.66	-0.59	1.76E-03	down
TSPAN4	ENSG0000021406	protein_coding;ret	2.0846	1.1924	1.75	0.81	1.76E-03	up
MS4A4E	ENSG0000021478	protein_coding;pro	0.1022	0.3499	0.29	-1.77	1.78E-03	down
LILRB3	ENSG0000020457	protein_coding;ret	1.3340	0.6162	2.16	1.11	1.78E-03	up
ZDHHC16	ENSG0000017130	protein_coding;pro	10.6884	6.2159	1.72	0.78	1.80E-03	up
ZNF347	ENSG0000019793	protein_coding;ret	0.2256	0.0924	2.44	1.29	1.80E-03	up
RPL4	ENSG0000017444	retained_intron;pro	301.4217	414.9412	0.73	-0.46	1.81E-03	down
MRPL18	ENSG0000011211	processed_transcri	61.2830	84.7276	0.72	-0.47	1.82E-03	down
CD59	ENSG0000008506	protein_coding;ret	12.6968	8.0083	1.59	0.66	1.82E-03	up
HBEGF	ENSG0000011307	protein_coding;ret	2.3167	4.1864	0.55	-0.85	1.83E-03	down
MPP3	ENSG0000016164	retained_intron;no	0.0789	0.2725	0.29	-1.79	1.83E-03	down
MANF	ENSG0000014505	nonsense_mediate	23.4171	16.3531	1.43	0.52	1.84E-03	up
MYBL1	ENSG0000018569	protein_coding;pro	0.2342	0.0827	2.83	1.50	1.85E-03	up
MTHFD1L	ENSG0000012025	protein_coding;pro	8.8879	12.4599	0.71	-0.49	1.92E-03	down

S100A4	ENSG0000019615	protein_coding;pr	116.6399	80.7996	1.44	0.53	1.94E-03	up
ACP5	ENSG0000010257	protein_coding;ret	0.5309	0.1986	2.67	1.42	1.95E-03	up
IARS	ENSG0000019630	protein_coding;pr	36.4970	50.3582	0.72	-0.46	1.96E-03	down
MED20	ENSG0000012464	protein_coding;pr	12.4964	7.8056	1.60	0.68	1.97E-03	up
RPL4P4	ENSG0000022963	processed_pseudo	18.0293	27.2495	0.66	-0.60	1.97E-03	down
TM9SF4	ENSG0000010133	retained_intron;pr	5.6540	8.1135	0.70	-0.52	1.98E-03	down
AC093849	ENSG0000028603	lincRNA	0.1822	0.6442	0.28	-1.82	1.99E-03	down
KLHL23	ENSG0000021316	protein_coding;ret	4.3567	2.6607	1.64	0.71	2.00E-03	up
TIMM9	ENSG0000010057	protein_coding;pr	14.8410	23.9183	0.62	-0.69	2.01E-03	down
CCDC43	ENSG0000018032	protein_coding;ret	8.9427	14.4655	0.62	-0.69	2.02E-03	down
RPS25	ENSG0000011818	nonsense_mediate	516.5259	716.2976	0.72	-0.47	2.05E-03	down
GAS2L3	ENSG0000013935	protein_coding;no	0.3834	0.8774	0.44	-1.19	2.06E-03	down
CHST10	ENSG0000011552	protein_coding;ret	1.2197	0.6335	1.93	0.95	2.06E-03	up
RPIA	ENSG0000015357	protein_coding	27.1772	38.2991	0.71	-0.49	2.08E-03	down
AC008763	ENSG0000026971	protein_coding	7.7284	1.1218	6.89	2.78	2.08E-03	up
MICAL1	ENSG0000013559	retained_intron;pr	3.3619	5.0630	0.66	-0.59	2.09E-03	down
EML3	ENSG0000014949	protein_coding;ret	2.5758	4.1282	0.62	-0.68	2.10E-03	down
RNASE6	ENSG0000016941	protein_coding	4.6261	8.6754	0.53	-0.91	2.10E-03	down
EIF3H	ENSG0000014767	protein_coding;ret	54.0958	78.5585	0.69	-0.54	2.13E-03	down
CLEC5A	ENSG0000025822	protein_coding;ret	72.1372	99.6071	0.72	-0.47	2.14E-03	down
CST7	ENSG0000007798	protein_coding	557.3984	402.8011	1.38	0.47	2.15E-03	up
EIF5	ENSG0000010066	retained_intron;pr	12.2819	17.1818	0.71	-0.48	2.15E-03	down
PLPP1	ENSG0000006711	protein_coding;ret	6.9673	4.0235	1.73	0.79	2.16E-03	up
LCMT2	ENSG0000016880	protein_coding	0.7229	1.3198	0.55	-0.87	2.16E-03	down
ATP1B1	ENSG0000014315	protein_coding	0.5594	0.1102	5.08	2.34	2.16E-03	up
LINC00599	ENSG0000025323	lincRNA	2.8563	1.5539	1.84	0.88	2.18E-03	up
RAD9B	ENSG0000015116	protein_coding;no	0.3765	0.8687	0.43	-1.21	2.18E-03	down
HERC6	ENSG0000013864	protein_coding;no	0.9340	0.4823	1.94	0.95	2.18E-03	up
ECHDC2	ENSG0000012131	retained_intron;no	0.6443	0.3297	1.95	0.97	2.19E-03	up
FOSL2	ENSG0000007542	protein_coding;pr	3.4293	6.2821	0.55	-0.87	2.20E-03	down
JAG1	ENSG0000010138	retained_intron;pr	3.6029	5.2010	0.69	-0.53	2.23E-03	down
PIF1	ENSG0000014045	protein_coding;ret	0.1890	0.4453	0.42	-1.24	2.23E-03	down
BCL11A	ENSG0000011986	protein_coding;pr	0.5848	0.9362	0.62	-0.68	2.23E-03	down
TUBG1	ENSG0000013146	retained_intron;pr	17.0237	11.4427	1.49	0.57	2.26E-03	up
RAMP1	ENSG0000013232	protein_coding	1.5979	0.6118	2.61	1.39	2.26E-03	up
LMAN2	ENSG0000016922	protein_coding;ret	42.8587	29.9122	1.43	0.52	2.26E-03	up
NRIP3	ENSG0000017535	protein_coding	7.4926	4.8623	1.54	0.62	2.27E-03	up
PHOSPHO2	ENSG0000014436	protein_coding;pr	2.4098	1.1625	2.07	1.05	2.27E-03	up
LSS	ENSG0000016028	retained_intron;pr	7.9211	4.8509	1.63	0.71	2.28E-03	up
MARCH2	ENSG0000009978	protein_coding;ret	2.3088	1.1769	1.96	0.97	2.29E-03	up
TMEM68	ENSG0000016790	protein_coding;ret	1.9692	3.1391	0.63	-0.67	2.30E-03	down
SETD5	ENSG0000016813	protein_coding;no	3.6645	2.3553	1.56	0.64	2.30E-03	up
RAD51B	ENSG0000018218	protein_coding;pr	0.4863	0.9191	0.53	-0.92	2.33E-03	down
SUMF2	ENSG0000012910	protein_coding;no	30.9952	21.7426	1.43	0.51	2.33E-03	up
ZSWIM4	ENSG0000013200	protein_coding	0.3457	0.1046	3.30	1.72	2.34E-03	up
TARS	ENSG0000011340	nonsense_mediate	32.6104	44.8114	0.73	-0.46	2.35E-03	down
EXT2	ENSG0000015134	protein_coding;ret	4.9079	3.0720	1.60	0.68	2.38E-03	up
IL7R	ENSG0000016868	protein_coding;pr	0.1787	0.0485	3.69	1.88	2.39E-03	up

ZNF213	ENSG0000008564	protein_coding;ret	1.2654	0.6822	1.85	0.89	2.39E-03	up
TFB1M	ENSG0000002963	processed_transcri	3.8079	6.0143	0.63	-0.66	2.39E-03	down
MZB1	ENSG0000017047	nonsense_mediate	7.6848	4.6316	1.66	0.73	2.39E-03	up
CCNB1	ENSG0000013405	protein_coding;no	13.7195	19.4843	0.70	-0.51	2.40E-03	down
CAMK1	ENSG0000013407	nonsense_mediate	20.9543	14.6233	1.43	0.52	2.40E-03	up
SEC11A	ENSG0000014061	protein_coding;ret	20.1730	27.5488	0.73	-0.45	2.41E-03	down
UBB	ENSG0000017031	protein_coding;pro	112.2732	81.3541	1.38	0.46	2.42E-03	up
DRAM1	ENSG0000013604	protein_coding;no	3.0754	1.6335	1.88	0.91	2.43E-03	up
ARF1	ENSG0000014376	protein_coding;pro	60.9147	34.4449	1.77	0.82	2.43E-03	up
RGL2	ENSG0000023744	retained_intron;pro	6.5843	4.2010	1.57	0.65	2.45E-03	up
DDIT3	ENSG0000017519	protein_coding	26.2490	38.5772	0.68	-0.56	2.45E-03	down
PLEKHA8P	ENSG0000013429	transcribed_proces	2.6457	1.4089	1.88	0.91	2.49E-03	up
RAB4A	ENSG0000016811	protein_coding;pro	11.3468	17.3898	0.65	-0.62	2.52E-03	down
BLM	ENSG0000019729	nonsense_mediate	6.9823	9.6867	0.72	-0.47	2.53E-03	down
NOMO3	ENSG0000010322	nonsense_mediate	8.6783	6.1383	1.41	0.50	2.54E-03	up
MAP7D2	ENSG0000018436	protein_coding;pro	0.4406	0.1818	2.42	1.28	2.55E-03	up
AMOTL1	ENSG0000016602	protein_coding;pro	0.8251	0.4384	1.88	0.91	2.56E-03	up
RPL24P4	ENSG0000018152	processed_pseudo	74.6202	112.0507	0.67	-0.59	2.56E-03	down
DHRS11	ENSG0000027853	protein_coding;ret	1.6041	0.8975	1.79	0.84	2.58E-03	up
FHDC1	ENSG0000013746	protein_coding	0.2007	0.0577	3.48	1.80	2.58E-03	up
SEPT8	ENSG0000016440	protein_coding;pro	1.5588	0.9130	1.71	0.77	2.61E-03	up
AC004803	ENSG0000025013	antisense	0.2692	0.6232	0.43	-1.21	2.61E-03	down
RPL23AP42	ENSG0000023485	processed_pseudo	80.6077	122.5888	0.66	-0.60	2.64E-03	down
STX3	ENSG0000016690	protein_coding;ret	8.3488	4.5610	1.83	0.87	2.68E-03	up
R3HDM2	ENSG0000017991	retained_intron;pro	3.8917	5.8754	0.66	-0.59	2.70E-03	down
STARD3NL	ENSG0000001027	protein_coding;ret	10.6071	14.6354	0.72	-0.46	2.72E-03	down
PLD3	ENSG0000010522	protein_coding;pro	3.7890	2.2403	1.69	0.76	2.72E-03	up
SNX33	ENSG0000017354	protein_coding	0.1023	0.2514	0.41	-1.30	2.73E-03	down
SLC1A4	ENSG0000011590	protein_coding;ret	8.5468	11.8250	0.72	-0.47	2.74E-03	down
HSD17B7	ENSG0000013219	processed_transcri	3.0342	1.7951	1.69	0.76	2.76E-03	up
KIAA1147	ENSG0000025709	protein_coding	3.1561	1.9846	1.59	0.67	2.77E-03	up
DECR2	ENSG0000024261	retained_intron;pro	2.3352	1.2499	1.87	0.90	2.77E-03	up
ERMAP	ENSG0000016401	protein_coding;ret	0.8234	1.4900	0.55	-0.86	2.79E-03	down
ARHGAP25	ENSG0000016321	protein_coding;pro	10.4997	7.3881	1.42	0.51	2.82E-03	up
SCAMP1	ENSG0000008536	protein_coding;no	2.7567	5.3361	0.52	-0.95	2.83E-03	down
CU633904	ENSG0000027607	lincRNA	0.5025	0.1305	3.85	1.95	2.85E-03	up
CABLES2	ENSG0000014967	protein_coding	1.1811	0.5281	2.24	1.16	2.85E-03	up
L2HGDH	ENSG0000008729	protein_coding;no	5.5298	3.6762	1.50	0.59	2.86E-03	up
MIER1	ENSG0000019816	protein_coding;pro	5.6900	3.7924	1.50	0.59	2.87E-03	up
BCL9L	ENSG0000018617	protein_coding;pro	0.4453	0.2132	2.09	1.06	2.88E-03	up
SLFN13	ENSG0000015476	retained_intron;no	25.5780	18.6769	1.37	0.45	2.89E-03	up
RBM4B	ENSG0000017391	protein_coding;no	7.9053	4.9651	1.59	0.67	2.91E-03	up
LINC01268	ENSG0000022750	lincRNA	0.8822	1.7701	0.50	-1.00	2.94E-03	down
PDK1	ENSG0000015225	protein_coding;ret	2.9003	4.0768	0.71	-0.49	2.97E-03	down
SYNCRIP	ENSG0000013531	protein_coding	35.3741	48.0206	0.74	-0.44	2.99E-03	down
POMGNT1	ENSG0000008599	protein_coding;pro	1.4439	2.4936	0.58	-0.79	2.99E-03	down
NUCB1	ENSG0000010480	protein_coding;ret	25.0882	17.8882	1.40	0.49	3.01E-03	up
C1orf54	ENSG0000011829	protein_coding	0.4997	1.2398	0.40	-1.31	3.02E-03	down

KCTD21	ENSG0000018899	protein_coding	0.5718	0.2299	2.49	1.31	3.03E-03	up
MELTF-AS	ENSG0000022810	antisense	1.5215	0.5975	2.55	1.35	3.03E-03	up
AC005837	ENSG0000026133	antisense	0.4916	0.1662	2.96	1.56	3.05E-03	up
AGRN	ENSG0000018815	retained_intron;pr	0.7031	0.3814	1.84	0.88	3.05E-03	up
FGD3	ENSG0000012708	nonsense_mediate	3.6229	2.2617	1.60	0.68	3.06E-03	up
ARG1	ENSG0000011852	protein_coding;pr	13.9829	8.9597	1.56	0.64	3.07E-03	up
PLP2	ENSG0000010200	protein_coding	93.7348	67.5659	1.39	0.47	3.07E-03	up
ELOC	ENSG0000015458	protein_coding	7.5834	10.7064	0.71	-0.50	3.09E-03	down
UPB1	ENSG0000010002	nonsense_mediate	0.4109	0.1865	2.20	1.14	3.09E-03	up
SPIN1	ENSG0000010672	processed_transcri	7.1905	2.9547	2.43	1.28	3.10E-03	up
ADAM9	ENSG0000016861	protein_coding;no	12.3042	8.8933	1.38	0.47	3.10E-03	up
ORAI3	ENSG0000017593	protein_coding;no	1.1980	3.4002	0.35	-1.50	3.12E-03	down
FKTN	ENSG0000010669	protein_coding;no	0.6855	1.8589	0.37	-1.44	3.12E-03	down
ARHGAP11	ENSG0000019882	protein_coding;pr	2.4540	3.9546	0.62	-0.69	3.13E-03	down
GPR84	ENSG0000013957	protein_coding	3.7484	2.0524	1.83	0.87	3.14E-03	up
C7orf31	ENSG0000015379	protein_coding	0.7320	1.3822	0.53	-0.92	3.16E-03	down
RPL7P1	ENSG0000021448	processed_pseudo	428.7221	582.8413	0.74	-0.44	3.20E-03	down
ZNF629	ENSG0000010287	protein_coding	1.0466	1.8246	0.57	-0.80	3.22E-03	down
C21orf59-T(	ENSG0000026559	protein_coding	3.3166	0.1347	24.62	4.62	3.22E-03	up
AL135905	ENSG0000028597	protein_coding	3.5823	5.6494	0.63	-0.66	3.23E-03	down
TCAM1P	ENSG0000024028	processed_transcri	2.2956	1.1294	2.03	1.02	3.24E-03	up
LRRC37B	ENSG0000018515	protein_coding;ret	1.8159	3.0905	0.59	-0.77	3.24E-03	down
GLCE	ENSG0000013860	protein_coding;pr	1.4810	0.7944	1.86	0.90	3.26E-03	up
MAP4K1	ENSG0000010481	protein_coding;ret	0.1699	0.4071	0.42	-1.26	3.27E-03	down
CSKMT	ENSG0000021475	protein_coding;pr	0.3465	1.0511	0.33	-1.60	3.28E-03	down
PMAIP1	ENSG0000014168	protein_coding;ret	15.4828	28.5624	0.54	-0.88	3.28E-03	down
SNRNP25	ENSG0000016198	retained_intron;pr	20.7770	14.5209	1.43	0.52	3.29E-03	up
ZNF641	ENSG0000016752	protein_coding;ret	1.7912	0.6662	2.69	1.43	3.29E-03	up
TUBB4B	ENSG0000018822	protein_coding;ret	78.7733	56.7405	1.39	0.47	3.30E-03	up
SMN2	ENSG0000020557	protein_coding;ret	7.2221	12.9478	0.56	-0.84	3.30E-03	down
PRADC1	ENSG0000013561	protein_coding;pr	13.8255	8.5578	1.62	0.69	3.31E-03	up
FN3KRP	ENSG0000014156	protein_coding;ret	12.8059	8.5082	1.51	0.59	3.32E-03	up
LMO2	ENSG0000013536	protein_coding;ret	17.4875	32.8872	0.53	-0.91	3.34E-03	down
SFMBT2	ENSG0000019887	protein_coding	1.3841	2.1770	0.64	-0.65	3.34E-03	down
AL591848	ENSG0000026085	lincRNA	0.6911	0.2871	2.41	1.27	3.36E-03	up
TULP3	ENSG0000007824	protein_coding;no	2.9407	1.5777	1.86	0.90	3.36E-03	up
SSR4	ENSG0000018087	processed_transcri	27.3504	19.8133	1.38	0.47	3.36E-03	up
SATB1-AS1	ENSG0000022895	antisense;retained_	0.1789	0.0791	2.26	1.18	3.40E-03	up
COPS8	ENSG0000019861	protein_coding;ret	5.4086	8.6126	0.63	-0.67	3.42E-03	down
CARD16	ENSG0000020439	protein_coding;ret	2.1585	3.8901	0.55	-0.85	3.43E-03	down
ASNS	ENSG0000007066	protein_coding;ret	20.0390	27.3172	0.73	-0.45	3.44E-03	down
IBTK	ENSG0000000570	retained_intron;pr	6.7650	9.3725	0.72	-0.47	3.46E-03	down
AAGAB	ENSG0000010359	processed_transcri	8.4231	12.3542	0.68	-0.55	3.47E-03	down
SH3D21	ENSG0000021419	protein_coding;no	0.2044	0.0737	2.77	1.47	3.48E-03	up
HDGFL3	ENSG0000016650	protein_coding;ret	0.1736	0.0737	2.36	1.24	3.50E-03	up
MSRB3	ENSG0000017409	protein_coding;pr	6.8092	13.7613	0.49	-1.02	3.52E-03	down
PTGR1	ENSG0000010685	nonsense_mediate	4.8079	2.9890	1.61	0.69	3.52E-03	up
ERO1B	ENSG0000008661	protein_coding	0.3919	0.1594	2.46	1.30	3.52E-03	up

DNAJB5	ENSG0000013709	retained_intron;pr	0.3241	0.1343	2.41	1.27	3.53E-03	up
GSN	ENSG0000014818	protein_coding;no	11.6699	8.4559	1.38	0.46	3.53E-03	up
AARS	ENSG0000009086	protein_coding;ret	51.2754	69.6356	0.74	-0.44	3.56E-03	down
NOMO1	ENSG0000010351	protein_coding;ret	19.0838	13.4583	1.42	0.50	3.57E-03	up
DHRS1	ENSG0000015737	nonsense_mediate	3.8035	2.2716	1.67	0.74	3.57E-03	up
AL354740	ENSG0000022533	retained_intron;an	0.0884	4.7479	0.02	-5.75	3.61E-03	down
RPS3AP26	ENSG0000021438	processed_pseudo	60.7986	85.9003	0.71	-0.50	3.65E-03	down
CAMLG	ENSG0000016461	protein_coding	5.9832	9.6362	0.62	-0.69	3.66E-03	down
RGL4	ENSG0000015949	protein_coding;no	2.1086	3.4136	0.62	-0.69	3.67E-03	down
NMI	ENSG0000012360	protein_coding;ret	15.6040	21.4959	0.73	-0.46	3.68E-03	down
STK39	ENSG0000019864	protein_coding;pr	2.4488	4.0063	0.61	-0.71	3.69E-03	down
GAREM2	ENSG0000015783	protein_coding;pr	0.4293	0.1980	2.17	1.12	3.70E-03	up
PAIP2B	ENSG0000012437	protein_coding	0.2712	0.1027	2.64	1.40	3.72E-03	up
MAEA	ENSG0000009031	protein_coding;no	5.5829	7.7249	0.72	-0.47	3.73E-03	down
ELOB	ENSG0000010336	protein_coding	100.3485	73.7563	1.36	0.44	3.73E-03	up
AC091057	ENSG0000026993	lincRNA	1.0142	2.3041	0.44	-1.18	3.76E-03	down
TRIOBP	ENSG0000010010	protein_coding;ret	2.1127	1.3497	1.57	0.65	3.77E-03	up
PLS1	ENSG0000012075	protein_coding;ret	2.9514	1.8312	1.61	0.69	3.79E-03	up
CYBA	ENSG0000005152	protein_coding;ret	25.7398	18.5897	1.38	0.47	3.82E-03	up
TRIM6	ENSG0000012123	protein_coding;pr	2.2376	1.1630	1.92	0.94	3.83E-03	up
OLMALINC	ENSG0000023582	lincRNA	0.2220	0.5120	0.43	-1.21	3.88E-03	down
ITM2B	ENSG0000013615	protein_coding;no	22.8185	15.8417	1.44	0.53	3.88E-03	up
BAIAP2-DT	ENSG0000022613	lincRNA	0.4350	0.9162	0.47	-1.07	3.89E-03	down
LIPA	ENSG0000010779	protein_coding;pr	2.6272	4.1828	0.63	-0.67	3.91E-03	down
CPEB2	ENSG0000013744	protein_coding;ret	0.5985	1.3206	0.45	-1.14	3.94E-03	down
STK24	ENSG0000010257	protein_coding;pr	4.1398	2.7725	1.49	0.58	3.94E-03	up
MGAT5	ENSG0000015212	protein_coding;pr	1.9231	3.6232	0.53	-0.91	3.94E-03	down
HSPA1A	ENSG0000020438	protein_coding	1.7495	0.9012	1.94	0.96	3.95E-03	up
CBX2	ENSG0000017389	protein_coding;ret	0.7870	0.4085	1.93	0.95	3.95E-03	up
TSPAN2	ENSG0000013419	protein_coding;pr	3.2632	5.3284	0.61	-0.71	3.96E-03	down
HEATR5B	ENSG0000000886	protein_coding;ret	6.2194	8.4456	0.74	-0.44	3.97E-03	down
HOMER3	ENSG0000005112	protein_coding;ret	9.1184	13.0035	0.70	-0.51	3.98E-03	down
LINC01215	ENSG0000027185	lincRNA	10.6833	7.0391	1.52	0.60	4.00E-03	up
TRPS1	ENSG0000010444	protein_coding	0.4556	0.2246	2.03	1.02	4.01E-03	up
RPL13AP25	ENSG0000013614	processed_pseudo	50.4821	72.6560	0.69	-0.53	4.03E-03	down
GM2A	ENSG0000019674	protein_coding	15.2103	20.6025	0.74	-0.44	4.03E-03	down
RCAN3	ENSG0000011760	protein_coding;no	0.4123	0.1891	2.18	1.12	4.03E-03	up
FAM72B	ENSG0000018861	protein_coding;pr	5.6281	8.9641	0.63	-0.67	4.04E-03	down
PARD3	ENSG0000014849	protein_coding;ret	0.8359	0.4467	1.87	0.90	4.05E-03	up
UBE2W	ENSG0000010434	nonsense_mediate	3.8320	5.3637	0.71	-0.49	4.06E-03	down
ATP6V0E2	ENSG0000017113	protein_coding;pr	0.5835	0.2771	2.11	1.07	4.09E-03	up
LAMP1	ENSG0000018589	protein_coding;ret	22.5459	15.7211	1.43	0.52	4.11E-03	up
QPCT	ENSG0000011582	protein_coding;ret	3.2335	1.7818	1.81	0.86	4.12E-03	up
SNHG4	ENSG0000028139	lincRNA	6.2555	8.9503	0.70	-0.52	4.13E-03	down
TIMELESS	ENSG0000011160	protein_coding;ret	19.4571	14.3441	1.36	0.44	4.13E-03	up
AC112128	ENSG0000028594	nonsense_mediate	0.2433	0.8256	0.29	-1.76	4.13E-03	down
TBL1XR1	ENSG0000017756	nonsense_mediate	6.2771	8.6276	0.73	-0.46	4.13E-03	down
LETMD1	ENSG0000005042	nonsense_mediate	10.2589	13.8492	0.74	-0.43	4.14E-03	down

ERG28	ENSG0000013393	protein_coding	14.8311	9.9787	1.49	0.57	4.16E-03	up
AUXG0100(	ENSG0000028621	antisense	0.1109	0.0337	3.29	1.72	4.17E-03	up
SLC25A32	ENSG0000016493	protein_coding;no	5.6763	8.9748	0.63	-0.66	4.19E-03	down
PRELID1	ENSG0000016923	protein_coding;no	30.8111	44.7576	0.69	-0.54	4.19E-03	down
ZWINT	ENSG0000012295	retained_intron;no	41.7176	30.4899	1.37	0.45	4.20E-03	up
C2CD3	ENSG0000016801	protein_coding;pro	2.3720	1.5524	1.53	0.61	4.20E-03	up
CNTNAP4	ENSG0000015291	protein_coding;ret	2.0799	3.1831	0.65	-0.61	4.20E-03	down
SDF2L1	ENSG0000012822	protein_coding;ret	13.0808	7.8418	1.67	0.74	4.25E-03	up
CD300LB	ENSG0000017878	protein_coding	6.0957	3.8347	1.59	0.67	4.26E-03	up
ATXN7	ENSG0000016363	protein_coding;ret	1.5616	0.8677	1.80	0.85	4.27E-03	up
TMEM260	ENSG0000007026	protein_coding;no	1.2996	2.1436	0.61	-0.72	4.29E-03	down
FAM161A	ENSG0000017026	protein_coding;ret	2.1327	1.2590	1.69	0.76	4.32E-03	up
ACSL1	ENSG0000015172	protein_coding;ret	15.9965	22.9377	0.70	-0.52	4.32E-03	down
ATP10D	ENSG0000014524	protein_coding;no	1.6768	0.9314	1.80	0.85	4.33E-03	up
FBXO36	ENSG0000015383	protein_coding;ret	0.0925	0.2627	0.35	-1.51	4.33E-03	down
ZNF578	ENSG0000025840	protein_coding;pro	0.3563	0.1732	2.06	1.04	4.35E-03	up
CHST4	ENSG0000014083	protein_coding;ret	1.0524	0.4927	2.14	1.10	4.41E-03	up
PRKCQ-AS	ENSG0000023794	lincRNA;retained_	0.3604	0.8140	0.44	-1.18	4.46E-03	down
AC007342	ENSG0000026007	retained_intron;tra	0.7466	0.3573	2.09	1.06	4.48E-03	up
NACA	ENSG0000019653	protein_coding;pro	46.6991	62.8705	0.74	-0.43	4.52E-03	down
SLU7	ENSG0000016460	protein_coding;ret	9.4013	12.9852	0.72	-0.47	4.52E-03	down
LIMS1	ENSG0000016975	protein_coding;pro	3.5845	2.2388	1.60	0.68	4.53E-03	up
RPL24	ENSG0000011439	protein_coding;ret	208.4791	284.3784	0.73	-0.45	4.53E-03	down
RPL9	ENSG0000016368	protein_coding;ret	210.4929	284.2054	0.74	-0.43	4.55E-03	down
SMCHD1	ENSG0000010159	nonsense_mediate	19.4599	26.3996	0.74	-0.44	4.56E-03	down
FKBP2	ENSG0000017348	nonsense_mediate	14.5527	9.2280	1.58	0.66	4.57E-03	up
UNC13B	ENSG0000019872	protein_coding;pro	1.4359	0.9305	1.54	0.63	4.57E-03	up
CALHM2	ENSG0000013817	processed_transcri	1.5045	2.6557	0.57	-0.82	4.59E-03	down
PLD4	ENSG0000016642	protein_coding;ret	0.0901	0.2668	0.34	-1.57	4.59E-03	down
XKR3	ENSG0000017296	protein_coding	0.6679	0.2339	2.85	1.51	4.60E-03	up
AIG1	ENSG0000014641	protein_coding;ret	11.4561	15.3239	0.75	-0.42	4.64E-03	down
ATL3	ENSG0000018474	protein_coding;pro	9.9654	13.3283	0.75	-0.42	4.64E-03	down
AC092683	ENSG0000023060	lincRNA	2.0833	1.1602	1.80	0.84	4.66E-03	up
LRRC25	ENSG0000017548	protein_coding	3.8995	2.2587	1.73	0.79	4.68E-03	up
KRT8	ENSG0000017042	retained_intron;pro	0.1930	0.0480	4.02	2.01	4.70E-03	up
TMEM147	ENSG0000010567	protein_coding;ret	33.2400	23.6488	1.41	0.49	4.70E-03	up
HNRNPA1P	ENSG0000021549	processed_pseudo;	19.4050	38.6943	0.50	-1.00	4.71E-03	down
WDR54	ENSG0000000544	protein_coding;ret	0.4772	1.0090	0.47	-1.08	4.72E-03	down
ARF6	ENSG0000016552	protein_coding	15.2586	20.7689	0.73	-0.44	4.73E-03	down
P2RY1	ENSG0000016986	protein_coding	0.5117	0.2286	2.24	1.16	4.74E-03	up
AIDA	ENSG0000018606	processed_transcri	2.0247	5.3021	0.38	-1.39	4.74E-03	down
ARHGAP33	ENSG0000000477	protein_coding;pro	1.1172	0.5831	1.92	0.94	4.75E-03	up
IPCEF1	ENSG0000007470	protein_coding;pro	2.7829	4.0241	0.69	-0.53	4.77E-03	down
YPEL3	ENSG0000009023	protein_coding;pro	0.6108	0.2672	2.29	1.19	4.79E-03	up
AHCYL2	ENSG0000015846	protein_coding;ret	1.5240	0.7303	2.09	1.06	4.83E-03	up
TSPAN17	ENSG0000004814	protein_coding;no	0.1842	0.4115	0.45	-1.16	4.84E-03	down
SKA2	ENSG0000018262	protein_coding;no	6.8294	10.0882	0.68	-0.56	4.85E-03	down
PODXL2	ENSG0000011463	protein_coding	31.7327	22.9563	1.38	0.47	4.85E-03	up

TRGJP1	ENSG0000021169	TR_J_gene	7.0338	17.0903	0.41	-1.28	4.90E-03	down
MCM8	ENSG0000012588	protein_coding	14.0778	10.4432	1.35	0.43	4.91E-03	up
CRNKL1	ENSG0000010134	protein_coding;ret	5.4527	7.8794	0.69	-0.53	4.92E-03	down
NPL	ENSG0000013583	protein_coding;pro	7.6125	5.2504	1.45	0.54	4.93E-03	up
TIMM8A	ENSG0000012695	protein_coding;no	5.8900	3.0593	1.93	0.95	4.93E-03	up
CENPE	ENSG0000013877	protein_coding;ret	4.1760	5.8043	0.72	-0.47	4.93E-03	down
FAM184A	ENSG0000011187	protein_coding;no	0.2595	0.1130	2.30	1.20	4.94E-03	up
MAFG	ENSG0000019706	protein_coding	5.5140	11.6086	0.47	-1.07	4.94E-03	down
TCP11L2	ENSG0000016604	protein_coding;no	0.3890	0.1664	2.34	1.22	4.97E-03	up
HSP90B1	ENSG0000016659	retained_intron;pro	205.3703	150.1819	1.37	0.45	4.98E-03	up
KCNJ15	ENSG0000015755	protein_coding;pro	0.1594	0.0647	2.46	1.30	4.98E-03	up
SEPT5	ENSG0000018470	protein_coding;ret	2.6509	0.8936	2.97	1.57	5.02E-03	up
EIF4A2	ENSG0000015697	protein_coding;ret	32.7552	44.4000	0.74	-0.44	5.05E-03	down
NIPA2	ENSG0000014015	protein_coding;pro	9.4134	15.5162	0.61	-0.72	5.06E-03	down
AC002350	ENSG0000027899	TEC	1.5236	0.7187	2.12	1.08	5.06E-03	up
RPL32P29	ENSG0000024427	processed_pseudo	17.4249	29.4429	0.59	-0.76	5.06E-03	down
SLC19A2	ENSG0000011747	protein_coding;ret	4.4705	8.7680	0.51	-0.97	5.06E-03	down
PHLDA1	ENSG0000013928	protein_coding	1.3231	0.7874	1.68	0.75	5.09E-03	up
PCK2	ENSG0000010088	protein_coding;ret	1.1384	2.0106	0.57	-0.82	5.13E-03	down
AC073896	ENSG0000014478	protein_coding	0.3584	2.5816	0.14	-2.85	5.13E-03	down
PRDX5	ENSG0000012643	protein_coding	207.1167	153.4771	1.35	0.43	5.14E-03	up
PON2	ENSG0000010585	protein_coding;pro	3.4116	2.0712	1.65	0.72	5.14E-03	up
AC079684	ENSG0000027494	sense_intronic	1.4610	3.5626	0.41	-1.29	5.15E-03	down
RARG	ENSG0000017281	processed_transcri	0.2135	0.0757	2.82	1.50	5.16E-03	up
CD74	ENSG0000001958	protein_coding;ret	6.6363	9.7352	0.68	-0.55	5.16E-03	down
CENPM	ENSG0000010016	protein_coding;ret	18.8255	12.5966	1.49	0.58	5.18E-03	up
NSRP1	ENSG0000012665	protein_coding;pro	4.6334	6.8740	0.67	-0.57	5.18E-03	down
SLC39A7	ENSG0000011247	processed_transcri	46.9875	34.6369	1.36	0.44	5.19E-03	up
ZNF618	ENSG0000015765	protein_coding;pro	0.0431	0.1214	0.36	-1.49	5.19E-03	down
FAM229B	ENSG0000020377	protein_coding	1.0375	0.4130	2.51	1.33	5.21E-03	up
IFRD2	ENSG0000021470	retained_intron;no	8.9092	12.4980	0.71	-0.49	5.22E-03	down
GUSB	ENSG0000016991	protein_coding;no	33.5861	24.4456	1.37	0.46	5.23E-03	up
AC093525	ENSG0000026161	antisense	1.0925	0.5070	2.15	1.11	5.25E-03	up
MATR3	ENSG0000028098	protein_coding	19.9171	30.9466	0.64	-0.64	5.26E-03	down
PTMA	ENSG0000018751	processed_transcri	517.6337	373.4743	1.39	0.47	5.28E-03	up
ICE2	ENSG0000012891	retained_intron;pro	1.8498	2.8142	0.66	-0.61	5.29E-03	down
FAM117B	ENSG0000013843	protein_coding;ret	0.6066	1.0623	0.57	-0.81	5.30E-03	down
MBNL1	ENSG0000015260	protein_coding;ret	24.4655	33.8414	0.72	-0.47	5.34E-03	down
TMEM87A	ENSG0000010397	protein_coding;ret	9.9025	13.3164	0.74	-0.43	5.36E-03	down
S100A11	ENSG0000016319	protein_coding;pro	339.9764	253.6510	1.34	0.42	5.37E-03	up
GRN	ENSG0000003058	retained_intron;pro	69.0630	50.8325	1.36	0.44	5.37E-03	up
DNAJC14	ENSG0000013539	protein_coding	7.4904	5.0222	1.49	0.58	5.39E-03	up
MYC	ENSG0000013699	protein_coding;pro	9.2778	4.3276	2.14	1.10	5.40E-03	up
ZNF236	ENSG0000013085	protein_coding;no	1.6741	1.0362	1.62	0.69	5.40E-03	up
MAN2B2	ENSG0000001328	protein_coding;pro	3.9364	2.5534	1.54	0.62	5.40E-03	up
MGAM2	ENSG0000025774	protein_coding;ret	0.3763	0.1817	2.07	1.05	5.42E-03	up
NOP53	ENSG0000010537	retained_intron;pro	29.1146	39.1270	0.74	-0.43	5.44E-03	down
AC092718	ENSG0000026106	sense_intronic	2.9436	5.5400	0.53	-0.91	5.45E-03	down

AL008721	ENSG0000027297	sense_intronic	1.1792	0.6081	1.94	0.96	5.49E-03	up
PSMD12	ENSG0000019717	retained_intron;pr	12.7417	17.0863	0.75	-0.42	5.49E-03	down
CDC42EP3	ENSG0000016317	protein_coding;ret	4.1397	6.2567	0.66	-0.60	5.52E-03	down
EHD4	ENSG0000010396	protein_coding;ret	0.6602	0.3586	1.84	0.88	5.53E-03	up
ME1	ENSG0000006583	protein_coding	0.6083	1.1567	0.53	-0.93	5.54E-03	down
SNX16	ENSG0000010449	protein_coding;ret	1.3114	2.2162	0.59	-0.76	5.55E-03	down
ZNF652	ENSG0000019874	protein_coding;no	1.6010	0.8801	1.82	0.86	5.56E-03	up
LRP1	ENSG0000012338	protein_coding;ret	0.3000	0.1521	1.97	0.98	5.56E-03	up
ACADM	ENSG0000011705	protein_coding;ret	14.6832	19.8530	0.74	-0.44	5.57E-03	down
FAM206A	ENSG0000011932	protein_coding;pr	11.5273	16.1856	0.71	-0.49	5.60E-03	down
METTL4	ENSG0000010157	protein_coding;ret	3.4823	5.6232	0.62	-0.69	5.60E-03	down
ADGRE1	ENSG0000017483	protein_coding;pr	5.1594	3.3261	1.55	0.63	5.61E-03	up
CCT4	ENSG0000011548	protein_coding;pr	89.7494	119.2071	0.75	-0.41	5.63E-03	down
PRDM5	ENSG0000013873	protein_coding;pr	0.8569	0.4784	1.79	0.84	5.66E-03	up
DPH5	ENSG0000011754	processed_transcri	7.7657	11.5352	0.67	-0.57	5.68E-03	down
ASB16-AS1	ENSG0000026708	antisense	1.7573	0.8159	2.15	1.11	5.69E-03	up
HAX1	ENSG0000014357	retained_intron;pr	24.6714	33.2469	0.74	-0.43	5.70E-03	down
CACNA2D3	ENSG0000015744	protein_coding;ret	1.5976	2.5844	0.62	-0.69	5.72E-03	down
RFWD3	ENSG0000016841	protein_coding;ret	12.8047	17.0535	0.75	-0.41	5.72E-03	down
JUN	ENSG0000017760	protein_coding	6.0574	9.2075	0.66	-0.60	5.73E-03	down
RPL9P7	ENSG0000023810	processed_pseudo	351.2708	489.8172	0.72	-0.48	5.75E-03	down
CCT6B	ENSG0000013214	retained_intron;pr	0.1623	0.3879	0.42	-1.26	5.78E-03	down
UTRN	ENSG0000015281	protein_coding;pr	1.9618	2.7897	0.70	-0.51	5.81E-03	down
TRAK2	ENSG0000011599	protein_coding;ret	5.2910	3.7681	1.40	0.49	5.86E-03	up
HHEX	ENSG0000015280	protein_coding;ret	14.9960	9.6477	1.55	0.64	5.88E-03	up
JAK1	ENSG0000016243	protein_coding;pr	15.6528	11.7136	1.34	0.42	5.92E-03	up
ADK	ENSG0000015611	protein_coding;pr	13.6527	18.9301	0.72	-0.47	5.94E-03	down
ANKRD23	ENSG0000016312	protein_coding;pr	0.2848	0.1009	2.82	1.50	5.96E-03	up
CCDC137	ENSG0000018529	nonsense_mediate	13.6548	6.6252	2.06	1.04	6.02E-03	up
BNIP2	ENSG0000014029	protein_coding;ret	8.9157	11.9351	0.75	-0.42	6.03E-03	down
NDUFAF4	ENSG0000012354	processed_transcri	6.6022	9.9204	0.67	-0.59	6.06E-03	down
WBP1	ENSG0000023977	processed_transcri	10.0896	6.2904	1.60	0.68	6.07E-03	up
SEMA4A	ENSG0000019618	protein_coding;pr	3.9398	5.6070	0.70	-0.51	6.10E-03	down
KIAA0319L	ENSG0000014268	protein_coding;pr	4.4464	3.0904	1.44	0.52	6.12E-03	up
ADGRG3	ENSG0000018288	nonsense_mediate	3.8726	2.4097	1.61	0.68	6.14E-03	up
MAN1A2	ENSG0000019816	protein_coding;pr	3.5554	2.4215	1.47	0.55	6.15E-03	up
GABPB1-A!	ENSG0000024487	antisense	0.7598	1.2021	0.63	-0.66	6.15E-03	down
HELB	ENSG0000012731	protein_coding;no	0.8554	0.3733	2.29	1.20	6.20E-03	up
RASGRF1	ENSG0000005833	protein_coding;pr	0.1551	0.0554	2.80	1.48	6.20E-03	up
NRG4	ENSG0000016975	nonsense_mediate	0.0970	0.2401	0.40	-1.31	6.21E-03	down
TBRG1	ENSG0000015414	nonsense_mediate	8.7190	5.9042	1.48	0.56	6.21E-03	up
SLC11A1	ENSG0000001828	retained_intron;pr	0.7197	0.3896	1.85	0.89	6.23E-03	up
AP1G2	ENSG0000021398	protein_coding;ret	5.8866	4.2120	1.40	0.48	6.23E-03	up
RAB11FIP4	ENSG0000013124	protein_coding;pr	0.4209	0.2401	1.75	0.81	6.26E-03	up
SLC35B3	ENSG0000012478	protein_coding;no	4.7687	7.3194	0.65	-0.62	6.27E-03	down
WWC2	ENSG0000015171	protein_coding;ret	0.0824	0.1853	0.44	-1.17	6.28E-03	down
PFN1	ENSG0000010851	protein_coding	335.5298	245.9255	1.36	0.45	6.31E-03	up
RNASET2	ENSG0000002629	nonsense_mediate	18.6313	13.7009	1.36	0.44	6.36E-03	up

AC011815	ENSG0000026857	lincRNA	3.1882	1.9808	1.61	0.69	6.37E-03	up
DUSP18	ENSG0000016706	protein_coding;no	0.3437	0.1499	2.29	1.20	6.38E-03	up
HAL	ENSG0000008411	protein_coding;no	3.3948	5.0819	0.67	-0.58	6.45E-03	down
KRT18	ENSG0000011105	protein_coding;ret	0.7574	0.2678	2.83	1.50	6.45E-03	up
RNF145	ENSG0000014586	processed_transcri	5.1554	8.0612	0.64	-0.64	6.49E-03	down
PHACTR2	ENSG0000011241	protein_coding;ret	0.8801	0.3572	2.46	1.30	6.49E-03	up
GNPTAB	ENSG0000011167	protein_coding;pro	4.1072	2.9359	1.40	0.48	6.53E-03	up
CASP2	ENSG0000010614	protein_coding;ret	6.7840	10.5595	0.64	-0.64	6.54E-03	down
ZNF404	ENSG0000017622	protein_coding;pro	2.9146	1.6894	1.73	0.79	6.55E-03	up
LRRC4	ENSG0000012859	protein_coding	0.2799	0.1157	2.42	1.27	6.57E-03	up
NOTCH1	ENSG0000014840	retained_intron;pro	1.3375	0.8257	1.62	0.70	6.58E-03	up
COLQ	ENSG0000020656	protein_coding;ret	0.3458	0.7409	0.47	-1.10	6.60E-03	down
SLC25A11	ENSG0000010852	protein_coding;ret	13.5076	9.0118	1.50	0.58	6.60E-03	up
HIST2H2AA	ENSG0000020381	protein_coding	5.3019	2.5591	2.07	1.05	6.62E-03	up
BSCL2	ENSG0000016800	protein_coding;pro	7.7809	5.1850	1.50	0.59	6.65E-03	up
CCDC91	ENSG0000012310	protein_coding;no	2.5443	3.7593	0.68	-0.56	6.70E-03	down
RPL23	ENSG0000012569	protein_coding;ret	166.8114	221.1984	0.75	-0.41	6.71E-03	down
AC002116	ENSG0000026769	antisense	2.6824	1.2212	2.20	1.14	6.72E-03	up
OXA1L	ENSG0000015546	retained_intron;pro	49.0334	65.6848	0.75	-0.42	6.73E-03	down
VAMP1	ENSG0000013919	protein_coding;ret	10.5084	6.9889	1.50	0.59	6.74E-03	up
ATOX1	ENSG0000017755	protein_coding;ret	9.5375	6.8925	1.38	0.47	6.75E-03	up
SPECC1L	ENSG0000010001	protein_coding;ret	7.1918	4.5636	1.58	0.66	6.75E-03	up
MAPK13	ENSG0000015671	protein_coding;pro	0.7331	0.3960	1.85	0.89	6.76E-03	up
ABCA7	ENSG0000006468	protein_coding;ret	0.9353	0.5413	1.73	0.79	6.77E-03	up
ZUP1	ENSG0000015397	processed_transcri	8.3539	12.0979	0.69	-0.53	6.79E-03	down
TNIK	ENSG0000015431	protein_coding;ret	0.0836	0.0338	2.47	1.31	6.80E-03	up
THADA	ENSG0000011597	protein_coding;ret	2.2774	3.1786	0.72	-0.48	6.81E-03	down
ZNF821	ENSG0000010298	protein_coding;pro	0.2309	0.5529	0.42	-1.26	6.82E-03	down
MAN2B1	ENSG0000010477	retained_intron;pro	34.5499	25.3265	1.36	0.45	6.84E-03	up
PCBP1-AS1	ENSG0000017981	antisense;lincRNA	0.4584	0.7110	0.64	-0.63	6.84E-03	down
EXOSC8	ENSG0000012069	nonsense_mediate	17.6078	23.8131	0.74	-0.44	6.85E-03	down
NDUFA4L2	ENSG0000018563	retained_intron;pro	0.2091	0.4705	0.44	-1.17	6.86E-03	down
FAM131A	ENSG0000017518	protein_coding;pro	0.1317	0.0550	2.40	1.26	6.91E-03	up
AC239798	ENSG0000023457	lincRNA	0.5929	1.1191	0.53	-0.92	6.94E-03	down
PANX1	ENSG0000011021	protein_coding	4.1378	2.5138	1.65	0.72	6.97E-03	up
AC127459	ENSG0000026056	3prime_overlappin	0.6255	1.5347	0.41	-1.29	6.99E-03	down
SUGT1P4-S	ENSG0000025487	processed_transcri	0.3021	0.6301	0.48	-1.06	7.01E-03	down
PYROXD1	ENSG0000012135	protein_coding;no	7.7246	10.9986	0.70	-0.51	7.05E-03	down
PRAM1	ENSG0000013324	protein_coding;ret	33.8532	25.0346	1.35	0.44	7.08E-03	up
RENBP	ENSG0000010203	protein_coding;ret	4.1106	2.3411	1.76	0.81	7.08E-03	up
PDIA3	ENSG0000016700	protein_coding;ret	83.1592	61.8619	1.34	0.43	7.08E-03	up
MBOAT7	ENSG0000012550	protein_coding;ret	8.2193	5.8302	1.41	0.50	7.14E-03	up
NAT1	ENSG0000017142	protein_coding;pro	1.8655	3.3829	0.55	-0.86	7.18E-03	down
ERI2	ENSG0000019667	retained_intron;pro	4.1620	5.8917	0.71	-0.50	7.20E-03	down
HNRNPDL	ENSG0000015279	protein_coding;no	44.9724	59.2495	0.76	-0.40	7.21E-03	down
INTS2	ENSG0000010850	protein_coding;ret	2.7378	3.8987	0.70	-0.51	7.22E-03	down
TNFSF9	ENSG0000012565	protein_coding	0.6194	1.3147	0.47	-1.09	7.24E-03	down
IL1B	ENSG0000012553	protein_coding;ret	4.3436	2.5239	1.72	0.78	7.28E-03	up

RARS	ENSG0000011364	retained_intron;pr	18.5471	24.9446	0.74	-0.43	7.28E-03	down
VTA1	ENSG0000000984	protein_coding;pr	9.9861	13.2467	0.75	-0.41	7.30E-03	down
ZNF569	ENSG0000019643	protein_coding;pr	2.0025	3.1470	0.64	-0.65	7.34E-03	down
USP11	ENSG0000010222	protein_coding;ret	2.1892	1.3546	1.62	0.69	7.35E-03	up
HAND2	ENSG0000016410	protein_coding;pr	0.2217	0.4901	0.45	-1.14	7.39E-03	down
LDLRAD4	ENSG0000016867	retained_intron;pr	0.1992	0.3607	0.55	-0.86	7.40E-03	down
AIMP2	ENSG0000010630	protein_coding;no	13.5415	19.3035	0.70	-0.51	7.43E-03	down
PARVB	ENSG0000018867	protein_coding;pr	5.8122	4.2223	1.38	0.46	7.44E-03	up
MTA3	ENSG0000005793	protein_coding;pr	0.4194	0.2303	1.82	0.86	7.49E-03	up
MID1IP1	ENSG0000016517	protein_coding	11.7457	16.7965	0.70	-0.52	7.52E-03	down
ARRB2	ENSG0000014148	protein_coding;no	8.5161	5.8775	1.45	0.53	7.53E-03	up
CKLF-CMT	ENSG0000025478	nonsense_mediate	0.4199	0.0534	7.86	2.97	7.54E-03	up
CMTM3	ENSG0000014093	retained_intron;pr	13.3677	9.0039	1.48	0.57	7.55E-03	up
CDK17	ENSG0000005975	protein_coding;ret	2.0846	3.1737	0.66	-0.61	7.56E-03	down
CD9	ENSG0000001027	protein_coding;ret	0.7648	0.3573	2.14	1.10	7.57E-03	up
CPNE3	ENSG0000008571	protein_coding;ret	1.6492	2.5503	0.65	-0.63	7.58E-03	down
UBXN8	ENSG0000010469	processed_transcri	4.1364	6.4331	0.64	-0.64	7.59E-03	down
FBXO41	ENSG0000016301	protein_coding;ret	1.5277	3.5664	0.43	-1.22	7.60E-03	down
KANTR	ENSG0000023259	processed_transcri	0.8017	0.4535	1.77	0.82	7.66E-03	up
CD79A	ENSG0000010536	protein_coding	0.9790	0.4049	2.42	1.27	7.66E-03	up
RAB5B	ENSG0000011154	protein_coding;no	15.4002	24.8414	0.62	-0.69	7.67E-03	down
YTHDF3	ENSG0000018572	protein_coding;no	7.7068	4.3444	1.77	0.83	7.69E-03	up
HSPA5	ENSG0000004457	protein_coding	193.4971	146.0045	1.33	0.41	7.73E-03	up
EFNA4	ENSG0000024336	protein_coding	0.7965	0.3004	2.65	1.41	7.76E-03	up
CLIC4P1	ENSG0000023673	processed_pseudo	1.1276	2.8341	0.40	-1.33	7.82E-03	down
GNG7	ENSG0000017653	protein_coding;no	0.3202	0.9718	0.33	-1.60	7.85E-03	down
PEX12	ENSG0000010873	protein_coding;no	1.2379	2.1424	0.58	-0.79	7.87E-03	down
PPP1CC	ENSG0000018629	protein_coding;ret	22.4242	29.7858	0.75	-0.41	7.87E-03	down
DOCK11	ENSG0000014725	protein_coding	13.2157	17.4120	0.76	-0.40	7.96E-03	down
PVT1	ENSG0000024985	lincRNA;misc_RN	0.8901	1.4286	0.62	-0.68	7.96E-03	down
DLG5	ENSG0000015120	protein_coding;pr	0.1155	0.0486	2.37	1.25	7.98E-03	up
TMEM173	ENSG0000018458	protein_coding;ret	1.7194	2.7110	0.63	-0.66	8.01E-03	down
UBE3A	ENSG0000011406	protein_coding;pr	2.9308	4.1696	0.70	-0.51	8.01E-03	down
JAZF1	ENSG0000015381	protein_coding;no	2.7824	4.2400	0.66	-0.61	8.02E-03	down
ANXA1	ENSG0000013504	protein_coding;pr	60.3215	45.3569	1.33	0.41	8.03E-03	up
SAR1A	ENSG0000007933	protein_coding;pr	12.9067	18.9620	0.68	-0.55	8.11E-03	down
YKT6	ENSG0000010663	retained_intron;pr	12.4796	7.7610	1.61	0.69	8.12E-03	up
METTL16	ENSG0000012780	protein_coding;pr	0.8137	1.4654	0.56	-0.85	8.13E-03	down
HCAR2	ENSG0000018278	protein_coding	1.2803	0.6359	2.01	1.01	8.16E-03	up
FEZ1	ENSG0000014955	retained_intron;pr	0.1858	0.0797	2.33	1.22	8.18E-03	up
CCNB2	ENSG0000015745	protein_coding;ret	13.9910	19.3584	0.72	-0.47	8.18E-03	down
HMOX1	ENSG0000010029	protein_coding;ret	0.8127	0.3722	2.18	1.13	8.18E-03	up
SENP8	ENSG0000016619	protein_coding	0.4977	0.8965	0.56	-0.85	8.21E-03	down
SAE1	ENSG0000014223	nonsense_mediate	46.4350	34.9649	1.33	0.41	8.25E-03	up
FBXO21	ENSG0000013510	protein_coding;pr	1.4471	0.8877	1.63	0.71	8.29E-03	up
FSBP	ENSG0000026581	protein_coding;no	2.0764	1.0704	1.94	0.96	8.32E-03	up
DDX19B	ENSG0000015734	protein_coding;no	9.2989	12.4474	0.75	-0.42	8.33E-03	down
RPL22P1	ENSG0000021317	processed_pseudo	238.1576	316.4621	0.75	-0.41	8.40E-03	down

MRPL45	ENSG0000027884	protein_coding	36.7533	48.5474	0.76	-0.40	8.40E-03	down
DPM3	ENSG0000017908	protein_coding	13.5424	8.1105	1.67	0.74	8.42E-03	up
WASH5P	ENSG0000028245	retained_intron;pr	0.5753	0.1505	3.82	1.93	8.45E-03	up
IKZF2	ENSG0000003041	protein_coding;ret	0.6728	0.3591	1.87	0.91	8.48E-03	up
TMEM33	ENSG0000010913	protein_coding;no	2.7325	4.3170	0.63	-0.66	8.52E-03	down
DENR	ENSG0000013972	protein_coding;ret	11.4193	15.4331	0.74	-0.43	8.53E-03	down
PRG3	ENSG0000015657	protein_coding	5.6276	3.0573	1.84	0.88	8.60E-03	up
GALC	ENSG0000005498	retained_intron;pr	6.9162	5.0442	1.37	0.46	8.60E-03	up
HS6ST1	ENSG0000013672	protein_coding;pr	5.3073	3.5907	1.48	0.56	8.61E-03	up
SUPT3H	ENSG0000019628	protein_coding;pr	2.0330	3.1335	0.65	-0.62	8.63E-03	down
AC243772	ENSG0000023303	antisense	0.3396	0.6797	0.50	-1.00	8.63E-03	down
POLR1D	ENSG0000018618	protein_coding;no	12.1868	16.1049	0.76	-0.40	8.65E-03	down
RABGAP1	ENSG0000001145	protein_coding;no	4.2269	3.0405	1.39	0.48	8.71E-03	up
AP003486	ENSG0000025545	lincRNA	0.6261	0.2912	2.15	1.10	8.71E-03	up
ARHGEF34	ENSG0000020495	unprocessed_pseu	0.2970	0.1223	2.43	1.28	8.79E-03	up
CLEC4A	ENSG0000011172	protein_coding	3.5941	1.8853	1.91	0.93	8.80E-03	up
PAK1	ENSG0000014926	protein_coding;ret	11.0274	7.5224	1.47	0.55	8.81E-03	up
CCT8	ENSG0000015626	retained_intron;pr	87.4463	114.5450	0.76	-0.39	8.82E-03	down
SNHG6	ENSG0000024591	lincRNA;retained_	115.2918	151.3508	0.76	-0.39	8.84E-03	down
AF233439	ENSG0000024585	lincRNA	0.8342	0.3541	2.36	1.24	8.89E-03	up
ZNF280D	ENSG0000013787	protein_coding;pr	1.7457	2.5424	0.69	-0.54	8.93E-03	down
RPS3A	ENSG0000014542	protein_coding;ret	723.6071	983.9039	0.74	-0.44	8.93E-03	down
CERK	ENSG0000010042	protein_coding;pr	8.0750	10.8988	0.74	-0.43	9.03E-03	down
CD4	ENSG0000001061	protein_coding;ret	43.6016	32.9549	1.32	0.40	9.05E-03	up
MDH2	ENSG0000014670	protein_coding;ret	75.7814	57.4596	1.32	0.40	9.06E-03	up
RUNX2	ENSG0000012481	protein_coding;no	0.8089	2.0104	0.40	-1.31	9.07E-03	down
AL160408	ENSG0000023062	lincRNA	1.1127	0.5916	1.88	0.91	9.09E-03	up
AATK	ENSG0000018140	protein_coding;no	0.1163	0.0304	3.82	1.93	9.10E-03	up
RPL21P119	ENSG0000022079	processed_pseudo	20.8817	32.6751	0.64	-0.65	9.16E-03	down
CYB5R3	ENSG0000010024	protein_coding;ret	5.7437	3.5820	1.60	0.68	9.21E-03	up
RNF8	ENSG0000011213	processed_transcri	2.7000	4.1497	0.65	-0.62	9.24E-03	down
C4orf3	ENSG0000016409	protein_coding	24.6004	18.4894	1.33	0.41	9.26E-03	up
PLEKHA8	ENSG0000010608	protein_coding;no	1.6132	0.8230	1.96	0.97	9.26E-03	up
NOL11	ENSG0000013093	retained_intron;pr	14.6044	19.2796	0.76	-0.40	9.28E-03	down
ABHD10	ENSG0000014482	protein_coding;no	18.3808	11.9719	1.54	0.62	9.32E-03	up
ERGIC3	ENSG0000012599	nonsense_mediate	30.7856	23.3208	1.32	0.40	9.37E-03	up
RAB32	ENSG0000011850	protein_coding	53.2275	70.2222	0.76	-0.40	9.42E-03	down
SDF4	ENSG0000007880	protein_coding;no	20.4654	15.1931	1.35	0.43	9.43E-03	up
BLOC1S5	ENSG0000018842	protein_coding;no	9.1873	6.1428	1.50	0.58	9.49E-03	up
MAGED1	ENSG0000017922	protein_coding;pr	5.4914	3.8482	1.43	0.51	9.54E-03	up
ANKRD12	ENSG0000010174	protein_coding;pr	4.4904	2.8942	1.55	0.63	9.64E-03	up
HS1BP3	ENSG0000011896	nonsense_mediate	0.7532	0.4672	1.61	0.69	9.64E-03	up
CETN2	ENSG0000014740	protein_coding;pr	17.3129	11.7083	1.48	0.56	9.64E-03	up
GALNT4	ENSG0000025759	protein_coding	1.0267	1.9367	0.53	-0.92	9.66E-03	down
ANXA3	ENSG0000013877	protein_coding;ret	4.3751	6.3582	0.69	-0.54	9.67E-03	down
TATDN3	ENSG0000020370	protein_coding;no	2.8665	4.5496	0.63	-0.67	9.68E-03	down
NDUFB10	ENSG0000014099	protein_coding;ret	94.9001	71.3096	1.33	0.41	9.69E-03	up
F8A3	ENSG0000027715	protein_coding	4.6796	2.1726	2.15	1.11	9.72E-03	up

AC005674	ENSG0000026149	sense_overlapping	0.8396	0.4321	1.94	0.96	9.74E-03	up
FCGR2B	ENSG0000007269	protein_coding;ret	0.7767	0.4265	1.82	0.86	9.75E-03	up
SMIM25	ENSG0000022439	lincRNA	1.1294	2.0136	0.56	-0.83	9.75E-03	down
AL034397	ENSG0000027453	antisense	2.5776	1.4409	1.79	0.84	9.85E-03	up
ZBTB49	ENSG0000016882	protein_coding;no	0.9797	1.8080	0.54	-0.88	9.85E-03	down
CDIPT	ENSG0000010350	protein_coding;pro	5.6697	8.5507	0.66	-0.59	9.86E-03	down
CYB561D1	ENSG0000017415	protein_coding	2.1535	1.3330	1.62	0.69	9.89E-03	up
TFB2M	ENSG0000016285	protein_coding	14.8565	20.4554	0.73	-0.46	9.92E-03	down
ACAD11	ENSG0000024030	protein_coding;ret	3.9898	5.7261	0.70	-0.52	9.93E-03	down
AFG1L	ENSG0000013553	protein_coding;pro	1.1810	1.8358	0.64	-0.64	9.94E-03	down
STAG3	ENSG0000006692	protein_coding;ret	7.7386	5.8216	1.33	0.41	9.95E-03	up
MINDY2	ENSG0000012892	protein_coding;no	0.4968	1.1488	0.43	-1.21	9.97E-03	down
ZFAND1	ENSG0000010423	protein_coding;no	11.3645	16.9947	0.67	-0.58	9.97E-03	down
STS	ENSG0000010184	protein_coding	0.4103	0.2056	2.00	1.00	9.98E-03	up
GALNT11	ENSG0000017823	nonsense_mediate	7.1545	5.3316	1.34	0.42	9.99E-03	up
CCDC106	ENSG0000017358	protein_coding;ret	4.4682	2.7749	1.61	0.69	1.00E-02	up
GABARAPI	ENSG0000013911	nonsense_mediate	9.0400	4.5712	1.98	0.98	1.00E-02	up
TCF12	ENSG0000014026	processed_transcri	11.2666	15.1437	0.74	-0.43	1.00E-02	down
CD68	ENSG0000012922	protein_coding	16.9102	12.1530	1.39	0.48	1.01E-02	up
TECR	ENSG0000009979	nonsense_mediate	9.8774	7.1650	1.38	0.46	1.01E-02	up
AC112220	ENSG0000027164	bidirectional_pron	0.4066	0.1974	2.06	1.04	1.01E-02	up
TRAPPC1	ENSG0000017004	protein_coding;no	34.1891	23.0573	1.48	0.57	1.01E-02	up
SLC25A12	ENSG0000011584	retained_intron;pro	3.6998	5.4288	0.68	-0.55	1.01E-02	down
SAMD13	ENSG0000020394	protein_coding;ret	0.6826	1.2453	0.55	-0.87	1.01E-02	down
SMG9	ENSG0000010577	retained_intron;pro	2.4973	3.6352	0.69	-0.54	1.01E-02	down
MTCP1	ENSG0000021482	protein_coding;pro	0.5258	1.1508	0.46	-1.13	1.02E-02	down
ARNTL2	ENSG0000002915	protein_coding;pro	3.4816	1.9328	1.80	0.85	1.02E-02	up
CCDC102B	ENSG0000015063	processed_transcri	0.6486	0.3306	1.96	0.97	1.02E-02	up
DENND2D	ENSG0000016277	processed_transcri	2.0045	3.0542	0.66	-0.61	1.02E-02	down
PARD6A	ENSG0000010298	protein_coding;ret	1.1108	0.4906	2.26	1.18	1.02E-02	up
STRA6LP	ENSG0000025503	processed_transcri	0.8141	0.4733	1.72	0.78	1.03E-02	up
LRFN4	ENSG0000017362	protein_coding;pro	3.0419	5.2432	0.58	-0.79	1.03E-02	down
CFLAR	ENSG0000000340	protein_coding;ret	1.7077	1.1455	1.49	0.58	1.04E-02	up
BTN3A3	ENSG0000011180	protein_coding;ret	0.8273	1.4569	0.57	-0.82	1.04E-02	down
OR2A20P	ENSG0000017035	processed_transcri	0.3077	0.1157	2.66	1.41	1.04E-02	up
BTBD9	ENSG0000018382	protein_coding;no	0.6951	0.3793	1.83	0.87	1.04E-02	up
ATP5IF1	ENSG0000013077	protein_coding	27.7230	20.8683	1.33	0.41	1.05E-02	up
CAMK2D	ENSG0000014534	protein_coding;ret	0.3559	0.6235	0.57	-0.81	1.05E-02	down
MGAT4A	ENSG0000007107	protein_coding;pro	4.3900	6.0952	0.72	-0.47	1.05E-02	down
KLHL35	ENSG0000014924	retained_intron;pro	1.2391	0.7016	1.77	0.82	1.05E-02	up
HACD3	ENSG0000007469	protein_coding;pro	7.5740	13.4174	0.56	-0.82	1.05E-02	down
IFNGR2	ENSG0000015912	protein_coding;no	25.2012	18.8400	1.34	0.42	1.05E-02	up
RGCC	ENSG0000010276	protein_coding;pro	16.2992	24.1041	0.68	-0.56	1.06E-02	down
ZNRF2	ENSG0000018023	protein_coding;pro	4.6200	6.8065	0.68	-0.56	1.06E-02	down
AP3S2	ENSG0000015782	protein_coding;no	1.7591	2.5713	0.68	-0.55	1.06E-02	down
LASP1	ENSG0000000283	protein_coding;ret	24.0490	18.0010	1.34	0.42	1.06E-02	up
DEPDC1	ENSG0000002452	protein_coding;ret	3.5911	5.4015	0.66	-0.59	1.07E-02	down
PQLC2	ENSG0000004048	nonsense_mediate	1.4989	2.5956	0.58	-0.79	1.07E-02	down

NDUFA12	ENSG0000018475	protein_coding;ret	24.5955	15.3367	1.60	0.68	1.07E-02	up
SMAD7	ENSG0000010166	retained_intron;pr	1.1105	1.8131	0.61	-0.71	1.07E-02	down
EIF2S2	ENSG0000012597	protein_coding	82.1274	106.8948	0.77	-0.38	1.07E-02	down
FGF14-AS2	ENSG0000027214	lincRNA	1.1208	0.4703	2.38	1.25	1.07E-02	up
GHRLOS	ENSG0000024028	antisense	0.2510	0.6357	0.39	-1.34	1.07E-02	down
GLIPR1	ENSG0000013927	protein_coding;no	4.1872	2.8446	1.47	0.56	1.08E-02	up
ATG16L2	ENSG0000016801	retained_intron;pr	11.3695	8.6069	1.32	0.40	1.08E-02	up
GDI2	ENSG0000005760	protein_coding;pr	41.9599	55.0661	0.76	-0.39	1.08E-02	down
ZNF805	ENSG0000020452	protein_coding	0.0403	0.2262	0.18	-2.49	1.08E-02	down
FGGY	ENSG0000017245	protein_coding;pr	0.2498	0.4321	0.58	-0.79	1.08E-02	down
DYNLL2	ENSG0000026436	protein_coding	13.9116	10.5514	1.32	0.40	1.08E-02	up
AC012254	ENSG0000026722	nonsense_mediate	0.0492	0.5121	0.10	-3.38	1.08E-02	down
ZNF471	ENSG0000019626	protein_coding;pr	0.7624	0.3767	2.02	1.02	1.09E-02	up
RAB12	ENSG0000020641	protein_coding;ret	4.8951	7.2870	0.67	-0.57	1.09E-02	down
IDI1	ENSG0000006706	protein_coding;ret	12.6098	9.2133	1.37	0.45	1.10E-02	up
PILRA	ENSG0000008551	protein_coding;ret	9.9255	6.2723	1.58	0.66	1.11E-02	up
CRTC1	ENSG0000010566	protein_coding	0.8470	0.4890	1.73	0.79	1.11E-02	up
LRG1	ENSG0000017123	protein_coding;pr	21.5335	15.4254	1.40	0.48	1.11E-02	up
AP2B1	ENSG0000000612	protein_coding;no	28.2131	20.2232	1.40	0.48	1.11E-02	up
HELZ2	ENSG0000013058	protein_coding;pr	0.1410	0.0626	2.25	1.17	1.11E-02	up
NAB2	ENSG0000016688	protein_coding;pr	5.3171	3.4259	1.55	0.63	1.11E-02	up
UTP4	ENSG0000014107	protein_coding;ret	13.6625	17.6728	0.77	-0.37	1.11E-02	down
TSTD1	ENSG0000021584	retained_intron;pr	8.2511	4.9641	1.66	0.73	1.13E-02	up
IL10RB-DT	ENSG0000022379	antisense	2.6326	1.4420	1.83	0.87	1.13E-02	up
NDE1	ENSG0000007286	nonsense_mediate	2.3909	3.6193	0.66	-0.60	1.13E-02	down
CENPK	ENSG0000012321	protein_coding;no	2.9545	4.4163	0.67	-0.58	1.13E-02	down
CROT	ENSG0000000546	protein_coding;ret	0.5382	0.2606	2.07	1.05	1.13E-02	up
BTF3	ENSG0000014574	protein_coding;ret	135.1379	175.3235	0.77	-0.38	1.13E-02	down
TNFAIP8L3	ENSG0000018357	protein_coding	0.2332	0.5027	0.46	-1.11	1.13E-02	down
G6PD	ENSG0000016021	protein_coding;ret	12.7642	9.0069	1.42	0.50	1.13E-02	up
NSMAF	ENSG0000003568	processed_transcri	12.7530	16.5567	0.77	-0.38	1.13E-02	down
APOO	ENSG0000018483	protein_coding;ret	2.7336	4.3422	0.63	-0.67	1.14E-02	down
NUDT13	ENSG0000016632	protein_coding;pr	1.2572	2.1093	0.60	-0.75	1.14E-02	down
EPOP	ENSG0000027360	protein_coding	0.7601	0.3835	1.98	0.99	1.15E-02	up
CCDC124	ENSG0000000708	protein_coding;ret	30.7128	22.1860	1.38	0.47	1.15E-02	up
S100PBP	ENSG0000011649	protein_coding;pr	3.5056	1.8779	1.87	0.90	1.15E-02	up
ZNF10	ENSG0000025622	protein_coding;ret	1.7866	0.9103	1.96	0.97	1.15E-02	up
ARHGAP10	ENSG0000007120	protein_coding;ret	0.6350	0.3681	1.73	0.79	1.15E-02	up
ZNF267	ENSG0000018594	protein_coding;ret	3.6521	6.0671	0.60	-0.73	1.15E-02	down
NEXN	ENSG0000016261	protein_coding;pr	0.1197	0.2648	0.45	-1.15	1.15E-02	down
EFR3A	ENSG0000013229	retained_intron;pr	5.6030	8.1356	0.69	-0.54	1.16E-02	down
RHBDD2	ENSG0000000548	nonsense_mediate	2.2328	1.2380	1.80	0.85	1.16E-02	up
AL359532	ENSG0000027291	sense_intronic	0.7342	1.5058	0.49	-1.04	1.16E-02	down
HIKESHI	ENSG0000014919	nonsense_mediate	9.1789	6.1380	1.50	0.58	1.17E-02	up
CDKN1B	ENSG0000011127	protein_coding;pr	11.8716	16.0522	0.74	-0.44	1.17E-02	down
AC022400	ENSG0000027908	TEC	11.1103	16.4247	0.68	-0.56	1.18E-02	down
HSPE1P2	ENSG0000025864	processed_pseudo	13.9292	7.8356	1.78	0.83	1.18E-02	up
SMIM14	ENSG0000016368	protein_coding;no	1.2404	0.7360	1.69	0.75	1.18E-02	up

RCN1	ENSG0000004944	protein_coding;ret	7.2721	5.1877	1.40	0.49	1.18E-02	up
MTO1	ENSG0000013529	protein_coding;no	3.2605	4.4409	0.73	-0.45	1.18E-02	down
CD53	ENSG0000014311	processed_transcri	42.5626	32.4319	1.31	0.39	1.18E-02	up
ANPEP	ENSG0000016682	protein_coding;pro	22.9715	16.9875	1.35	0.44	1.18E-02	up
EEF1B2P3	ENSG0000023247	processed_pseudo;	77.9469	102.4637	0.76	-0.39	1.19E-02	down
IFT57	ENSG0000011444	protein_coding;pro	37.3623	48.2599	0.77	-0.37	1.19E-02	down
OSBPL11	ENSG0000014490	protein_coding	2.9854	1.9549	1.53	0.61	1.19E-02	up
ITGB2-AS1	ENSG0000022703	antisense	3.6004	1.1354	3.17	1.66	1.19E-02	up
CERCAM	ENSG0000016712	retained_intron;pro	0.5613	0.2986	1.88	0.91	1.19E-02	up
MEPCE	ENSG0000014683	protein_coding;ret	12.6937	9.1180	1.39	0.48	1.19E-02	up
RNF121	ENSG0000013752	protein_coding;ret	5.3639	3.3304	1.61	0.69	1.19E-02	up
NREP	ENSG0000013498	protein_coding;pro	7.4478	5.3584	1.39	0.48	1.21E-02	up
MMS22L	ENSG0000014626	retained_intron;pro	1.9238	2.5417	0.76	-0.40	1.21E-02	down
CEACAM8	ENSG0000012446	protein_coding	11.7310	16.2730	0.72	-0.47	1.21E-02	down
PBRM1	ENSG0000016393	protein_coding;ret	4.9375	6.5912	0.75	-0.42	1.22E-02	down
RAB3A	ENSG0000010564	retained_intron;pro	0.4871	0.1906	2.56	1.35	1.22E-02	up
PCDHGA8	ENSG0000025376	protein_coding	0.0435	0.1024	0.42	-1.24	1.23E-02	down
IL2RG	ENSG0000014716	protein_coding;ret	30.5627	23.1348	1.32	0.40	1.23E-02	up
RNF41	ENSG0000018185	retained_intron;pro	7.6123	10.3255	0.74	-0.44	1.24E-02	down
SRA1	ENSG0000021352	retained_intron;pro	7.9105	5.4043	1.46	0.55	1.24E-02	up
UBE2E2	ENSG0000018224	protein_coding;no	4.7355	6.9178	0.68	-0.55	1.24E-02	down
ACOT7	ENSG0000009702	protein_coding;no	8.1755	5.6366	1.45	0.54	1.24E-02	up
EBP	ENSG0000014715	protein_coding;pro	36.1157	27.2370	1.33	0.41	1.25E-02	up
CIITA	ENSG0000017958	protein_coding;ret	0.0450	0.0974	0.46	-1.11	1.25E-02	down
TGFA	ENSG0000016323	protein_coding;pro	1.4844	0.8607	1.72	0.79	1.25E-02	up
LRRC1	ENSG0000013726	protein_coding;no	0.1997	0.0825	2.42	1.27	1.25E-02	up
ARPC4	ENSG0000024155	protein_coding;ret	34.1095	48.7120	0.70	-0.51	1.25E-02	down
ZNF773	ENSG0000015243	protein_coding;no	0.2759	0.6135	0.45	-1.15	1.26E-02	down
FAM214B	ENSG0000000523	protein_coding;pro	2.5131	1.5386	1.63	0.71	1.26E-02	up
MTIF2	ENSG0000008576	protein_coding;pro	24.6718	31.9152	0.77	-0.37	1.26E-02	down
AC097534	ENSG0000024877	antisense	1.7430	0.6839	2.55	1.35	1.26E-02	up
ICOSLG	ENSG0000016022	protein_coding	0.2859	0.5062	0.56	-0.82	1.26E-02	down
C11orf1	ENSG0000013772	protein_coding	3.1365	4.7055	0.67	-0.59	1.26E-02	down
CARNMT1	ENSG0000015601	protein_coding	5.7065	7.7987	0.73	-0.45	1.26E-02	down
SIGLEC12	ENSG0000025452	protein_coding;no	2.3772	3.8816	0.61	-0.71	1.26E-02	down
EMC1	ENSG0000012746	protein_coding;ret	8.6169	6.5318	1.32	0.40	1.26E-02	up
BUB1B	ENSG0000015697	protein_coding;ret	12.3424	16.2910	0.76	-0.40	1.26E-02	down
PRKCQ	ENSG0000006567	protein_coding	0.3469	0.1064	3.26	1.70	1.26E-02	up
AC013489	ENSG0000017386	protein_coding;pro	0.3060	0.9239	0.33	-1.59	1.26E-02	down
BRCA2	ENSG0000013961	nonsense_mediate	3.7609	2.6850	1.40	0.49	1.27E-02	up
AC133644	ENSG0000028487	processed_transcri	2.8398	4.2899	0.66	-0.60	1.27E-02	down
AC115223	ENSG0000024319	processed_pseudo;	40.6147	54.8913	0.74	-0.43	1.27E-02	down
ID2	ENSG0000011573	protein_coding;ret	17.9217	12.9793	1.38	0.47	1.28E-02	up
GPI	ENSG0000010522	retained_intron;pro	24.6159	32.4298	0.76	-0.40	1.28E-02	down
MARCO	ENSG0000001916	protein_coding;ret	0.0715	0.4761	0.15	-2.74	1.29E-02	down
SGK1	ENSG0000011851	protein_coding;ret	3.4957	2.4186	1.45	0.53	1.29E-02	up
GOLGA8A	ENSG0000017526	processed_transcri	18.3722	13.8103	1.33	0.41	1.29E-02	up
SPART	ENSG0000013310	processed_transcri	19.4086	25.8251	0.75	-0.41	1.30E-02	down

TXNIP	ENSG0000026597	protein_coding;ret	93.9828	122.9292	0.76	-0.39	1.30E-02	down
EXOG	ENSG0000015703	protein_coding;no	1.6392	1.0046	1.63	0.71	1.30E-02	up
SIK1	ENSG0000014217	protein_coding;ret	0.1993	0.3949	0.50	-0.99	1.31E-02	down
WARS2	ENSG0000011687	protein_coding;pro	3.1082	4.6342	0.67	-0.58	1.31E-02	down
TMEM230	ENSG0000008906	protein_coding;pro	24.1223	12.4507	1.94	0.95	1.31E-02	up
RAB2B	ENSG0000012947	protein_coding;pro	3.9175	5.7747	0.68	-0.56	1.31E-02	down
GPR155	ENSG0000016332	protein_coding;pro	0.1433	0.0633	2.27	1.18	1.32E-02	up
ACTG1	ENSG0000018400	retained_intron;pro	277.9734	212.7925	1.31	0.39	1.32E-02	up
IL5RA	ENSG0000009118	protein_coding;pro	1.0977	0.6584	1.67	0.74	1.32E-02	up
CFD	ENSG0000019776	protein_coding	54.6707	40.9941	1.33	0.42	1.33E-02	up
RPL22L1	ENSG0000016358	retained_intron;pro	6.8384	11.0555	0.62	-0.69	1.33E-02	down
CD163	ENSG0000017757	protein_coding;ret	0.3026	0.5558	0.54	-0.88	1.33E-02	down
SLC27A5	ENSG0000008380	nonsense_mediate	1.2955	0.8184	1.58	0.66	1.33E-02	up
RBM3	ENSG0000010231	protein_coding;pro	35.4829	46.2316	0.77	-0.38	1.33E-02	down
ZNF23	ENSG0000016737	protein_coding;ret	1.0552	2.1270	0.50	-1.01	1.33E-02	down
ATP6V1G2-	ENSG0000025487	nonsense_mediate	1.9000	6.2485	0.30	-1.72	1.33E-02	down
ZNF311	ENSG0000019793	retained_intron;pro	1.2841	2.2817	0.56	-0.83	1.33E-02	down
FGFR1	ENSG0000007778	nonsense_mediate	0.1209	0.2192	0.55	-0.86	1.33E-02	down
NLN	ENSG0000012321	retained_intron;pro	3.1562	2.2562	1.40	0.48	1.34E-02	up
HSP90AB1	ENSG0000009638	protein_coding	463.9373	593.8594	0.78	-0.36	1.34E-02	down
AC046185	ENSG0000027936	TEC	5.5658	3.5168	1.58	0.66	1.34E-02	up
RAP1A	ENSG0000011647	processed_transcri	12.0672	16.6184	0.73	-0.46	1.34E-02	down
ST3GAL3	ENSG0000012609	nonsense_mediate	0.3549	0.5503	0.64	-0.63	1.35E-02	down
FOXP2	ENSG0000012857	nonsense_mediate	0.1052	0.1927	0.55	-0.87	1.35E-02	down
BTF3L4	ENSG0000013471	protein_coding;ret	7.8443	10.4507	0.75	-0.41	1.35E-02	down
POP5	ENSG0000016727	retained_intron;pro	14.8451	10.4047	1.43	0.51	1.36E-02	up
NCOA4	ENSG0000026641	protein_coding	119.2467	154.0507	0.77	-0.37	1.36E-02	down
NIFK	ENSG0000015543	protein_coding;ret	29.1499	38.1354	0.76	-0.39	1.36E-02	down
SLC25A36	ENSG0000011412	nonsense_mediate	11.0046	5.7536	1.91	0.94	1.37E-02	up
SERPINB8	ENSG0000016640	protein_coding	7.8005	10.4386	0.75	-0.42	1.37E-02	down
C17orf51	ENSG0000021271	protein_coding;lin	0.1493	0.0702	2.13	1.09	1.37E-02	up
PRUNE1	ENSG0000014336	protein_coding;pro	2.4842	4.2409	0.59	-0.77	1.38E-02	down
SUPT16HP1	ENSG0000025623	processed_pseudo;	0.4519	0.2102	2.15	1.10	1.38E-02	up
ATP6AP1	ENSG0000007155	protein_coding;pro	16.1873	12.1416	1.33	0.41	1.38E-02	up
FAM102A	ENSG0000016710	protein_coding;pro	0.3856	0.1973	1.95	0.97	1.38E-02	up
ARMCX5-G	ENSG0000027114	processed_transcri	0.5888	1.4815	0.40	-1.33	1.38E-02	down
DDX59	ENSG0000011819	protein_coding	2.5613	3.8622	0.66	-0.59	1.38E-02	down
PSPH	ENSG0000014673	protein_coding;no	1.1407	1.9089	0.60	-0.74	1.39E-02	down
AC010719	ENSG0000027093	lincRNA	1.6848	0.7708	2.19	1.13	1.39E-02	up
IFRD1	ENSG0000000665	protein_coding;pro	8.0503	10.9314	0.74	-0.44	1.40E-02	down
MTERF2	ENSG0000012083	protein_coding;pro	0.2802	0.1209	2.32	1.21	1.40E-02	up
DUSP12	ENSG0000008172	protein_coding;no	12.4827	17.5439	0.71	-0.49	1.40E-02	down
AUH	ENSG0000014809	protein_coding;pro	2.1402	3.4452	0.62	-0.69	1.40E-02	down
RPS26P8	ENSG0000020465	processed_pseudo;	5.7349	13.2489	0.43	-1.21	1.41E-02	down
AL354718	ENSG0000027516	processed_pseudo;	1.1459	2.3856	0.48	-1.06	1.41E-02	down
AMN1	ENSG0000015174	protein_coding;pro	1.6058	0.9300	1.73	0.79	1.41E-02	up
AC073349	ENSG0000023433	processed_pseudo;	0.8580	0.3739	2.29	1.20	1.41E-02	up
ARRDC1-A	ENSG0000020399	antisense	3.4474	2.0549	1.68	0.75	1.41E-02	up

GCNT1	ENSG0000018721	protein_coding;pr	0.8254	2.0614	0.40	-1.32	1.42E-02	down
TMEM109	ENSG0000011010	protein_coding;pr	49.6258	38.1294	1.30	0.38	1.42E-02	up
UBE2A	ENSG0000007772	protein_coding;ret	7.6163	11.2610	0.68	-0.56	1.42E-02	down
PPP2R3C	ENSG0000009202	protein_coding;no	7.6102	10.3280	0.74	-0.44	1.43E-02	down
CTDSPL2	ENSG0000013777	retained_intron;pr	4.7676	6.4058	0.74	-0.43	1.43E-02	down
ZNF467	ENSG0000018144	protein_coding	2.1712	1.2744	1.70	0.77	1.43E-02	up
DDTL	ENSG0000009997	protein_coding	15.1998	10.5161	1.45	0.53	1.43E-02	up
AL590867	ENSG0000021842	processed_pseudo	49.3608	68.9444	0.72	-0.48	1.43E-02	down
TNRC6A	ENSG0000009090	protein_coding;ret	3.3170	2.4109	1.38	0.46	1.43E-02	up
RMND1	ENSG0000015590	protein_coding;no	8.8379	11.9948	0.74	-0.44	1.44E-02	down
LARS	ENSG0000013370	protein_coding;ret	30.1012	38.7788	0.78	-0.37	1.44E-02	down
B3GLCT	ENSG0000018767	protein_coding;pr	0.7099	0.3659	1.94	0.96	1.44E-02	up
RPL6	ENSG0000008900	protein_coding;ret	306.9539	392.8701	0.78	-0.36	1.44E-02	down
ECHS1	ENSG0000012788	protein_coding	118.8903	91.9086	1.29	0.37	1.45E-02	up
WRNIP1	ENSG0000012453	protein_coding	12.0785	9.0090	1.34	0.42	1.45E-02	up
GIHCG	ENSG0000025769	retained_intron;lin	35.0507	46.2401	0.76	-0.40	1.45E-02	down
ARMC6	ENSG0000010567	protein_coding;no	5.2890	7.5387	0.70	-0.51	1.45E-02	down
BAZ2A	ENSG0000007610	protein_coding;ret	4.3329	3.0456	1.42	0.51	1.45E-02	up
AC079250	ENSG0000023097	processed_pseudo	14.0600	21.8244	0.64	-0.63	1.46E-02	down
ADGRE2	ENSG0000012750	protein_coding;no	2.0152	1.3594	1.48	0.57	1.46E-02	up
AC068888	ENSG0000025733	antisense	0.1825	0.4810	0.38	-1.40	1.47E-02	down
EPHX1	ENSG0000014381	protein_coding;pr	2.4230	1.4457	1.68	0.75	1.47E-02	up
EIF4E3	ENSG0000016341	protein_coding;pr	1.3496	0.8974	1.50	0.59	1.47E-02	up
MIR600HG	ENSG0000023690	sense_intronic	1.6655	1.0279	1.62	0.70	1.47E-02	up
ADI1	ENSG0000018255	protein_coding	19.9514	14.1638	1.41	0.49	1.47E-02	up
PDLIM5	ENSG0000016311	protein_coding;no	1.8344	1.2491	1.47	0.55	1.48E-02	up
CTSA	ENSG0000006460	protein_coding;ret	22.1868	16.7018	1.33	0.41	1.48E-02	up
TOMM40L	ENSG0000015888	protein_coding;pr	2.0767	4.6333	0.45	-1.16	1.48E-02	down
TTC9C	ENSG0000016222	nonsense_mediate	2.8399	4.5672	0.62	-0.69	1.48E-02	down
RPL18	ENSG0000006317	nonsense_mediate	164.1864	213.7742	0.77	-0.38	1.48E-02	down
TRIB3	ENSG0000010125	protein_coding	20.0847	27.4254	0.73	-0.45	1.49E-02	down
IPMK	ENSG0000015115	protein_coding	1.8523	2.7761	0.67	-0.58	1.49E-02	down
DENND5A	ENSG0000018401	retained_intron;pr	5.0446	6.6307	0.76	-0.39	1.49E-02	down
LINC00909	ENSG0000026424	lincRNA	4.3628	6.3479	0.69	-0.54	1.50E-02	down
SNORA44	ENSG0000027354	snoRNA	0.7735	5.3824	0.14	-2.80	1.50E-02	down
CRYBG3	ENSG0000008020	protein_coding;pr	0.9987	0.6279	1.59	0.67	1.50E-02	up
ADGRA3	ENSG0000015299	protein_coding;ret	4.6653	3.4963	1.33	0.42	1.50E-02	up
ASB7	ENSG0000018347	protein_coding;ret	1.2282	1.9139	0.64	-0.64	1.51E-02	down
MTG1	ENSG0000014882	protein_coding;ret	5.1189	3.5712	1.43	0.52	1.51E-02	up
FASN	ENSG0000016971	retained_intron;pr	6.4056	4.5099	1.42	0.51	1.52E-02	up
NPDC1	ENSG0000010728	protein_coding;ret	1.3965	0.7620	1.83	0.87	1.52E-02	up
EIF4B	ENSG0000006304	protein_coding;no	71.5564	107.5783	0.67	-0.59	1.52E-02	down
KIAA1211	ENSG0000010926	protein_coding;pr	0.2615	0.1153	2.27	1.18	1.52E-02	up
KLHL2	ENSG0000010946	protein_coding;pr	9.5114	6.9970	1.36	0.44	1.53E-02	up
MLST8	ENSG0000016796	protein_coding;ret	6.4501	8.8946	0.73	-0.46	1.53E-02	down
ICAM3	ENSG0000007666	retained_intron;pr	31.4391	22.8513	1.38	0.46	1.53E-02	up
CPM	ENSG0000013567	protein_coding;ret	0.8912	1.3946	0.64	-0.65	1.54E-02	down
ZNF664	ENSG0000017919	protein_coding;pr	17.9551	8.0158	2.24	1.16	1.55E-02	up

AKR1A1	ENSG0000011744	protein_coding;pro	22.6460	17.3029	1.31	0.39	1.55E-02	up
CCHCR1	ENSG0000020453	protein_coding;no	4.4705	3.0751	1.45	0.54	1.55E-02	up
ISL2	ENSG0000015955	protein_coding;no	2.3047	3.7489	0.61	-0.70	1.55E-02	down
UTP18	ENSG0000001126	nonsense_mediate	24.7807	31.9194	0.78	-0.37	1.56E-02	down
TUBB2A	ENSG0000013726	protein_coding;pro	0.6453	0.2928	2.20	1.14	1.56E-02	up
FNDC10	ENSG0000022859	protein_coding	3.4176	2.0678	1.65	0.72	1.56E-02	up
ATP5MC1	ENSG0000015919	processed_transcri	29.4901	22.3562	1.32	0.40	1.56E-02	up
NOLC1	ENSG0000016619	protein_coding;no	67.3236	51.7189	1.30	0.38	1.57E-02	up
ZNF518B	ENSG0000017816	protein_coding;ret	0.0734	0.1585	0.46	-1.11	1.57E-02	down
AL365181	ENSG0000027206	lincRNA	0.1490	0.3271	0.46	-1.13	1.58E-02	down
TERF2	ENSG0000013260	protein_coding;pro	2.9548	4.6795	0.63	-0.66	1.58E-02	down
MT-ND4L	ENSG0000021290	protein_coding	########	########	1.29	0.37	1.59E-02	up
NAXE	ENSG0000016338	protein_coding;pro	25.7760	19.1926	1.34	0.43	1.59E-02	up
NUS1P1	ENSG0000023563	processed_pseudo;	0.5980	1.6754	0.36	-1.49	1.59E-02	down
AL353622	ENSG0000027060	antisense	0.3529	0.7960	0.44	-1.17	1.60E-02	down
GBE1	ENSG0000011448	protein_coding;ret	15.1722	11.5506	1.31	0.39	1.60E-02	up
FAM217B	ENSG0000019622	protein_coding;pro	0.5949	1.6887	0.35	-1.51	1.60E-02	down
ERC1	ENSG0000008280	protein_coding;no	0.9114	0.5445	1.67	0.74	1.60E-02	up
USP49	ENSG0000016466	protein_coding;no	1.4100	2.0909	0.67	-0.57	1.61E-02	down
DUSP3	ENSG0000010886	protein_coding;pro	9.1354	11.8378	0.77	-0.37	1.61E-02	down
SATB1	ENSG0000018256	protein_coding;ret	1.8214	1.2631	1.44	0.53	1.61E-02	up
AHDC1	ENSG0000012670	protein_coding;pro	0.3193	0.1608	1.99	0.99	1.61E-02	up
TBCD	ENSG0000014155	protein_coding;pro	1.9731	1.3327	1.48	0.57	1.61E-02	up
GPR137B	ENSG0000007758	protein_coding;pro	3.3008	2.0340	1.62	0.70	1.61E-02	up
POLR3H	ENSG0000010041	protein_coding;no	4.6292	6.5156	0.71	-0.49	1.62E-02	down
TEP1	ENSG0000012956	protein_coding;no	1.3652	0.8857	1.54	0.62	1.62E-02	up
RAC2	ENSG0000012834	protein_coding;ret	136.8109	105.5948	1.30	0.37	1.62E-02	up
EPB41L5	ENSG0000011510	protein_coding;pro	0.1352	0.0603	2.24	1.16	1.62E-02	up
RPL13AP5	ENSG0000023655	processed_pseudo;	228.3428	299.8638	0.76	-0.39	1.62E-02	down
PIK3CB	ENSG0000005138	protein_coding;ret	7.9975	11.5865	0.69	-0.53	1.63E-02	down
FAM86C2P	ENSG0000016017	processed_transcri	0.4582	0.8473	0.54	-0.89	1.63E-02	down
CDK2AP2	ENSG0000016779	nonsense_mediate	5.4593	8.1609	0.67	-0.58	1.63E-02	down
CENPH	ENSG0000015304	protein_coding;ret	6.5510	9.6470	0.68	-0.56	1.63E-02	down
NANS	ENSG0000009538	processed_transcri	15.8031	11.9482	1.32	0.40	1.63E-02	up
PLEKHF2	ENSG0000017589	protein_coding	9.5876	13.0057	0.74	-0.44	1.63E-02	down
NDUFA3	ENSG0000017090	nonsense_mediate	16.8881	12.5719	1.34	0.43	1.64E-02	up
FTH1	ENSG0000016799	protein_coding;ret	79.2730	58.2270	1.36	0.45	1.64E-02	up
EGLN1	ENSG0000013576	protein_coding;pro	5.8813	7.6728	0.77	-0.38	1.64E-02	down
PLAC8	ENSG0000014528	protein_coding;pro	60.0494	76.8992	0.78	-0.36	1.64E-02	down
GMIP	ENSG0000008963	protein_coding;no	9.5568	6.8948	1.39	0.47	1.64E-02	up
HNRNPH1	ENSG0000016904	nonsense_mediate	17.3673	22.3909	0.78	-0.37	1.65E-02	down
BTRC	ENSG0000016616	protein_coding;pro	3.2761	2.2460	1.46	0.54	1.65E-02	up
CCDC115	ENSG0000013671	protein_coding;no	10.1187	7.4767	1.35	0.44	1.65E-02	up
NDUFB2	ENSG0000009026	nonsense_mediate	17.1338	12.6385	1.36	0.44	1.65E-02	up
NAT14	ENSG0000009097	protein_coding;pro	2.8699	1.5309	1.87	0.91	1.65E-02	up
PPA1	ENSG0000018081	retained_intron;pro	55.6479	71.2556	0.78	-0.36	1.66E-02	down
SLC5A3	ENSG0000019874	protein_coding	8.7187	6.5330	1.33	0.42	1.66E-02	up
ACSM3	ENSG0000000518	processed_transcri	3.7498	5.0870	0.74	-0.44	1.67E-02	down

ZNF573	ENSG0000018914	protein_coding;pro	0.5373	0.2699	1.99	0.99	1.67E-02	up
ITGA1	ENSG0000021394	protein_coding;pro	0.0735	0.0332	2.21	1.15	1.67E-02	up
MUC19	ENSG0000020559	processed_transcri	0.0484	0.0971	0.50	-1.00	1.68E-02	down
ASF1B	ENSG0000010501	protein_coding;no	20.6746	15.4469	1.34	0.42	1.68E-02	up
EPHA1-AS1	ENSG0000022915	antisense	0.1953	0.3881	0.50	-0.99	1.68E-02	down
SIVA1	ENSG0000018499	nonsense_mediate	20.4103	15.5527	1.31	0.39	1.69E-02	up
PPP1R15A	ENSG0000008707	protein_coding	11.3949	15.4700	0.74	-0.44	1.69E-02	down
RUFY1	ENSG0000017678	protein_coding;no	4.4728	6.0403	0.74	-0.43	1.69E-02	down
TADA3	ENSG0000017114	retained_intron;pro	19.9292	15.4356	1.29	0.37	1.70E-02	up
NPEPPS	ENSG0000014127	protein_coding;no	6.0173	4.6140	1.30	0.38	1.70E-02	up
DRAM2	ENSG0000015617	processed_transcri	16.9319	22.3914	0.76	-0.40	1.70E-02	down
SUV39H1	ENSG0000010194	protein_coding;pro	2.8639	4.7498	0.60	-0.73	1.70E-02	down
PKIG	ENSG0000016873	protein_coding;pro	0.6999	0.3377	2.07	1.05	1.71E-02	up
ROMO1	ENSG0000012599	protein_coding	36.7605	24.9721	1.47	0.56	1.71E-02	up
SENP6	ENSG0000011270	nonsense_mediate	6.2074	7.9608	0.78	-0.36	1.71E-02	down
LINC00339	ENSG0000021851	processed_transcri	0.5588	1.0054	0.56	-0.85	1.71E-02	down
IMPA1	ENSG0000013373	retained_intron;pro	7.2962	9.6879	0.75	-0.41	1.71E-02	down
KIF21B	ENSG0000011685	protein_coding;ret	4.4577	3.1322	1.42	0.51	1.71E-02	up
MMP2	ENSG0000008724	protein_coding;no	1.0672	1.7416	0.61	-0.71	1.71E-02	down
PECAM1	ENSG0000026137	protein_coding;pro	6.9227	9.0315	0.77	-0.38	1.71E-02	down
EMP3	ENSG0000014222	protein_coding;ret	50.4037	38.5569	1.31	0.39	1.72E-02	up
ARHGEF5	ENSG0000005032	protein_coding	0.1447	0.0404	3.58	1.84	1.72E-02	up
MR1	ENSG0000015302	protein_coding;pro	3.1332	1.8804	1.67	0.74	1.72E-02	up
MEF2B	ENSG0000021399	protein_coding	0.8188	0.2641	3.10	1.63	1.73E-02	up
PPM1L	ENSG0000016359	protein_coding;pro	1.0479	0.6891	1.52	0.60	1.73E-02	up
ARFGEF2	ENSG0000012419	protein_coding;pro	7.2114	9.3217	0.77	-0.37	1.73E-02	down
LINC01222	ENSG0000023341	lincRNA	1.1097	0.5281	2.10	1.07	1.76E-02	up
ATP1B3	ENSG0000006984	protein_coding;ret	53.4200	39.1985	1.36	0.45	1.76E-02	up
HNRNPAB	ENSG0000019745	protein_coding;ret	72.3340	92.2299	0.78	-0.35	1.76E-02	down
CMSS1	ENSG0000018422	protein_coding;pro	7.3909	9.5159	0.78	-0.36	1.76E-02	down
CILP2	ENSG0000016016	protein_coding;pro	2.6393	1.7065	1.55	0.63	1.77E-02	up
TOP1MT	ENSG0000018442	protein_coding;ret	14.0770	18.1312	0.78	-0.37	1.77E-02	down
SIGLEC14	ENSG0000025441	protein_coding;ret	4.1370	2.6183	1.58	0.66	1.77E-02	up
COPS7A	ENSG0000011165	protein_coding;no	6.6069	9.2842	0.71	-0.49	1.78E-02	down
PSMD1	ENSG0000017369	protein_coding;ret	15.5331	11.8939	1.31	0.39	1.78E-02	up
CDC6	ENSG0000009480	protein_coding;ret	11.1293	8.5388	1.30	0.38	1.78E-02	up
AC093323	ENSG0000017084	nonsense_mediate	8.8733	11.7750	0.75	-0.41	1.78E-02	down
AC121761	ENSG0000025749	antisense	0.6055	1.2487	0.48	-1.04	1.78E-02	down
FTH1P11	ENSG0000023726	processed_pseudo	3.3670	1.5824	2.13	1.09	1.79E-02	up
TNFRSF9	ENSG0000004924	protein_coding;no	0.1097	0.0414	2.65	1.41	1.79E-02	up
RPL7	ENSG0000014760	protein_coding;ret	533.2017	690.1498	0.77	-0.37	1.80E-02	down
DENND4C	ENSG0000013714	protein_coding;no	1.6397	1.1054	1.48	0.57	1.80E-02	up
CSK	ENSG0000010365	protein_coding;ret	7.3692	9.8514	0.75	-0.42	1.80E-02	down
ZC3H12A	ENSG0000016387	protein_coding;no	3.3219	2.1151	1.57	0.65	1.81E-02	up
PMF1-BGL	ENSG0000026023	protein_coding	9.0689	4.0958	2.21	1.15	1.81E-02	up
LYAR	ENSG0000014522	protein_coding;ret	23.9590	31.5536	0.76	-0.40	1.81E-02	down
SARS	ENSG0000003169	protein_coding;pro	19.4402	24.9530	0.78	-0.36	1.82E-02	down
GOLGA8N	ENSG0000023265	protein_coding;ret	2.2190	3.2551	0.68	-0.55	1.83E-02	down

CCDC30	ENSG0000018640	processed_transcri	0.0288	0.0614	0.47	-1.09	1.83E-02	down
ZNF815P	ENSG0000023594	transcribed_unpro	1.8183	1.0097	1.80	0.85	1.83E-02	up
RAN	ENSG0000013234	protein_coding;ret	61.3288	80.3325	0.76	-0.39	1.83E-02	down
TMBIM4	ENSG0000015595	nonsense_mediate	11.6804	15.1061	0.77	-0.37	1.84E-02	down
GCNT2	ENSG0000011184	processed_transcri	0.0850	0.0380	2.24	1.16	1.84E-02	up
ECHDC1	ENSG0000009314	nonsense_mediate	7.9865	10.4595	0.76	-0.39	1.84E-02	down
ZEB2	ENSG0000016955	protein_coding;ret	3.1891	4.3908	0.73	-0.46	1.84E-02	down
AD000671	ENSG0000026712	nonsense_mediate	0.9694	0.4265	2.27	1.18	1.84E-02	up
KARS	ENSG0000006542	protein_coding;no	47.1794	59.9888	0.79	-0.35	1.85E-02	down
REPS2	ENSG0000016989	protein_coding;pro	0.1373	0.0500	2.75	1.46	1.85E-02	up
CEP95	ENSG0000025889	retained_intron;no	4.5520	6.0007	0.76	-0.40	1.86E-02	down
LRP10	ENSG0000019732	protein_coding;ret	4.2933	2.9861	1.44	0.52	1.86E-02	up
ST7L	ENSG0000000734	nonsense_mediate	1.5653	2.2844	0.69	-0.55	1.86E-02	down
AC119396	ENSG0000026326	protein_coding	3.1953	1.9064	1.68	0.75	1.86E-02	up
POC5	ENSG0000015235	protein_coding;ret	3.2224	4.7239	0.68	-0.55	1.86E-02	down
NUMA1	ENSG0000013749	protein_coding;ret	4.3356	3.2559	1.33	0.41	1.86E-02	up
CENPA	ENSG0000011516	protein_coding;pro	2.7621	4.7875	0.58	-0.79	1.87E-02	down
TMEM183B	ENSG0000022483	processed_pseudo	4.3093	6.8540	0.63	-0.67	1.87E-02	down
RPL7P32	ENSG0000023100	processed_pseudo	8.3818	13.0044	0.64	-0.63	1.87E-02	down
CD14	ENSG0000017045	protein_coding	35.5167	25.9471	1.37	0.45	1.87E-02	up
TTK	ENSG0000011274	protein_coding;ret	10.4967	13.5348	0.78	-0.37	1.87E-02	down
FEN1	ENSG0000016849	protein_coding	91.7891	71.4740	1.28	0.36	1.88E-02	up
HIRIP3	ENSG0000014992	protein_coding;ret	19.0857	14.6203	1.31	0.38	1.88E-02	up
ZNF528	ENSG0000016755	protein_coding;ret	2.4848	1.5275	1.63	0.70	1.88E-02	up
RPSAP14	ENSG0000023398	processed_pseudo	3.7634	1.8121	2.08	1.05	1.88E-02	up
TSPAN31	ENSG0000013545	protein_coding;ret	4.0512	2.2965	1.76	0.82	1.89E-02	up
ZYG11B	ENSG0000016237	protein_coding	3.2473	2.3151	1.40	0.49	1.89E-02	up
TPGS2	ENSG0000013477	nonsense_mediate	12.7352	9.8346	1.29	0.37	1.89E-02	up
MTURN	ENSG0000018035	protein_coding	3.7720	1.6659	2.26	1.18	1.90E-02	up
ACOX1	ENSG0000016153	protein_coding;ret	3.2482	2.2981	1.41	0.50	1.90E-02	up
LAMTOR4	ENSG0000018818	protein_coding;pro	28.1587	21.2261	1.33	0.41	1.90E-02	up
RPS13	ENSG0000011070	protein_coding;pro	321.7401	411.9073	0.78	-0.36	1.91E-02	down
AC009090	ENSG0000027980	TEC	0.4033	0.1769	2.28	1.19	1.91E-02	up
MARCH1	ENSG0000014541	protein_coding;ret	1.1459	1.7196	0.67	-0.59	1.91E-02	down
MBP	ENSG0000019797	processed_transcri	6.8584	8.7376	0.78	-0.35	1.91E-02	down
BCKDK	ENSG0000010350	retained_intron;pro	7.1081	5.0451	1.41	0.49	1.91E-02	up
PRXL2B	ENSG0000015787	protein_coding;ret	4.0330	1.8713	2.16	1.11	1.91E-02	up
RPRD2	ENSG0000016312	protein_coding;pro	7.2311	5.5526	1.30	0.38	1.92E-02	up
IFNGR1	ENSG0000002769	protein_coding;no	34.6341	49.6655	0.70	-0.52	1.93E-02	down
B3GALT4	ENSG0000023586	protein_coding;pro	0.4920	0.2214	2.22	1.15	1.93E-02	up
PLEKHG2	ENSG0000009092	protein_coding;ret	0.0939	0.2053	0.46	-1.13	1.94E-02	down
DLEU1	ENSG0000017612	lincRNA;processe	0.6041	0.8731	0.69	-0.53	1.94E-02	down
SIPA1L2	ENSG0000011699	protein_coding;pro	0.4356	0.7089	0.61	-0.70	1.94E-02	down
NUP188	ENSG0000009531	retained_intron;pro	10.2366	7.8963	1.30	0.37	1.94E-02	up
PXN-AS1	ENSG0000025585	antisense	1.4694	0.8466	1.74	0.80	1.94E-02	up
PRKACB	ENSG0000014287	protein_coding;pro	5.4003	7.0650	0.76	-0.39	1.95E-02	down
CITED2	ENSG0000016444	protein_coding	39.8822	51.4232	0.78	-0.37	1.95E-02	down
KCTD1	ENSG0000013450	protein_coding;pro	0.2291	0.1098	2.09	1.06	1.95E-02	up

HCN3	ENSG0000014363	protein_coding;pro	0.7655	0.4254	1.80	0.85	1.95E-02	up
LRP6	ENSG0000007001	nonsense_mediate	0.9526	0.6166	1.54	0.63	1.95E-02	up
EEF1B2P6	ENSG0000021326	processed_pseudo	4.4417	7.2519	0.61	-0.71	1.95E-02	down
RPL26L1	ENSG0000003724	protein_coding	11.1123	16.2206	0.69	-0.55	1.95E-02	down
KRAS	ENSG0000013370	protein_coding	5.2737	6.9006	0.76	-0.39	1.96E-02	down
KIAA1755	ENSG0000014963	protein_coding;ret	0.2824	0.1268	2.23	1.16	1.97E-02	up
BCLAF1P2	ENSG0000027980	processed_pseudo	1.3651	2.4732	0.55	-0.86	1.97E-02	down
MALT1	ENSG0000017217	protein_coding;no	3.5264	2.3230	1.52	0.60	1.98E-02	up
MAP2K5	ENSG0000013776	protein_coding;ret	1.2628	1.9448	0.65	-0.62	1.98E-02	down
MYDGF	ENSG0000007484	protein_coding;ret	62.7469	47.6070	1.32	0.40	1.98E-02	up
WDR7	ENSG0000009115	protein_coding;pro	2.1454	1.5271	1.40	0.49	1.99E-02	up
RPS6	ENSG0000013715	protein_coding;pro	515.0162	652.2649	0.79	-0.34	1.99E-02	down
SUCNR1	ENSG0000019882	protein_coding	79.3160	61.5173	1.29	0.37	1.99E-02	up
TUBA1B	ENSG0000012341	protein_coding;ret	677.4952	519.1777	1.30	0.38	1.99E-02	up
CCL3L1	ENSG0000027608	retained_intron;pro	0.3696	0.7660	0.48	-1.05	1.99E-02	down
VAV2	ENSG0000016029	protein_coding;pro	0.3595	0.6159	0.58	-0.78	2.00E-02	down
PHTF2	ENSG0000000657	protein_coding;pro	1.9835	1.3505	1.47	0.55	2.00E-02	up
NDRG1	ENSG0000010441	retained_intron;pro	7.6267	10.0022	0.76	-0.39	2.00E-02	down
AAMDC	ENSG0000008788	protein_coding;no	4.0033	2.4820	1.61	0.69	2.01E-02	up
ZNF766	ENSG0000019621	protein_coding;ret	2.6134	4.4485	0.59	-0.77	2.01E-02	down
DCTN5	ENSG0000016684	protein_coding;ret	5.9160	3.4108	1.73	0.79	2.01E-02	up
SPACA6	ENSG0000018231	retained_intron;pro	0.4339	0.2116	2.05	1.04	2.02E-02	up
FSCN1	ENSG0000007561	protein_coding;ret	0.3090	0.6194	0.50	-1.00	2.02E-02	down
CNPY4	ENSG0000016699	processed_transcri	3.8680	2.4569	1.57	0.65	2.02E-02	up
CWC27	ENSG0000015301	protein_coding;pro	6.4582	8.8364	0.73	-0.45	2.02E-02	down
LAPTM4A	ENSG0000006869	protein_coding;ret	46.7361	36.0584	1.30	0.37	2.04E-02	up
RF02103	ENSG0000027608	misc_RNA	16.4746	27.4568	0.60	-0.74	2.04E-02	down
TRIB2	ENSG0000007157	protein_coding;pro	1.3557	0.8276	1.64	0.71	2.05E-02	up
EDEM2	ENSG0000008829	protein_coding	22.7557	17.2961	1.32	0.40	2.05E-02	up
SELPLG	ENSG0000011087	protein_coding	49.2355	38.3332	1.28	0.36	2.05E-02	up
SIRPB1	ENSG0000010130	protein_coding;no	14.3524	11.1931	1.28	0.36	2.05E-02	up
MAZ	ENSG0000010349	retained_intron;pro	21.0061	28.6315	0.73	-0.45	2.06E-02	down
GSTM3	ENSG0000013420	protein_coding;pro	0.3607	0.6410	0.56	-0.83	2.06E-02	down
AC024293	ENSG0000024431	processed_pseudo	133.2630	175.2268	0.76	-0.39	2.06E-02	down
NRBP1	ENSG0000011521	protein_coding;ret	18.1377	14.2514	1.27	0.35	2.08E-02	up
NONO	ENSG0000014714	protein_coding;pro	44.5001	58.9162	0.76	-0.40	2.10E-02	down
AL121832	ENSG0000027543	sense_intronic	0.3894	0.1773	2.20	1.13	2.10E-02	up
TLN1	ENSG0000013707	protein_coding;ret	13.7594	10.4380	1.32	0.40	2.10E-02	up
OFD1	ENSG0000004665	protein_coding;pro	8.3768	10.9974	0.76	-0.39	2.10E-02	down
GDF11	ENSG0000013541	protein_coding	0.7069	1.0952	0.65	-0.63	2.10E-02	down
DHTKD1	ENSG0000018119	protein_coding;ret	2.2330	1.4736	1.52	0.60	2.10E-02	up
DMXL2	ENSG0000010409	protein_coding;ret	7.5392	9.6714	0.78	-0.36	2.10E-02	down
RPL3P4	ENSG0000023257	processed_pseudo	578.3743	733.1482	0.79	-0.34	2.11E-02	down
FAM241A	ENSG0000017474	protein_coding	1.8471	2.6038	0.71	-0.50	2.12E-02	down
MIA3	ENSG0000015430	protein_coding;pro	11.5126	8.9322	1.29	0.37	2.12E-02	up
MTHFS	ENSG0000013637	protein_coding;no	0.7999	1.5062	0.53	-0.91	2.12E-02	down
INAFM2	ENSG0000025933	protein_coding;pro	4.9419	7.0687	0.70	-0.52	2.13E-02	down
ZNF496	ENSG0000016271	processed_transcri	2.5088	3.3597	0.75	-0.42	2.13E-02	down

AC006033	ENSG0000027290	lincRNA	1.3131	2.1856	0.60	-0.74	2.13E-02	down
CCDC28B	ENSG0000016005	protein_coding;no	0.6531	0.3211	2.03	1.02	2.14E-02	up
AC021078	ENSG0000023055	retained_intron	4.2997	5.6089	0.77	-0.38	2.14E-02	down
NAP1L1	ENSG0000018710	nonsense_mediate	37.1441	47.3851	0.78	-0.35	2.14E-02	down
SPTLC2	ENSG0000010059	protein_coding;ret	4.1500	3.1139	1.33	0.41	2.15E-02	up
G3BP2	ENSG0000013875	protein_coding;pro	28.2588	21.3952	1.32	0.40	2.15E-02	up
NT5C3A	ENSG0000012264	protein_coding;no	12.9839	19.1141	0.68	-0.56	2.15E-02	down
TRIM45	ENSG0000013425	protein_coding;no	0.4747	0.2188	2.17	1.12	2.16E-02	up
CYCS	ENSG0000017211	protein_coding	60.8428	77.8817	0.78	-0.36	2.16E-02	down
TMX4	ENSG0000012582	protein_coding;no	13.2225	10.3572	1.28	0.35	2.17E-02	up
MYPOP	ENSG0000017618	protein_coding	1.8499	1.0333	1.79	0.84	2.18E-02	up
SELL	ENSG0000018840	protein_coding;pro	1.1763	0.6782	1.73	0.79	2.18E-02	up
C9orf85	ENSG0000015562	protein_coding;no	2.1677	3.1618	0.69	-0.54	2.18E-02	down
YARS	ENSG0000013468	protein_coding;pro	14.0985	18.1707	0.78	-0.37	2.18E-02	down
DNAJC11	ENSG0000000792	protein_coding;pro	11.5542	8.8779	1.30	0.38	2.18E-02	up
MZT1	ENSG0000020489	protein_coding	17.5970	23.3851	0.75	-0.41	2.19E-02	down
TFG	ENSG0000011435	protein_coding;pro	11.7416	15.0224	0.78	-0.36	2.19E-02	down
PIP5K1A	ENSG0000014339	protein_coding;pro	5.5709	7.6377	0.73	-0.46	2.19E-02	down
IL18	ENSG0000015078	processed_transcri	7.3460	10.3541	0.71	-0.50	2.20E-02	down
TMEM9B	ENSG0000017534	protein_coding	8.2372	11.5181	0.72	-0.48	2.20E-02	down
FEM1B	ENSG0000016901	protein_coding	6.4609	8.2408	0.78	-0.35	2.21E-02	down
ZNF77	ENSG0000017569	protein_coding;pro	7.1577	4.8020	1.49	0.58	2.21E-02	up
IER3	ENSG0000013733	protein_coding	7.2175	4.6340	1.56	0.64	2.21E-02	up
IL3RA	ENSG0000018529	protein_coding	0.1710	0.6059	0.28	-1.82	2.22E-02	down
CCNG1	ENSG0000011332	protein_coding;ret	9.5154	12.6045	0.75	-0.41	2.22E-02	down
IER3IP1	ENSG0000013404	protein_coding	35.6456	27.9565	1.28	0.35	2.22E-02	up
CEMIP2	ENSG0000013504	protein_coding;ret	0.1514	0.0757	2.00	1.00	2.23E-02	up
ZNF879	ENSG0000023428	protein_coding	0.7654	0.4355	1.76	0.81	2.24E-02	up
EIF2S2P4	ENSG0000012869	processed_pseudo;	13.3094	19.1581	0.69	-0.53	2.24E-02	down
AC093525	ENSG0000026993	antisense	0.5293	0.2782	1.90	0.93	2.24E-02	up
PGM1	ENSG0000007973	protein_coding;pro	17.2952	13.3838	1.29	0.37	2.24E-02	up
ICMT	ENSG0000011623	nonsense_mediate	16.7000	11.5294	1.45	0.53	2.24E-02	up
SNRNP200	ENSG0000014402	protein_coding;ret	21.3487	16.6310	1.28	0.36	2.25E-02	up
ZCCHC8	ENSG0000003303	protein_coding;ret	4.9010	6.4720	0.76	-0.40	2.25E-02	down
DBNDD2	ENSG0000024427	protein_coding	6.1960	4.1915	1.48	0.56	2.25E-02	up
SNN	ENSG0000018460	protein_coding	5.4137	3.7518	1.44	0.53	2.25E-02	up
MTMR3	ENSG0000010033	protein_coding;ret	5.0613	3.8473	1.32	0.40	2.27E-02	up
ABCE1	ENSG0000016416	protein_coding;no	22.9670	29.0624	0.79	-0.34	2.27E-02	down
HSD17B7P2	ENSG0000009925	processed_transcri	0.9986	0.5048	1.98	0.98	2.27E-02	up
DEFA1	ENSG0000020604	protein_coding	2124.8753	2732.3319	0.78	-0.36	2.28E-02	down
ZNF451	ENSG0000011220	retained_intron;pro	2.3182	3.0292	0.77	-0.39	2.28E-02	down
TOP2B	ENSG0000007709	protein_coding;no	40.8773	51.6448	0.79	-0.34	2.28E-02	down
MRRF	ENSG0000014818	nonsense_mediate	2.8306	4.0762	0.69	-0.53	2.28E-02	down
SMIM3	ENSG0000025623	protein_coding	25.8318	32.9103	0.78	-0.35	2.28E-02	down
TMEM179B	ENSG0000018547	protein_coding;ret	18.0700	13.0323	1.39	0.47	2.29E-02	up
MTPAP	ENSG0000010795	protein_coding;pro	5.6434	7.2023	0.78	-0.35	2.29E-02	down
TPK1	ENSG0000019651	protein_coding;no	0.4828	0.7758	0.62	-0.68	2.30E-02	down
LDLRAD2	ENSG0000018794	protein_coding;pro	1.0035	0.5266	1.91	0.93	2.30E-02	up

ELOVL3	ENSG0000011991	protein_coding	2.7412	1.5968	1.72	0.78	2.30E-02	up
DIP2A	ENSG0000016030	retained_intron;pro	2.4781	1.7735	1.40	0.48	2.31E-02	up
AC147651	ENSG0000023718	antisense	2.0952	3.9210	0.53	-0.90	2.32E-02	down
RAB28	ENSG0000015786	protein_coding;no	8.6491	12.0488	0.72	-0.48	2.32E-02	down
BAMBI	ENSG0000009573	protein_coding;ret	1.5957	0.9329	1.71	0.77	2.32E-02	up
MMP15	ENSG0000010299	protein_coding	1.1965	0.7399	1.62	0.69	2.33E-02	up
NUP37	ENSG0000007518	protein_coding;ret	10.3390	13.6013	0.76	-0.40	2.33E-02	down
APOC2	ENSG0000023490	protein_coding	18.8516	12.4549	1.51	0.60	2.33E-02	up
DERL3	ENSG0000009995	protein_coding;ret	3.7725	2.5080	1.50	0.59	2.33E-02	up
MARC1	ENSG0000018620	protein_coding;pro	1.6341	1.1180	1.46	0.55	2.33E-02	up
NUDT1	ENSG0000010626	protein_coding;pro	28.9884	22.2064	1.31	0.38	2.34E-02	up
ZNF428	ENSG0000013111	protein_coding;ret	13.6780	9.4394	1.45	0.54	2.34E-02	up
BOLA1	ENSG0000017809	protein_coding;pro	3.4323	2.0189	1.70	0.77	2.34E-02	up
AP006621	ENSG0000025528	lincRNA	1.5581	0.8108	1.92	0.94	2.34E-02	up
ANKRD28	ENSG0000020656	processed_transcri	3.7488	5.1437	0.73	-0.46	2.35E-02	down
MRPL53	ENSG0000020482	protein_coding;ret	6.0270	11.0551	0.55	-0.88	2.35E-02	down
QTRT2	ENSG0000015157	retained_intron;pro	1.9057	3.1483	0.61	-0.72	2.36E-02	down
MVD	ENSG0000016750	protein_coding;ret	2.1542	1.3811	1.56	0.64	2.36E-02	up
TAF10	ENSG0000016633	retained_intron;pro	7.6252	5.8138	1.31	0.39	2.36E-02	up
FAM221A	ENSG0000018873	retained_intron;no	0.1888	0.0926	2.04	1.03	2.37E-02	up
ASAH2	ENSG0000018861	protein_coding;no	0.2096	0.1036	2.02	1.02	2.38E-02	up
APOE	ENSG0000013020	protein_coding;ret	0.5324	0.9874	0.54	-0.89	2.38E-02	down
ERCC5	ENSG0000013489	protein_coding;ret	7.7639	9.9411	0.78	-0.36	2.38E-02	down
SIGLEC17P	ENSG0000017110	processed_transcri	1.4098	2.3568	0.60	-0.74	2.38E-02	down
PLAGL2	ENSG0000012600	protein_coding	5.6508	7.4373	0.76	-0.40	2.38E-02	down
AASDH	ENSG0000015742	protein_coding;no	2.8323	4.0427	0.70	-0.51	2.39E-02	down
CCNQ	ENSG0000026291	protein_coding;no	3.4420	5.4674	0.63	-0.67	2.39E-02	down
CD3EAP	ENSG0000011787	protein_coding	3.4959	2.0035	1.74	0.80	2.39E-02	up
TREML2	ENSG0000011219	protein_coding	4.6327	3.1944	1.45	0.54	2.39E-02	up
KIF13A	ENSG0000013717	protein_coding;pro	0.4375	0.2627	1.67	0.74	2.40E-02	up
AC005076	ENSG0000022404	antisense	1.6095	0.7813	2.06	1.04	2.40E-02	up
TRAM2	ENSG0000006530	protein_coding	2.8323	1.9780	1.43	0.52	2.41E-02	up
SERPINE2	ENSG0000013591	protein_coding;ret	0.2099	0.0897	2.34	1.23	2.41E-02	up
AC116533	ENSG0000024439	processed_pseudo;	678.8471	857.9099	0.79	-0.34	2.41E-02	down
TRGC2	ENSG0000022719	TR_C_gene	58.7835	75.4646	0.78	-0.36	2.41E-02	down
AC107075	ENSG0000027799	unprocessed_pseu	4.9052	8.7567	0.56	-0.84	2.41E-02	down
QSOX1	ENSG0000011626	protein_coding;no	14.3162	10.8944	1.31	0.39	2.41E-02	up
SH3BGR	ENSG0000018543	protein_coding	0.2927	0.7422	0.39	-1.34	2.42E-02	down
C6orf163	ENSG0000020387	protein_coding;pro	0.7005	0.3611	1.94	0.96	2.42E-02	up
KREMEN1	ENSG0000018376	protein_coding;pro	0.1908	0.0922	2.07	1.05	2.43E-02	up
MMP25-AS	ENSG0000026197	antisense	0.7018	0.4250	1.65	0.72	2.44E-02	up
VMP1	ENSG0000006271	retained_intron;pro	7.9419	10.3255	0.77	-0.38	2.44E-02	down
SSH1	ENSG0000008411	nonsense_mediate	1.7723	1.2659	1.40	0.49	2.44E-02	up
MTMR1	ENSG0000006360	retained_intron;pro	5.1545	3.7793	1.36	0.45	2.44E-02	up
PLXND1	ENSG0000000439	retained_intron;pro	7.1741	5.2522	1.37	0.45	2.44E-02	up
RHBDD1	ENSG0000014446	protein_coding;ret	3.2113	4.4119	0.73	-0.46	2.44E-02	down
UBTD1	ENSG0000016588	protein_coding	2.3921	1.4188	1.69	0.75	2.45E-02	up
RPS15A	ENSG0000013441	protein_coding;no	180.0384	226.9886	0.79	-0.33	2.46E-02	down

SNHG3	ENSG0000024212	retained_intron;lin	16.0430	20.2623	0.79	-0.34	2.46E-02	down
ECM1	ENSG0000014336	processed_transcri	0.1721	0.3624	0.47	-1.07	2.46E-02	down
CFAP58	ENSG0000012005	protein_coding	0.7466	1.2582	0.59	-0.75	2.47E-02	down
AC140134	ENSG0000017997	unprocessed_pseu	0.6070	1.0177	0.60	-0.75	2.47E-02	down
USB1	ENSG0000010300	retained_intron;pr	4.6064	3.4662	1.33	0.41	2.47E-02	up
DNAJA2	ENSG0000006934	protein_coding;no	10.8723	13.7959	0.79	-0.34	2.47E-02	down
GALNS	ENSG0000014101	protein_coding;ret	2.5440	1.7325	1.47	0.55	2.48E-02	up
ERAP1	ENSG0000016430	protein_coding;ret	3.3104	2.3689	1.40	0.48	2.48E-02	up
ZNF410	ENSG0000011972	protein_coding;no	3.1038	4.6240	0.67	-0.58	2.49E-02	down
AC093668	ENSG0000028498	protein_coding	10.1815	4.5432	2.24	1.16	2.49E-02	up
FOLR3	ENSG0000011020	nonsense_mediate	1.9940	1.1231	1.78	0.83	2.49E-02	up
RSBN1	ENSG0000008101	protein_coding;no	3.6221	4.8965	0.74	-0.43	2.49E-02	down
INPP1	ENSG0000015168	protein_coding;pr	2.8636	1.8021	1.59	0.67	2.50E-02	up
MT-ND1	ENSG0000019888	protein_coding	########	8314.2769	1.29	0.37	2.50E-02	up
ZDHHC2	ENSG0000010421	protein_coding;ret	17.0813	21.4765	0.80	-0.33	2.51E-02	down
INKA2	ENSG0000019785	protein_coding;pr	1.6714	1.0831	1.54	0.63	2.51E-02	up
OSM	ENSG0000009998	protein_coding	0.2273	0.4921	0.46	-1.11	2.51E-02	down
BATF3	ENSG0000012368	protein_coding;pr	0.3258	0.7127	0.46	-1.13	2.51E-02	down
BCL2L2	ENSG0000012947	protein_coding	2.2873	6.8072	0.34	-1.57	2.52E-02	down
CBR3-AS1	ENSG0000023683	antisense;retained_	0.1551	0.0577	2.69	1.43	2.53E-02	up
IFT81	ENSG0000012297	protein_coding;pr	0.1857	0.3432	0.54	-0.89	2.53E-02	down
CES4A	ENSG0000017282	retained_intron;no	0.2404	0.0981	2.45	1.29	2.53E-02	up
CDS2	ENSG0000010129	protein_coding;pr	4.6634	3.6116	1.29	0.37	2.53E-02	up
EOGT	ENSG0000016337	protein_coding;ret	1.6589	1.0237	1.62	0.70	2.54E-02	up
AC009779	ENSG0000025805	antisense	2.2184	1.2477	1.78	0.83	2.54E-02	up
TMEM199	ENSG0000024404	protein_coding;ret	2.8528	1.7898	1.59	0.67	2.54E-02	up
ARHGAP17	ENSG0000014075	protein_coding;ret	2.5016	3.3524	0.75	-0.42	2.55E-02	down
RNH1	ENSG0000002319	protein_coding;no	10.4275	13.0794	0.80	-0.33	2.55E-02	down
AKAP8L	ENSG0000001124	retained_intron;no	8.2085	6.1940	1.33	0.41	2.55E-02	up
PIWIL4	ENSG0000013462	protein_coding;no	7.7306	10.1017	0.77	-0.39	2.56E-02	down
PMP22	ENSG0000010909	protein_coding;no	0.7070	1.2225	0.58	-0.79	2.56E-02	down
GPCPD1	ENSG0000012577	retained_intron;pr	13.5848	17.0686	0.80	-0.33	2.57E-02	down
MT-ND5	ENSG0000019878	protein_coding	3584.9320	2809.2345	1.28	0.35	2.57E-02	up
CES1	ENSG0000019884	protein_coding;no	22.1667	17.2706	1.28	0.36	2.58E-02	up
AGPAT2	ENSG0000016969	retained_intron;pr	13.9206	9.7821	1.42	0.51	2.58E-02	up
ATF4P3	ENSG0000022821	processed_pseudo	1.6116	2.8204	0.57	-0.81	2.58E-02	down
HNRNPA1P	ENSG0000022457	processed_transcri	23.5123	30.5918	0.77	-0.38	2.59E-02	down
PDLIM7	ENSG0000019692	protein_coding;no	6.3216	4.5954	1.38	0.46	2.61E-02	up
HYPK	ENSG0000024202	protein_coding;pr	2.6410	1.1473	2.30	1.20	2.61E-02	up
RAD50	ENSG0000011352	protein_coding;no	3.7581	5.0176	0.75	-0.42	2.62E-02	down
TNFRSF10C	ENSG0000017353	protein_coding;ret	2.7786	1.6461	1.69	0.76	2.62E-02	up
ARPC3P5	ENSG0000021402	processed_pseudo	0.9497	1.9976	0.48	-1.07	2.62E-02	down
E2F1	ENSG0000010141	protein_coding	18.1039	13.6715	1.32	0.41	2.62E-02	up
ADH5P4	ENSG0000023385	processed_pseudo	1.0397	0.5178	2.01	1.01	2.63E-02	up
SUSD1	ENSG0000010686	protein_coding;pr	4.5192	3.1185	1.45	0.54	2.65E-02	up
VLDLR-AS	ENSG0000023640	antisense	0.1713	0.3492	0.49	-1.03	2.65E-02	down
TXNL1	ENSG0000009116	protein_coding;no	9.4285	11.8620	0.79	-0.33	2.66E-02	down
ZNF596	ENSG0000017274	protein_coding;ret	0.2296	0.4732	0.49	-1.04	2.66E-02	down

BCAT2	ENSG0000010555	protein_coding;ret	6.6176	4.8082	1.38	0.46	2.66E-02	up
NDUFS7	ENSG0000011528	protein_coding;ret	4.6494	3.3860	1.37	0.46	2.66E-02	up
RNASE3	ENSG0000016939	protein_coding	6.7637	4.0266	1.68	0.75	2.66E-02	up
PSEN1	ENSG0000008081	protein_coding;no	5.0936	3.7294	1.37	0.45	2.66E-02	up
RUBCNL	ENSG0000010244	retained_intron;pro	3.0796	2.1479	1.43	0.52	2.69E-02	up
EP400	ENSG0000018349	protein_coding;ret	1.4419	1.0186	1.42	0.50	2.69E-02	up
PITHD1	ENSG0000005775	protein_coding	42.9222	33.6841	1.27	0.35	2.70E-02	up
ACSL3	ENSG0000012398	protein_coding;ret	10.5123	8.2793	1.27	0.34	2.70E-02	up
FAM98A	ENSG0000011981	retained_intron;pro	8.3436	6.3146	1.32	0.40	2.70E-02	up
STXBP5-AS	ENSG0000023345	antisense	0.7824	1.2262	0.64	-0.65	2.70E-02	down
HRH2	ENSG0000011374	nonsense_mediate	2.9154	4.0974	0.71	-0.49	2.71E-02	down
TMEM273	ENSG0000020416	processed_transcri	0.1525	0.3106	0.49	-1.03	2.71E-02	down
MAGT1	ENSG0000010215	protein_coding;ret	14.5003	10.6765	1.36	0.44	2.71E-02	up
HNRNPA1	ENSG0000013548	protein_coding;ret	333.9275	417.1201	0.80	-0.32	2.71E-02	down
TARBP2	ENSG0000013954	nonsense_mediate	2.6882	3.8442	0.70	-0.52	2.71E-02	down
NOP9	ENSG0000019694	protein_coding;no	8.3382	6.2453	1.34	0.42	2.71E-02	up
PIP5K1C	ENSG0000018611	protein_coding;pro	1.6155	1.0289	1.57	0.65	2.72E-02	up
AC073111	ENSG0000028469	processed_transcri	0.3817	0.1675	2.28	1.19	2.72E-02	up
HILPDA	ENSG0000013524	protein_coding;pro	10.8875	7.7426	1.41	0.49	2.72E-02	up
MTF1	ENSG0000018878	protein_coding;pro	5.0453	3.8542	1.31	0.39	2.72E-02	up
GTF2IRD2E	ENSG0000017442	protein_coding;ret	1.7975	1.2153	1.48	0.56	2.73E-02	up
DNAJB9	ENSG0000012859	protein_coding;pro	6.7673	9.4795	0.71	-0.49	2.73E-02	down
CLEC4D	ENSG0000016652	protein_coding	3.0048	4.5087	0.67	-0.59	2.73E-02	down
DHCR7	ENSG0000017289	retained_intron;pro	9.3548	6.7133	1.39	0.48	2.73E-02	up
PUS1	ENSG0000017719	protein_coding;ret	4.1819	5.8859	0.71	-0.49	2.74E-02	down
HSPA1B	ENSG0000020438	protein_coding	3.1993	2.0593	1.55	0.64	2.74E-02	up
CCNJ	ENSG0000010744	protein_coding	7.3716	9.5499	0.77	-0.37	2.74E-02	down
TMEM19	ENSG0000013929	protein_coding	1.2570	2.0823	0.60	-0.73	2.74E-02	down
REPS1	ENSG0000013559	protein_coding;no	3.6123	4.7082	0.77	-0.38	2.75E-02	down
SLC26A11	ENSG0000018104	protein_coding;ret	0.5174	0.2824	1.83	0.87	2.76E-02	up
PPP1R26	ENSG0000019642	protein_coding;pro	0.3628	0.1964	1.85	0.89	2.76E-02	up
FAM234A	ENSG0000016793	protein_coding;ret	3.0313	2.0472	1.48	0.57	2.76E-02	up
RCN2	ENSG0000011790	protein_coding;ret	3.9077	5.0428	0.77	-0.37	2.76E-02	down
RPA1	ENSG0000013238	retained_intron;pro	19.0841	15.0761	1.27	0.34	2.77E-02	up
RPS12	ENSG0000011230	retained_intron;pro	2195.5031	2751.3218	0.80	-0.33	2.77E-02	down
PVRIG	ENSG0000021341	protein_coding;pro	0.5178	0.1227	4.22	2.08	2.77E-02	up
PAN2	ENSG0000013547	retained_intron;pro	6.1098	7.7762	0.79	-0.35	2.77E-02	down
TRAF3IP2	ENSG0000005697	protein_coding;ret	2.5119	3.5747	0.70	-0.51	2.77E-02	down
AC104232	ENSG0000025400	lincRNA	7.2472	11.0509	0.66	-0.61	2.78E-02	down
FLNA	ENSG0000019692	protein_coding;ret	30.3545	23.0535	1.32	0.40	2.78E-02	up
DEF8	ENSG0000014099	protein_coding;ret	2.7249	1.6518	1.65	0.72	2.79E-02	up
SLX1A-SUL	ENSG0000021359	processed_transcri	1.4892	0.8040	1.85	0.89	2.79E-02	up
PIGW	ENSG0000027716	protein_coding	3.2303	5.1000	0.63	-0.66	2.79E-02	down
TMEM41A	ENSG0000016390	protein_coding;pro	1.2824	1.8434	0.70	-0.52	2.81E-02	down
ALG11	ENSG0000025371	protein_coding;pro	1.1454	1.9004	0.60	-0.73	2.82E-02	down
C6orf47	ENSG0000020443	protein_coding	7.1063	4.9897	1.42	0.51	2.82E-02	up
BMP8B	ENSG0000011698	protein_coding	1.0224	1.5564	0.66	-0.61	2.83E-02	down
PTX4	ENSG0000025169	protein_coding	2.7733	1.6268	1.70	0.77	2.83E-02	up

HKR1	ENSG0000018166	retained_intron;pr	3.8473	2.2299	1.73	0.79	2.85E-02	up
KDELC2	ENSG0000017820	protein_coding;pr	7.9363	6.0736	1.31	0.39	2.85E-02	up
PROS1	ENSG0000018450	protein_coding;no	0.4060	0.2073	1.96	0.97	2.85E-02	up
ZNF512	ENSG0000024394	retained_intron;pr	19.2963	15.1738	1.27	0.35	2.85E-02	up
PLCH1	ENSG0000011480	protein_coding;ret	0.6291	0.9799	0.64	-0.64	2.85E-02	down
ZNF558	ENSG0000016778	protein_coding;ret	1.1124	1.6124	0.69	-0.54	2.86E-02	down
IFI27L1	ENSG0000016594	protein_coding;ret	4.3560	2.8518	1.53	0.61	2.86E-02	up
FAM49A	ENSG0000019787	protein_coding;ret	0.4969	0.8718	0.57	-0.81	2.87E-02	down
ORMDL1	ENSG0000012869	protein_coding;pr	11.8231	16.8139	0.70	-0.51	2.87E-02	down
STK40	ENSG0000019618	protein_coding;pr	5.0249	3.6744	1.37	0.45	2.88E-02	up
PRPS2	ENSG0000010191	protein_coding	17.7856	12.9570	1.37	0.46	2.88E-02	up
EVL	ENSG0000019640	protein_coding;ret	4.8211	3.7088	1.30	0.38	2.88E-02	up
PTTG1	ENSG0000016461	protein_coding;ret	16.7896	21.5487	0.78	-0.36	2.90E-02	down
TUSC1	ENSG0000019868	protein_coding	2.2863	1.3562	1.69	0.75	2.90E-02	up
RPL3	ENSG0000010031	protein_coding;pr	626.7709	789.9211	0.79	-0.33	2.91E-02	down
BZW1	ENSG0000008215	protein_coding;ret	21.8817	31.5015	0.69	-0.53	2.91E-02	down
SENP1	ENSG0000007938	protein_coding;no	4.3127	6.2350	0.69	-0.53	2.92E-02	down
PGAP1	ENSG0000019712	retained_intron;pr	1.7142	1.0070	1.70	0.77	2.92E-02	up
STARD4	ENSG0000016421	protein_coding;no	5.1354	3.7001	1.39	0.47	2.92E-02	up
IQGAP2	ENSG0000014570	protein_coding;no	5.5643	4.3017	1.29	0.37	2.93E-02	up
AC011462	ENSG0000010532	protein_coding;ret	9.5716	6.8159	1.40	0.49	2.93E-02	up
CALM3	ENSG0000016001	protein_coding;ret	26.6554	19.8340	1.34	0.43	2.94E-02	up
PRR11	ENSG0000006848	nonsense_mediate	7.0209	9.2099	0.76	-0.39	2.94E-02	down
GINS2	ENSG0000013115	protein_coding	10.5726	7.7650	1.36	0.45	2.96E-02	up
INTS3	ENSG0000014362	protein_coding;no	2.9820	3.8413	0.78	-0.37	2.96E-02	down
UBA5	ENSG0000008130	protein_coding;ret	6.0242	7.7470	0.78	-0.36	2.96E-02	down
FOXRED1	ENSG0000011007	nonsense_mediate	5.2920	3.8913	1.36	0.44	2.97E-02	up
CLEC11A	ENSG0000010547	protein_coding	75.6116	58.3525	1.30	0.37	2.97E-02	up
RIC8B	ENSG0000011178	protein_coding;ret	1.2963	0.8263	1.57	0.65	2.98E-02	up
ZNF418	ENSG0000019672	protein_coding;pr	1.9563	1.0109	1.94	0.95	2.98E-02	up
PPP5C	ENSG0000001148	protein_coding;ret	8.0982	6.1134	1.32	0.41	2.98E-02	up
GRB2	ENSG0000017788	protein_coding;no	16.2961	29.4061	0.55	-0.85	2.98E-02	down
TMPPE	ENSG0000018816	protein_coding	1.1450	0.6495	1.76	0.82	2.99E-02	up
TBC1D4	ENSG0000013611	protein_coding;pr	2.1843	1.5001	1.46	0.54	2.99E-02	up
PLEKHM3	ENSG0000017838	protein_coding;pr	1.3195	0.9204	1.43	0.52	3.00E-02	up
OSBPL1A	ENSG0000014144	nonsense_mediate	11.5932	14.7244	0.79	-0.34	3.01E-02	down
SLC25A28	ENSG0000015528	protein_coding;ret	15.1343	21.1883	0.71	-0.49	3.02E-02	down
RPS24	ENSG0000013832	protein_coding;ret	252.5938	358.2738	0.71	-0.50	3.02E-02	down
ILK	ENSG0000016633	protein_coding;pr	17.1757	13.4388	1.28	0.35	3.02E-02	up
FHIT	ENSG0000018928	protein_coding;ret	0.6728	1.0302	0.65	-0.61	3.02E-02	down
NICN1	ENSG0000014502	protein_coding;pr	0.4502	0.8781	0.51	-0.96	3.02E-02	down
CHMP2A	ENSG0000013072	protein_coding;ret	29.1864	22.8099	1.28	0.36	3.02E-02	up
RASSF1	ENSG0000006802	protein_coding;no	12.3203	15.9001	0.77	-0.37	3.02E-02	down
AL356414	ENSG0000020530	lincRNA	2.3303	4.1699	0.56	-0.84	3.03E-02	down
LIAS	ENSG0000012189	retained_intron;pr	1.3737	1.9119	0.72	-0.48	3.03E-02	down
BMP1	ENSG0000016848	protein_coding;no	0.8922	0.5638	1.58	0.66	3.04E-02	up
RNASEH1-/	ENSG0000023417	antisense	5.7055	8.4428	0.68	-0.57	3.06E-02	down
P4HA1	ENSG0000012288	protein_coding;ret	27.4383	21.7220	1.26	0.34	3.07E-02	up

BBS12	ENSG0000018100	protein_coding	0.7633	0.4290	1.78	0.83	3.08E-02	up
RNF165	ENSG0000014162	protein_coding;no	0.8058	0.5306	1.52	0.60	3.09E-02	up
AC093591	ENSG0000024402	processed_pseudo;	82.4649	106.6321	0.77	-0.37	3.09E-02	down
SLC9A3R1	ENSG0000010906	protein_coding;ret	11.0431	6.0871	1.81	0.86	3.09E-02	up
SVIL-AS1	ENSG0000022459	processed_transcri	0.4528	0.9703	0.47	-1.10	3.09E-02	down
NFYA	ENSG0000000116	protein_coding	17.0003	13.3801	1.27	0.35	3.10E-02	up
FAM111B	ENSG0000018905	protein_coding;ret	0.3139	0.1541	2.04	1.03	3.10E-02	up
RAD51AP1	ENSG0000011124	protein_coding;ret	19.2922	14.5019	1.33	0.41	3.11E-02	up
RPL12	ENSG0000019795	protein_coding;pro	907.6036	1138.9803	0.80	-0.33	3.11E-02	down
HDLBP	ENSG0000011567	protein_coding;ret	18.7910	14.8990	1.26	0.33	3.11E-02	up
NUDT3	ENSG0000027232	protein_coding	20.0353	13.1348	1.53	0.61	3.11E-02	up
VPS37C	ENSG0000016798	protein_coding;ret	3.3139	4.7131	0.70	-0.51	3.13E-02	down
MELTF	ENSG0000016397	retained_intron;pro	0.1792	0.0942	1.90	0.93	3.13E-02	up
LGALS1	ENSG0000010009	protein_coding;ret	72.6981	57.3017	1.27	0.34	3.13E-02	up
KLHL22	ENSG0000009991	protein_coding;pro	1.4920	0.9501	1.57	0.65	3.14E-02	up
UBE3C	ENSG0000000933	retained_intron;pro	7.5994	9.5177	0.80	-0.32	3.14E-02	down
PDXDC1	ENSG0000017988	protein_coding;ret	11.1713	8.9307	1.25	0.32	3.14E-02	up
RPP21	ENSG0000024137	processed_transcri	11.1814	7.9307	1.41	0.50	3.15E-02	up
NT5DC2	ENSG0000016826	protein_coding;ret	5.4383	3.8768	1.40	0.49	3.15E-02	up
GON7	ENSG0000017027	protein_coding	14.3175	19.9675	0.72	-0.48	3.15E-02	down
SPINT2	ENSG0000016764	retained_intron;pro	0.2180	0.0660	3.30	1.72	3.15E-02	up
PCGF3	ENSG0000018561	protein_coding;no	5.5841	4.2050	1.33	0.41	3.15E-02	up
CLASP2	ENSG0000016353	protein_coding;ret	4.2658	5.3857	0.79	-0.34	3.15E-02	down
TRPV1	ENSG0000019668	nonsense_mediate	0.3757	0.1930	1.95	0.96	3.16E-02	up
CPNE2	ENSG0000014084	retained_intron;pro	6.2838	4.7833	1.31	0.39	3.16E-02	up
SPECC1	ENSG0000012848	protein_coding;pro	0.6188	0.3911	1.58	0.66	3.16E-02	up
GBA	ENSG0000017762	processed_transcri	13.2645	10.1729	1.30	0.38	3.17E-02	up
SLC2A3	ENSG0000005980	retained_intron;pro	9.1058	7.0661	1.29	0.37	3.17E-02	up
TMED6	ENSG0000015731	protein_coding;ret	0.3011	0.6357	0.47	-1.08	3.17E-02	down
SOBP	ENSG0000011232	protein_coding;pro	0.1495	0.2698	0.55	-0.85	3.17E-02	down
ZC3H12D	ENSG0000017819	protein_coding;pro	0.4790	0.7614	0.63	-0.67	3.18E-02	down
CD84	ENSG0000006629	protein_coding;ret	5.7723	4.5224	1.28	0.35	3.18E-02	up
AC133552	ENSG0000026258	processed_transcri	0.9122	0.4506	2.02	1.02	3.18E-02	up
CD38	ENSG0000000446	protein_coding;ret	5.3048	6.8496	0.77	-0.37	3.18E-02	down
MRFAP1	ENSG0000017901	protein_coding;ret	38.2807	67.2557	0.57	-0.81	3.19E-02	down
COPS6	ENSG0000016809	nonsense_mediate	23.1410	18.0079	1.29	0.36	3.20E-02	up
USP31	ENSG0000010340	protein_coding	0.9768	0.5085	1.92	0.94	3.20E-02	up
IFIT2	ENSG0000011992	protein_coding	0.1884	0.0829	2.27	1.18	3.20E-02	up
USP45	ENSG0000012355	protein_coding;ret	1.7596	2.6020	0.68	-0.56	3.21E-02	down
POLR2M	ENSG0000025552	protein_coding;no	4.0955	5.5093	0.74	-0.43	3.22E-02	down
ATP2A2	ENSG0000017443	protein_coding;ret	5.4595	7.3449	0.74	-0.43	3.25E-02	down
EIF3L	ENSG0000010012	protein_coding;no	143.1234	179.4755	0.80	-0.33	3.25E-02	down
ITGA5	ENSG0000016163	retained_intron;pro	6.4159	8.2226	0.78	-0.36	3.27E-02	down
RASGRP4	ENSG0000017177	retained_intron;pro	0.6391	0.3511	1.82	0.86	3.27E-02	up
FHL1	ENSG0000002226	protein_coding;pro	0.3183	0.1702	1.87	0.90	3.28E-02	up
VDR	ENSG0000011142	protein_coding;no	1.5749	2.5929	0.61	-0.72	3.28E-02	down
IRF5	ENSG0000012860	retained_intron;pro	1.3559	2.0737	0.65	-0.61	3.28E-02	down
VPS13D	ENSG0000004870	protein_coding;no	1.7346	1.3155	1.32	0.40	3.28E-02	up

HPS4	ENSG0000010009	nonsense_mediate	4.1688	5.2594	0.79	-0.34	3.29E-02	down
SNX3	ENSG0000011233	nonsense_mediate	80.1888	103.3088	0.78	-0.37	3.29E-02	down
LINC00205	ENSG0000022376	bidirectional_pron	0.5262	0.1632	3.22	1.69	3.29E-02	up
SNX10	ENSG0000008630	protein_coding;pro	8.2609	13.4392	0.61	-0.70	3.29E-02	down
CHD7	ENSG0000017131	protein_coding;ret	2.4088	1.8134	1.33	0.41	3.30E-02	up
RN7SK	ENSG0000028329	snRNA	8.3301	4.6929	1.78	0.83	3.30E-02	up
FAM204A	ENSG0000016566	processed_transcri	2.9127	3.6782	0.79	-0.34	3.30E-02	down
SETD6	ENSG0000010303	protein_coding;ret	6.3981	3.7140	1.72	0.78	3.31E-02	up
OPA1	ENSG0000019883	protein_coding;no	9.3449	11.7716	0.79	-0.33	3.31E-02	down
RAB1B	ENSG0000017490	protein_coding	61.9774	49.1354	1.26	0.33	3.31E-02	up
ACVR2A	ENSG0000012198	protein_coding;pro	0.2780	0.1475	1.89	0.91	3.32E-02	up
JOSD2	ENSG0000016167	protein_coding;ret	2.7168	1.6254	1.67	0.74	3.32E-02	up
PLTP	ENSG0000010097	protein_coding	3.7180	5.3317	0.70	-0.52	3.32E-02	down
MOGS	ENSG0000011527	retained_intron;no	4.2385	6.3913	0.66	-0.59	3.32E-02	down
AC009302	ENSG0000023386	processed_pseudo;	15.7493	10.1027	1.56	0.64	3.33E-02	up
ZNF22	ENSG0000016551	protein_coding	28.4092	35.9772	0.79	-0.34	3.33E-02	down
COPRS	ENSG0000017230	protein_coding;no	16.6352	11.8959	1.40	0.48	3.33E-02	up
STBD1	ENSG0000011880	protein_coding	6.5812	4.7720	1.38	0.46	3.34E-02	up
PHF14	ENSG0000010644	retained_intron;pro	2.6338	3.3776	0.78	-0.36	3.35E-02	down
NXF1	ENSG0000016223	retained_intron;pro	12.0601	9.4703	1.27	0.35	3.35E-02	up
MAP1S	ENSG0000013047	protein_coding;no	1.7648	1.1108	1.59	0.67	3.35E-02	up
AP000781	ENSG0000025497	protein_coding;ret	13.1407	1.4744	8.91	3.16	3.36E-02	up
ACLY	ENSG0000013147	protein_coding;pro	26.5445	20.8049	1.28	0.35	3.36E-02	up
SMG1P5	ENSG0000018360	processed_transcri	2.4429	3.5320	0.69	-0.53	3.36E-02	down
FGD5-AS1	ENSG0000022573	antisense	24.2038	19.2709	1.26	0.33	3.36E-02	up
PTPRS	ENSG0000010542	protein_coding;ret	0.1322	0.2405	0.55	-0.86	3.37E-02	down
GLUD1P3	ENSG0000025095	processed_transcri	1.1605	0.5354	2.17	1.12	3.38E-02	up
BMP2K	ENSG0000013875	protein_coding;no	7.1356	5.5754	1.28	0.36	3.39E-02	up
AL138759	ENSG0000024433	sense_overlapping	4.9798	2.9588	1.68	0.75	3.39E-02	up
ARPC1A	ENSG0000024168	protein_coding;pro	16.6723	12.5780	1.33	0.41	3.39E-02	up
SLC40A1	ENSG0000013844	protein_coding;ret	1.7278	1.1350	1.52	0.61	3.41E-02	up
KCNE1	ENSG0000018050	processed_transcri	0.4464	0.2498	1.79	0.84	3.41E-02	up
C1orf162	ENSG0000014311	protein_coding;pro	48.4732	38.7408	1.25	0.32	3.41E-02	up
ADM	ENSG0000014892	protein_coding	1.8811	1.1658	1.61	0.69	3.41E-02	up
MIR497HG	ENSG0000026753	antisense	0.4672	0.1711	2.73	1.45	3.42E-02	up
RASA3	ENSG0000018598	protein_coding;pro	7.6419	9.8344	0.78	-0.36	3.42E-02	down
SLC25A3	ENSG0000007541	protein_coding;no	30.6887	38.6947	0.79	-0.33	3.42E-02	down
RBMXL1	ENSG0000021351	protein_coding	8.4653	6.5362	1.30	0.37	3.42E-02	up
IGF2R	ENSG0000019708	protein_coding;ret	5.4674	4.3478	1.26	0.33	3.42E-02	up
LMNB2	ENSG0000017661	processed_transcri	11.1338	14.1125	0.79	-0.34	3.43E-02	down
NMT2	ENSG0000015246	protein_coding;pro	0.0682	0.1455	0.47	-1.09	3.44E-02	down
INSIG1	ENSG0000018648	protein_coding;ret	12.8793	17.3172	0.74	-0.43	3.44E-02	down
NMNAT3	ENSG0000016386	nonsense_mediate	0.2880	0.4438	0.65	-0.62	3.44E-02	down
MGST1	ENSG0000000839	protein_coding;TE	15.8660	19.7095	0.80	-0.31	3.45E-02	down
MON1B	ENSG0000010311	protein_coding;ret	4.6030	6.0127	0.77	-0.39	3.46E-02	down
ATF3	ENSG0000016277	protein_coding;ret	1.0067	1.5546	0.65	-0.63	3.47E-02	down
PCDHGA10	ENSG0000025384	protein_coding	0.0696	0.1460	0.48	-1.07	3.47E-02	down
CPLANE2	ENSG0000013288	protein_coding	0.6343	0.3214	1.97	0.98	3.47E-02	up

FYTTD1	ENSG0000012206	protein_coding;no	6.7108	4.4168	1.52	0.60	3.49E-02	up
AKT3	ENSG0000011702	protein_coding;pro	0.5732	0.8619	0.67	-0.59	3.50E-02	down
RAB3IP	ENSG0000012732	protein_coding;ret	0.8910	0.5999	1.49	0.57	3.50E-02	up
RTEL1-TNF	ENSG0000002603	retained_intron;no	0.6193	0.3293	1.88	0.91	3.50E-02	up
LPAR2	ENSG0000006454	retained_intron;pro	3.8099	2.5222	1.51	0.60	3.50E-02	up
PIGG	ENSG0000017422	protein_coding;no	3.2101	2.4386	1.32	0.40	3.52E-02	up
DPH6	ENSG0000013414	protein_coding;pro	0.7404	1.0794	0.69	-0.54	3.53E-02	down
BROX	ENSG0000016281	protein_coding;pro	7.5165	9.5947	0.78	-0.35	3.53E-02	down
AGPAT5	ENSG0000015518	protein_coding;pro	16.7968	24.4196	0.69	-0.54	3.53E-02	down
AC020915	ENSG0000026851	antisense	1.3541	0.7683	1.76	0.82	3.53E-02	up
TMEM30A	ENSG0000011269	protein_coding	39.7387	31.0763	1.28	0.35	3.54E-02	up
PITPNC1	ENSG0000015421	protein_coding;ret	2.8579	3.8648	0.74	-0.44	3.54E-02	down
PAM	ENSG0000014573	protein_coding;no	2.2042	1.5815	1.39	0.48	3.54E-02	up
HIST1H2BD	ENSG0000015837	protein_coding	2.2503	1.2212	1.84	0.88	3.55E-02	up
MAGI3	ENSG0000008102	protein_coding;pro	0.2129	0.1158	1.84	0.88	3.56E-02	up
NAA20	ENSG0000017341	processed_transcri	18.7423	23.5966	0.79	-0.33	3.57E-02	down
RPS20	ENSG0000000898	protein_coding;ret	217.4617	271.8058	0.80	-0.32	3.58E-02	down
EMILIN2	ENSG0000013220	protein_coding;ret	6.1764	4.7904	1.29	0.37	3.58E-02	up
GFOD2	ENSG0000014109	retained_intron;pro	1.9135	1.3550	1.41	0.50	3.59E-02	up
AL049634	ENSG0000026086	protein_coding	1.2376	0.6230	1.99	0.99	3.59E-02	up
CYB5B	ENSG0000010301	retained_intron;pro	12.3838	16.6822	0.74	-0.43	3.60E-02	down
WBP1L	ENSG0000016627	protein_coding;pro	6.1973	4.8058	1.29	0.37	3.61E-02	up
ZNF528-AS	ENSG0000026983	lincRNA;retained_	1.8920	1.1736	1.61	0.69	3.61E-02	up
RPS7P11	ENSG0000021332	processed_pseudo	102.1535	127.6178	0.80	-0.32	3.62E-02	down
FCAR	ENSG0000018643	protein_coding;pro	15.2372	11.8452	1.29	0.36	3.62E-02	up
LIN7C	ENSG0000014894	protein_coding	4.5868	6.4891	0.71	-0.50	3.63E-02	down
SLC50A1	ENSG0000016924	nonsense_mediate	13.0456	9.5258	1.37	0.45	3.64E-02	up
RNASEK-C	ENSG0000016193	retained_intron;pro	10.9944	7.2610	1.51	0.60	3.65E-02	up
ZDHHC7	ENSG0000015378	protein_coding;no	14.2432	11.1761	1.27	0.35	3.65E-02	up
PGD	ENSG0000014265	protein_coding;no	119.0223	95.2759	1.25	0.32	3.65E-02	up
ANKLE1	ENSG0000016011	nonsense_mediate	4.9903	3.2566	1.53	0.62	3.66E-02	up
SPDL1	ENSG0000004027	protein_coding;pro	4.6272	6.1808	0.75	-0.42	3.66E-02	down
ANKRD18D	ENSG0000022643	processed_transcri	0.2061	0.4217	0.49	-1.03	3.66E-02	down
AL671277	ENSG0000023766	unprocessed_pseu	1.7020	0.6033	2.82	1.50	3.66E-02	up
RPS3AP25	ENSG0000023238	processed_pseudo	2.3210	3.8165	0.61	-0.72	3.66E-02	down
ABCA5	ENSG0000015426	nonsense_mediate	0.8664	0.5877	1.47	0.56	3.67E-02	up
CTH	ENSG0000011676	protein_coding;pro	1.8876	2.8516	0.66	-0.60	3.67E-02	down
ARMC1	ENSG0000010444	protein_coding;no	16.2849	11.5124	1.41	0.50	3.68E-02	up
RCC2	ENSG0000017905	protein_coding;pro	27.9750	35.3107	0.79	-0.34	3.69E-02	down
CHPF	ENSG0000012398	protein_coding	0.1973	0.3708	0.53	-0.91	3.69E-02	down
EEF1A1	ENSG0000015650	protein_coding;ret	3152.1325	3902.6746	0.81	-0.31	3.70E-02	down
DUSP5P1	ENSG0000018392	processed_pseudo	1.6568	0.9018	1.84	0.88	3.70E-02	up
ATP5ME	ENSG0000016902	retained_intron;pro	107.4871	85.6145	1.26	0.33	3.70E-02	up
THOC7	ENSG0000016363	protein_coding;ret	33.2901	41.5678	0.80	-0.32	3.71E-02	down
GPR34	ENSG0000017165	protein_coding	1.3677	0.7974	1.72	0.78	3.72E-02	up
CMTR2	ENSG0000018091	protein_coding;ret	4.8158	6.1570	0.78	-0.35	3.72E-02	down
AP001324	ENSG0000022761	processed_pseudo	120.3472	151.8570	0.79	-0.34	3.72E-02	down
OCRL	ENSG0000012212	protein_coding;no	2.9603	4.0376	0.73	-0.45	3.72E-02	down

LYL1	ENSG0000010490	protein_coding;ret	21.7485	27.3865	0.79	-0.33	3.73E-02	down
RFC5	ENSG0000011144	protein_coding;pr	20.0455	15.7874	1.27	0.34	3.73E-02	up
SH2B3	ENSG0000011125	protein_coding	3.7688	5.0444	0.75	-0.42	3.73E-02	down
AC026401	ENSG0000028020	lincRNA	4.3396	6.6866	0.65	-0.62	3.73E-02	down
TAF6	ENSG0000010629	protein_coding;ret	5.9486	4.2821	1.39	0.47	3.74E-02	up
CHAF1B	ENSG0000015925	protein_coding;pr	6.2547	4.6706	1.34	0.42	3.74E-02	up
CWC22	ENSG0000016351	protein_coding	13.4963	17.0219	0.79	-0.33	3.74E-02	down
PPFIBP2	ENSG0000016638	protein_coding;ret	0.3950	0.5985	0.66	-0.60	3.74E-02	down
FGR	ENSG0000000093	protein_coding;pr	24.6128	19.5344	1.26	0.33	3.74E-02	up
KMT2D	ENSG0000016754	protein_coding;pr	1.8655	1.3801	1.35	0.43	3.75E-02	up
MZT2A	ENSG0000017327	protein_coding;ret	11.6168	8.9166	1.30	0.38	3.76E-02	up
PDPR	ENSG0000009085	retained_intron;pr	8.8350	5.6148	1.57	0.65	3.76E-02	up
TREM1	ENSG0000012473	protein_coding;ret	1.6038	1.0433	1.54	0.62	3.76E-02	up
EGR3	ENSG0000017938	protein_coding;pr	0.3191	0.1716	1.86	0.89	3.77E-02	up
NDUFV2	ENSG0000017812	processed_transcri	48.9763	60.6743	0.81	-0.31	3.77E-02	down
SLC7A5	ENSG0000010325	protein_coding;ret	10.8303	13.8723	0.78	-0.36	3.78E-02	down
GPR82	ENSG0000017165	protein_coding;pr	1.2602	0.7656	1.65	0.72	3.78E-02	up
NCSTN	ENSG0000016273	nonsense_mediate	25.6812	20.4767	1.25	0.33	3.78E-02	up
RSRC2	ENSG0000011101	nonsense_mediate	4.5595	6.1643	0.74	-0.44	3.78E-02	down
AL162458	ENSG0000028579	antisense	0.0896	0.0426	2.10	1.07	3.79E-02	up
IKZF5	ENSG0000009557	protein_coding;pr	5.5670	7.5775	0.73	-0.44	3.80E-02	down
PCF11	ENSG0000016549	protein_coding;TE	8.6604	10.7250	0.81	-0.31	3.81E-02	down
ADAMTS1(	ENSG0000014230	protein_coding;ret	0.4175	0.2371	1.76	0.82	3.83E-02	up
EPDR1	ENSG0000008628	protein_coding	4.4276	2.2109	2.00	1.00	3.83E-02	up
AL049697	ENSG0000021320	processed_transcri	0.4826	0.2006	2.41	1.27	3.84E-02	up
PRSS57	ENSG0000018519	protein_coding	1.7669	0.9720	1.82	0.86	3.85E-02	up
MERTK	ENSG0000015320	protein_coding;ret	1.3715	2.0217	0.68	-0.56	3.85E-02	down
NOC3L	ENSG0000017314	processed_transcri	7.5403	9.6816	0.78	-0.36	3.85E-02	down
AL441992	ENSG0000022347	antisense	3.2745	1.7210	1.90	0.93	3.85E-02	up
PSMC4	ENSG0000001327	protein_coding;ret	30.5883	24.2781	1.26	0.33	3.85E-02	up
UBASH3B	ENSG0000015412	protein_coding;ret	1.7845	2.4446	0.73	-0.45	3.86E-02	down
PIBF1	ENSG0000008353	protein_coding;pr	4.2889	5.8240	0.74	-0.44	3.86E-02	down
AL391832	ENSG0000025808	lincRNA	3.0512	1.7470	1.75	0.80	3.86E-02	up
SLC38A9	ENSG0000017705	protein_coding;ret	1.2940	1.7992	0.72	-0.48	3.87E-02	down
MIS18BP1	ENSG0000012953	nonsense_mediate	26.3108	32.6189	0.81	-0.31	3.87E-02	down
PROK2	ENSG0000016342	protein_coding	16.7157	21.8789	0.76	-0.39	3.88E-02	down
RRN3P1	ENSG0000024812	processed_transcri	1.8908	2.6535	0.71	-0.49	3.88E-02	down
TSPAN14	ENSG0000010821	processed_transcri	3.1361	4.2014	0.75	-0.42	3.88E-02	down
WDR17	ENSG0000015062	protein_coding;pr	0.2149	0.0970	2.22	1.15	3.89E-02	up
TEX15	ENSG0000013386	protein_coding;pr	4.6640	3.6105	1.29	0.37	3.90E-02	up
BCL2	ENSG0000017179	protein_coding;pr	3.9681	5.2955	0.75	-0.42	3.90E-02	down
LPL	ENSG0000017544	protein_coding;pr	28.3320	35.0724	0.81	-0.31	3.91E-02	down
GPX4	ENSG0000016746	protein_coding;ret	40.3717	31.5757	1.28	0.35	3.91E-02	up
GPSM3	ENSG0000021365	nonsense_mediate	35.8716	22.9302	1.56	0.65	3.92E-02	up
PPP1R10	ENSG0000020456	protein_coding;pr	2.5917	3.6002	0.72	-0.47	3.93E-02	down
OSBPL3	ENSG0000007088	protein_coding;no	1.4727	1.0215	1.44	0.53	3.93E-02	up
NAA38	ENSG0000018301	protein_coding;pr	7.3933	5.1809	1.43	0.51	3.94E-02	up
AP006222	ENSG0000022846	processed_transcri	0.3899	0.1960	1.99	0.99	3.95E-02	up

BZW1P2	ENSG0000019840	processed_pseudo;	1.0050	1.8149	0.55	-0.85	3.95E-02	down
WDTC1	ENSG0000014278	protein_coding;no	8.4530	6.5529	1.29	0.37	3.96E-02	up
TMEM63A	ENSG0000019618	protein_coding;pro	1.8258	1.3074	1.40	0.48	3.96E-02	up
CBFA2T3	ENSG0000012999	retained_intron;pr	1.0339	0.6817	1.52	0.60	3.99E-02	up
FOXN3	ENSG0000005325	protein_coding;pro	3.3418	4.2564	0.79	-0.35	4.00E-02	down
AC005332	ENSG0000027747	lincRNA	0.3676	0.1916	1.92	0.94	4.00E-02	up
ELP4	ENSG0000010991	protein_coding;no	0.5443	0.7489	0.73	-0.46	4.03E-02	down
HMGN3	ENSG0000011841	protein_coding	82.0192	65.6213	1.25	0.32	4.04E-02	up
SIDT1	ENSG0000007285	protein_coding;ret	0.0617	0.1523	0.41	-1.30	4.04E-02	down
KCTD12	ENSG0000017869	protein_coding	3.1028	2.2083	1.41	0.49	4.04E-02	up
ZNF548	ENSG0000018878	protein_coding;no	1.1528	0.7237	1.59	0.67	4.05E-02	up
CSTA	ENSG0000012155	protein_coding	116.2882	144.6606	0.80	-0.31	4.05E-02	down
TBC1D10A	ENSG0000009999	protein_coding;no	0.2646	0.1394	1.90	0.92	4.05E-02	up
PLA2G12A	ENSG0000012373	protein_coding;pro	3.4664	2.4577	1.41	0.50	4.06E-02	up
GNPNAT1	ENSG0000010052	protein_coding;ret	14.1372	11.2184	1.26	0.33	4.06E-02	up
LRRC39	ENSG0000012247	protein_coding	0.5959	1.0740	0.55	-0.85	4.08E-02	down
U2SURP	ENSG0000016371	retained_intron;pr	13.4276	16.7046	0.80	-0.32	4.08E-02	down
AGAP4	ENSG0000018823	processed_transcri	0.9774	2.0482	0.48	-1.07	4.08E-02	down
NFAT5	ENSG0000010290	nonsense_mediate	0.9351	1.5616	0.60	-0.74	4.08E-02	down
SLC25A39	ENSG0000001330	retained_intron;pr	16.3427	12.1617	1.34	0.43	4.09E-02	up
ING3	ENSG0000007124	protein_coding;no	3.5919	4.8675	0.74	-0.44	4.09E-02	down
GAS6-AS1	ENSG0000023369	antisense	0.3553	0.5720	0.62	-0.69	4.09E-02	down
IZUMO4	ENSG0000009984	retained_intron;pr	0.2807	0.1386	2.02	1.02	4.09E-02	up
AC005261	ENSG0000026820	lincRNA	3.3426	4.6686	0.72	-0.48	4.09E-02	down
AC073957	ENSG0000027315	antisense	0.5562	0.3138	1.77	0.83	4.10E-02	up
ECE1	ENSG0000011729	protein_coding;ret	0.4679	0.2872	1.63	0.70	4.11E-02	up
SLC7A7	ENSG0000015546	protein_coding;ret	0.9071	1.3978	0.65	-0.62	4.11E-02	down
RN7SL4P	ENSG0000026374	misc_RNA	9.8063	4.0031	2.45	1.29	4.11E-02	up
TEX10	ENSG0000013689	protein_coding;pro	7.5809	9.4884	0.80	-0.32	4.12E-02	down
C15orf39	ENSG0000016717	protein_coding	2.1649	3.1528	0.69	-0.54	4.12E-02	down
USP16	ENSG0000015625	protein_coding;ret	11.3418	14.0519	0.81	-0.31	4.12E-02	down
ZBTB40	ENSG0000018467	protein_coding	4.9251	3.8295	1.29	0.36	4.12E-02	up
SEPSECS-A	ENSG0000028150	antisense	1.9378	1.2133	1.60	0.68	4.12E-02	up
AC126323	ENSG0000016610	transcribed_unpro	0.4776	0.7856	0.61	-0.72	4.13E-02	down
GGPS1	ENSG0000015290	protein_coding;pro	4.2794	6.0027	0.71	-0.49	4.13E-02	down
TYMS	ENSG0000017689	protein_coding;ret	113.8608	91.5580	1.24	0.31	4.14E-02	up
HSD17B6	ENSG0000002542	protein_coding;pro	0.4677	0.2462	1.90	0.93	4.14E-02	up
NDUFA11	ENSG0000017488	protein_coding;ret	19.5722	15.5375	1.26	0.33	4.15E-02	up
HES1	ENSG0000011431	protein_coding;ret	0.5870	0.1946	3.02	1.59	4.15E-02	up
ENO1P4	ENSG0000024179	processed_pseudo;	1.2396	0.5256	2.36	1.24	4.16E-02	up
FKBPL	ENSG0000020431	protein_coding	4.1824	2.6479	1.58	0.66	4.16E-02	up
ZNF7	ENSG0000014778	protein_coding;ret	2.5799	1.2996	1.99	0.99	4.16E-02	up
SPATC1L	ENSG0000016028	protein_coding	3.9934	2.6997	1.48	0.56	4.17E-02	up
CDK2	ENSG0000012337	processed_transcri	14.4769	11.4095	1.27	0.34	4.17E-02	up
BTBD6P1	ENSG0000022910	processed_pseudo;	0.8076	0.4034	2.00	1.00	4.17E-02	up
CHD2	ENSG0000017357	protein_coding;no	3.5512	4.8209	0.74	-0.44	4.19E-02	down
STAT2	ENSG0000017058	retained_intron;pr	6.4578	4.9491	1.30	0.38	4.19E-02	up
APOM	ENSG0000020444	protein_coding	0.7276	1.3571	0.54	-0.90	4.21E-02	down

MINDY1	ENSG0000014340	protein_coding;prc	2.0512	1.3660	1.50	0.59	4.21E-02	up
TONSL	ENSG0000016094	protein_coding;ret	4.3961	3.2316	1.36	0.44	4.22E-02	up
COG2	ENSG0000013577	protein_coding;ret	3.5334	4.4720	0.79	-0.34	4.22E-02	down
H2AFVP1	ENSG0000025874	transcribed_proces	4.2659	2.6078	1.64	0.71	4.23E-02	up
UBAP2L	ENSG0000014356	protein_coding;prc	18.0132	14.4456	1.25	0.32	4.23E-02	up
H3F3B	ENSG0000013247	retained_intron;pr	41.9616	56.4308	0.74	-0.43	4.23E-02	down
HDAC6	ENSG0000009463	protein_coding;no	4.1847	3.2558	1.29	0.36	4.24E-02	up
NQO1	ENSG0000018101	protein_coding;ret	1.2182	1.9884	0.61	-0.71	4.25E-02	down
AC092171	ENSG0000027308	lincRNA	0.9984	1.8404	0.54	-0.88	4.25E-02	down
CDKN2C	ENSG0000012308	protein_coding	14.0162	10.8838	1.29	0.36	4.26E-02	up
RPL7AP6	ENSG0000024207	processed_pseudo;	90.3271	112.4274	0.80	-0.32	4.26E-02	down
SLC28A3	ENSG0000019750	protein_coding;prc	7.9983	6.2694	1.28	0.35	4.26E-02	up
ACAT2	ENSG0000012043	processed_transcri	31.4501	24.6544	1.28	0.35	4.26E-02	up
NPAS1	ENSG0000013075	protein_coding;ret	0.8276	0.4727	1.75	0.81	4.28E-02	up
MIR4435-2I	ENSG0000017296	lincRNA;retained_	0.8440	1.2190	0.69	-0.53	4.29E-02	down
AC022415	ENSG0000028613	nonsense_mediate	0.8039	0.3884	2.07	1.05	4.29E-02	up
UFL1	ENSG0000001412	protein_coding;prc	12.6204	15.8005	0.80	-0.32	4.31E-02	down
TTC7A	ENSG0000006872	retained_intron;pr	2.4936	3.6265	0.69	-0.54	4.32E-02	down
DHX33	ENSG0000000510	protein_coding;no	3.7316	4.9979	0.75	-0.42	4.33E-02	down
RAB37	ENSG0000017279	protein_coding;ret	4.5376	3.2236	1.41	0.49	4.33E-02	up
PLLP	ENSG0000010293	processed_transcri	0.1266	0.2753	0.46	-1.12	4.34E-02	down
EEF2	ENSG0000016765	protein_coding;ret	455.1860	561.6015	0.81	-0.30	4.35E-02	down
ANAPC15	ENSG0000011020	protein_coding;prc	10.2123	7.2029	1.42	0.50	4.35E-02	up
POLR3GL	ENSG0000012185	retained_intron;pr	18.2714	13.8817	1.32	0.40	4.35E-02	up
NBPF10	ENSG0000027142	protein_coding	0.4654	0.7088	0.66	-0.61	4.36E-02	down
AC110285	ENSG0000026287	lincRNA	0.9098	0.4708	1.93	0.95	4.36E-02	up
RTN4RL2	ENSG0000018690	protein_coding	1.0089	0.5631	1.79	0.84	4.37E-02	up
ERCC6L2	ENSG0000018215	nonsense_mediate	1.2386	1.6960	0.73	-0.45	4.37E-02	down
ABCF2	ENSG0000028529	protein_coding	1.8439	3.3544	0.55	-0.86	4.38E-02	down
ERCC6L	ENSG0000018687	protein_coding	5.5500	4.1042	1.35	0.44	4.38E-02	up
HDAC1	ENSG0000011647	protein_coding;prc	41.0151	33.0491	1.24	0.31	4.40E-02	up
COG8	ENSG0000021338	protein_coding;prc	3.3317	4.3700	0.76	-0.39	4.40E-02	down
CCDC142	ENSG0000013563	nonsense_mediate	3.3448	1.6913	1.98	0.98	4.40E-02	up
C12orf45	ENSG0000015113	protein_coding;no	0.4604	0.6843	0.67	-0.57	4.41E-02	down
PLK1	ENSG0000016685	retained_intron;pr	4.6971	6.2970	0.75	-0.42	4.41E-02	down
ADAR	ENSG0000016071	protein_coding;ret	13.1335	9.8027	1.34	0.42	4.42E-02	up
ADGRE4P	ENSG0000026875	retained_intron;pr	7.7619	5.7062	1.36	0.44	4.42E-02	up
MIR22HG	ENSG0000018659	lincRNA	0.2949	0.1316	2.24	1.16	4.43E-02	up
SGPL1	ENSG0000016622	protein_coding;prc	3.1144	2.2915	1.36	0.44	4.44E-02	up
AC087477	ENSG0000025927	lincRNA	0.0759	0.1490	0.51	-0.97	4.45E-02	down
CYB5RL	ENSG0000021588	nonsense_mediate	2.0324	1.3411	1.52	0.60	4.45E-02	up
RPL10P9	ENSG0000023391	processed_pseudo;	229.7073	283.0081	0.81	-0.30	4.45E-02	down
TGFBR1	ENSG0000010679	protein_coding;no	6.9087	11.6939	0.59	-0.76	4.45E-02	down
ZNF696	ENSG0000018573	protein_coding	0.4735	0.2452	1.93	0.95	4.45E-02	up
KIF1BP	ENSG0000019895	protein_coding;ret	3.4082	2.4610	1.38	0.47	4.46E-02	up
NUDT7	ENSG0000014087	processed_transcri	2.9482	4.4133	0.67	-0.58	4.46E-02	down
NDUFB7	ENSG0000009979	protein_coding;no	123.4390	97.1170	1.27	0.35	4.47E-02	up
LAMTOR1	ENSG0000014935	protein_coding;ret	16.4788	12.5063	1.32	0.40	4.47E-02	up

HECA	ENSG0000011240	protein_coding	4.7222	6.1724	0.77	-0.39	4.48E-02	down
HYOU1	ENSG0000014942	protein_coding;no	11.3637	9.0704	1.25	0.33	4.48E-02	up
ADH5	ENSG0000019789	protein_coding;no	18.3940	14.7822	1.24	0.32	4.48E-02	up
XYLT1	ENSG0000010348	protein_coding;ret	2.2559	2.9935	0.75	-0.41	4.49E-02	down
RESF1	ENSG0000017471	processed_transcri	9.6829	7.0842	1.37	0.45	4.49E-02	up
GTF2F2	ENSG0000018834	protein_coding;pro	11.9136	14.8614	0.80	-0.32	4.49E-02	down
NCF4	ENSG0000010036	protein_coding;pro	136.5790	110.0311	1.24	0.31	4.50E-02	up
INSIG2	ENSG0000012562	processed_transcri	2.7255	1.5746	1.73	0.79	4.50E-02	up
ACSS3	ENSG0000011105	processed_transcri	0.4143	0.2707	1.53	0.61	4.51E-02	up
CHD8	ENSG0000010088	protein_coding;ret	2.8597	2.2918	1.25	0.32	4.52E-02	up
UTP6	ENSG0000010865	retained_intron;pro	13.7148	16.7931	0.82	-0.29	4.52E-02	down
SDHAP1	ENSG0000018548	processed_transcri	2.9214	2.0085	1.45	0.54	4.52E-02	up
PPAN-P2RY	ENSG0000024320	protein_coding	1.2317	0.4339	2.84	1.51	4.52E-02	up
PTP4A1	ENSG0000011224	protein_coding;pro	11.7998	14.5377	0.81	-0.30	4.53E-02	down
ADCY9	ENSG0000016210	protein_coding;pro	0.5565	0.8087	0.69	-0.54	4.53E-02	down
RASAL1	ENSG0000011134	processed_transcri	2.9348	2.0990	1.40	0.48	4.54E-02	up
AC027020	ENSG0000027012	lincRNA	0.3701	0.1742	2.12	1.09	4.54E-02	up
NR4A1	ENSG0000012335	retained_intron;pro	0.1213	0.2173	0.56	-0.84	4.55E-02	down
TFDP1	ENSG0000019817	nonsense_mediate	58.7336	47.3898	1.24	0.31	4.55E-02	up
MYO9B	ENSG0000009933	protein_coding;ret	5.4476	4.2378	1.29	0.36	4.55E-02	up
IRF7	ENSG0000018550	protein_coding;ret	0.5774	0.3130	1.84	0.88	4.55E-02	up
VASH1-AS1	ENSG0000025830	lincRNA	0.4580	0.8822	0.52	-0.95	4.55E-02	down
ITGA9	ENSG0000014466	protein_coding;ret	0.4356	0.2647	1.65	0.72	4.56E-02	up
PUDP	ENSG0000013002	protein_coding;pro	5.0704	3.6418	1.39	0.48	4.56E-02	up
GOLGA2	ENSG0000016711	protein_coding;ret	5.2650	4.0688	1.29	0.37	4.56E-02	up
AL671277	ENSG0000022776	unprocessed_pseu	1.9443	1.1137	1.75	0.80	4.58E-02	up
TKFC	ENSG0000014947	retained_intron;pro	0.7882	1.1556	0.68	-0.55	4.58E-02	down
LSM4	ENSG0000013052	protein_coding;ret	21.6637	31.5861	0.69	-0.54	4.58E-02	down
TP53TG1	ENSG0000018216	lincRNA	1.2225	0.6394	1.91	0.93	4.59E-02	up
AL080250	ENSG0000022579	lincRNA	1.4297	2.2518	0.63	-0.66	4.60E-02	down
SPN	ENSG0000019747	protein_coding;no	19.2249	27.4346	0.70	-0.51	4.60E-02	down
GTF2IRD2	ENSG0000019627	protein_coding;ret	0.7737	0.4771	1.62	0.70	4.61E-02	up
DEFA1B	ENSG0000024024	protein_coding	1536.4421	1909.6260	0.80	-0.31	4.61E-02	down
KDM6B	ENSG0000013251	protein_coding;pro	1.3061	0.8735	1.50	0.58	4.62E-02	up
CD55	ENSG0000019635	protein_coding;ret	16.6409	13.3792	1.24	0.31	4.62E-02	up
CICP14	ENSG0000028149	processed_pseudo	1.4436	0.8955	1.61	0.69	4.62E-02	up
PFDN4	ENSG0000010113	protein_coding;no	12.0674	15.9518	0.76	-0.40	4.64E-02	down
HPGDS	ENSG0000016310	protein_coding;ret	1.7956	1.1216	1.60	0.68	4.64E-02	up
PPP1R1B	ENSG0000013177	protein_coding;ret	1.0320	0.6619	1.56	0.64	4.64E-02	up
C8orf59	ENSG0000017673	protein_coding;ret	18.5365	23.1081	0.80	-0.32	4.65E-02	down
GPD2	ENSG0000011515	protein_coding;no	11.7060	9.4135	1.24	0.31	4.65E-02	up
ANKRD18A	ENSG0000018007	protein_coding;pro	1.8024	2.5207	0.72	-0.48	4.66E-02	down
SHMT1	ENSG0000017697	retained_intron;pro	2.2322	1.6010	1.39	0.48	4.66E-02	up
SFI1	ENSG0000019808	protein_coding;ret	1.8520	1.3548	1.37	0.45	4.67E-02	up
AMFR	ENSG0000015946	protein_coding;ret	5.1325	3.6983	1.39	0.47	4.67E-02	up
SUN1	ENSG0000016482	retained_intron;pro	3.5077	2.7646	1.27	0.34	4.68E-02	up
BISPR	ENSG0000028285	lincRNA	1.0945	1.8641	0.59	-0.77	4.69E-02	down
IQSEC1	ENSG0000014471	protein_coding;pro	0.3055	0.1794	1.70	0.77	4.69E-02	up

NUP43	ENSG0000012025	protein_coding;pr	13.2802	16.5114	0.80	-0.31	4.69E-02	down
ZSCAN18	ENSG0000012141	protein_coding;ret	1.0504	0.6344	1.66	0.73	4.70E-02	up
LAT2	ENSG0000008673	protein_coding;ret	34.4541	43.5657	0.79	-0.34	4.70E-02	down
PSME3	ENSG0000013146	protein_coding;ret	15.9027	9.9602	1.60	0.68	4.71E-02	up
WDR12	ENSG0000013844	protein_coding;ret	10.1064	12.3409	0.82	-0.29	4.72E-02	down
RPSAP12	ENSG0000024008	processed_pseudo	28.3471	16.3410	1.73	0.79	4.73E-02	up
ZNF517	ENSG0000019736	protein_coding;no	0.4804	0.2437	1.97	0.98	4.73E-02	up
STAP1	ENSG0000003572	protein_coding	3.6362	2.3233	1.57	0.65	4.73E-02	up
ACTA2	ENSG0000010779	protein_coding;pr	0.2845	0.1493	1.91	0.93	4.75E-02	up
CNTF	ENSG0000024268	protein_coding	0.3868	0.6976	0.55	-0.85	4.75E-02	down
STK38	ENSG0000011207	protein_coding	11.8464	9.3564	1.27	0.34	4.76E-02	up
AC008554	ENSG0000023744	protein_coding;no	1.3979	0.8250	1.69	0.76	4.76E-02	up
SLC27A2	ENSG0000014028	protein_coding;ret	13.7138	10.7003	1.28	0.36	4.76E-02	up
STAT5B	ENSG0000017375	protein_coding;ret	6.8119	8.4771	0.80	-0.32	4.77E-02	down
SPRTN	ENSG0000001007	protein_coding;pr	1.2901	0.7412	1.74	0.80	4.77E-02	up
GOLGA2P5	ENSG0000023810	retained_intron;pr	1.2646	0.8491	1.49	0.57	4.78E-02	up
PELO	ENSG0000015268	protein_coding;pr	6.2198	4.6770	1.33	0.41	4.79E-02	up
SERINC5	ENSG0000016430	protein_coding;no	1.6452	2.2216	0.74	-0.43	4.80E-02	down
RPS27AP16	ENSG0000022463	transcribed_proce	68.5635	90.2022	0.76	-0.40	4.80E-02	down
VEGFA	ENSG0000011271	retained_intron;pr	4.9139	6.0544	0.81	-0.30	4.80E-02	down
PARP14	ENSG0000017319	retained_intron;pr	1.1773	1.6213	0.73	-0.46	4.81E-02	down
TSC22D3	ENSG0000015751	protein_coding	6.6621	8.4535	0.79	-0.34	4.82E-02	down
SLC39A1	ENSG0000014357	protein_coding;pr	16.3267	12.5310	1.30	0.38	4.82E-02	up
C17orf49	ENSG0000025831	retained_intron;pr	0.7095	1.1801	0.60	-0.73	4.82E-02	down
CCDC88B	ENSG0000016807	processed_transcri	4.8295	3.7923	1.27	0.35	4.83E-02	up
NCOA7	ENSG0000011191	protein_coding;pr	4.0947	5.1949	0.79	-0.34	4.83E-02	down
CCNG2	ENSG0000013876	protein_coding;pr	2.3859	1.7167	1.39	0.47	4.83E-02	up
FBXO6	ENSG0000011666	protein_coding;ret	6.2114	4.3320	1.43	0.52	4.85E-02	up
AC080038	ENSG0000001102	retained_intron;pr	6.1317	4.8157	1.27	0.35	4.86E-02	up
LIX1L	ENSG0000027160	protein_coding	3.2553	4.4486	0.73	-0.45	4.89E-02	down
LINC00665	ENSG0000023267	lincRNA	3.6281	2.7268	1.33	0.41	4.89E-02	up
MRPL20-AS	ENSG0000022487	antisense;retained_	1.4639	2.8126	0.52	-0.94	4.89E-02	down
H2AFZ	ENSG0000016403	processed_transcri	225.0063	170.8914	1.32	0.40	4.90E-02	up
PLCB1	ENSG0000018262	protein_coding;pr	0.1419	0.2411	0.59	-0.76	4.91E-02	down
AC000120	ENSG0000028595	protein_coding	0.2290	0.9998	0.23	-2.13	4.91E-02	down
CC2D1A	ENSG0000013202	protein_coding;ret	3.1248	2.2616	1.38	0.47	4.91E-02	up
CIB1	ENSG0000018504	protein_coding	36.7635	45.7966	0.80	-0.32	4.91E-02	down
TPRKB	ENSG0000014403	protein_coding;ret	14.3122	18.2375	0.78	-0.35	4.91E-02	down
CCDC78	ENSG0000016200	retained_intron;pr	0.2420	0.4241	0.57	-0.81	4.91E-02	down
QSER1	ENSG0000006074	protein_coding;ret	2.2728	3.0047	0.76	-0.40	4.92E-02	down
TRERF1	ENSG0000012449	protein_coding	5.3501	6.6905	0.80	-0.32	4.92E-02	down
DIAPH2	ENSG0000014720	protein_coding	3.2759	4.2689	0.77	-0.38	4.92E-02	down
DENND5B	ENSG0000017045	protein_coding;no	0.4161	0.2725	1.53	0.61	4.92E-02	up
CELSR1	ENSG0000007527	protein_coding;pr	0.0519	0.0969	0.54	-0.90	4.92E-02	down
FBXL4	ENSG0000011223	protein_coding	3.9171	4.9459	0.79	-0.34	4.93E-02	down
AC104794	ENSG0000026007	lincRNA	3.0571	2.0049	1.52	0.61	4.93E-02	up
EIF2S3	ENSG0000013074	processed_transcri	77.4946	95.9711	0.81	-0.31	4.94E-02	down
TBX19	ENSG0000014317	processed_transcri	0.3925	0.6856	0.57	-0.80	4.94E-02	down

IPO7	ENSG0000020533	nonsense_mediate	44.6892	54.8809	0.81	-0.30	4.94E-02	down
TSN	ENSG0000021146	nonsense_mediate	9.5255	14.3725	0.66	-0.59	4.94E-02	down
MIR222HG	ENSG0000027006	lincRNA	3.1147	4.3513	0.72	-0.48	4.95E-02	down
GOLGA4	ENSG0000014467	protein_coding;pro	2.8785	4.1600	0.69	-0.53	4.95E-02	down
SSR3	ENSG0000011485	retained_intron;pr	30.8785	37.8332	0.82	-0.29	4.95E-02	down
CREB3	ENSG0000010717	protein_coding;pro	10.8169	7.8811	1.37	0.46	4.96E-02	up
FAM219B	ENSG0000017876	retained_intron;pr	5.0137	2.9021	1.73	0.79	4.96E-02	up
PIM2	ENSG0000010209	protein_coding;pro	3.1807	4.4749	0.71	-0.49	4.97E-02	down
SLC35F1	ENSG0000019637	protein_coding	0.1084	0.2137	0.51	-0.98	4.97E-02	down
EXOC2	ENSG0000011268	protein_coding;pro	6.2607	4.7467	1.32	0.40	4.97E-02	up
QARS	ENSG0000017205	retained_intron;no	37.3670	46.0917	0.81	-0.30	4.98E-02	down
BTBD6	ENSG0000018488	retained_intron;pr	8.2477	6.0189	1.37	0.45	4.98E-02	up
FAM110A	ENSG0000012589	protein_coding	1.1011	0.6821	1.61	0.69	4.99E-02	up
LINC00115	ENSG0000022588	lincRNA	1.9529	1.1554	1.69	0.76	4.99E-02	up
MPHOSPH1	ENSG0000012438	protein_coding;ret	10.6779	13.5756	0.79	-0.35	5.00E-02	down
DOT1L	ENSG0000010488	protein_coding;pro	3.0193	2.2851	1.32	0.40	5.00E-02	up

Supplementary Table 4. GO enrichment analysis of differentially expressed genes.

GO_ID	GO_Term	GO_function	GO_class	S.gene.number	TS.gene.number	B.gene.number	TB.gene.number	pvalue
GO:0019221	cytokine-mediated signaling pa	BP	6	27	562	295	19809	9.91E-08
GO:0009986	cell surface	CC	4	42	562	623	19809	2.17E-07
GO:0006955	immune response	BP	4	36	562	494	19809	2.57E-07
GO:0050729	positive regulation of inflamm	BP	9	13	562	85	19809	7.66E-07
GO:0009897	external side of plasma membr	CC	5	28	562	357	19809	1.35E-06
GO:0006954	inflammatory response	BP	6	28	562	382	19809	5.00E-06
GO:0071276	cellular response to cadmium i	BP	8	8	562	37	19809	7.46E-06
GO:0007155	cell adhesion	BP	4	38	562	631	19809	1.17E-05
GO:0030198	extracellular matrix organizati	BP	6	20	562	235	19809	1.34E-05
GO:0097191	extrinsic apoptotic signaling pa	BP	6	8	562	45	19809	3.41E-05
GO:0062023	collagen-containing extracellu	CC	5	25	562	361	19809	4.11E-05
GO:0005886	plasma membrane	CC	4	182	562	5001	19809	7.01E-05
GO:0036018	cellular response to erythropoi	BP	8	3	562	4	19809	8.89E-05
GO:2000391	positive regulation of neutroph	BP	9	3	562	4	19809	8.89E-05
GO:0050901	leukocyte tethering or rolling	BP	8	5	562	18	19809	1.14E-04
GO:0043068	positive regulation of program	BP	8	4	562	10	19809	1.17E-04
GO:0000790	nuclear chromatin	CC	7	18	562	235	19809	1.39E-04
GO:0004896	cytokine receptor activity	MF	6	8	562	55	19809	1.50E-04
GO:0001525	angiogenesis	BP	5	19	562	258	19809	1.53E-04
GO:0002376	immune system process	BP	3	39	562	740	19809	1.64E-04
GO:0030728	ovulation	BP	5	4	562	11	19809	1.80E-04
GO:0002576	platelet degranulation	BP	9	12	562	122	19809	1.84E-04
GO:0019955	cytokine binding	MF	5	7	562	43	19809	1.89E-04
GO:0043235	receptor complex	CC	4	16	562	204	19809	2.46E-04
GO:0071711	basement membrane organizat	BP	7	4	562	12	19809	2.65E-04
GO:0043312	neutrophil degranulation	BP	9	28	562	481	19809	2.87E-04
GO:0007267	cell-cell signaling	BP	5	17	562	231	19809	3.38E-04
GO:0005887	integral component of plasma	CC	6	61	562	1378	19809	3.63E-04
GO:0070374	positive regulation of ERK1 a	BP	12	16	562	213	19809	4.00E-04
GO:1901166	neural crest cell migration inv	BP	9	3	562	6	19809	4.26E-04
GO:0061312	BMP signaling pathway involv	BP	11	3	562	6	19809	4.26E-04
GO:0010273	detoxification of copper ion	BP	5	4	562	14	19809	5.11E-04
GO:0010955	negative regulation of protein	BP	10	4	562	14	19809	5.11E-04
GO:0071773	cellular response to BMP stim	BP	8	6	562	37	19809	5.58E-04
GO:0005615	extracellular space	CC	4	75	562	1812	19809	5.64E-04
GO:0030667	secretory granule membrane	CC	7	10	562	101	19809	5.93E-04
GO:0043394	proteoglycan binding	MF	5	4	562	15	19809	6.82E-04
GO:0033540	fatty acid beta-oxidation using	BP	12	4	562	15	19809	6.82E-04
GO:0016021	integral component of membra	CC	5	192	562	5545	19809	6.82E-04
GO:0071280	cellular response to copper ion	BP	8	5	562	26	19809	7.25E-04
GO:0004908	interleukin-1 receptor activity	MF	7	3	562	7	19809	7.30E-04
GO:0090210	regulation of establishment of	BP	8	2	562	2	19809	8.04E-04
GO:0047017	prostaglandin-F synthase activ	MF	7	2	562	2	19809	8.04E-04
GO:0010725	regulation of primitive erythro	BP	9	2	562	2	19809	8.04E-04
GO:0070488	neutrophil aggregation	BP	8	2	562	2	19809	8.04E-04
GO:0035854	eosinophil fate commitment	BP	6	2	562	2	19809	8.04E-04

GO:1904835	dorsal root ganglion morphoge	BP	7	2	562	2	19809	8.04E-04
GO:0004339	glucan 1,4-alpha-glucosidase a	MF	8	2	562	2	19809	8.04E-04
GO:0097154	GABAergic neuron differentia	BP	7	2	562	2	19809	8.04E-04
GO:0017116	single-stranded DNA-depende	MF	12	2	562	2	19809	8.04E-04
GO:0021649	vestibulocochlear nerve structu	BP	6	2	562	2	19809	8.04E-04
GO:0051493	regulation of cytoskeleton orga	BP	8	5	562	27	19809	8.69E-04
GO:0071407	cellular response to organic cy	BP	7	8	562	71	19809	8.84E-04
GO:0034113	heterotypic cell-cell adhesion	BP	6	5	562	28	19809	1.03E-03
GO:0045121	membrane raft	CC	6	16	562	233	19809	1.05E-03
GO:0045766	positive regulation of angiogen	BP	8	12	562	148	19809	1.07E-03
GO:0007566	embryo implantation	BP	4	6	562	42	19809	1.12E-03
GO:0031092	platelet alpha granule membrar	CC	8	4	562	17	19809	1.14E-03
GO:0061626	pharyngeal arch artery morpho	BP	8	3	562	8	19809	1.14E-03
GO:0031666	positive regulation of lipopoly	BP	8	3	562	8	19809	1.14E-03
GO:0005577	fibrinogen complex	CC	4	3	562	8	19809	1.14E-03
GO:0008285	negative regulation of cell pop	BP	7	25	562	450	19809	1.16E-03
GO:0021983	pituitary gland development	BP	7	5	562	29	19809	1.22E-03
GO:0008305	integrin complex	CC	5	5	562	29	19809	1.22E-03
GO:0050727	regulation of inflammatory res	BP	8	8	562	75	19809	1.27E-03
GO:0045926	negative regulation of growth	BP	6	4	562	18	19809	1.43E-03
GO:0045648	positive regulation of erythroc	BP	9	5	562	30	19809	1.43E-03
GO:0043025	neuronal cell body	CC	5	23	562	407	19809	1.44E-03
GO:0101003	ficolin-1-rich granule membrar	CC	8	7	562	60	19809	1.50E-03
GO:0009611	response to wounding	BP	5	8	562	77	19809	1.51E-03
GO:0051897	positive regulation of protein k	BP	10	13	562	177	19809	1.67E-03
GO:0002523	leukocyte migration involved i	BP	5	3	562	9	19809	1.68E-03
GO:0021612	facial nerve structural organiza	BP	6	3	562	9	19809	1.68E-03
GO:0014049	positive regulation of glutamat	BP	10	3	562	9	19809	1.68E-03
GO:0005102	signaling receptor binding	MF	5	23	562	413	19809	1.74E-03
GO:0050718	positive regulation of interleuk	BP	10	5	562	32	19809	1.93E-03
GO:0007166	cell surface receptor signaling	BP	5	17	562	271	19809	1.99E-03
GO:1904707	positive regulation of vascular	BP	9	6	562	47	19809	2.03E-03
GO:0032526	response to retinoic acid	BP	6	6	562	47	19809	2.03E-03
GO:0008201	heparin binding	MF	6	12	562	161	19809	2.19E-03
GO:0052650	NADP-retinol dehydrogenase a	MF	9	3	562	10	19809	2.35E-03
GO:0071322	cellular response to carbohydra	BP	7	2	562	3	19809	2.37E-03
GO:0021828	gonadotrophin-releasing horm	BP	8	2	562	3	19809	2.37E-03
GO:0032301	MutSalpha complex	CC	6	2	562	3	19809	2.37E-03
GO:0004165	dodecenoyl-CoA delta-isomera	MF	7	2	562	3	19809	2.37E-03
GO:1903375	facioacoustic ganglion develop	BP	8	2	562	3	19809	2.37E-03
GO:0032602	chemokine production	BP	5	2	562	3	19809	2.37E-03
GO:1905517	macrophage migration	BP	6	2	562	3	19809	2.37E-03
GO:0097374	sensory neuron axon guidance	BP	7	2	562	3	19809	2.37E-03
GO:0071415	cellular response to purine-con	BP	8	2	562	3	19809	2.37E-03
GO:0010574	regulation of vascular endothe	BP	7	2	562	3	19809	2.37E-03
GO:0021533	cell differentiation in hindbrai	BP	6	2	562	3	19809	2.37E-03
GO:0003779	actin binding	MF	6	21	562	374	19809	2.46E-03
GO:0005576	extracellular region	CC	3	91	562	2407	19809	2.49E-03
GO:0019838	growth factor binding	MF	5	6	562	49	19809	2.52E-03
GO:0030425	dendrite	CC	6	24	562	455	19809	2.84E-03

GO:0060333	interferon-gamma-mediated si	BP	7	7	562	67	19809	2.85E-03
GO:0001707	mesoderm formation	BP	6	5	562	35	19809	2.90E-03
GO:0001975	response to amphetamine	BP	6	5	562	35	19809	2.90E-03
GO:0045765	regulation of angiogenesis	BP	7	5	562	35	19809	2.90E-03
GO:0008360	regulation of cell shape	BP	8	11	562	146	19809	3.03E-03
GO:0030424	axon	CC	6	21	562	381	19809	3.05E-03
GO:0071294	cellular response to zinc ion	BP	8	4	562	22	19809	3.12E-03
GO:0017154	semaphorin receptor activity	MF	6	3	562	11	19809	3.16E-03
GO:0008329	signaling pattern recognition re	MF	6	3	562	11	19809	3.16E-03
GO:0071803	positive regulation of podoson	BP	8	3	562	11	19809	3.16E-03
GO:1904996	positive regulation of leukocyt	BP	9	3	562	11	19809	3.16E-03
GO:2001241	positive regulation of extrinsic	BP	11	3	562	11	19809	3.16E-03
GO:0002116	semaphorin receptor complex	CC	5	3	562	11	19809	3.16E-03
GO:0050786	RAGE receptor binding	MF	6	3	562	11	19809	3.16E-03
GO:0030154	cell differentiation	BP	5	42	562	952	19809	3.18E-03
GO:0001649	osteoblast differentiation	BP	6	9	562	106	19809	3.20E-03
GO:0010595	positive regulation of endothel	BP	10	6	562	52	19809	3.42E-03
GO:0030165	PDZ domain binding	MF	6	8	562	88	19809	3.51E-03
GO:0009617	response to bacterium	BP	7	9	562	108	19809	3.63E-03
GO:0008015	blood circulation	BP	6	5	562	37	19809	3.72E-03
GO:0048469	cell maturation	BP	5	5	562	37	19809	3.72E-03
GO:0005178	integrin binding	MF	6	10	562	129	19809	3.76E-03
GO:0030168	platelet activation	BP	5	9	562	109	19809	3.86E-03
GO:0070098	chemokine-mediated signaling	BP	7	7	562	71	19809	3.95E-03
GO:1990909	Wnt signalosome	CC	4	3	562	12	19809	4.13E-03
GO:0002430	complement receptor mediated	BP	7	3	562	12	19809	4.13E-03
GO:2000300	regulation of synaptic vesicle e	BP	7	6	562	54	19809	4.13E-03
GO:0030501	positive regulation of bone mi	BP	8	5	562	38	19809	4.18E-03
GO:0005201	extracellular matrix structural	MF	4	10	562	131	19809	4.20E-03
GO:1903561	extracellular vesicle	CC	6	6	562	55	19809	4.53E-03
GO:0001701	in utero embryonic developme	BP	8	13	562	199	19809	4.59E-03
GO:0097490	sympathetic neuron projection	BP	7	2	562	4	19809	4.64E-03
GO:0097491	sympathetic neuron projection	BP	6	2	562	4	19809	4.64E-03
GO:0001839	neural plate morphogenesis	BP	8	2	562	4	19809	4.64E-03
GO:1902285	semaphorin-plexin signaling pa	BP	7	2	562	4	19809	4.64E-03
GO:0051599	response to hydrostatic pressu	BP	5	2	562	4	19809	4.64E-03
GO:0032280	symmetric synapse	CC	5	2	562	4	19809	4.64E-03
GO:0035662	Toll-like receptor 4 binding	MF	7	2	562	4	19809	4.64E-03
GO:0036486	ventral trunk neural crest cell r	BP	10	2	562	4	19809	4.64E-03
GO:0045123	cellular extravasation	BP	5	2	562	4	19809	4.64E-03
GO:0036438	maintenance of lens transparer	BP	8	2	562	4	19809	4.64E-03
GO:0001845	phagolysosome assembly	BP	9	2	562	4	19809	4.64E-03
GO:0002724	regulation of T cell cytokine p	BP	10	2	562	4	19809	4.64E-03
GO:0032010	phagolysosome	CC	10	2	562	4	19809	4.64E-03
GO:0061309	cardiac neural crest cell develo	BP	9	2	562	4	19809	4.64E-03
GO:0014054	positive regulation of gamma-a	BP	9	2	562	4	19809	4.64E-03
GO:0032119	sequestering of zinc ion	BP	6	2	562	4	19809	4.64E-03
GO:0002667	regulation of T cell anergy	BP	8	2	562	4	19809	4.64E-03
GO:0044752	response to human chorionic g	BP	7	2	562	4	19809	4.64E-03
GO:2001198	regulation of dendritic cell dif	BP	8	2	562	4	19809	4.64E-03

GO:0099626	voltage-gated calcium channel	MF	11	2	562	4	19809	4.64E-03
GO:0003266	regulation of secondary heart f	BP	10	2	562	4	19809	4.64E-03
GO:0010469	regulation of signaling recepto	BP	8	23	562	450	19809	5.02E-03
GO:0051894	positive regulation of focal adh	BP	9	4	562	25	19809	5.05E-03
GO:0021854	hypothalamus development	BP	5	3	562	13	19809	5.25E-03
GO:0035641	locomotory exploration behavi	BP	5	3	562	13	19809	5.25E-03
GO:0090162	establishment of epithelial cell	BP	6	3	562	13	19809	5.25E-03
GO:0030278	regulation of ossification	BP	6	3	562	13	19809	5.25E-03
GO:0007229	integrin-mediated signaling pa	BP	6	8	562	94	19809	5.26E-03
GO:0019233	sensory perception of pain	BP	7	6	562	57	19809	5.41E-03
GO:0034599	cellular response to oxidative s	BP	6	8	562	95	19809	5.60E-03
GO:0016209	antioxidant activity	MF	3	4	562	26	19809	5.83E-03
GO:0001756	somitogenesis	BP	7	6	562	58	19809	5.89E-03
GO:0071346	cellular response to interferon-	BP	8	8	562	96	19809	5.96E-03
GO:0007568	aging	BP	4	12	562	183	19809	6.15E-03
GO:0070062	extracellular exosome	CC	7	80	562	2142	19809	6.26E-03
GO:0005925	focal adhesion	CC	7	21	562	407	19809	6.45E-03
GO:0099509	regulation of presynaptic cytos	BP	13	3	562	14	19809	6.55E-03
GO:0030502	negative regulation of bone mi	BP	8	3	562	14	19809	6.55E-03
GO:0050710	negative regulation of cytokine	BP	8	3	562	14	19809	6.55E-03
GO:0045087	innate immune response	BP	5	25	562	514	19809	6.57E-03
GO:0030246	carbohydrate binding	MF	4	13	562	210	19809	7.14E-03
GO:0008270	zinc ion binding	MF	8	36	562	825	19809	7.20E-03
GO:0042803	protein homodimerization acti	MF	6	37	562	855	19809	7.37E-03
GO:0002693	positive regulation of cellular e	BP	8	2	562	5	19809	7.59E-03
GO:0047023	androsterone dehydrogenase ac	MF	7	2	562	5	19809	7.59E-03
GO:0002925	positive regulation of humoral	BP	12	2	562	5	19809	7.59E-03
GO:0071621	granulocyte chemotaxis	BP	6	2	562	5	19809	7.59E-03
GO:0048385	regulation of retinoic acid rece	BP	8	2	562	5	19809	7.59E-03
GO:0048318	axial mesoderm development	BP	7	2	562	5	19809	7.59E-03
GO:0031726	CCR1 chemokine receptor bin	MF	9	2	562	5	19809	7.59E-03
GO:2000321	positive regulation of T-helper	BP	9	2	562	5	19809	7.59E-03
GO:0061551	trigeminal ganglion developme	BP	8	2	562	5	19809	7.59E-03
GO:0050544	arachidonic acid binding	MF	8	2	562	5	19809	7.59E-03
GO:0032783	ELL-EAF complex	CC	5	2	562	5	19809	7.59E-03
GO:0050798	activated T cell proliferation	BP	9	2	562	5	19809	7.59E-03
GO:0021637	trigeminal nerve structural org	BP	6	2	562	5	19809	7.59E-03
GO:0070051	fibrinogen binding	MF	5	2	562	5	19809	7.59E-03
GO:0034103	regulation of tissue remodeling	BP	6	2	562	5	19809	7.59E-03
GO:0007044	cell-substrate junction assembl	BP	7	2	562	5	19809	7.59E-03
GO:0051495	positive regulation of cytoskel	BP	9	2	562	5	19809	7.59E-03
GO:0045345	positive regulation of MHC cla	BP	9	2	562	5	19809	7.59E-03
GO:0050728	negative regulation of inflamm	BP	9	9	562	121	19809	7.61E-03
GO:0006882	cellular zinc ion homeostasis	BP	12	4	562	28	19809	7.64E-03
GO:0001569	branching involved in blood ve	BP	7	4	562	28	19809	7.64E-03
GO:0051602	response to electrical stimulus	BP	5	4	562	28	19809	7.64E-03
GO:2001234	negative regulation of apoptoti	BP	9	4	562	28	19809	7.64E-03
GO:0043547	positive regulation of GTPase	BP	8	18	562	336	19809	7.76E-03
GO:0030036	actin cytoskeleton organization	BP	7	11	562	166	19809	7.90E-03
GO:0031091	platelet alpha granule	CC	9	3	562	15	19809	8.01E-03

GO:0045028	G protein-coupled purinergic r	MF	8	3	562	15	19809	8.01E-03
GO:0035589	G protein-coupled purinergic r	BP	7	3	562	15	19809	8.01E-03
GO:0071560	cellular response to transformi	BP	8	6	562	62	19809	8.14E-03
GO:0071944	cell periphery	CC	4	6	562	62	19809	8.14E-03
GO:0000187	activation of MAPK activity	BP	13	9	562	123	19809	8.44E-03
GO:0007517	muscle organ development	BP	6	8	562	102	19809	8.52E-03
GO:0030593	neutrophil chemotaxis	BP	7	7	562	82	19809	8.66E-03
GO:0035987	endodermal cell differentiation	BP	6	4	562	29	19809	8.67E-03
GO:0009968	negative regulation of signal tr	BP	8	6	562	64	19809	9.47E-03
GO:0007399	nervous system development	BP	6	25	562	530	19809	9.48E-03
GO:0010718	positive regulation of epithelia	BP	8	5	562	46	19809	9.49E-03
GO:0051044	positive regulation of membrai	BP	9	3	562	16	19809	9.66E-03
GO:0048485	sympathetic nervous system de	BP	6	3	562	16	19809	9.66E-03
GO:0038084	vascular endothelial growth fa	BP	8	3	562	16	19809	9.66E-03
GO:0007492	endoderm development	BP	6	4	562	30	19809	9.78E-03
GO:0050832	defense response to fungus	BP	8	4	562	30	19809	9.78E-03
GO:0071456	cellular response to hypoxia	BP	6	9	562	126	19809	9.81E-03
GO:0007275	multicellular organism develop	BP	4	46	562	1138	19809	1.01E-02
GO:0005518	collagen binding	MF	5	6	562	65	19809	1.02E-02
GO:0060337	type I interferon signaling path	BP	7	6	562	65	19809	1.02E-02
GO:0006919	activation of cysteine-type end	BP	11	7	562	85	19809	1.05E-02
GO:0043388	positive regulation of DNA bi	BP	7	4	562	31	19809	1.10E-02
GO:0048873	homeostasis of number of cells	BP	8	4	562	31	19809	1.10E-02
GO:0050840	extracellular matrix binding	MF	4	4	562	31	19809	1.10E-02
GO:0019966	interleukin-1 binding	MF	6	2	562	6	19809	1.12E-02
GO:0016671	oxidoreductase activity, acting	MF	6	2	562	6	19809	1.12E-02
GO:0060385	axonogenesis involved in inne	BP	10	2	562	6	19809	1.12E-02
GO:0098885	modification of postsynaptic a	BP	8	2	562	6	19809	1.12E-02
GO:0031630	regulation of synaptic vesicle f	BP	9	2	562	6	19809	1.12E-02
GO:0016004	phospholipase activator activit	MF	7	2	562	6	19809	1.12E-02
GO:0004630	phospholipase D activity	MF	8	2	562	6	19809	1.12E-02
GO:0031670	cellular response to nutrient	BP	8	2	562	6	19809	1.12E-02
GO:0047035	testosterone dehydrogenase (N	MF	7	2	562	6	19809	1.12E-02
GO:0048505	regulation of timing of cell dif	BP	7	2	562	6	19809	1.12E-02
GO:0043141	ATP-dependent 5'-3' DNA heli	MF	12	2	562	6	19809	1.12E-02
GO:0021785	branchiomotor neuron axon gu	BP	8	2	562	6	19809	1.12E-02
GO:0043619	regulation of transcription fror	BP	11	2	562	6	19809	1.12E-02
GO:0010757	negative regulation of plasmin	BP	11	2	562	6	19809	1.12E-02
GO:0042415	norepinephrine metabolic proc	BP	9	2	562	6	19809	1.12E-02
GO:1904628	cellular response to phorbol 13	BP	7	2	562	6	19809	1.12E-02
GO:0032962	positive regulation of inositol t	BP	11	2	562	6	19809	1.12E-02
GO:0032924	activin receptor signaling path	BP	8	3	562	17	19809	1.15E-02
GO:0050995	negative regulation of lipid cat	BP	8	3	562	17	19809	1.15E-02
GO:0030224	monocyte differentiation	BP	8	3	562	17	19809	1.15E-02
GO:0031093	platelet alpha granule lumen	CC	8	6	562	67	19809	1.17E-02
GO:0006909	phagocytosis	BP	7	6	562	67	19809	1.17E-02
GO:0001934	positive regulation of protein p	BP	10	11	562	176	19809	1.19E-02
GO:0005164	tumor necrosis factor receptor	MF	8	4	562	32	19809	1.23E-02
GO:0042113	B cell activation	BP	7	4	562	32	19809	1.23E-02
GO:0032355	response to estradiol	BP	6	8	562	109	19809	1.24E-02

GO:0009612	response to mechanical stimulu	BP	5	6	562	68	19809	1.26E-02
GO:0043195	terminal bouton	CC	6	5	562	50	19809	1.34E-02
GO:0043122	regulation of I-kappaB kinase/	BP	9	3	562	18	19809	1.35E-02
GO:0003203	endocardial cushion morphoge	BP	7	3	562	18	19809	1.35E-02
GO:0014911	positive regulation of smooth r	BP	9	3	562	18	19809	1.35E-02
GO:0099092	postsynaptic density, intracellu	CC	5	3	562	18	19809	1.35E-02
GO:0043204	perikaryon	CC	5	9	562	133	19809	1.36E-02
GO:0048247	lymphocyte chemotaxis	BP	6	4	562	33	19809	1.37E-02
GO:0060828	regulation of canonical Wnt si	BP	9	4	562	33	19809	1.37E-02
GO:0045893	positive regulation of transcrip	BP	9	28	562	632	19809	1.38E-02
GO:0060326	cell chemotaxis	BP	6	7	562	90	19809	1.41E-02
GO:0045652	regulation of megakaryocyte d	BP	8	6	562	70	19809	1.44E-02
GO:0005782	peroxisomal matrix	CC	8	5	562	51	19809	1.45E-02
GO:0010043	response to zinc ion	BP	7	4	562	34	19809	1.52E-02
GO:0098978	glutamatergic synapse	CC	4	18	562	361	19809	1.53E-02
GO:0051005	negative regulation of lipoprot	BP	9	2	562	7	19809	1.53E-02
GO:0098821	BMP receptor activity	MF	10	2	562	7	19809	1.53E-02
GO:0009414	response to water deprivation	BP	5	2	562	7	19809	1.53E-02
GO:0051798	positive regulation of hair folli	BP	8	2	562	7	19809	1.53E-02
GO:0035425	autocrine signaling	BP	6	2	562	7	19809	1.53E-02
GO:0005021	vascular endothelial growth fa	MF	10	2	562	7	19809	1.53E-02
GO:0036016	cellular response to interleukin	BP	8	2	562	7	19809	1.53E-02
GO:0070852	cell body fiber	CC	6	2	562	7	19809	1.53E-02
GO:0045650	negative regulation of macroph	BP	10	2	562	7	19809	1.53E-02
GO:0005025	transforming growth factor bet	MF	11	2	562	7	19809	1.53E-02
GO:0008499	UDP-galactose:beta-N-acetylg	MF	8	2	562	7	19809	1.53E-02
GO:0003223	ventricular compact myocardiu	BP	9	2	562	7	19809	1.53E-02
GO:0006935	chemotaxis	BP	5	10	562	159	19809	1.55E-02
GO:0045598	regulation of fat cell differenti	BP	7	3	562	19	19809	1.57E-02
GO:1901741	positive regulation of myoblas	BP	11	3	562	19	19809	1.57E-02
GO:0097718	disordered domain specific bin	MF	6	4	562	35	19809	1.67E-02
GO:0055072	iron ion homeostasis	BP	11	4	562	35	19809	1.67E-02
GO:0051496	positive regulation of stress fib	BP	8	5	562	53	19809	1.69E-02
GO:0007411	axon guidance	BP	6	12	562	210	19809	1.71E-02
GO:0030335	positive regulation of cell migr	BP	8	13	562	235	19809	1.71E-02
GO:0030097	hemopoiesis	BP	7	6	562	73	19809	1.74E-02
GO:0043065	positive regulation of apoptoti	BP	9	18	562	367	19809	1.78E-02
GO:0006807	nitrogen compound metabolic	BP	4	3	562	20	19809	1.81E-02
GO:0001916	positive regulation of T cell m	BP	8	3	562	20	19809	1.81E-02
GO:0002063	chondrocyte development	BP	6	3	562	20	19809	1.81E-02
GO:0004745	retinol dehydrogenase activity	MF	7	3	562	20	19809	1.81E-02
GO:0032753	positive regulation of interleuk	BP	8	3	562	20	19809	1.81E-02
GO:0071526	semaphorin-plexin signaling pa	BP	6	4	562	36	19809	1.84E-02
GO:2001240	negative regulation of extrinsi	BP	10	4	562	36	19809	1.84E-02
GO:0042572	retinol metabolic process	BP	11	4	562	36	19809	1.84E-02
GO:0005581	collagen trimer	CC	4	7	562	95	19809	1.84E-02
GO:0060070	canonical Wnt signaling pathw	BP	8	6	562	74	19809	1.85E-02
GO:0038023	signaling receptor activity	MF	4	13	562	239	19809	1.93E-02
GO:0035331	negative regulation of hippo si	BP	10	2	562	8	19809	2.01E-02
GO:0003183	mitral valve morphogenesis	BP	7	2	562	8	19809	2.01E-02

GO:0002544	chronic inflammatory response	BP	7	2	562	8	19809	2.01E-02
GO:0032754	positive regulation of interleuk	BP	8	2	562	8	19809	2.01E-02
GO:0051045	negative regulation of membra	BP	9	2	562	8	19809	2.01E-02
GO:0032926	negative regulation of activin r	BP	10	2	562	8	19809	2.01E-02
GO:0001758	retinal dehydrogenase activity	MF	7	2	562	8	19809	2.01E-02
GO:0032675	regulation of interleukin-6 pro	BP	7	2	562	8	19809	2.01E-02
GO:0071316	cellular response to nicotine	BP	7	2	562	8	19809	2.01E-02
GO:0072205	metanephric collecting duct de	BP	7	2	562	8	19809	2.01E-02
GO:0030219	megakaryocyte differentiation	BP	7	2	562	8	19809	2.01E-02
GO:0048846	axon extension involved in axo	BP	8	2	562	8	19809	2.01E-02
GO:0002438	acute inflammatory response to	BP	8	2	562	8	19809	2.01E-02
GO:0045930	negative regulation of mitotic c	BP	8	4	562	37	19809	2.02E-02
GO:0001837	epithelial to mesenchymal tran	BP	7	4	562	37	19809	2.02E-02
GO:0033613	activating transcription factor b	MF	7	4	562	37	19809	2.02E-02
GO:0007160	cell-matrix adhesion	BP	6	7	562	97	19809	2.04E-02
GO:0030054	cell junction	CC	3	34	562	829	19809	2.06E-02
GO:0031012	extracellular matrix	CC	4	14	562	267	19809	2.07E-02
GO:0005089	Rho guanyl-nucleotide exchan	MF	9	6	562	77	19809	2.21E-02
GO:0060412	ventricular septum morphogen	BP	6	4	562	38	19809	2.21E-02
GO:2001237	negative regulation of extrinsic	BP	10	4	562	38	19809	2.21E-02
GO:0090050	positive regulation of cell migr	BP	12	4	562	38	19809	2.21E-02
GO:0042177	negative regulation of protein c	BP	8	4	562	38	19809	2.21E-02
GO:0071222	cellular response to lipopolysa	BP	8	10	562	169	19809	2.26E-02
GO:0032496	response to lipopolysaccharide	BP	7	10	562	169	19809	2.26E-02
GO:0005125	cytokine activity	MF	7	12	562	219	19809	2.28E-02
GO:0035556	intracellular signal transductio	BP	5	21	562	461	19809	2.32E-02
GO:0050431	transforming growth factor bet	MF	6	3	562	22	19809	2.34E-02
GO:0060384	innervation	BP	4	3	562	22	19809	2.34E-02
GO:0005667	transcription factor complex	CC	4	12	562	220	19809	2.36E-02
GO:0043507	positive regulation of JUN kin	BP	13	4	562	39	19809	2.41E-02
GO:0045429	positive regulation of nitric ox	BP	9	4	562	39	19809	2.41E-02
GO:0045746	negative regulation of Notch si	BP	9	4	562	39	19809	2.41E-02
GO:0042475	odontogenesis of dentin-contai	BP	7	5	562	58	19809	2.41E-02
GO:0035091	phosphatidylinositol binding	MF	6	7	562	101	19809	2.49E-02
GO:0071243	cellular response to arsenic-co	BP	6	2	562	9	19809	2.53E-02
GO:0032836	glomerular basement membran	BP	7	2	562	9	19809	2.53E-02
GO:0048339	paraxial mesoderm developme	BP	7	2	562	9	19809	2.53E-02
GO:0071394	cellular response to testosteron	BP	7	2	562	9	19809	2.53E-02
GO:0044309	neuron spine	CC	6	2	562	9	19809	2.53E-02
GO:0044598	doxorubicin metabolic process	BP	6	2	562	9	19809	2.53E-02
GO:0044597	daunorubicin metabolic proces	BP	6	2	562	9	19809	2.53E-02
GO:0031232	extrinsic component of externa	CC	6	2	562	9	19809	2.53E-02
GO:0060982	coronary artery morphogenesis	BP	8	2	562	9	19809	2.53E-02
GO:0048617	embryonic foregut morphogen	BP	6	2	562	9	19809	2.53E-02
GO:0070233	negative regulation of T cell ap	BP	12	2	562	9	19809	2.53E-02
GO:0014808	release of sequestered calcium	BP	12	2	562	9	19809	2.53E-02
GO:0061549	sympathetic ganglion develop	BP	7	2	562	9	19809	2.53E-02
GO:0003143	embryonic heart tube morphog	BP	6	2	562	9	19809	2.53E-02
GO:0002020	protease binding	MF	6	7	562	102	19809	2.61E-02
GO:0048471	perinuclear region of cytoplasm	CC	6	29	562	697	19809	2.62E-02

GO:0010628	positive regulation of gene exp	BP	8	20	562	439	19809	2.63E-02
GO:1903589	positive regulation of blood ve	BP	10	3	562	23	19809	2.64E-02
GO:0005520	insulin-like growth factor bind	MF	6	3	562	23	19809	2.64E-02
GO:0014032	neural crest cell development	BP	7	3	562	23	19809	2.64E-02
GO:0006952	defense response	BP	5	9	562	149	19809	2.64E-02
GO:0001666	response to hypoxia	BP	5	10	562	174	19809	2.70E-02
GO:0048661	positive regulation of smooth r	BP	8	5	562	60	19809	2.75E-02
GO:0071347	cellular response to interleukin	BP	8	6	562	81	19809	2.75E-02
GO:0098869	cellular oxidant detoxification	BP	5	6	562	81	19809	2.75E-02
GO:0009953	dorsal/ventral pattern formatio	BP	6	4	562	41	19809	2.84E-02
GO:1902038	positive regulation of hematop	BP	9	1	562	1	19809	2.84E-02
GO:0090193	positive regulation of glomeru	BP	8	1	562	1	19809	2.84E-02
GO:0150018	basal dendrite development	BP	9	1	562	1	19809	2.84E-02
GO:0051709	regulation of killing of cells of	BP	6	1	562	1	19809	2.84E-02
GO:1904591	positive regulation of protein i	BP	8	1	562	1	19809	2.84E-02
GO:0043170	macromolecule metabolic proc	BP	5	1	562	1	19809	2.84E-02
GO:0070230	positive regulation of lymphoc	BP	11	1	562	1	19809	2.84E-02
GO:0047020	15-hydroxyprostaglandin-D de	MF	7	1	562	1	19809	2.84E-02
GO:0090361	regulation of platelet-derived g	BP	7	1	562	1	19809	2.84E-02
GO:0042628	mating plug formation	BP	6	1	562	1	19809	2.84E-02
GO:0008470	isovaleryl-CoA dehydrogenase	MF	7	1	562	1	19809	2.84E-02
GO:0005166	neurotrophin p75 receptor binc	MF	8	1	562	1	19809	2.84E-02
GO:0052894	norspermine:oxygen oxidoredu	MF	7	1	562	1	19809	2.84E-02
GO:0035304	regulation of protein dephosph	BP	9	1	562	1	19809	2.84E-02
GO:0019544	arginine catabolic process to g	BP	13	1	562	1	19809	2.84E-02
GO:0034516	response to vitamin B6	BP	7	1	562	1	19809	2.84E-02
GO:0021626	central nervous system matura	BP	6	1	562	1	19809	2.84E-02
GO:0060301	positive regulation of cytokine	BP	9	1	562	1	19809	2.84E-02
GO:1900053	negative regulation of retinoic	BP	9	1	562	1	19809	2.84E-02
GO:0045703	ketoreductase activity	MF	6	1	562	1	19809	2.84E-02
GO:0061445	endocardial cushion cell fate c	BP	9	1	562	1	19809	2.84E-02
GO:0038161	prolactin signaling pathway	BP	6	1	562	1	19809	2.84E-02
GO:0099400	caveola neck	CC	5	1	562	1	19809	2.84E-02
GO:0050647	5-hydroxy-6E,8Z,11Z,14Z-ico:	MF	7	1	562	1	19809	2.84E-02
GO:2000764	positive regulation of semapho	BP	8	1	562	1	19809	2.84E-02
GO:0002818	intracellular defense response	BP	7	1	562	1	19809	2.84E-02
GO:0021683	cerebellar granular layer morp	BP	5	1	562	1	19809	2.84E-02
GO:0003837	beta-ureidopropionase activity	MF	7	1	562	1	19809	2.84E-02
GO:0044195	nucleoplasmic reticulum	CC	6	1	562	1	19809	2.84E-02
GO:0051867	general adaptation syndrome, l	BP	4	1	562	1	19809	2.84E-02
GO:0032567	dGTP binding	MF	9	1	562	1	19809	2.84E-02
GO:1902891	negative regulation of root hai	BP	8	1	562	1	19809	2.84E-02
GO:0045751	negative regulation of Toll sig	BP	9	1	562	1	19809	2.84E-02
GO:0002536	respiratory burst involved in ir	BP	5	1	562	1	19809	2.84E-02
GO:0019546	arginine deiminase pathway	BP	13	1	562	1	19809	2.84E-02
GO:0061196	fungiform papilla developmen	BP	5	1	562	1	19809	2.84E-02
GO:0010900	negative regulation of phospha	BP	8	1	562	1	19809	2.84E-02
GO:0060658	nipple morphogenesis	BP	5	1	562	1	19809	2.84E-02
GO:0045977	positive regulation of mitotic c	BP	9	1	562	1	19809	2.84E-02
GO:0061756	leukocyte adhesion to vascular	BP	7	1	562	1	19809	2.84E-02

GO:0010477	response to sulfur dioxide	BP	6	1	562	1	19809	2.84E-02
GO:0097185	cellular response to azide	BP	7	1	562	1	19809	2.84E-02
GO:0090594	inflammatory response to wou	BP	7	1	562	1	19809	2.84E-02
GO:0072141	renal interstitial fibroblast dev	BP	6	1	562	1	19809	2.84E-02
GO:0002067	glandular epithelial cell differe	BP	8	1	562	1	19809	2.84E-02
GO:0001910	regulation of leukocyte mediat	BP	6	1	562	1	19809	2.84E-02
GO:0032621	interleukin-18 production	BP	5	1	562	1	19809	2.84E-02
GO:1903841	cellular response to arsenite(3-	BP	7	1	562	1	19809	2.84E-02
GO:0046722	lactic acid secretion	BP	14	1	562	1	19809	2.84E-02
GO:0090265	positive regulation of immune	BP	8	1	562	1	19809	2.84E-02
GO:0045032	ADP-activated adenosine rece	MF	8	1	562	1	19809	2.84E-02
GO:0006061	sorbitol biosynthetic process	BP	10	1	562	1	19809	2.84E-02
GO:0016863	intramolecular oxidoreductase	MF	6	1	562	1	19809	2.84E-02
GO:0060872	semicircular canal developmen	BP	6	1	562	1	19809	2.84E-02
GO:2000691	negative regulation of cardiac	BP	9	1	562	1	19809	2.84E-02
GO:0033396	beta-alanine biosynthetic proc	BP	11	1	562	1	19809	2.84E-02
GO:0031764	type 1 galanin receptor binding	MF	9	1	562	1	19809	2.84E-02
GO:1904414	positive regulation of cardiac	BP	7	1	562	1	19809	2.84E-02
GO:0061767	negative regulation of lung blo	BP	7	1	562	1	19809	2.84E-02
GO:1902378	VEGF-activated neuropilin sig	BP	10	1	562	1	19809	2.84E-02
GO:0071820	N-box binding	MF	11	1	562	1	19809	2.84E-02
GO:1905430	cellular response to glycine	BP	9	1	562	1	19809	2.84E-02
GO:2001202	negative regulation of transfor	BP	9	1	562	1	19809	2.84E-02
GO:0072683	T cell extravasation	BP	6	1	562	1	19809	2.84E-02
GO:0052047	interaction with other organism	BP	5	1	562	1	19809	2.84E-02
GO:2000227	negative regulation of pancrea	BP	9	1	562	1	19809	2.84E-02
GO:0072677	eosinophil migration	BP	7	1	562	1	19809	2.84E-02
GO:0004919	interleukin-9 receptor activity	MF	7	1	562	1	19809	2.84E-02
GO:1900044	regulation of protein K63-link	BP	12	1	562	1	19809	2.84E-02
GO:0052600	propane-1,3-diamine oxidase a	MF	7	1	562	1	19809	2.84E-02
GO:1905929	positive regulation of invadop	BP	9	1	562	1	19809	2.84E-02
GO:0097238	cellular response to methylgly	BP	8	1	562	1	19809	2.84E-02
GO:1903125	negative regulation of thioredo	BP	11	1	562	1	19809	2.84E-02
GO:0070572	positive regulation of neuron p	BP	12	1	562	1	19809	2.84E-02
GO:0035716	chemokine (C-C motif) ligand	MF	8	1	562	1	19809	2.84E-02
GO:0016990	arginine deiminase activity	MF	7	1	562	1	19809	2.84E-02
GO:0046880	regulation of follicle-stimulati	BP	9	1	562	1	19809	2.84E-02
GO:0002002	regulation of angiotensin level	BP	6	1	562	1	19809	2.84E-02
GO:0098902	regulation of membrane depol	BP	7	1	562	1	19809	2.84E-02
GO:0070678	preprotein binding	MF	5	1	562	1	19809	2.84E-02
GO:1904620	cellular response to dimethyl s	BP	7	1	562	1	19809	2.84E-02
GO:1990407	calcitonin gene-related peptide	MF	8	1	562	1	19809	2.84E-02
GO:0099569	presynaptic cytoskeleton	CC	7	1	562	1	19809	2.84E-02
GO:0061670	evoked neurotransmitter secret	BP	7	1	562	1	19809	2.84E-02
GO:0035874	cellular response to copper ion	BP	9	1	562	1	19809	2.84E-02
GO:0071966	fungal-type cell wall polysacc	BP	9	1	562	1	19809	2.84E-02
GO:1905872	negative regulation of protein	BP	7	1	562	1	19809	2.84E-02
GO:0043509	activin A complex	CC	5	1	562	1	19809	2.84E-02
GO:0016160	amylase activity	MF	7	1	562	1	19809	2.84E-02
GO:1903904	negative regulation of establis	BP	10	1	562	1	19809	2.84E-02

GO:0008458	carnitine O-octanoyltransferas	MF	9	1	562	1	19809	2.84E-02
GO:0009013	succinate-semialdehyde dehyd	MF	7	1	562	1	19809	2.84E-02
GO:0004398	histidine decarboxylase activit	MF	7	1	562	1	19809	2.84E-02
GO:0004034	aldose 1-epimerase activity	MF	7	1	562	1	19809	2.84E-02
GO:0048264	determination of ventral identi	BP	7	1	562	1	19809	2.84E-02
GO:1905875	negative regulation of postsyna	BP	9	1	562	1	19809	2.84E-02
GO:0002578	negative regulation of antigen	BP	7	1	562	1	19809	2.84E-02
GO:0034592	synaptic vesicle lumen	CC	4	1	562	1	19809	2.84E-02
GO:2000570	positive regulation of T-helper	BP	12	1	562	1	19809	2.84E-02
GO:0002246	wound healing involved in infl	BP	7	1	562	1	19809	2.84E-02
GO:0072705	cellular response to mercaptoe	BP	8	1	562	1	19809	2.84E-02
GO:1904887	Wnt signalosome assembly	BP	7	1	562	1	19809	2.84E-02
GO:0050701	interleukin-1 secretion	BP	12	1	562	1	19809	2.84E-02
GO:0002828	regulation of type 2 immune re	BP	7	1	562	1	19809	2.84E-02
GO:0002581	negative regulation of antigen	BP	8	1	562	1	19809	2.84E-02
GO:2000464	positive regulation of astrocyte	BP	9	1	562	1	19809	2.84E-02
GO:0090075	relaxation of muscle	BP	6	1	562	1	19809	2.84E-02
GO:0052599	methylputrescine oxidase activ	MF	7	1	562	1	19809	2.84E-02
GO:0045186	zonula adherens assembly	BP	8	1	562	1	19809	2.84E-02
GO:0048685	negative regulation of collatera	BP	9	1	562	1	19809	2.84E-02
GO:2000415	positive regulation of fibronec	BP	11	1	562	1	19809	2.84E-02
GO:1990858	cellular response to lectin	BP	6	1	562	1	19809	2.84E-02
GO:0003830	beta-1,4-mannosylglycoprotein	MF	8	1	562	1	19809	2.84E-02
GO:0006848	pyruvate transport	BP	11	1	562	1	19809	2.84E-02
GO:0036131	prostaglandin D2 11-ketoreduc	MF	7	1	562	1	19809	2.84E-02
GO:0061768	magnesium:sodium antiporter	MF	10	1	562	1	19809	2.84E-02
GO:0061981	3-hydroxykynureninase activit	MF	7	1	562	1	19809	2.84E-02
GO:0150090	multiple spine synapse organiz	BP	7	1	562	1	19809	2.84E-02
GO:2000763	positive regulation of transcrip	BP	11	1	562	1	19809	2.84E-02
GO:0052598	histamine oxidase activity	MF	7	1	562	1	19809	2.84E-02
GO:0043795	glyceraldehyde oxidoreductase	MF	6	1	562	1	19809	2.84E-02
GO:0043132	NAD transport	BP	9	1	562	1	19809	2.84E-02
GO:0072010	glomerular epithelium develop	BP	9	1	562	1	19809	2.84E-02
GO:2001264	negative regulation of C-C che	BP	8	1	562	1	19809	2.84E-02
GO:0034369	plasma lipoprotein particle ren	BP	8	1	562	1	19809	2.84E-02
GO:2000661	positive regulation of interleuk	BP	10	1	562	1	19809	2.84E-02
GO:0060825	fibroblast growth factor recept	BP	9	1	562	1	19809	2.84E-02
GO:0098542	defense response to other orga	BP	6	1	562	1	19809	2.84E-02
GO:2000451	positive regulation of CD8-pos	BP	10	1	562	1	19809	2.84E-02
GO:0050646	5-oxo-6E,8Z,11Z,14Z-icosatet	MF	5	1	562	1	19809	2.84E-02
GO:0004925	prolactin receptor activity	MF	7	1	562	1	19809	2.84E-02
GO:0000304	response to singlet oxygen	BP	7	1	562	1	19809	2.84E-02
GO:0014835	myoblast differentiation involv	BP	7	1	562	1	19809	2.84E-02
GO:0005147	oncostatin-M receptor binding	MF	7	1	562	1	19809	2.84E-02
GO:0061108	seminal vesicle epithelium dev	BP	6	1	562	1	19809	2.84E-02
GO:0071795	K11-linked polyubiquitin mod	MF	7	1	562	1	19809	2.84E-02
GO:0061048	negative regulation of branchin	BP	9	1	562	1	19809	2.84E-02
GO:0061881	positive regulation of anterogra	BP	9	1	562	1	19809	2.84E-02
GO:0031765	type 2 galanin receptor binding	MF	9	1	562	1	19809	2.84E-02
GO:0043310	negative regulation of eosinopl	BP	10	1	562	1	19809	2.84E-02

GO:0036479	peroxidase inhibitor activity	MF	6	1	562	1	19809	2.84E-02
GO:0016854	racemase and epimerase activi	MF	5	1	562	1	19809	2.84E-02
GO:0004676	3-phosphoinositide-dependent	MF	9	1	562	1	19809	2.84E-02
GO:0071133	alpha9-beta1 integrin-ADAM8	CC	6	1	562	1	19809	2.84E-02
GO:0005983	starch catabolic process	BP	10	1	562	1	19809	2.84E-02
GO:0004055	argininosuccinate synthase act	MF	6	1	562	1	19809	2.84E-02
GO:0030429	kynureninase activity	MF	7	1	562	1	19809	2.84E-02
GO:0090096	positive regulation of metanep	BP	9	1	562	1	19809	2.84E-02
GO:0031686	A1 adenosine receptor binding	MF	8	1	562	1	19809	2.84E-02
GO:0051886	negative regulation of timing c	BP	10	1	562	1	19809	2.84E-02
GO:0004877	complement component C3b r	MF	7	1	562	1	19809	2.84E-02
GO:0061837	neuropeptide processing	BP	9	1	562	1	19809	2.84E-02
GO:2000697	negative regulation of epitheli	BP	9	1	562	1	19809	2.84E-02
GO:0050712	negative regulation of interleu	BP	10	1	562	1	19809	2.84E-02
GO:2000313	regulation of fibroblast growth	BP	9	1	562	1	19809	2.84E-02
GO:0052895	N1-acetylspermine:oxygen oxi	MF	7	1	562	1	19809	2.84E-02
GO:1904237	positive regulation of substrate	BP	8	1	562	1	19809	2.84E-02
GO:0002300	CD8-positive, alpha-beta intra	BP	11	1	562	1	19809	2.84E-02
GO:0070553	nicotinic acid receptor activity	MF	7	1	562	1	19809	2.84E-02
GO:1990678	histone H4-K16 deacetylation	BP	13	1	562	1	19809	2.84E-02
GO:0048319	axial mesoderm morphogenesi	BP	7	1	562	1	19809	2.84E-02
GO:0004587	ornithine-oxo-acid transamina	MF	7	1	562	1	19809	2.84E-02
GO:2000977	regulation of forebrain neuron	BP	10	1	562	1	19809	2.84E-02
GO:0052597	diamine oxidase activity	MF	7	1	562	1	19809	2.84E-02
GO:2000224	regulation of testosterone bios	BP	9	1	562	1	19809	2.84E-02
GO:0036130	prostaglandin H2 endoperoxid	MF	7	1	562	1	19809	2.84E-02
GO:1901557	response to fenofibrate	BP	6	1	562	1	19809	2.84E-02
GO:0060427	lung connective tissue develop	BP	7	1	562	1	19809	2.84E-02
GO:0000435	positive regulation of transcrip	BP	11	1	562	1	19809	2.84E-02
GO:0016495	C-X3-C chemokine receptor a	MF	9	1	562	1	19809	2.84E-02
GO:2001107	negative regulation of Rho gua	BP	9	1	562	1	19809	2.84E-02
GO:2001031	positive regulation of cellular	BP	10	1	562	1	19809	2.84E-02
GO:0044546	NLRP3 inflammasome comple	BP	8	1	562	1	19809	2.84E-02
GO:0071545	inositol phosphate catabolic pr	BP	8	1	562	1	19809	2.84E-02
GO:0016793	triphosphoric monoester hydro	MF	7	1	562	1	19809	2.84E-02
GO:0042107	cytokine metabolic process	BP	7	1	562	1	19809	2.84E-02
GO:0033682	ATP-dependent 5'-3' DNA/RN	MF	12	1	562	1	19809	2.84E-02
GO:0001770	establishment of natural killer	BP	7	1	562	1	19809	2.84E-02
GO:0140194	negative regulation of adenyla	BP	10	1	562	1	19809	2.84E-02
GO:1905040	otic placode development	BP	6	1	562	1	19809	2.84E-02
GO:0061573	actin filament bundle retrograd	BP	9	1	562	1	19809	2.84E-02
GO:0047273	galactosylgalactosylglucosylce	MF	8	1	562	1	19809	2.84E-02
GO:0034739	histone deacetylase activity (H	MF	8	1	562	1	19809	2.84E-02
GO:0099013	neuronal dense core vesicle lu	CC	5	1	562	1	19809	2.84E-02
GO:0004092	carnitine O-acetyltransferase a	MF	9	1	562	1	19809	2.84E-02
GO:0034211	GTP-dependent protein kinase	MF	9	1	562	1	19809	2.84E-02
GO:0071384	cellular response to corticoste	BP	8	1	562	1	19809	2.84E-02
GO:0031766	type 3 galanin receptor binding	MF	9	1	562	1	19809	2.84E-02
GO:0004777	succinate-semialdehyde dehyd	MF	7	1	562	1	19809	2.84E-02
GO:0050648	5(S)-hydroxyperoxy-6E,8Z,11	MF	7	1	562	1	19809	2.84E-02

GO:0023025	MHC class Ib protein complex	MF	6	1	562	1	19809	2.84E-02
GO:0061163	endoplasmic reticulum polariz	BP	7	1	562	1	19809	2.84E-02
GO:0010121	arginine catabolic process to p	BP	14	1	562	1	19809	2.84E-02
GO:0005582	collagen type XV trimer	CC	6	1	562	1	19809	2.84E-02
GO:0035715	chemokine (C-C motif) ligand	MF	8	1	562	1	19809	2.84E-02
GO:0030882	lipid antigen binding	MF	5	1	562	1	19809	2.84E-02
GO:0021998	neural plate mediolateral regio	BP	7	1	562	1	19809	2.84E-02
GO:0035564	regulation of kidney size	BP	6	1	562	1	19809	2.84E-02
GO:0035705	T-helper 17 cell chemotaxis	BP	9	1	562	1	19809	2.84E-02
GO:0031962	mineralocorticoid receptor bin	MF	9	1	562	1	19809	2.84E-02
GO:1905835	cellular response to pyrimidine	BP	8	1	562	1	19809	2.84E-02
GO:0072703	cellular response to methyl me	BP	7	1	562	1	19809	2.84E-02
GO:0032396	inhibitory MHC class I recepto	MF	7	1	562	1	19809	2.84E-02
GO:0006083	acetate metabolic process	BP	7	1	562	1	19809	2.84E-02
GO:2001203	positive regulation of transforr	BP	9	1	562	1	19809	2.84E-02
GO:1905285	fibrous ring of heart morphoge	BP	6	1	562	1	19809	2.84E-02
GO:0008832	dGTPase activity	MF	8	1	562	1	19809	2.84E-02
GO:0000053	argininosuccinate metabolic pr	BP	6	1	562	1	19809	2.84E-02
GO:0060896	neural plate pattern specificati	BP	5	1	562	1	19809	2.84E-02
GO:0046449	creatinine metabolic process	BP	8	1	562	1	19809	2.84E-02
GO:0061106	negative regulation of stomach	BP	8	1	562	1	19809	2.84E-02
GO:0001861	complement component C4b re	MF	7	1	562	1	19809	2.84E-02
GO:0048352	paraxial mesoderm structural c	BP	6	1	562	1	19809	2.84E-02
GO:0045381	regulation of interleukin-18 bi	BP	8	1	562	1	19809	2.84E-02
GO:1902946	protein localization to early en	BP	9	1	562	1	19809	2.84E-02
GO:0051021	GDP-dissociation inhibitor bin	MF	5	1	562	1	19809	2.84E-02
GO:0047915	ganglioside galactosyltransfera	MF	8	1	562	1	19809	2.84E-02
GO:1902498	regulation of protein autoubiqu	BP	11	1	562	1	19809	2.84E-02
GO:0007193	adenylate cyclase-inhibiting G	BP	8	5	562	61	19809	2.92E-02
GO:0042157	lipoprotein metabolic process	BP	7	3	562	24	19809	2.95E-02
GO:0045671	negative regulation of osteocla	BP	10	3	562	24	19809	2.95E-02
GO:0010608	posttranscriptional regulation c	BP	8	3	562	24	19809	2.95E-02
GO:0043236	laminin binding	MF	5	3	562	24	19809	2.95E-02
GO:0006693	prostaglandin metabolic proces	BP	10	3	562	24	19809	2.95E-02
GO:0045089	positive regulation of innate in	BP	8	3	562	24	19809	2.95E-02
GO:0045879	negative regulation of smoothe	BP	9	3	562	24	19809	2.95E-02
GO:0051092	positive regulation of NF-kapp	BP	7	9	562	152	19809	2.96E-02
GO:0050776	regulation of immune response	BP	6	11	562	202	19809	2.97E-02
GO:0001657	ureteric bud development	BP	7	4	562	42	19809	3.07E-02
GO:0002062	chondrocyte differentiation	BP	6	4	562	42	19809	3.07E-02
GO:0003690	double-stranded DNA binding	MF	7	8	562	129	19809	3.08E-02
GO:0045669	positive regulation of osteobla	BP	8	5	562	62	19809	3.11E-02
GO:0042102	positive regulation of T cell pr	BP	11	5	562	62	19809	3.11E-02
GO:0048762	mesenchymal cell differentiati	BP	6	2	562	10	19809	3.11E-02
GO:0007567	parturition	BP	5	2	562	10	19809	3.11E-02
GO:0006900	vesicle budding from membrar	BP	7	2	562	10	19809	3.11E-02
GO:0061037	negative regulation of cartilage	BP	7	2	562	10	19809	3.11E-02
GO:0006930	substrate-dependent cell migra	BP	8	2	562	10	19809	3.11E-02
GO:0042135	neurotransmitter catabolic proc	BP	7	2	562	10	19809	3.11E-02
GO:0032060	bleb assembly	BP	8	2	562	10	19809	3.11E-02

GO:0003680	AT DNA binding	MF	9	2	562	10	19809	3.11E-02
GO:0010467	gene expression	BP	6	2	562	10	19809	3.11E-02
GO:0086013	membrane repolarization durir	BP	10	2	562	10	19809	3.11E-02
GO:0042448	progesterone metabolic proces	BP	8	2	562	10	19809	3.11E-02
GO:0071287	cellular response to manganese	BP	8	2	562	10	19809	3.11E-02
GO:0045080	positive regulation of chemoki	BP	9	2	562	10	19809	3.11E-02
GO:0033089	positive regulation of T cell di	BP	11	2	562	10	19809	3.11E-02
GO:0001843	neural tube closure	BP	7	6	562	84	19809	3.21E-02
GO:0033993	response to lipid	BP	6	3	562	25	19809	3.29E-02
GO:0048843	negative regulation of axon ex	BP	9	3	562	25	19809	3.29E-02
GO:0048844	artery morphogenesis	BP	7	3	562	25	19809	3.29E-02
GO:0022617	extracellular matrix disassemb	BP	6	5	562	63	19809	3.30E-02
GO:0002548	monocyte chemotaxis	BP	6	4	562	43	19809	3.31E-02
GO:0001541	ovarian follicle development	BP	6	4	562	43	19809	3.31E-02
GO:0008277	regulation of G protein-couple	BP	8	4	562	43	19809	3.31E-02
GO:0019722	calcium-mediated signaling	BP	7	6	562	85	19809	3.38E-02
GO:0048010	vascular endothelial growth fa	BP	8	5	562	64	19809	3.50E-02
GO:0032729	positive regulation of interfero	BP	8	4	562	44	19809	3.56E-02
GO:0042104	positive regulation of activatec	BP	12	3	562	26	19809	3.64E-02
GO:0046697	decidualization	BP	5	3	562	26	19809	3.64E-02
GO:0042493	response to drug	BP	5	15	562	316	19809	3.68E-02
GO:0006625	protein targeting to peroxisom	BP	12	5	562	65	19809	3.71E-02
GO:0001664	G protein-coupled receptor bin	MF	6	5	562	65	19809	3.71E-02
GO:0007169	transmembrane receptor protei	BP	7	7	562	110	19809	3.72E-02
GO:0034101	erythrocyte homeostasis	BP	5	2	562	11	19809	3.73E-02
GO:0051246	regulation of protein metabolic	BP	7	2	562	11	19809	3.73E-02
GO:0001778	plasma membrane repair	BP	7	2	562	11	19809	3.73E-02
GO:0010918	positive regulation of mitochor	BP	7	2	562	11	19809	3.73E-02
GO:0015386	potassium:proton antiporter ac	MF	12	2	562	11	19809	3.73E-02
GO:0048711	positive regulation of astrocyte	BP	12	2	562	11	19809	3.73E-02
GO:0097242	amyloid-beta clearance	BP	4	2	562	11	19809	3.73E-02
GO:0097284	hepatocyte apoptotic process	BP	8	2	562	11	19809	3.73E-02
GO:0000185	activation of MAPKKK activit	BP	13	2	562	11	19809	3.73E-02
GO:0032731	positive regulation of interleuk	BP	9	2	562	11	19809	3.73E-02
GO:0032204	regulation of telomere mainten	BP	9	2	562	11	19809	3.73E-02
GO:0048012	hepatocyte growth factor recep	BP	8	2	562	11	19809	3.73E-02
GO:0032691	negative regulation of interleul	BP	9	2	562	11	19809	3.73E-02
GO:0086009	membrane repolarization	BP	8	2	562	11	19809	3.73E-02
GO:0008037	cell recognition	BP	4	2	562	11	19809	3.73E-02
GO:0030134	COPII-coated ER to Golgi trar	CC	8	4	562	45	19809	3.82E-02
GO:0032870	cellular response to hormone s	BP	6	4	562	45	19809	3.82E-02
GO:0016020	membrane	CC	3	261	562	8459	19809	3.83E-02
GO:0014068	positive regulation of phospha	BP	10	6	562	88	19809	3.90E-02
GO:0035023	regulation of Rho protein signa	BP	11	6	562	88	19809	3.90E-02
GO:0001958	endochondral ossification	BP	6	3	562	27	19809	4.01E-02
GO:0071377	cellular response to glucagon s	BP	8	3	562	27	19809	4.01E-02
GO:0003148	outflow tract septum morphoge	BP	6	3	562	27	19809	4.01E-02
GO:0001755	neural crest cell migration	BP	8	4	562	46	19809	4.10E-02
GO:0060021	roof of mouth development	BP	5	5	562	67	19809	4.14E-02
GO:0007050	cell cycle arrest	BP	5	8	562	138	19809	4.32E-02

GO:0048812	neuron projection morphogene	BP	8	5	562	68	19809	4.37E-02
GO:0050918	positive chemotaxis	BP	6	4	562	47	19809	4.38E-02
GO:0071364	cellular response to epidermal	BP	6	4	562	47	19809	4.38E-02
GO:0030983	mismatched DNA binding	MF	8	2	562	12	19809	4.39E-02
GO:0032695	negative regulation of interleul	BP	8	2	562	12	19809	4.39E-02
GO:0030371	translation repressor activity	MF	4	2	562	12	19809	4.39E-02
GO:2000178	negative regulation of neural p	BP	8	2	562	12	19809	4.39E-02
GO:0042308	negative regulation of protein i	BP	10	2	562	12	19809	4.39E-02
GO:0055119	relaxation of cardiac muscle	BP	7	2	562	12	19809	4.39E-02
GO:0048715	negative regulation of oligode	BP	12	2	562	12	19809	4.39E-02
GO:0090331	negative regulation of platelet	BP	10	2	562	12	19809	4.39E-02
GO:0000002	mitochondrial genome mainter	BP	7	2	562	12	19809	4.39E-02
GO:0043652	engulfment of apoptotic cell	BP	9	2	562	12	19809	4.39E-02
GO:0006700	C21-steroid hormone biosynth	BP	8	2	562	12	19809	4.39E-02
GO:0015385	sodium:proton antiporter activ	MF	12	2	562	12	19809	4.39E-02
GO:1902287	semaphorin-plexin signaling p	BP	8	2	562	12	19809	4.39E-02
GO:0010763	positive regulation of fibrobla	BP	9	2	562	12	19809	4.39E-02
GO:0002003	angiotensin maturation	BP	9	2	562	12	19809	4.39E-02
GO:0045591	positive regulation of regulato	BP	11	2	562	12	19809	4.39E-02
GO:0045730	respiratory burst	BP	4	2	562	12	19809	4.39E-02
GO:0097028	dendritic cell differentiation	BP	7	2	562	12	19809	4.39E-02
GO:0051926	negative regulation of calcium	BP	9	2	562	12	19809	4.39E-02
GO:0090197	positive regulation of chemoki	BP	9	2	562	12	19809	4.39E-02
GO:0030426	growth cone	CC	5	8	562	139	19809	4.48E-02
GO:0044212	transcription regulatory region	MF	7	12	562	244	19809	4.63E-02
GO:0050890	cognition	BP	6	4	562	48	19809	4.68E-02
GO:0051056	regulation of small GTPase me	BP	9	8	562	141	19809	4.80E-02
GO:0030511	positive regulation of transfor	BP	10	3	562	29	19809	4.81E-02
GO:2000778	positive regulation of interleuk	BP	9	3	562	29	19809	4.81E-02
GO:0050715	positive regulation of cytokine	BP	8	3	562	29	19809	4.81E-02
GO:0002102	podosome	CC	4	3	562	29	19809	4.81E-02
GO:0046513	ceramide biosynthetic process	BP	10	3	562	29	19809	4.81E-02
GO:0048020	CCR chemokine receptor bind	MF	8	3	562	29	19809	4.81E-02
GO:0005604	basement membrane	CC	6	6	562	93	19809	4.89E-02
GO:0050900	leukocyte migration	BP	4	10	562	193	19809	4.91E-02
GO:0008009	chemokine activity	MF	8	4	562	49	19809	4.98E-02
GO:0045668	negative regulation of osteobla	BP	8	4	562	49	19809	4.98E-02
GO:0045599	negative regulation of fat cell (	BP	8	4	562	49	19809	4.98E-02
GO:0016446	somatic hypermutation of imm	BP	6	2	562	13	19809	5.10E-02
GO:0039706	co-receptor binding	MF	5	2	562	13	19809	5.10E-02
GO:0060088	auditory receptor cell stereocil	BP	9	2	562	13	19809	5.10E-02
GO:0001775	cell activation	BP	4	2	562	13	19809	5.10E-02
GO:0004032	alditol:NADP+ 1-oxidoreducta	MF	9	2	562	13	19809	5.10E-02
GO:0004675	transmembrane receptor protei	MF	9	2	562	13	19809	5.10E-02
GO:0048266	behavioral response to pain	BP	4	2	562	13	19809	5.10E-02
GO:0008106	alcohol dehydrogenase (NADI	MF	8	2	562	13	19809	5.10E-02
GO:0050966	detection of mechanical stimul	BP	7	2	562	13	19809	5.10E-02
GO:0051770	positive regulation of nitric-ox	BP	9	2	562	13	19809	5.10E-02
GO:0042531	positive regulation of tyrosine	BP	11	5	562	71	19809	5.10E-02
GO:0071300	cellular response to retinoic ac	BP	7	5	562	71	19809	5.10E-02

GO:0033209	tumor necrosis factor-mediatec	BP	7	7	562	118	19809	5.10E-02
GO:0045471	response to ethanol	BP	6	7	562	118	19809	5.10E-02
GO:0004867	serine-type endopeptidase inhi	MF	8	6	562	94	19809	5.11E-02
GO:0030334	regulation of cell migration	BP	7	6	562	94	19809	5.11E-02
GO:0016477	cell migration	BP	6	11	562	221	19809	5.12E-02
GO:0007596	blood coagulation	BP	7	10	562	195	19809	5.20E-02
GO:0046677	response to antibiotic	BP	5	3	562	30	19809	5.23E-02
GO:0030669	clathrin-coated endocytic vesic	CC	9	3	562	30	19809	5.23E-02
GO:0051966	regulation of synaptic transmis	BP	9	3	562	30	19809	5.23E-02
GO:0051491	positive regulation of filopodiu	BP	11	3	562	30	19809	5.23E-02
GO:0030833	regulation of actin filament po	BP	8	3	562	30	19809	5.23E-02
GO:0048008	platelet-derived growth factor	BP	8	3	562	30	19809	5.23E-02
GO:0046427	positive regulation of receptor	BP	10	3	562	30	19809	5.23E-02
GO:0051968	positive regulation of synaptic	BP	9	3	562	30	19809	5.23E-02
GO:0010976	positive regulation of neuron p	BP	11	7	562	119	19809	5.30E-02
GO:0008630	intrinsic apoptotic signaling pa	BP	7	4	562	50	19809	5.30E-02
GO:0042060	wound healing	BP	6	6	562	95	19809	5.33E-02
GO:0070821	tertiary granule membrane	CC	8	5	562	72	19809	5.36E-02
GO:0005515	protein binding	MF	4	326	562	10822	19809	5.59E-02
GO:0038102	activin receptor antagonist acti	MF	7	1	562	2	19809	5.59E-02
GO:0030546	receptor activator activity	MF	5	1	562	2	19809	5.59E-02
GO:0046370	fructose biosynthetic process	BP	9	1	562	2	19809	5.59E-02
GO:0034669	integrin alpha4-beta7 complex	CC	6	1	562	2	19809	5.59E-02
GO:0003366	cell-matrix adhesion involved	BP	7	1	562	2	19809	5.59E-02
GO:0004699	calcium-independent protein k	MF	10	1	562	2	19809	5.59E-02
GO:1902499	positive regulation of protein a	BP	12	1	562	2	19809	5.59E-02
GO:0035669	TRAM-dependent toll-like rec	BP	10	1	562	2	19809	5.59E-02
GO:0071796	K6-linked polyubiquitin modif	MF	7	1	562	2	19809	5.59E-02
GO:0045030	UTP-activated nucleotide rece	MF	10	1	562	2	19809	5.59E-02
GO:0097475	motor neuron migration	BP	8	1	562	2	19809	5.59E-02
GO:2001183	negative regulation of interleul	BP	9	1	562	2	19809	5.59E-02
GO:0051153	regulation of striated muscle c	BP	8	1	562	2	19809	5.59E-02
GO:0034395	regulation of transcription fror	BP	10	1	562	2	19809	5.59E-02
GO:0035821	modification of morphology oı	BP	5	1	562	2	19809	5.59E-02
GO:1905279	regulation of retrograde transp	BP	7	1	562	2	19809	5.59E-02
GO:0050975	sensory perception of touch	BP	8	1	562	2	19809	5.59E-02
GO:0002215	defense response to nematode	BP	7	1	562	2	19809	5.59E-02
GO:0030832	regulation of actin filament ler	BP	6	1	562	2	19809	5.59E-02
GO:0034665	integrin alpha1-beta1 complex	CC	6	1	562	2	19809	5.59E-02
GO:0071242	cellular response to ammoniun	BP	7	1	562	2	19809	5.59E-02
GO:0021893	cerebral cortex GABAergic int	BP	7	1	562	2	19809	5.59E-02
GO:0002305	CD8-positive, gamma-delta int	BP	11	1	562	2	19809	5.59E-02
GO:0070548	L-glutamine aminotransferase	MF	7	1	562	2	19809	5.59E-02
GO:0021636	trigeminal nerve morphogenes	BP	6	1	562	2	19809	5.59E-02
GO:1904586	cellular response to putrescine	BP	8	1	562	2	19809	5.59E-02
GO:0003064	regulation of heart rate by horı	BP	11	1	562	2	19809	5.59E-02
GO:0070165	positive regulation of adiponec	BP	9	1	562	2	19809	5.59E-02
GO:0004909	interleukin-1, type I, activating	MF	8	1	562	2	19809	5.59E-02
GO:0032655	regulation of interleukin-12 pr	BP	7	1	562	2	19809	5.59E-02
GO:0051100	negative regulation of binding	BP	6	1	562	2	19809	5.59E-02

GO:1900924	negative regulation of glycine	BP	8	1	562	2	19809	5.59E-02
GO:0036413	histone H3-R26 citrullination	BP	11	1	562	2	19809	5.59E-02
GO:0071418	cellular response to amine stir	BP	8	1	562	2	19809	5.59E-02
GO:0001621	ADP receptor activity	MF	9	1	562	2	19809	5.59E-02
GO:0072509	divalent inorganic cation trans	MF	8	1	562	2	19809	5.59E-02
GO:1900248	negative regulation of cytoplas	BP	12	1	562	2	19809	5.59E-02
GO:2000334	positive regulation of blood mi	BP	7	1	562	2	19809	5.59E-02
GO:0019442	tryptophan catabolic process tc	BP	9	1	562	2	19809	5.59E-02
GO:0030222	eosinophil differentiation	BP	9	1	562	2	19809	5.59E-02
GO:0016492	G protein-coupled neurotensin	MF	8	1	562	2	19809	5.59E-02
GO:1903217	negative regulation of protein j	BP	11	1	562	2	19809	5.59E-02
GO:0098508	endothelial to hematopoietic tr	BP	8	1	562	2	19809	5.59E-02
GO:0018931	naphthalene metabolic process	BP	6	1	562	2	19809	5.59E-02
GO:0051386	regulation of neurotrophin TRI	BP	8	1	562	2	19809	5.59E-02
GO:0032473	cytoplasmic side of mitochond	CC	5	1	562	2	19809	5.59E-02
GO:0048378	regulation of lateral mesoderm	BP	10	1	562	2	19809	5.59E-02
GO:0045844	positive regulation of striated r	BP	9	1	562	2	19809	5.59E-02
GO:0043512	inhibin A complex	CC	5	1	562	2	19809	5.59E-02
GO:0003977	UDP-N-acetylglucosamine dip	MF	8	1	562	2	19809	5.59E-02
GO:0045125	bioactive lipid receptor activity	MF	7	1	562	2	19809	5.59E-02
GO:0045601	regulation of endothelial cell d	BP	8	1	562	2	19809	5.59E-02
GO:0010623	programmed cell death involve	BP	6	1	562	2	19809	5.59E-02
GO:0033363	secretory granule organization	BP	7	1	562	2	19809	5.59E-02
GO:0009756	carbohydrate mediated signalii	BP	5	1	562	2	19809	5.59E-02
GO:0004392	heme oxygenase (decyclizing)	MF	6	1	562	2	19809	5.59E-02
GO:0003430	growth plate cartilage chondro	BP	7	1	562	2	19809	5.59E-02
GO:0004056	argininosuccinate lyase activit	MF	7	1	562	2	19809	5.59E-02
GO:0035410	dihydrotestosterone 17-beta-de	MF	7	1	562	2	19809	5.59E-02
GO:0045031	ATP-activated adenosine recep	MF	8	1	562	2	19809	5.59E-02
GO:0021557	oculomotor nerve developmen	BP	7	1	562	2	19809	5.59E-02
GO:0018008	N-terminal peptidyl-glycine N-	BP	12	1	562	2	19809	5.59E-02
GO:0060352	cell adhesion molecule produc	BP	4	1	562	2	19809	5.59E-02
GO:0072560	type B pancreatic cell maturati	BP	9	1	562	2	19809	5.59E-02
GO:0110025	DNA strand resection involvec	BP	8	1	562	2	19809	5.59E-02
GO:1904172	positive regulation of bleb asse	BP	10	1	562	2	19809	5.59E-02
GO:2000017	positive regulation of determin	BP	7	1	562	2	19809	5.59E-02
GO:0060978	angiogenesis involved in coror	BP	6	1	562	2	19809	5.59E-02
GO:0035717	chemokine (C-C motif) ligand	MF	8	1	562	2	19809	5.59E-02
GO:0002876	positive regulation of chronic i	BP	11	1	562	2	19809	5.59E-02
GO:0047787	delta4-3-oxosteroid 5beta-redu	MF	8	1	562	2	19809	5.59E-02
GO:0061325	cell proliferation involved in o	BP	5	1	562	2	19809	5.59E-02
GO:1905521	regulation of macrophage migi	BP	7	1	562	2	19809	5.59E-02
GO:0099040	ceramide translocation	BP	9	1	562	2	19809	5.59E-02
GO:0045585	positive regulation of cytotoxi	BP	11	1	562	2	19809	5.59E-02
GO:0009826	unidimensional cell growth	BP	7	1	562	2	19809	5.59E-02
GO:0071464	cellular response to hydrostati	BP	6	1	562	2	19809	5.59E-02
GO:0001781	neutrophil apoptotic process	BP	8	1	562	2	19809	5.59E-02
GO:0045029	UDP-activated nucleotide rece	MF	10	1	562	2	19809	5.59E-02
GO:0002774	Fc receptor mediated inhibitor	BP	8	1	562	2	19809	5.59E-02
GO:0019483	beta-alanine biosynthetic proce	BP	10	1	562	2	19809	5.59E-02

GO:0031943	regulation of glucocorticoid m	BP	9	1	562	2	19809	5.59E-02
GO:1904713	beta-catenin destruction compl	MF	5	1	562	2	19809	5.59E-02
GO:1905078	positive regulation of interleuk	BP	9	1	562	2	19809	5.59E-02
GO:0110091	negative regulation of hippoca	BP	11	1	562	2	19809	5.59E-02
GO:0046006	regulation of activated T cell p	BP	11	1	562	2	19809	5.59E-02
GO:0045957	negative regulation of complem	BP	9	1	562	2	19809	5.59E-02
GO:2000525	positive regulation of T cell co	BP	10	1	562	2	19809	5.59E-02
GO:1990646	cellular response to prolactin	BP	8	1	562	2	19809	5.59E-02
GO:2000473	positive regulation of hematop	BP	9	1	562	2	19809	5.59E-02
GO:0071065	alpha9-beta1 integrin-vascular	CC	6	1	562	2	19809	5.59E-02
GO:1903281	positive regulation of calcium:	BP	9	1	562	2	19809	5.59E-02
GO:0051606	detection of stimulus	BP	4	1	562	2	19809	5.59E-02
GO:0007549	dosage compensation	BP	9	1	562	2	19809	5.59E-02
GO:0021988	olfactory lobe development	BP	5	1	562	2	19809	5.59E-02
GO:1903538	regulation of meiotic cell cycle	BP	8	1	562	2	19809	5.59E-02
GO:0032986	protein-DNA complex disasser	BP	7	1	562	2	19809	5.59E-02
GO:1990859	cellular response to endothelin	BP	8	1	562	2	19809	5.59E-02
GO:0002506	polysaccharide assembly with	BP	8	1	562	2	19809	5.59E-02
GO:0051119	sugar transmembrane transport	MF	6	1	562	2	19809	5.59E-02
GO:0004379	glycylpeptide N-tetradecanoylt	MF	8	1	562	2	19809	5.59E-02
GO:0016155	formyltetrahydrofolate dehydro	MF	7	1	562	2	19809	5.59E-02
GO:1904017	cellular response to Thyroglob	BP	6	1	562	2	19809	5.59E-02
GO:0002035	brain renin-angiotensin system	BP	7	1	562	2	19809	5.59E-02
GO:0030350	iron-responsive element bindir	MF	8	1	562	2	19809	5.59E-02
GO:0038190	VEGF-activated neuropilin sig	BP	9	1	562	2	19809	5.59E-02
GO:0051885	positive regulation of timing o	BP	10	1	562	2	19809	5.59E-02
GO:0070699	type II activin receptor binding	MF	9	1	562	2	19809	5.59E-02
GO:0061928	glutathione specific gamma-gl	MF	7	1	562	2	19809	5.59E-02
GO:0007525	somatic muscle development	BP	6	1	562	2	19809	5.59E-02
GO:0045994	positive regulation of translati	BP	12	1	562	2	19809	5.59E-02
GO:0006575	cellular modified amino acid n	BP	5	1	562	2	19809	5.59E-02
GO:0070667	negative regulation of mast cel	BP	9	1	562	2	19809	5.59E-02
GO:0032838	plasma membrane bounded cel	CC	7	1	562	2	19809	5.59E-02
GO:0046331	lateral inhibition	BP	7	1	562	2	19809	5.59E-02
GO:0004698	calcium-dependent protein kin	MF	10	1	562	2	19809	5.59E-02
GO:1902336	positive regulation of retinal g	BP	12	1	562	2	19809	5.59E-02
GO:0070052	collagen V binding	MF	6	1	562	2	19809	5.59E-02
GO:0001855	complement component C4b b	MF	6	1	562	2	19809	5.59E-02
GO:0033883	pyridoxal phosphatase activity	MF	8	1	562	2	19809	5.59E-02
GO:0061368	behavioral response to formali	BP	6	1	562	2	19809	5.59E-02
GO:0051649	establishment of localization in	BP	5	1	562	2	19809	5.59E-02
GO:0045761	regulation of adenylate cyclase	BP	7	1	562	2	19809	5.59E-02
GO:1902947	regulation of tau-protein kinas	BP	11	1	562	2	19809	5.59E-02
GO:0150056	amylin receptor complex 1	CC	9	1	562	2	19809	5.59E-02
GO:0050754	positive regulation of fractalki	BP	10	1	562	2	19809	5.59E-02
GO:1905664	regulation of calcium ion impo	BP	7	1	562	2	19809	5.59E-02
GO:0004157	dihydropyrimidinase activity	MF	7	1	562	2	19809	5.59E-02
GO:0044753	amphisome	CC	8	1	562	2	19809	5.59E-02
GO:0001694	histamine biosynthetic process	BP	10	1	562	2	19809	5.59E-02
GO:0044861	protein transport into plasma n	BP	13	1	562	2	19809	5.59E-02

GO:0035332	positive regulation of hippo sig	BP	10	1	562	2	19809	5.59E-02
GO:0045550	geranylgeranyl reductase activ	MF	5	1	562	2	19809	5.59E-02
GO:2000412	positive regulation of thymocy	BP	10	1	562	2	19809	5.59E-02
GO:0021514	ventral spinal cord interneuron	BP	7	1	562	2	19809	5.59E-02
GO:0006393	termination of mitochondrial tı	BP	10	1	562	2	19809	5.59E-02
GO:0097102	endothelial tip cell fate specifi	BP	8	1	562	2	19809	5.59E-02
GO:0004910	interleukin-1, type II, blocking	MF	8	1	562	2	19809	5.59E-02
GO:0071378	cellular response to growth hoı	BP	8	1	562	2	19809	5.59E-02
GO:2000309	positive regulation of tumor ne	BP	9	1	562	2	19809	5.59E-02
GO:0060557	positive regulation of vitamin I	BP	11	1	562	2	19809	5.59E-02
GO:0030221	basophil differentiation	BP	9	1	562	2	19809	5.59E-02
GO:2000815	regulation of mRNA stability i	BP	12	1	562	2	19809	5.59E-02
GO:0016488	farnesol catabolic process	BP	10	1	562	2	19809	5.59E-02
GO:1990406	CGRP receptor complex	CC	8	1	562	2	19809	5.59E-02
GO:0008768	UDP-sugar diphosphatase activ	MF	8	1	562	2	19809	5.59E-02
GO:0061032	visceral serous pericardium de	BP	5	1	562	2	19809	5.59E-02
GO:0032764	negative regulation of mast cel	BP	9	1	562	2	19809	5.59E-02
GO:1903929	primary palate development	BP	6	1	562	2	19809	5.59E-02
GO:0043000	Golgi to plasma membrane CF	BP	11	1	562	2	19809	5.59E-02
GO:1902023	L-arginine transport	BP	12	1	562	2	19809	5.59E-02
GO:0035606	peptidyl-cysteine S-trans-nitro	BP	11	1	562	2	19809	5.59E-02
GO:0060713	labyrinthine layer morphogene	BP	5	1	562	2	19809	5.59E-02
GO:0042701	progesterone secretion	BP	7	1	562	2	19809	5.59E-02
GO:0060327	cytoplasmic actin-based contra	BP	7	1	562	2	19809	5.59E-02
GO:0004917	interleukin-7 receptor activity	MF	7	1	562	2	19809	5.59E-02
GO:2000981	negative regulation of inner ea	BP	12	1	562	2	19809	5.59E-02
GO:0046459	short-chain fatty acid metaboli	BP	10	1	562	2	19809	5.59E-02
GO:0006678	glucosylceramide metabolic pı	BP	11	1	562	2	19809	5.59E-02
GO:0006681	galactosylceramide metabolic	BP	11	1	562	2	19809	5.59E-02
GO:1905006	negative regulation of epitheli	BP	10	1	562	2	19809	5.59E-02
GO:0048641	regulation of skeletal muscle ti	BP	9	1	562	2	19809	5.59E-02
GO:0002439	chronic inflammatory response	BP	8	1	562	2	19809	5.59E-02
GO:0061441	renal artery morphogenesis	BP	8	1	562	2	19809	5.59E-02
GO:2000418	positive regulation of eosinopł	BP	8	1	562	2	19809	5.59E-02
GO:2001038	regulation of cellular response	BP	6	1	562	2	19809	5.59E-02
GO:0006788	heme oxidation	BP	9	1	562	2	19809	5.59E-02
GO:0019049	evasion or tolerance of host de	BP	8	1	562	2	19809	5.59E-02
GO:0001507	acetylcholine catabolic process	BP	8	1	562	2	19809	5.59E-02
GO:0070838	divalent metal ion transport	BP	9	1	562	2	19809	5.59E-02
GO:0046592	polyamine oxidase activity	MF	7	1	562	2	19809	5.59E-02
GO:0033142	progesterone receptor binding	MF	9	1	562	2	19809	5.59E-02
GO:0010465	nerve growth factor receptor a	MF	6	1	562	2	19809	5.59E-02
GO:1904139	regulation of microglial cell m	BP	11	1	562	2	19809	5.59E-02
GO:0007497	posterior midgut development	BP	5	1	562	2	19809	5.59E-02
GO:2000978	negative regulation of forebrai	BP	11	1	562	2	19809	5.59E-02
GO:0010519	negative regulation of phosphc	BP	9	1	562	2	19809	5.59E-02
GO:0071144	heteromeric SMAD protein co	CC	6	1	562	2	19809	5.59E-02
GO:0002645	positive regulation of toleranc	BP	7	1	562	2	19809	5.59E-02
GO:0014806	smooth muscle hyperplasia	BP	8	1	562	2	19809	5.59E-02
GO:0060279	positive regulation of ovulatio	BP	7	1	562	2	19809	5.59E-02

GO:0021558	trochlear nerve development	BP	7	1	562	2	19809	5.59E-02
GO:1901534	positive regulation of hematop	BP	8	1	562	2	19809	5.59E-02
GO:0002276	basophil activation involved in	BP	8	1	562	2	19809	5.59E-02
GO:0009258	10-formyltetrahydrofolate cata	BP	10	1	562	2	19809	5.59E-02
GO:0015207	adenine transmembrane transp	MF	7	1	562	2	19809	5.59E-02
GO:2000351	regulation of endothelial cell a	BP	10	1	562	2	19809	5.59E-02
GO:0006649	phospholipid transfer to memb	BP	9	1	562	2	19809	5.59E-02
GO:2000866	positive regulation of estradiol	BP	9	1	562	2	19809	5.59E-02
GO:0097021	lymphocyte migration into lym	BP	6	1	562	2	19809	5.59E-02
GO:1902871	positive regulation of amacrine	BP	11	1	562	2	19809	5.59E-02
GO:0048340	paraxial mesoderm morphogen	BP	7	1	562	2	19809	5.59E-02
GO:0061459	L-arginine transmembrane tran	MF	9	1	562	2	19809	5.59E-02
GO:1905289	regulation of CAMKK-AMPK	BP	10	1	562	2	19809	5.59E-02
GO:0031685	adenosine receptor binding	MF	7	1	562	2	19809	5.59E-02
GO:0009450	gamma-aminobutyric acid cata	BP	10	1	562	2	19809	5.59E-02
GO:0014041	regulation of neuron maturatio	BP	10	1	562	2	19809	5.59E-02
GO:0051799	negative regulation of hair foll	BP	8	1	562	2	19809	5.59E-02
GO:0004482	mRNA (guanine-N7-)-methylt	MF	8	1	562	2	19809	5.59E-02
GO:0002752	cell surface pattern recognition	BP	8	1	562	2	19809	5.59E-02
GO:0032127	dense core granule membrane	CC	8	1	562	2	19809	5.59E-02
GO:0047757	chondroitin-glucuronate 5-epim	MF	7	1	562	2	19809	5.59E-02
GO:0060049	regulation of protein glycosyla	BP	9	1	562	2	19809	5.59E-02
GO:1902823	negative regulation of late end	BP	9	1	562	2	19809	5.59E-02
GO:0006753	nucleoside phosphate metaboli	BP	7	1	562	2	19809	5.59E-02
GO:0001806	type IV hypersensitivity	BP	8	1	562	2	19809	5.59E-02
GO:0060302	negative regulation of cytokine	BP	9	1	562	2	19809	5.59E-02
GO:0016404	15-hydroxyprostaglandin dehy	MF	7	1	562	2	19809	5.59E-02
GO:1902608	positive regulation of large co	BP	9	1	562	2	19809	5.59E-02
GO:1903775	regulation of DNA double-stra	BP	11	1	562	2	19809	5.59E-02
GO:0010559	regulation of glycoprotein bios	BP	8	1	562	2	19809	5.59E-02
GO:0031533	mRNA cap methyltransferase c	CC	6	1	562	2	19809	5.59E-02
GO:0052901	spermine:oxygen oxidoreducta	MF	7	1	562	2	19809	5.59E-02
GO:0002537	nitric oxide production involve	BP	5	1	562	2	19809	5.59E-02
GO:0002767	immune response-inhibiting ce	BP	7	1	562	2	19809	5.59E-02
GO:1902257	negative regulation of apoptoti	BP	8	1	562	2	19809	5.59E-02
GO:0046208	spermine catabolic process	BP	11	1	562	2	19809	5.59E-02
GO:0007186	G protein-coupled receptor sig	BP	5	42	562	1150	19809	5.62E-02
GO:0005080	protein kinase C binding	MF	8	4	562	51	19809	5.63E-02
GO:0005901	caveola	CC	8	5	562	73	19809	5.63E-02
GO:1902895	positive regulation of pri-miRN	BP	11	3	562	31	19809	5.68E-02
GO:0002040	sprouting angiogenesis	BP	6	3	562	31	19809	5.68E-02
GO:0044267	cellular protein metabolic proc	BP	7	11	562	225	19809	5.68E-02
GO:0000165	MAPK cascade	BP	11	13	562	280	19809	5.71E-02
GO:0030324	lung development	BP	6	6	562	97	19809	5.78E-02
GO:0042535	positive regulation of tumor ne	BP	10	2	562	14	19809	5.84E-02
GO:0002675	positive regulation of acute inf	BP	10	2	562	14	19809	5.84E-02
GO:0007398	ectoderm development	BP	6	2	562	14	19809	5.84E-02
GO:1902902	negative regulation of autopha	BP	11	2	562	14	19809	5.84E-02
GO:0016242	negative regulation of macroau	BP	10	2	562	14	19809	5.84E-02
GO:1904645	response to amyloid-beta	BP	8	2	562	14	19809	5.84E-02

GO:0071361	cellular response to ethanol	BP	7	2	562	14	19809	5.84E-02
GO:0044130	negative regulation of growth (	BP	7	2	562	14	19809	5.84E-02
GO:0061045	negative regulation of wound l	BP	8	2	562	14	19809	5.84E-02
GO:0005940	septin ring	CC	8	2	562	14	19809	5.84E-02
GO:0032740	positive regulation of interleuk	BP	8	2	562	14	19809	5.84E-02
GO:0051145	smooth muscle cell differentia	BP	7	2	562	14	19809	5.84E-02
GO:0030194	positive regulation of blood co	BP	8	2	562	14	19809	5.84E-02
GO:0021675	nerve development	BP	5	2	562	14	19809	5.84E-02
GO:0050435	amyloid-beta metabolic proces	BP	8	2	562	14	19809	5.84E-02
GO:0033630	positive regulation of cell adhe	BP	7	2	562	14	19809	5.84E-02
GO:0099523	presynaptic cytosol	CC	4	2	562	14	19809	5.84E-02
GO:0042627	chylomicron	CC	5	2	562	14	19809	5.84E-02
GO:0031105	septin complex	CC	4	2	562	14	19809	5.84E-02
GO:0045947	negative regulation of translati	BP	11	2	562	14	19809	5.84E-02
GO:0048185	activin binding	MF	5	2	562	14	19809	5.84E-02
GO:0050804	modulation of chemical synapt	BP	8	5	562	74	19809	5.90E-02
GO:0010466	negative regulation of peptidas	BP	10	7	562	122	19809	5.90E-02
GO:0006968	cellular defense response	BP	6	4	562	52	19809	5.96E-02
GO:0000977	RNA polymerase II regulatory	MF	9	14	562	310	19809	5.97E-02
GO:0048545	response to steroid hormone	BP	6	3	562	32	19809	6.14E-02
GO:0007631	feeding behavior	BP	4	3	562	32	19809	6.14E-02
GO:0005834	heterotrimeric G-protein comp	CC	5	3	562	32	19809	6.14E-02
GO:0030316	osteoclast differentiation	BP	8	3	562	32	19809	6.14E-02
GO:0090102	cochlea development	BP	5	3	562	32	19809	6.14E-02
GO:0001974	blood vessel remodeling	BP	5	3	562	32	19809	6.14E-02
GO:1900745	positive regulation of p38MAI	BP	13	3	562	32	19809	6.14E-02
GO:0060325	face morphogenesis	BP	5	3	562	32	19809	6.14E-02
GO:0004888	transmembrane signaling recep	MF	5	11	562	229	19809	6.28E-02
GO:0099061	integral component of postsyn	CC	6	4	562	53	19809	6.31E-02
GO:0031397	negative regulation of protein	BP	11	4	562	53	19809	6.31E-02
GO:1904813	ficolin-1-rich granule lumen	CC	7	7	562	124	19809	6.33E-02
GO:0030175	filopodium	CC	7	5	562	76	19809	6.47E-02
GO:0045944	positive regulation of transcrip	BP	10	41	562	1133	19809	6.56E-02
GO:0070848	response to growth factor	BP	6	2	562	15	19809	6.61E-02
GO:0070742	C2H2 zinc finger domain bind	MF	6	2	562	15	19809	6.61E-02
GO:0031643	positive regulation of myelinat	BP	7	2	562	15	19809	6.61E-02
GO:0007512	adult heart development	BP	7	2	562	15	19809	6.61E-02
GO:0002526	acute inflammatory response	BP	7	2	562	15	19809	6.61E-02
GO:0022408	negative regulation of cell-cell	BP	7	2	562	15	19809	6.61E-02
GO:2000573	positive regulation of DNA bio	BP	9	2	562	15	19809	6.61E-02
GO:0098719	sodium ion import across plasi	BP	11	2	562	15	19809	6.61E-02
GO:0005024	transforming growth factor bet	MF	10	2	562	15	19809	6.61E-02
GO:0000900	translation repressor activity, r	MF	5	2	562	15	19809	6.61E-02
GO:0050678	regulation of epithelial cell pro	BP	7	2	562	15	19809	6.61E-02
GO:0045618	positive regulation of keratino	BP	10	2	562	15	19809	6.61E-02
GO:0050965	detection of temperature stimu	BP	8	2	562	15	19809	6.61E-02
GO:0004697	protein kinase C activity	MF	9	2	562	15	19809	6.61E-02
GO:0070207	protein homotrimerization	BP	9	3	562	33	19809	6.61E-02
GO:0097421	liver regeneration	BP	8	3	562	33	19809	6.61E-02
GO:0071549	cellular response to dexametha	BP	7	3	562	33	19809	6.61E-02

GO:0043014	alpha-tubulin binding	MF	7	3	562	33	19809	6.61E-02
GO:0003151	outflow tract morphogenesis	BP	5	4	562	54	19809	6.67E-02
GO:0016324	apical plasma membrane	CC	5	14	562	316	19809	6.77E-02
GO:0006486	protein glycosylation	BP	9	9	562	178	19809	6.77E-02
GO:0000139	Golgi membrane	CC	7	25	562	643	19809	7.03E-02
GO:0007599	hemostasis	BP	6	4	562	55	19809	7.04E-02
GO:0071230	cellular response to amino acic	BP	7	4	562	55	19809	7.04E-02
GO:0030170	pyridoxal phosphate binding	MF	6	4	562	55	19809	7.04E-02
GO:0042100	B cell proliferation	BP	8	3	562	34	19809	7.11E-02
GO:0031663	lipopolysaccharide-mediated s	BP	6	3	562	34	19809	7.11E-02
GO:2000352	negative regulation of endothe	BP	11	3	562	34	19809	7.11E-02
GO:0045773	positive regulation of axon ext	BP	8	3	562	34	19809	7.11E-02
GO:0005794	Golgi apparatus	CC	6	46	562	1300	19809	7.16E-02
GO:0006874	cellular calcium ion homeosta:	BP	11	6	562	103	19809	7.29E-02
GO:0004222	metalloendopeptidase activity	MF	8	6	562	103	19809	7.29E-02
GO:1904469	positive regulation of tumor ne	BP	10	2	562	16	19809	7.42E-02
GO:0045778	positive regulation of ossificat	BP	7	2	562	16	19809	7.42E-02
GO:2000679	positive regulation of transcrip	BP	8	2	562	16	19809	7.42E-02
GO:0035729	cellular response to hepatocyte	BP	8	2	562	16	19809	7.42E-02
GO:0034116	positive regulation of heteroty]	BP	8	2	562	16	19809	7.42E-02
GO:0008191	metalloendopeptidase inhibitoı	MF	8	2	562	16	19809	7.42E-02
GO:0023026	MHC class II protein complex	MF	6	2	562	16	19809	7.42E-02
GO:0061028	establishment of endothelial ba	BP	8	2	562	16	19809	7.42E-02
GO:0007342	fusion of sperm to egg plasma	BP	5	2	562	16	19809	7.42E-02
GO:0006309	apoptotic DNA fragmentation	BP	10	2	562	16	19809	7.42E-02
GO:0048489	synaptic vesicle transport	BP	6	2	562	16	19809	7.42E-02
GO:1990869	cellular response to chemokine	BP	8	2	562	16	19809	7.42E-02
GO:0030169	low-density lipoprotein particl	MF	7	2	562	16	19809	7.42E-02
GO:0045910	negative regulation of DNA re	BP	10	2	562	16	19809	7.42E-02
GO:0043525	positive regulation of neuron a	BP	10	4	562	56	19809	7.42E-02
GO:0031532	actin cytoskeleton reorganizati	BP	8	4	562	56	19809	7.42E-02
GO:0007189	adenylate cyclase-activating G	BP	8	6	562	104	19809	7.56E-02
GO:0007179	transforming growth factor bet	BP	8	6	562	104	19809	7.56E-02
GO:0050919	negative chemotaxis	BP	6	3	562	35	19809	7.62E-02
GO:0060349	bone morphogenesis	BP	6	3	562	35	19809	7.62E-02
GO:0015026	coreceptor activity	MF	5	3	562	35	19809	7.62E-02
GO:1902042	negative regulation of extrinsic	BP	11	3	562	35	19809	7.62E-02
GO:0043209	myelin sheath	CC	4	3	562	35	19809	7.62E-02
GO:0030901	midbrain development	BP	5	3	562	35	19809	7.62E-02
GO:0006812	cation transport	BP	7	5	562	80	19809	7.70E-02
GO:0006979	response to oxidative stress	BP	5	7	562	130	19809	7.72E-02
GO:0016266	O-glycan processing	BP	11	4	562	57	19809	7.81E-02
GO:0001503	ossification	BP	4	6	562	105	19809	7.84E-02
GO:0005912	adherens junction	CC	5	5	562	81	19809	8.02E-02
GO:0097110	scaffold protein binding	MF	5	4	562	58	19809	8.21E-02
GO:0042733	embryonic digit morphogenesi	BP	6	4	562	58	19809	8.21E-02
GO:0007200	phospholipase C-activating G]	BP	6	4	562	58	19809	8.21E-02
GO:0071380	cellular response to prostaglan	BP	8	2	562	17	19809	8.25E-02
GO:0043032	positive regulation of macroph	BP	8	2	562	17	19809	8.25E-02
GO:0046965	retinoid X receptor binding	MF	9	2	562	17	19809	8.25E-02

GO:0001655	urogenital system developmen	BP	6	2	562	17	19809	8.25E-02
GO:2000010	positive regulation of protein l	BP	7	2	562	17	19809	8.25E-02
GO:0014002	astrocyte development	BP	7	2	562	17	19809	8.25E-02
GO:0016010	dystrophin-associated glycopro	CC	5	2	562	17	19809	8.25E-02
GO:0006044	N-acetylglucosamine metaboli	BP	8	2	562	17	19809	8.25E-02
GO:0008209	androgen metabolic process	BP	7	2	562	17	19809	8.25E-02
GO:0097038	perinuclear endoplasmic reticu	CC	7	2	562	17	19809	8.25E-02
GO:0046688	response to copper ion	BP	7	2	562	17	19809	8.25E-02
GO:0042326	negative regulation of phospho	BP	10	2	562	17	19809	8.25E-02
GO:0046325	negative regulation of glucose	BP	10	2	562	17	19809	8.25E-02
GO:0048747	muscle fiber development	BP	8	2	562	17	19809	8.25E-02
GO:0030225	macrophage differentiation	BP	8	2	562	17	19809	8.25E-02
GO:1903980	positive regulation of microgli	BP	9	2	562	17	19809	8.25E-02
GO:0015299	solute:proton antiporter activit	MF	10	2	562	17	19809	8.25E-02
GO:0051284	positive regulation of sequeste	BP	7	1	562	3	19809	8.27E-02
GO:0002291	T cell activation via T cell rece	BP	9	1	562	3	19809	8.27E-02
GO:1903352	L-ornithine transmembrane tra	BP	8	1	562	3	19809	8.27E-02
GO:0072061	inner medullary collecting duc	BP	7	1	562	3	19809	8.27E-02
GO:0033007	negative regulation of mast cel	BP	8	1	562	3	19809	8.27E-02
GO:0071475	cellular hyperosmotic salinity	BP	8	1	562	3	19809	8.27E-02
GO:0071626	mastication	BP	6	1	562	3	19809	8.27E-02
GO:0038189	neuropilin signaling pathway	BP	6	1	562	3	19809	8.27E-02
GO:0048017	inositol lipid-mediated signali	BP	6	1	562	3	19809	8.27E-02
GO:0045077	negative regulation of interfero	BP	9	1	562	3	19809	8.27E-02
GO:0046885	regulation of hormone biosynt	BP	8	1	562	3	19809	8.27E-02
GO:0048841	regulation of axon extension i	BP	8	1	562	3	19809	8.27E-02
GO:2001247	positive regulation of phospha	BP	8	1	562	3	19809	8.27E-02
GO:0042668	auditory receptor cell fate dete	BP	7	1	562	3	19809	8.27E-02
GO:2000439	positive regulation of monocyt	BP	9	1	562	3	19809	8.27E-02
GO:0000710	meiotic mismatch repair	BP	10	1	562	3	19809	8.27E-02
GO:1901857	positive regulation of cellular	BP	8	1	562	3	19809	8.27E-02
GO:0021773	striatal medium spiny neuron c	BP	9	1	562	3	19809	8.27E-02
GO:0044346	fibroblast apoptotic process	BP	7	1	562	3	19809	8.27E-02
GO:0005010	insulin-like growth factor-acti	MF	10	1	562	3	19809	8.27E-02
GO:1990752	microtubule end	CC	6	1	562	3	19809	8.27E-02
GO:0038172	interleukin-33-mediated signal	BP	7	1	562	3	19809	8.27E-02
GO:0004013	adenosylhomocysteinase activi	MF	7	1	562	3	19809	8.27E-02
GO:1903765	negative regulation of potassiu	BP	12	1	562	3	19809	8.27E-02
GO:0000822	inositol hexakisphosphate bind	MF	5	1	562	3	19809	8.27E-02
GO:0021553	olfactory nerve development	BP	7	1	562	3	19809	8.27E-02
GO:0001838	embryonic epithelial tube form	BP	7	1	562	3	19809	8.27E-02
GO:0042984	regulation of amyloid precurso	BP	9	1	562	3	19809	8.27E-02
GO:0046103	inosine biosynthetic process	BP	12	1	562	3	19809	8.27E-02
GO:0010693	negative regulation of alkaline	BP	12	1	562	3	19809	8.27E-02
GO:0071283	cellular response to iron(III) io	BP	9	1	562	3	19809	8.27E-02
GO:1903679	positive regulation of cap-inde	BP	13	1	562	3	19809	8.27E-02
GO:0060672	epithelial cell morphogenesis i	BP	5	1	562	3	19809	8.27E-02
GO:0071400	cellular response to oleic acid	BP	8	1	562	3	19809	8.27E-02
GO:1902937	inward rectifier potassium cha	CC	10	1	562	3	19809	8.27E-02
GO:1904373	response to kainic acid	BP	7	1	562	3	19809	8.27E-02

GO:0005128	erythropoietin receptor binding	MF	7	1	562	3	19809	8.27E-02
GO:0032450	maltose alpha-glucosidase acti	MF	9	1	562	3	19809	8.27E-02
GO:2001027	negative regulation of endothe	BP	11	1	562	3	19809	8.27E-02
GO:0051033	RNA transmembrane transport	MF	7	1	562	3	19809	8.27E-02
GO:1902692	regulation of neuroblast prolif	BP	9	1	562	3	19809	8.27E-02
GO:0060675	ureteric bud morphogenesis	BP	11	1	562	3	19809	8.27E-02
GO:0045023	G0 to G1 transition	BP	5	1	562	3	19809	8.27E-02
GO:0045619	regulation of lymphocyte diffe	BP	8	1	562	3	19809	8.27E-02
GO:1904761	negative regulation of myofibr	BP	8	1	562	3	19809	8.27E-02
GO:0019471	4-hydroxyproline metabolic pr	BP	6	1	562	3	19809	8.27E-02
GO:0048101	calcium- and calmodulin-regul	MF	11	1	562	3	19809	8.27E-02
GO:2001258	negative regulation of cation c	BP	8	1	562	3	19809	8.27E-02
GO:0002068	glandular epithelial cell develc	BP	8	1	562	3	19809	8.27E-02
GO:2001204	regulation of osteoclast develo	BP	10	1	562	3	19809	8.27E-02
GO:0097487	multivesicular body, internal v	CC	7	1	562	3	19809	8.27E-02
GO:0046061	dATP catabolic process	BP	12	1	562	3	19809	8.27E-02
GO:0005944	phosphatidylinositol 3-kinase c	CC	9	1	562	3	19809	8.27E-02
GO:0060164	regulation of timing of neuron	BP	10	1	562	3	19809	8.27E-02
GO:0019482	beta-alanine metabolic process	BP	9	1	562	3	19809	8.27E-02
GO:0051654	establishment of mitochondrio	BP	7	1	562	3	19809	8.27E-02
GO:0045058	T cell selection	BP	4	1	562	3	19809	8.27E-02
GO:0098900	regulation of action potential	BP	6	1	562	3	19809	8.27E-02
GO:0002408	myeloid dendritic cell chemota	BP	7	1	562	3	19809	8.27E-02
GO:1905747	negative regulation of saliva se	BP	9	1	562	3	19809	8.27E-02
GO:1904999	positive regulation of leukocyt	BP	10	1	562	3	19809	8.27E-02
GO:2000172	regulation of branching morph	BP	8	1	562	3	19809	8.27E-02
GO:0032929	negative regulation of superox	BP	10	1	562	3	19809	8.27E-02
GO:2000078	positive regulation of type B p	BP	9	1	562	3	19809	8.27E-02
GO:0047844	deoxycytidine deaminase activ	MF	7	1	562	3	19809	8.27E-02
GO:0004912	interleukin-3 receptor activity	MF	7	1	562	3	19809	8.27E-02
GO:0050955	thermoception	BP	8	1	562	3	19809	8.27E-02
GO:0061092	positive regulation of phospho	BP	8	1	562	3	19809	8.27E-02
GO:0042625	ATPase coupled ion transmem	MF	6	1	562	3	19809	8.27E-02
GO:1904928	coreceptor activity involved in	MF	7	1	562	3	19809	8.27E-02
GO:0044308	axonal spine	CC	6	1	562	3	19809	8.27E-02
GO:0003383	apical constriction	BP	7	1	562	3	19809	8.27E-02
GO:1902803	regulation of synaptic vesicle t	BP	7	1	562	3	19809	8.27E-02
GO:0030859	polarized epithelial cell differe	BP	7	1	562	3	19809	8.27E-02
GO:0047045	testosterone 17-beta-dehydrog	MF	7	1	562	3	19809	8.27E-02
GO:0106005	RNA 5'-cap (guanine-N7)-met	BP	12	1	562	3	19809	8.27E-02
GO:0048625	myoblast fate commitment	BP	6	1	562	3	19809	8.27E-02
GO:0002793	positive regulation of peptide s	BP	9	1	562	3	19809	8.27E-02
GO:0045959	negative regulation of comple	BP	13	1	562	3	19809	8.27E-02
GO:0032916	positive regulation of transfor	BP	9	1	562	3	19809	8.27E-02
GO:0030538	embryonic genitalia morphoge	BP	6	1	562	3	19809	8.27E-02
GO:0071492	cellular response to UV-A	BP	9	1	562	3	19809	8.27E-02
GO:1902309	negative regulation of peptidyl	BP	11	1	562	3	19809	8.27E-02
GO:0035745	T-helper 2 cell cytokine produ	BP	8	1	562	3	19809	8.27E-02
GO:2001140	positive regulation of phospho	BP	9	1	562	3	19809	8.27E-02
GO:0002476	antigen processing and present	BP	7	1	562	3	19809	8.27E-02

GO:0033745	L-methionine-(R)-S-oxide redu	MF	7	1	562	3	19809	8.27E-02
GO:0047804	cysteine-S-conjugate beta-lyas	MF	6	1	562	3	19809	8.27E-02
GO:0002605	negative regulation of dendriti	BP	8	1	562	3	19809	8.27E-02
GO:0010046	response to mycotoxin	BP	6	1	562	3	19809	8.27E-02
GO:0050711	negative regulation of interleul	BP	9	1	562	3	19809	8.27E-02
GO:0060914	heart formation	BP	6	1	562	3	19809	8.27E-02
GO:0071871	response to epinephrine	BP	8	1	562	3	19809	8.27E-02
GO:0005030	neurotrophin receptor activity	MF	5	1	562	3	19809	8.27E-02
GO:0004558	alpha-1,4-glucosidase activity	MF	9	1	562	3	19809	8.27E-02
GO:0060559	positive regulation of calcidiol	BP	9	1	562	3	19809	8.27E-02
GO:0009595	detection of biotic stimulus	BP	5	1	562	3	19809	8.27E-02
GO:0006154	adenosine catabolic process	BP	7	1	562	3	19809	8.27E-02
GO:0043308	eosinophil degranulation	BP	9	1	562	3	19809	8.27E-02
GO:0010751	negative regulation of nitric ox	BP	10	1	562	3	19809	8.27E-02
GO:0150020	basal dendrite arborization	BP	11	1	562	3	19809	8.27E-02
GO:0070012	oligopeptidase activity	MF	6	1	562	3	19809	8.27E-02
GO:0043420	anthranilate metabolic process	BP	7	1	562	3	19809	8.27E-02
GO:0044773	mitotic DNA damage checkpo	BP	8	1	562	3	19809	8.27E-02
GO:0047044	androstan-3-alpha,17-beta-diol	MF	7	1	562	3	19809	8.27E-02
GO:0000052	citrulline metabolic process	BP	10	1	562	3	19809	8.27E-02
GO:0097447	dendritic tree	CC	6	1	562	3	19809	8.27E-02
GO:0042610	CD8 receptor binding	MF	6	1	562	3	19809	8.27E-02
GO:1990408	calcitonin gene-related peptide	BP	7	1	562	3	19809	8.27E-02
GO:0003994	aconitate hydratase activity	MF	7	1	562	3	19809	8.27E-02
GO:0002374	cytokine secretion involved in	BP	12	1	562	3	19809	8.27E-02
GO:0035880	embryonic nail plate morphoge	BP	6	1	562	3	19809	8.27E-02
GO:0030880	RNA polymerase complex	CC	5	1	562	3	19809	8.27E-02
GO:0046882	negative regulation of follicle-	BP	10	1	562	3	19809	8.27E-02
GO:0002118	aggressive behavior	BP	5	1	562	3	19809	8.27E-02
GO:0042978	ornithine decarboxylase activa	MF	6	1	562	3	19809	8.27E-02
GO:0019087	transformation of host cell by	BP	8	1	562	3	19809	8.27E-02
GO:0097454	Schwann cell microvillus	CC	8	1	562	3	19809	8.27E-02
GO:0048634	regulation of muscle organ dev	BP	7	1	562	3	19809	8.27E-02
GO:0044406	adhesion of symbiont to host	BP	4	1	562	3	19809	8.27E-02
GO:0051795	positive regulation of timing o	BP	10	1	562	3	19809	8.27E-02
GO:1900005	positive regulation of serine-ty	BP	12	1	562	3	19809	8.27E-02
GO:0072282	metanephric nephron tubule m	BP	10	1	562	3	19809	8.27E-02
GO:1905684	regulation of plasma membran	BP	9	1	562	3	19809	8.27E-02
GO:0052851	ferric-chelate reductase (NAD	MF	7	1	562	3	19809	8.27E-02
GO:0017159	pantetheine hydrolase activity	MF	7	1	562	3	19809	8.27E-02
GO:1900449	regulation of glutamate recepto	BP	8	1	562	3	19809	8.27E-02
GO:0038179	neurotrophin signaling pathwa	BP	6	1	562	3	19809	8.27E-02
GO:0008823	cupric reductase activity	MF	7	1	562	3	19809	8.27E-02
GO:0004047	aminomethyltransferase activit	MF	7	1	562	3	19809	8.27E-02
GO:1901491	negative regulation of lymphar	BP	8	1	562	3	19809	8.27E-02
GO:0018636	phenanthrene 9,10-monooxyge	MF	7	1	562	3	19809	8.27E-02
GO:0061009	common bile duct developmen	BP	6	1	562	3	19809	8.27E-02
GO:0042450	arginine biosynthetic process v	BP	13	1	562	3	19809	8.27E-02
GO:2000974	negative regulation of pro-B ce	BP	10	1	562	3	19809	8.27E-02
GO:0030070	insulin processing	BP	9	1	562	3	19809	8.27E-02

GO:0006598	polyamine catabolic process	BP	10	1	562	3	19809	8.27E-02
GO:0047655	allyl-alcohol dehydrogenase ac	MF	7	1	562	3	19809	8.27E-02
GO:0001730	2'-5'-oligoadenylate synthetase	MF	8	1	562	3	19809	8.27E-02
GO:0001069	regulatory region RNA binding	MF	7	1	562	3	19809	8.27E-02
GO:0048382	mesendoderm development	BP	5	1	562	3	19809	8.27E-02
GO:1905748	hard palate morphogenesis	BP	5	1	562	3	19809	8.27E-02
GO:1903413	cellular response to bile acid	BP	7	1	562	3	19809	8.27E-02
GO:0060201	clathrin-sculpted acetylcholine	CC	8	1	562	3	19809	8.27E-02
GO:0002457	T cell antigen processing and p	BP	5	1	562	3	19809	8.27E-02
GO:0051280	negative regulation of release o	BP	8	1	562	3	19809	8.27E-02
GO:0007509	mesoderm migration involved	BP	9	1	562	3	19809	8.27E-02
GO:0003829	beta-1,3-galactosyl-O-glycosyl	MF	8	1	562	3	19809	8.27E-02
GO:0002486	antigen processing and present	BP	9	1	562	3	19809	8.27E-02
GO:0009448	gamma-aminobutyric acid met	BP	9	1	562	3	19809	8.27E-02
GO:0042269	regulation of natural killer cell	BP	7	1	562	3	19809	8.27E-02
GO:0002689	negative regulation of leukocy	BP	8	1	562	3	19809	8.27E-02
GO:0032800	receptor biosynthetic process	BP	7	1	562	3	19809	8.27E-02
GO:0005996	monosaccharide metabolic pro	BP	6	1	562	3	19809	8.27E-02
GO:0010758	regulation of macrophage cher	BP	8	1	562	3	19809	8.27E-02
GO:0046879	hormone secretion	BP	7	1	562	3	19809	8.27E-02
GO:0090259	regulation of retinal ganglion c	BP	11	1	562	3	19809	8.27E-02
GO:0021882	regulation of transcription fror	BP	11	1	562	3	19809	8.27E-02
GO:0050717	positive regulation of interleuk	BP	10	1	562	3	19809	8.27E-02
GO:0051248	negative regulation of protein r	BP	8	1	562	3	19809	8.27E-02
GO:0035276	ethanol binding	MF	5	1	562	3	19809	8.27E-02
GO:0061146	Peyer's patch morphogenesis	BP	6	1	562	3	19809	8.27E-02
GO:1905114	cell surface receptor signaling	BP	6	1	562	3	19809	8.27E-02
GO:0004875	complement receptor activity	MF	6	1	562	3	19809	8.27E-02
GO:0070735	protein-glycine ligase activity	MF	7	1	562	3	19809	8.27E-02
GO:0045925	positive regulation of female re	BP	8	1	562	3	19809	8.27E-02
GO:0032485	regulation of Ral protein signa	BP	11	1	562	3	19809	8.27E-02
GO:0097638	L-arginine import across plasn	BP	13	1	562	3	19809	8.27E-02
GO:0008109	N-acetyllactosaminide beta-1,6	MF	8	1	562	3	19809	8.27E-02
GO:0008597	calcium-dependent protein seri	MF	7	1	562	3	19809	8.27E-02
GO:0044393	microspike	CC	6	1	562	3	19809	8.27E-02
GO:0019805	quinolinate biosynthetic proce:	BP	10	1	562	3	19809	8.27E-02
GO:0050912	detection of chemical stimulus	BP	7	1	562	3	19809	8.27E-02
GO:0019254	carnitine metabolic process, Co	BP	8	1	562	3	19809	8.27E-02
GO:0072300	positive regulation of metanep	BP	10	1	562	3	19809	8.27E-02
GO:0003278	apoptotic process involved in l	BP	9	1	562	3	19809	8.27E-02
GO:0032142	single guanine insertion bindir	MF	11	1	562	3	19809	8.27E-02
GO:0035809	regulation of urine volume	BP	6	1	562	3	19809	8.27E-02
GO:0045608	negative regulation of inner ea	BP	8	1	562	3	19809	8.27E-02
GO:0071074	eukaryotic initiation factor eIF	MF	6	1	562	3	19809	8.27E-02
GO:0045054	constitutive secretory pathway	BP	8	1	562	3	19809	8.27E-02
GO:0098583	learned vocalization behavior	BP	5	1	562	3	19809	8.27E-02
GO:0000023	maltose metabolic process	BP	8	1	562	3	19809	8.27E-02
GO:0047718	indanol dehydrogenase activity	MF	7	1	562	3	19809	8.27E-02
GO:1904693	midbrain morphogenesis	BP	5	1	562	3	19809	8.27E-02
GO:0004117	calmodulin-dependent cyclic-n	MF	10	1	562	3	19809	8.27E-02

GO:0005220	inositol 1,4,5-trisphosphate-sei	MF	10	1	562	3	19809	8.27E-02
GO:0060693	regulation of branching involv	BP	8	1	562	3	19809	8.27E-02
GO:0072049	comma-shaped body morphogo	BP	5	1	562	3	19809	8.27E-02
GO:0098706	iron ion import across cell outo	BP	12	1	562	3	19809	8.27E-02
GO:0006203	dGTP catabolic process	BP	12	1	562	3	19809	8.27E-02
GO:0071379	cellular response to prostaglan	BP	7	1	562	3	19809	8.27E-02
GO:0002503	peptide antigen assembly with	BP	9	1	562	3	19809	8.27E-02
GO:0071691	cardiac muscle thin filament as	BP	7	1	562	3	19809	8.27E-02
GO:0051684	maintenance of Golgi location	BP	7	1	562	3	19809	8.27E-02
GO:0097053	L-kynurenine catabolic process	BP	7	1	562	3	19809	8.27E-02
GO:1901671	positive regulation of superoxi	BP	8	1	562	3	19809	8.27E-02
GO:0071363	cellular response to growth fac	BP	7	4	562	59	19809	8.62E-02
GO:0006953	acute-phase response	BP	8	3	562	37	19809	8.68E-02
GO:0007498	mesoderm development	BP	6	3	562	37	19809	8.68E-02
GO:0001772	immunological synapse	CC	5	3	562	37	19809	8.68E-02
GO:0017147	Wnt-protein binding	MF	5	3	562	37	19809	8.68E-02
GO:0042056	chemoattractant activity	MF	7	3	562	37	19809	8.68E-02
GO:0051289	protein homotetramerization	BP	9	5	562	83	19809	8.70E-02
GO:0071356	cellular response to tumor necı	BP	8	7	562	134	19809	8.74E-02
GO:0009615	response to virus	BP	7	6	562	109	19809	9.00E-02
GO:0061001	regulation of dendritic spine m	BP	10	2	562	18	19809	9.12E-02
GO:0044849	estrous cycle	BP	5	2	562	18	19809	9.12E-02
GO:0071318	cellular response to ATP	BP	9	2	562	18	19809	9.12E-02
GO:0071360	cellular response to exogenous	BP	9	2	562	18	19809	9.12E-02
GO:0001818	negative regulation of cytokine	BP	7	2	562	18	19809	9.12E-02
GO:0048598	embryonic morphogenesis	BP	5	2	562	18	19809	9.12E-02
GO:2000114	regulation of establishment of	BP	7	2	562	18	19809	9.12E-02
GO:0045780	positive regulation of bone res	BP	9	2	562	18	19809	9.12E-02
GO:0010529	negative regulation of transpos	BP	7	2	562	18	19809	9.12E-02
GO:0004866	endopeptidase inhibitor activit	MF	7	3	562	38	19809	9.24E-02
GO:0034614	cellular response to reactive ox	BP	7	3	562	38	19809	9.24E-02
GO:0007163	establishment or maintenance o	BP	4	3	562	38	19809	9.24E-02
GO:0072659	protein localization to plasma r	BP	8	7	562	136	19809	9.27E-02
GO:0006631	fatty acid metabolic process	BP	9	8	562	163	19809	9.33E-02
GO:0071345	cellular response to cytokine st	BP	7	4	562	61	19809	9.47E-02
GO:0043406	positive regulation of MAP kir	BP	12	4	562	61	19809	9.47E-02
GO:0048013	ephrin receptor signaling pathv	BP	8	5	562	86	19809	9.76E-02
GO:0008219	cell death	BP	4	3	562	39	19809	9.81E-02
GO:0032781	positive regulation of ATPase	BP	8	3	562	39	19809	9.81E-02
GO:0006006	glucose metabolic process	BP	8	4	562	62	19809	9.91E-02
GO:0042826	histone deacetylase binding	MF	6	6	562	112	19809	9.93E-02
GO:0007520	myoblast fusion	BP	6	2	562	19	19809	1.00E-01
GO:0002076	osteoblast development	BP	6	2	562	19	19809	1.00E-01
GO:0033280	response to vitamin D	BP	7	2	562	19	19809	1.00E-01
GO:0008157	protein phosphatase 1 binding	MF	8	2	562	19	19809	1.00E-01
GO:0010038	response to metal ion	BP	6	2	562	19	19809	1.00E-01
GO:0008340	determination of adult lifespan	BP	4	2	562	19	19809	1.00E-01
GO:0007263	nitric oxide mediated signal tra	BP	7	2	562	19	19809	1.00E-01
GO:0004653	polypeptide N-acetylgalactosai	MF	8	2	562	19	19809	1.00E-01
GO:0060252	positive regulation of glial cell	BP	8	2	562	19	19809	1.00E-01

GO:0030150	protein import into mitochondı	BP	12	2	562	19	19809	1.00E-01
GO:0031668	cellular response to extracellul	BP	5	2	562	19	19809	1.00E-01
GO:0006869	lipid transport	BP	7	7	562	139	19809	1.01E-01
GO:0030509	BMP signaling pathway	BP	10	5	562	87	19809	1.01E-01
GO:0051607	defense response to virus	BP	5	9	562	194	19809	1.02E-01
GO:0035019	somatic stem cell population n	BP	5	4	562	63	19809	1.04E-01
GO:0034446	substrate adhesion-dependent (	BP	8	3	562	40	19809	1.04E-01
GO:0070491	repressing transcription factor	MF	7	3	562	40	19809	1.04E-01
GO:0030217	T cell differentiation	BP	8	3	562	40	19809	1.04E-01
GO:0090263	positive regulation of canonica	BP	10	7	562	141	19809	1.07E-01
GO:0042472	inner ear morphogenesis	BP	6	4	562	64	19809	1.08E-01
GO:0050839	cell adhesion molecule binding	MF	5	4	562	64	19809	1.08E-01
GO:0051965	positive regulation of synapse	BP	7	4	562	64	19809	1.08E-01
GO:0008349	MAP kinase kinase kinase kin	MF	9	1	562	4	19809	1.09E-01
GO:0060676	ureteric bud formation	BP	10	1	562	4	19809	1.09E-01
GO:0102345	3-hydroxy-lignoceroyl-CoA de	MF	7	1	562	4	19809	1.09E-01
GO:1905643	positive regulation of DNA me	BP	10	1	562	4	19809	1.09E-01
GO:0006931	substrate-dependent cell migra	BP	6	1	562	4	19809	1.09E-01
GO:0003839	gamma-glutamylcyclotransfera	MF	7	1	562	4	19809	1.09E-01
GO:0097643	amylin receptor activity	MF	8	1	562	4	19809	1.09E-01
GO:0090599	alpha-glucosidase activity	MF	8	1	562	4	19809	1.09E-01
GO:0097367	carbohydrate derivative bindin	MF	4	1	562	4	19809	1.09E-01
GO:0071395	cellular response to jasmonic a	BP	7	1	562	4	19809	1.09E-01
GO:0006546	glycine catabolic process	BP	12	1	562	4	19809	1.09E-01
GO:0051707	response to other organism	BP	6	1	562	4	19809	1.09E-01
GO:0003274	endocardial cushion fusion	BP	6	1	562	4	19809	1.09E-01
GO:1903238	positive regulation of leukocyt	BP	9	1	562	4	19809	1.09E-01
GO:0035751	regulation of lysosomal lumen	BP	13	1	562	4	19809	1.09E-01
GO:1905049	negative regulation of metallo	BP	11	1	562	4	19809	1.09E-01
GO:1901078	negative regulation of relaxatic	BP	7	1	562	4	19809	1.09E-01
GO:0060534	trachea cartilage development	BP	7	1	562	4	19809	1.09E-01
GO:1900245	positive regulation of MDA-5	BP	8	1	562	4	19809	1.09E-01
GO:0051098	regulation of binding	BP	5	1	562	4	19809	1.09E-01
GO:2000666	negative regulation of interleul	BP	9	1	562	4	19809	1.09E-01
GO:0015734	taurine transport	BP	10	1	562	4	19809	1.09E-01
GO:0007161	calcium-independent cell-matr	BP	7	1	562	4	19809	1.09E-01
GO:0050974	detection of mechanical stimul	BP	6	1	562	4	19809	1.09E-01
GO:0001880	Mullerian duct regression	BP	5	1	562	4	19809	1.09E-01
GO:1903278	positive regulation of sodium i	BP	12	1	562	4	19809	1.09E-01
GO:0070384	Harderian gland development	BP	7	1	562	4	19809	1.09E-01
GO:0004982	N-formyl peptide receptor acti	MF	7	1	562	4	19809	1.09E-01
GO:0051400	BH domain binding	MF	6	1	562	4	19809	1.09E-01
GO:0021575	hindbrain morphogenesis	BP	5	1	562	4	19809	1.09E-01
GO:0021555	midbrain-hindbrain boundary ı	BP	6	1	562	4	19809	1.09E-01
GO:0004112	cyclic-nucleotide phosphodies	MF	8	1	562	4	19809	1.09E-01
GO:1901837	negative regulation of transcrip	BP	11	1	562	4	19809	1.09E-01
GO:0070884	regulation of calcineurin-NFA	BP	11	1	562	4	19809	1.09E-01
GO:0031587	positive regulation of inositol	BP	11	1	562	4	19809	1.09E-01
GO:0102420	sn-1-glycerol-3-phosphate C16	MF	7	1	562	4	19809	1.09E-01
GO:0060923	cardiac muscle cell fate comm	BP	7	1	562	4	19809	1.09E-01

GO:0030947	regulation of vascular endothe	BP	8	1	562	4	19809	1.09E-01
GO:1901299	negative regulation of hydroge	BP	9	1	562	4	19809	1.09E-01
GO:0001515	opioid peptide activity	MF	7	1	562	4	19809	1.09E-01
GO:0060203	clathrin-sculpted glutamate tra	CC	8	1	562	4	19809	1.09E-01
GO:0015853	adenine transport	BP	9	1	562	4	19809	1.09E-01
GO:0030730	sequestering of triglyceride	BP	6	1	562	4	19809	1.09E-01
GO:0035166	post-embryonic hemopoiesis	BP	8	1	562	4	19809	1.09E-01
GO:0036336	dendritic cell migration	BP	5	1	562	4	19809	1.09E-01
GO:0048105	establishment of body hair pla	BP	8	1	562	4	19809	1.09E-01
GO:1905867	epididymis development	BP	6	1	562	4	19809	1.09E-01
GO:0031014	troponin T binding	MF	6	1	562	4	19809	1.09E-01
GO:0010724	regulation of definitive erythrc	BP	9	1	562	4	19809	1.09E-01
GO:0016807	cysteine-type carboxypeptidas	MF	9	1	562	4	19809	1.09E-01
GO:0042168	heme metabolic process	BP	8	1	562	4	19809	1.09E-01
GO:0051450	myoblast proliferation	BP	4	1	562	4	19809	1.09E-01
GO:0006499	N-terminal protein myristoylat	BP	11	1	562	4	19809	1.09E-01
GO:0023029	MHC class Ib protein binding	MF	7	1	562	4	19809	1.09E-01
GO:0001635	calcitonin gene-related peptide	MF	8	1	562	4	19809	1.09E-01
GO:0070820	tertiary granule	CC	9	1	562	4	19809	1.09E-01
GO:0071896	protein localization to adheren	BP	7	1	562	4	19809	1.09E-01
GO:0045833	negative regulation of lipid me	BP	7	1	562	4	19809	1.09E-01
GO:0097296	activation of cysteine-type end	BP	12	1	562	4	19809	1.09E-01
GO:0002155	regulation of thyroid hormone	BP	8	1	562	4	19809	1.09E-01
GO:0015770	sucrose transport	BP	10	1	562	4	19809	1.09E-01
GO:0005471	ATP:ADP antiporter activity	MF	10	1	562	4	19809	1.09E-01
GO:0021892	cerebral cortex GABAergic int	BP	9	1	562	4	19809	1.09E-01
GO:0002826	negative regulation of T-helpe	BP	10	1	562	4	19809	1.09E-01
GO:0140058	neuron projection arborization	BP	9	1	562	4	19809	1.09E-01
GO:0016508	long-chain-enoyl-CoA hydrata	MF	7	1	562	4	19809	1.09E-01
GO:0031628	opioid receptor binding	MF	7	1	562	4	19809	1.09E-01
GO:0003186	tricuspid valve morphogenesis	BP	7	1	562	4	19809	1.09E-01
GO:0030888	regulation of B cell proliferatic	BP	10	1	562	4	19809	1.09E-01
GO:0000818	nuclear MIS12/MIND comple:	CC	5	1	562	4	19809	1.09E-01
GO:0016888	endodeoxyribonuclease activit	MF	9	1	562	4	19809	1.09E-01
GO:0050265	RNA uridylyltransferase activi	MF	8	1	562	4	19809	1.09E-01
GO:0010748	negative regulation of long-cha	BP	11	1	562	4	19809	1.09E-01
GO:0060513	prostatic bud formation	BP	8	1	562	4	19809	1.09E-01
GO:0010916	negative regulation of very-lov	BP	8	1	562	4	19809	1.09E-01
GO:0002051	osteoblast fate commitment	BP	6	1	562	4	19809	1.09E-01
GO:0102344	3-hydroxy-behenoyl-CoA dehy	MF	7	1	562	4	19809	1.09E-01
GO:0005124	scavenger receptor binding	MF	6	1	562	4	19809	1.09E-01
GO:0097013	phagocytic vesicle lumen	CC	8	1	562	4	19809	1.09E-01
GO:0019693	ribose phosphate metabolic pro	BP	7	1	562	4	19809	1.09E-01
GO:0072050	S-shaped body morphogenesis	BP	5	1	562	4	19809	1.09E-01
GO:0030943	mitochondrion targeting seque	MF	7	1	562	4	19809	1.09E-01
GO:0032376	positive regulation of choleste	BP	10	1	562	4	19809	1.09E-01
GO:0071677	positive regulation of mononuc	BP	8	1	562	4	19809	1.09E-01
GO:1905166	negative regulation of lysosom	BP	11	1	562	4	19809	1.09E-01
GO:1904894	positive regulation of receptor	BP	9	1	562	4	19809	1.09E-01
GO:0003352	regulation of cilium movement	BP	8	1	562	4	19809	1.09E-01

GO:0030055	cell-substrate junction	CC	4	1	562	4	19809	1.09E-01
GO:0033353	S-adenosylmethionine cycle	BP	6	1	562	4	19809	1.09E-01
GO:0016048	detection of temperature stimu	BP	6	1	562	4	19809	1.09E-01
GO:0061333	renal tubule morphogenesis	BP	8	1	562	4	19809	1.09E-01
GO:1902951	negative regulation of dendriti	BP	9	1	562	4	19809	1.09E-01
GO:0060161	positive regulation of dopamin	BP	10	1	562	4	19809	1.09E-01
GO:0043218	compact myelin	CC	4	1	562	4	19809	1.09E-01
GO:0035910	ascending aorta morphogenesi	BP	5	1	562	4	19809	1.09E-01
GO:0060665	regulation of branching involv	BP	7	1	562	4	19809	1.09E-01
GO:0022028	tangential migration from the s	BP	9	1	562	4	19809	1.09E-01
GO:0090330	regulation of platelet aggregati	BP	9	1	562	4	19809	1.09E-01
GO:0072012	glomerulus vasculature develo	BP	6	1	562	4	19809	1.09E-01
GO:1905232	cellular response to L-glutama	BP	9	1	562	4	19809	1.09E-01
GO:1990696	USH2 complex	CC	4	1	562	4	19809	1.09E-01
GO:1903845	negative regulation of cellular	BP	8	1	562	4	19809	1.09E-01
GO:0015189	L-lysine transmembrane transp	MF	7	1	562	4	19809	1.09E-01
GO:0045604	regulation of epidermal cell di	BP	8	1	562	4	19809	1.09E-01
GO:0016361	activin receptor activity, type I	MF	11	1	562	4	19809	1.09E-01
GO:0032375	negative regulation of choleste	BP	10	1	562	4	19809	1.09E-01
GO:0071409	cellular response to cyclohexir	BP	7	1	562	4	19809	1.09E-01
GO:0035799	ureter maturation	BP	7	1	562	4	19809	1.09E-01
GO:0018119	peptidyl-cysteine S-nitrosylati	BP	10	1	562	4	19809	1.09E-01
GO:0016080	synaptic vesicle targeting	BP	5	1	562	4	19809	1.09E-01
GO:0018094	protein polyglycylation	BP	11	1	562	4	19809	1.09E-01
GO:0048368	lateral mesoderm development	BP	7	1	562	4	19809	1.09E-01
GO:0014802	terminal cisterna	CC	7	1	562	4	19809	1.09E-01
GO:0047115	trans-1,2-dihydrobenzene-1,2-	MF	7	1	562	4	19809	1.09E-01
GO:0008506	sucrose:proton symporter activ	MF	8	1	562	4	19809	1.09E-01
GO:0038165	oncostatin-M-mediated signali	BP	7	1	562	4	19809	1.09E-01
GO:0035585	calcium-mediated signaling us	BP	8	1	562	4	19809	1.09E-01
GO:0042534	regulation of tumor necrosis fa	BP	9	1	562	4	19809	1.09E-01
GO:0051712	positive regulation of killing o	BP	7	1	562	4	19809	1.09E-01
GO:0051414	response to cortisol	BP	6	1	562	4	19809	1.09E-01
GO:0035112	genitalia morphogenesis	BP	5	1	562	4	19809	1.09E-01
GO:0004619	phosphoglycerate mutase activ	MF	7	1	562	4	19809	1.09E-01
GO:0043121	neurotrophin binding	MF	6	1	562	4	19809	1.09E-01
GO:0042296	ISG15 transferase activity	MF	6	1	562	4	19809	1.09E-01
GO:0102343	3-hydroxy-arachidoyl-CoA del	MF	7	1	562	4	19809	1.09E-01
GO:0071359	cellular response to dsRNA	BP	8	1	562	4	19809	1.09E-01
GO:0038162	erythropoietin-mediated signal	BP	7	1	562	4	19809	1.09E-01
GO:0009264	deoxyribonucleotide catabolic	BP	10	1	562	4	19809	1.09E-01
GO:0050867	positive regulation of cell activ	BP	7	1	562	4	19809	1.09E-01
GO:0033031	positive regulation of neutroph	BP	7	1	562	4	19809	1.09E-01
GO:0071733	transcriptional activation by pr	BP	11	1	562	4	19809	1.09E-01
GO:0003253	cardiac neural crest cell migrat	BP	9	1	562	4	19809	1.09E-01
GO:0102158	very-long-chain 3-hydroxyacyl	MF	7	1	562	4	19809	1.09E-01
GO:0060159	regulation of dopamine recept	BP	9	1	562	4	19809	1.09E-01
GO:0034767	positive regulation of ion trans	BP	9	1	562	4	19809	1.09E-01
GO:0010730	negative regulation of hydroge	BP	10	1	562	4	19809	1.09E-01
GO:0015181	arginine transmembrane transp	MF	7	1	562	4	19809	1.09E-01

GO:0090554	phosphatidylcholine-translocat	MF	7	1	562	4	19809	1.09E-01
GO:0048742	regulation of skeletal muscle fi	BP	10	1	562	4	19809	1.09E-01
GO:0004082	bisphosphoglycerate mutase ac	MF	7	1	562	4	19809	1.09E-01
GO:0042904	9-cis-retinoic acid biosynthetic	BP	12	1	562	4	19809	1.09E-01
GO:0016447	somatic recombination of imm	BP	6	1	562	4	19809	1.09E-01
GO:0010041	response to iron(III) ion	BP	8	1	562	4	19809	1.09E-01
GO:0010521	telomerase inhibitor activity	MF	6	1	562	4	19809	1.09E-01
GO:0003219	cardiac right ventricle formatic	BP	7	1	562	4	19809	1.09E-01
GO:0060700	regulation of ribonuclease acti	BP	9	1	562	4	19809	1.09E-01
GO:0060355	positive regulation of cell adhe	BP	7	1	562	4	19809	1.09E-01
GO:0043242	negative regulation of protein c	BP	8	1	562	4	19809	1.09E-01
GO:0070779	D-aspartate import across plas	BP	13	1	562	4	19809	1.09E-01
GO:0032909	regulation of transforming gro	BP	8	1	562	4	19809	1.09E-01
GO:1905477	positive regulation of protein l	BP	7	1	562	4	19809	1.09E-01
GO:0033743	peptide-methionine (R)-S-oxid	MF	7	1	562	4	19809	1.09E-01
GO:0035089	establishment of apical/basal c	BP	7	1	562	4	19809	1.09E-01
GO:0060244	negative regulation of cell prol	BP	8	1	562	4	19809	1.09E-01
GO:0051791	medium-chain fatty acid metab	BP	10	1	562	4	19809	1.09E-01
GO:0048713	regulation of oligodendrocyte c	BP	11	1	562	4	19809	1.09E-01
GO:0016742	hydroxymethyl-, formyl- and r	MF	6	1	562	4	19809	1.09E-01
GO:0072594	establishment of protein locali	BP	8	1	562	4	19809	1.09E-01
GO:0016278	lysine N-methyltransferase act	MF	8	1	562	4	19809	1.09E-01
GO:0070851	growth factor receptor binding	MF	6	1	562	4	19809	1.09E-01
GO:0004366	glycerol-3-phosphate O-acyltra	MF	8	1	562	4	19809	1.09E-01
GO:0065002	intracellular protein transmeml	BP	11	1	562	4	19809	1.09E-01
GO:0007010	cytoskeleton organization	BP	6	8	562	169	19809	1.09E-01
GO:0043410	positive regulation of MAPK c	BP	11	6	562	115	19809	1.09E-01
GO:0005242	inward rectifier potassium cha	MF	10	2	562	20	19809	1.09E-01
GO:0006509	membrane protein ectodomain	BP	9	2	562	20	19809	1.09E-01
GO:0006885	regulation of pH	BP	10	2	562	20	19809	1.09E-01
GO:0050730	regulation of peptidyl-tyrosine	BP	10	2	562	20	19809	1.09E-01
GO:0008637	apoptotic mitochondrial chang	BP	7	2	562	20	19809	1.09E-01
GO:0045638	negative regulation of myeloid	BP	8	2	562	20	19809	1.09E-01
GO:0007178	transmembrane receptor protei	BP	7	2	562	20	19809	1.09E-01
GO:0023019	signal transduction involved in	BP	5	2	562	20	19809	1.09E-01
GO:0031435	mitogen-activated protein kina	MF	8	2	562	20	19809	1.09E-01
GO:0032332	positive regulation of chondro	BP	8	2	562	20	19809	1.09E-01
GO:0016327	apicolateral plasma membrane	CC	5	2	562	20	19809	1.09E-01
GO:0099175	regulation of postsynapse orga	BP	8	2	562	20	19809	1.09E-01
GO:1990573	potassium ion import across pl	BP	11	3	562	41	19809	1.10E-01
GO:0048706	embryonic skeletal system dev	BP	7	3	562	41	19809	1.10E-01
GO:0017046	peptide hormone binding	MF	6	3	562	41	19809	1.10E-01
GO:0017048	Rho GTPase binding	MF	9	3	562	41	19809	1.10E-01
GO:0001942	hair follicle development	BP	6	3	562	41	19809	1.10E-01
GO:0042130	negative regulation of T cell pi	BP	11	3	562	41	19809	1.10E-01
GO:0015629	actin cytoskeleton	CC	7	10	562	226	19809	1.11E-01
GO:0016055	Wnt signaling pathway	BP	7	10	562	226	19809	1.11E-01
GO:0007030	Golgi organization	BP	6	6	562	116	19809	1.12E-01
GO:0051592	response to calcium ion	BP	7	4	562	65	19809	1.13E-01
GO:0031225	anchored component of memb	CC	5	7	562	143	19809	1.13E-01

GO:0019899	enzyme binding	MF	5	15	562	375	19809	1.16E-01
GO:0045184	establishment of protein locali:	BP	6	3	562	42	19809	1.16E-01
GO:0004864	protein phosphatase inhibitor a	MF	7	3	562	42	19809	1.16E-01
GO:0007605	sensory perception of sound	BP	8	7	562	144	19809	1.16E-01
GO:0050731	positive regulation of peptidyl-	BP	11	5	562	91	19809	1.17E-01
GO:0008283	cell population proliferation	BP	3	14	562	346	19809	1.17E-01
GO:0030666	endocytic vesicle membrane	CC	7	4	562	66	19809	1.18E-01
GO:0048245	eosinophil chemotaxis	BP	7	2	562	21	19809	1.18E-01
GO:0001556	oocyte maturation	BP	5	2	562	21	19809	1.18E-01
GO:0050860	negative regulation of T cell re	BP	10	2	562	21	19809	1.18E-01
GO:0043277	apoptotic cell clearance	BP	8	2	562	21	19809	1.18E-01
GO:0045821	positive regulation of glycolyti	BP	11	2	562	21	19809	1.18E-01
GO:0036120	cellular response to platelet-de	BP	6	2	562	21	19809	1.18E-01
GO:0021772	olfactory bulb development	BP	5	2	562	21	19809	1.18E-01
GO:0010033	response to organic substance	BP	5	5	562	92	19809	1.21E-01
GO:0060173	limb development	BP	6	3	562	43	19809	1.22E-01
GO:0048705	skeletal system morphogenesis	BP	6	3	562	43	19809	1.22E-01
GO:0035094	response to nicotine	BP	6	3	562	43	19809	1.22E-01
GO:0030574	collagen catabolic process	BP	5	3	562	43	19809	1.22E-01
GO:0043524	negative regulation of neuron a	BP	10	7	562	146	19809	1.22E-01
GO:0007389	pattern specification process	BP	4	4	562	67	19809	1.22E-01
GO:0046872	metal ion binding	MF	6	117	562	3735	19809	1.25E-01
GO:0065003	protein-containing complex as:	BP	6	6	562	120	19809	1.26E-01
GO:0051000	positive regulation of nitric-ox	BP	9	2	562	22	19809	1.28E-01
GO:0005922	connexin complex	CC	6	2	562	22	19809	1.28E-01
GO:0061640	cytoskeleton-dependent cytoki	BP	6	2	562	22	19809	1.28E-01
GO:0008361	regulation of cell size	BP	6	2	562	22	19809	1.28E-01
GO:0008045	motor neuron axon guidance	BP	7	2	562	22	19809	1.28E-01
GO:0010596	negative regulation of endothe	BP	10	2	562	22	19809	1.28E-01
GO:0004950	chemokine receptor activity	MF	8	2	562	22	19809	1.28E-01
GO:0016620	oxidoreductase activity, acting	MF	6	2	562	22	19809	1.28E-01
GO:0050806	positive regulation of synaptic	BP	8	2	562	22	19809	1.28E-01
GO:0030159	receptor signaling complex sca	MF	7	2	562	22	19809	1.28E-01
GO:0033627	cell adhesion mediated by inte	BP	5	2	562	22	19809	1.28E-01
GO:0042098	T cell proliferation	BP	8	2	562	22	19809	1.28E-01
GO:0071310	cellular response to organic su	BP	6	3	562	44	19809	1.29E-01
GO:0032570	response to progesterone	BP	6	3	562	44	19809	1.29E-01
GO:0019216	regulation of lipid metabolic p	BP	7	5	562	94	19809	1.29E-01
GO:0055037	recycling endosome	CC	8	6	562	121	19809	1.30E-01
GO:0007219	Notch signaling pathway	BP	6	6	562	121	19809	1.30E-01
GO:0090090	negative regulation of canonic:	BP	10	8	562	177	19809	1.32E-01
GO:0032092	positive regulation of protein b	BP	7	4	562	69	19809	1.32E-01
GO:0050796	regulation of insulin secretion	BP	10	4	562	69	19809	1.32E-01
GO:0061844	antimicrobial humoral immune	BP	7	4	562	69	19809	1.32E-01
GO:0050830	defense response to Gram-posi	BP	9	5	562	95	19809	1.33E-01
GO:0022900	electron transport chain	BP	6	5	562	95	19809	1.33E-01
GO:0003824	catalytic activity	MF	3	20	562	538	19809	1.34E-01
GO:0098794	postsynapse	CC	4	6	562	122	19809	1.34E-01
GO:0030414	peptidase inhibitor activity	MF	6	6	562	122	19809	1.34E-01
GO:0061737	leukotriene signaling pathway	BP	6	1	562	5	19809	1.34E-01

GO:0000064	L-ornithine transmembrane tra	MF	7	1	562	5	19809	1.34E-01
GO:1905007	positive regulation of epithelia	BP	10	1	562	5	19809	1.34E-01
GO:0048790	maintenance of presynaptic act	BP	7	1	562	5	19809	1.34E-01
GO:0008582	regulation of synaptic growth a	BP	7	1	562	5	19809	1.34E-01
GO:0002141	stereocilia ankle link	CC	6	1	562	5	19809	1.34E-01
GO:0030263	apoptotic chromosome conden	BP	10	1	562	5	19809	1.34E-01
GO:0006477	protein sulfation	BP	9	1	562	5	19809	1.34E-01
GO:0015277	kainate selective glutamate rec	MF	8	1	562	5	19809	1.34E-01
GO:0006710	androgen catabolic process	BP	8	1	562	5	19809	1.34E-01
GO:1900028	negative regulation of ruffle as	BP	10	1	562	5	19809	1.34E-01
GO:0004668	protein-arginine deiminase act	MF	7	1	562	5	19809	1.34E-01
GO:0006670	sphingosine metabolic process	BP	8	1	562	5	19809	1.34E-01
GO:0071504	cellular response to heparin	BP	7	1	562	5	19809	1.34E-01
GO:0071547	piP-body	CC	9	1	562	5	19809	1.34E-01
GO:0051023	regulation of immunoglobulin	BP	9	1	562	5	19809	1.34E-01
GO:0030896	checkpoint clamp complex	CC	4	1	562	5	19809	1.34E-01
GO:0060666	dichotomous subdivision of ter	BP	8	1	562	5	19809	1.34E-01
GO:0043243	positive regulation of protein c	BP	8	1	562	5	19809	1.34E-01
GO:0060011	Sertoli cell proliferation	BP	4	1	562	5	19809	1.34E-01
GO:0060171	stereocilium membrane	CC	7	1	562	5	19809	1.34E-01
GO:0002248	connective tissue replacement	BP	6	1	562	5	19809	1.34E-01
GO:0070569	uridylyltransferase activity	MF	7	1	562	5	19809	1.34E-01
GO:0060043	regulation of cardiac muscle ce	BP	7	1	562	5	19809	1.34E-01
GO:0042490	mechanoreceptor differentiatic	BP	7	1	562	5	19809	1.34E-01
GO:0042541	hemoglobin biosynthetic proce	BP	8	1	562	5	19809	1.34E-01
GO:1904036	negative regulation of epithelia	BP	10	1	562	5	19809	1.34E-01
GO:0001915	negative regulation of T cell m	BP	8	1	562	5	19809	1.34E-01
GO:0044194	cytolytic granule	CC	9	1	562	5	19809	1.34E-01
GO:1903215	negative regulation of protein t	BP	10	1	562	5	19809	1.34E-01
GO:0033227	dsRNA transport	BP	10	1	562	5	19809	1.34E-01
GO:0008296	3'-5'-exodeoxyribonuclease act	MF	9	1	562	5	19809	1.34E-01
GO:0002740	negative regulation of cytokine	BP	9	1	562	5	19809	1.34E-01
GO:0022614	membrane to membrane docki	BP	5	1	562	5	19809	1.34E-01
GO:0019318	hexose metabolic process	BP	7	1	562	5	19809	1.34E-01
GO:0002520	immune system development	BP	4	1	562	5	19809	1.34E-01
GO:0043570	maintenance of DNA repeat el	BP	8	1	562	5	19809	1.34E-01
GO:0032714	negative regulation of interleul	BP	8	1	562	5	19809	1.34E-01
GO:0005134	interleukin-2 receptor binding	MF	7	1	562	5	19809	1.34E-01
GO:0031802	type 5 metabotropic glutamate	MF	8	1	562	5	19809	1.34E-01
GO:0031228	intrinsic component of Golgi m	CC	6	1	562	5	19809	1.34E-01
GO:0008379	thioredoxin peroxidase activity	MF	5	1	562	5	19809	1.34E-01
GO:0060664	epithelial cell proliferation inv	BP	5	1	562	5	19809	1.34E-01
GO:1903401	L-lysine transmembrane transp	BP	8	1	562	5	19809	1.34E-01
GO:0036006	cellular response to macrophag	BP	8	1	562	5	19809	1.34E-01
GO:0003161	cardiac conduction system dev	BP	9	1	562	5	19809	1.34E-01
GO:1990910	response to hypobaric hypoxia	BP	6	1	562	5	19809	1.34E-01
GO:0038156	interleukin-3-mediated signalin	BP	7	1	562	5	19809	1.34E-01
GO:1904428	negative regulation of tubulin d	BP	10	1	562	5	19809	1.34E-01
GO:0044241	lipid digestion	BP	5	1	562	5	19809	1.34E-01
GO:0046813	receptor-mediated virion attacl	BP	7	1	562	5	19809	1.34E-01

GO:0016212	kynurenine-oxoglutarate transa	MF	8	1	562	5	19809	1.34E-01
GO:0048006	antigen processing and present	BP	6	1	562	5	19809	1.34E-01
GO:0030321	transepithelial chloride transpo	BP	10	1	562	5	19809	1.34E-01
GO:2001044	regulation of integrin-mediatec	BP	8	1	562	5	19809	1.34E-01
GO:0097368	establishment of Sertoli cell ba	BP	8	1	562	5	19809	1.34E-01
GO:0010656	negative regulation of muscle	BP	10	1	562	5	19809	1.34E-01
GO:0030884	exogenous lipid antigen bindin	MF	6	1	562	5	19809	1.34E-01
GO:0021650	vestibulocochlear nerve forma	BP	6	1	562	5	19809	1.34E-01
GO:0070293	renal absorption	BP	6	1	562	5	19809	1.34E-01
GO:1903307	positive regulation of regulate	BP	9	1	562	5	19809	1.34E-01
GO:1990726	Lsm1-7-Pat1 complex	CC	5	1	562	5	19809	1.34E-01
GO:1903721	positive regulation of I-kappaE	BP	11	1	562	5	19809	1.34E-01
GO:0000018	regulation of DNA recombinat	BP	9	1	562	5	19809	1.34E-01
GO:0045578	negative regulation of B cell d	BP	10	1	562	5	19809	1.34E-01
GO:0004096	catalase activity	MF	5	1	562	5	19809	1.34E-01
GO:0043111	replication fork arrest	BP	9	1	562	5	19809	1.34E-01
GO:0051546	keratinocyte migration	BP	9	1	562	5	19809	1.34E-01
GO:0045607	regulation of inner ear auditor	BP	9	1	562	5	19809	1.34E-01
GO:2001181	positive regulation of interleuk	BP	9	1	562	5	19809	1.34E-01
GO:0018812	3-hydroxyacyl-CoA dehydrata	MF	7	1	562	5	19809	1.34E-01
GO:0071224	cellular response to peptidogly	BP	8	1	562	5	19809	1.34E-01
GO:0070083	clathrin-sculpted monoamine t	CC	8	1	562	5	19809	1.34E-01
GO:0010988	regulation of low-density lipop	BP	7	1	562	5	19809	1.34E-01
GO:0051464	positive regulation of cortisol	BP	12	1	562	5	19809	1.34E-01
GO:0010961	cellular magnesium ion homeo	BP	11	1	562	5	19809	1.34E-01
GO:0045794	negative regulation of cell volu	BP	8	1	562	5	19809	1.34E-01
GO:0031622	positive regulation of fever ger	BP	11	1	562	5	19809	1.34E-01
GO:0006526	arginine biosynthetic process	BP	12	1	562	5	19809	1.34E-01
GO:1990349	gap junction-mediated intercel	BP	7	1	562	5	19809	1.34E-01
GO:0045321	leukocyte activation	BP	5	1	562	5	19809	1.34E-01
GO:0055129	L-proline biosynthetic process	BP	13	1	562	5	19809	1.34E-01
GO:0032983	kainate selective glutamate rec	CC	8	1	562	5	19809	1.34E-01
GO:0060050	positive regulation of protein g	BP	10	1	562	5	19809	1.34E-01
GO:0035696	monocyte extravasation	BP	6	1	562	5	19809	1.34E-01
GO:0019960	C-X3-C chemokine binding	MF	7	1	562	5	19809	1.34E-01
GO:0070189	kynurenine metabolic process	BP	6	1	562	5	19809	1.34E-01
GO:0019815	B cell receptor complex	CC	5	1	562	5	19809	1.34E-01
GO:0003357	noradrenergic neuron different	BP	7	1	562	5	19809	1.34E-01
GO:0102121	ceramidase activity	MF	7	1	562	5	19809	1.34E-01
GO:0010463	mesenchymal cell proliferatior	BP	4	1	562	5	19809	1.34E-01
GO:0097350	neutrophil clearance	BP	5	1	562	5	19809	1.34E-01
GO:0051900	regulation of mitochondrial de	BP	7	1	562	5	19809	1.34E-01
GO:0003289	atrial septum primum morphog	BP	7	1	562	5	19809	1.34E-01
GO:0031547	brain-derived neurotrophic fac	BP	8	1	562	5	19809	1.34E-01
GO:0097070	ductus arteriosus closure	BP	8	1	562	5	19809	1.34E-01
GO:0001851	complement component C3b b	MF	6	1	562	5	19809	1.34E-01
GO:0045716	positive regulation of low-dens	BP	10	1	562	5	19809	1.34E-01
GO:0018101	protein citrullination	BP	11	1	562	5	19809	1.34E-01
GO:0032611	interleukin-1 beta production	BP	6	1	562	5	19809	1.34E-01
GO:0043615	astrocyte cell migration	BP	8	1	562	5	19809	1.34E-01

GO:0071799	cellular response to prostaglan	BP	8	1	562	5	19809	1.34E-01
GO:1904141	positive regulation of microgli	BP	12	1	562	5	19809	1.34E-01
GO:0043533	inositol 1,3,4,5 tetrakisphospha	MF	6	1	562	5	19809	1.34E-01
GO:0014905	myoblast fusion involved in sk	BP	7	1	562	5	19809	1.34E-01
GO:0021544	subpallium development	BP	5	1	562	5	19809	1.34E-01
GO:0031727	CCR2 chemokine receptor bin	MF	9	1	562	5	19809	1.34E-01
GO:0048406	nerve growth factor binding	MF	7	1	562	5	19809	1.34E-01
GO:0070345	negative regulation of fat cell p	BP	8	1	562	5	19809	1.34E-01
GO:0006547	histidine metabolic process	BP	10	1	562	5	19809	1.34E-01
GO:0017038	protein import	BP	10	1	562	5	19809	1.34E-01
GO:0046934	phosphatidylinositol-4,5-bisph	MF	8	1	562	5	19809	1.34E-01
GO:0060406	positive regulation of penile er	BP	7	1	562	5	19809	1.34E-01
GO:0006824	cobalt ion transport	BP	10	1	562	5	19809	1.34E-01
GO:0098639	collagen binding involved in c	MF	6	1	562	5	19809	1.34E-01
GO:0048570	notochord morphogenesis	BP	7	1	562	5	19809	1.34E-01
GO:2001301	lipoxin biosynthetic process	BP	12	1	562	5	19809	1.34E-01
GO:0019863	IgE binding	MF	6	1	562	5	19809	1.34E-01
GO:0030091	protein repair	BP	8	1	562	5	19809	1.34E-01
GO:2000556	positive regulation of T-helper	BP	12	1	562	5	19809	1.34E-01
GO:0030156	benzodiazepine receptor bindi	MF	6	1	562	5	19809	1.34E-01
GO:0014042	positive regulation of neuron n	BP	11	1	562	5	19809	1.34E-01
GO:0005534	galactose binding	MF	6	1	562	5	19809	1.34E-01
GO:0036414	histone citrullination	BP	10	1	562	5	19809	1.34E-01
GO:0051222	positive regulation of protein t	BP	7	1	562	5	19809	1.34E-01
GO:0019441	tryptophan catabolic process to	BP	12	1	562	5	19809	1.34E-01
GO:1901203	positive regulation of extracell	BP	7	1	562	5	19809	1.34E-01
GO:0038163	thrombopoietin-mediated signa	BP	8	1	562	5	19809	1.34E-01
GO:0034711	inhibin binding	MF	5	1	562	5	19809	1.34E-01
GO:0042270	protection from natural killer c	BP	9	1	562	5	19809	1.34E-01
GO:0070290	N-acylphosphatidylethanolami	MF	8	1	562	5	19809	1.34E-01
GO:0030858	positive regulation of epithelia	BP	8	1	562	5	19809	1.34E-01
GO:0001740	Barr body	CC	8	1	562	5	19809	1.34E-01
GO:0007356	thorax and anterior abdomen d	BP	7	1	562	5	19809	1.34E-01
GO:0032143	single thymine insertion bindi	MF	11	1	562	5	19809	1.34E-01
GO:0002666	positive regulation of T cell to	BP	8	1	562	5	19809	1.34E-01
GO:0050951	sensory perception of tempera	BP	7	1	562	5	19809	1.34E-01
GO:0032534	regulation of microvillus asser	BP	10	1	562	5	19809	1.34E-01
GO:0006101	citrate metabolic process	BP	7	1	562	5	19809	1.34E-01
GO:0031013	troponin I binding	MF	6	1	562	5	19809	1.34E-01
GO:2000660	negative regulation of interleu	BP	10	1	562	5	19809	1.34E-01
GO:1903347	negative regulation of bicellula	BP	9	1	562	5	19809	1.34E-01
GO:0006220	pyrimidine nucleotide metabol	BP	9	1	562	5	19809	1.34E-01
GO:0001405	PAM complex, Tim23 associa	CC	8	1	562	5	19809	1.34E-01
GO:0030035	microspike assembly	BP	8	1	562	5	19809	1.34E-01
GO:2000343	positive regulation of chemoki	BP	9	1	562	5	19809	1.34E-01
GO:0070231	T cell apoptotic process	BP	9	1	562	5	19809	1.34E-01
GO:0030883	endogenous lipid antigen bindi	MF	6	1	562	5	19809	1.34E-01
GO:0016202	regulation of striated muscle ti	BP	8	1	562	5	19809	1.34E-01
GO:0060485	mesenchyme development	BP	6	1	562	5	19809	1.34E-01
GO:0006290	pyrimidine dimer repair	BP	9	1	562	5	19809	1.34E-01

GO:0030881	beta-2-microglobulin binding	MF	5	1	562	5	19809	1.34E-01
GO:2000405	negative regulation of T cell m	BP	9	1	562	5	19809	1.34E-01
GO:0035509	negative regulation of myosin-	BP	13	1	562	5	19809	1.34E-01
GO:0030618	transforming growth factor bet	MF	5	1	562	5	19809	1.34E-01
GO:0015936	coenzyme A metabolic process	BP	7	1	562	5	19809	1.34E-01
GO:0010715	regulation of extracellular mat	BP	8	1	562	5	19809	1.34E-01
GO:0015809	arginine transport	BP	8	1	562	5	19809	1.34E-01
GO:1990511	piRNA biosynthetic process	BP	11	1	562	5	19809	1.34E-01
GO:0004957	prostaglandin E receptor activi	MF	10	1	562	5	19809	1.34E-01
GO:0009408	response to heat	BP	5	3	562	45	19809	1.35E-01
GO:0008144	drug binding	MF	4	4	562	70	19809	1.37E-01
GO:0007224	smoothened signaling pathway	BP	6	4	562	70	19809	1.37E-01
GO:0044305	calyx of Held	CC	7	2	562	23	19809	1.38E-01
GO:0016493	C-C chemokine receptor activi	MF	9	2	562	23	19809	1.38E-01
GO:0008483	transaminase activity	MF	6	2	562	23	19809	1.38E-01
GO:0048872	homeostasis of number of cells	BP	6	2	562	23	19809	1.38E-01
GO:0006730	one-carbon metabolic process	BP	5	2	562	23	19809	1.38E-01
GO:0001965	G-protein alpha-subunit bindin	MF	5	2	562	23	19809	1.38E-01
GO:0001892	embryonic placenta developme	BP	5	2	562	23	19809	1.38E-01
GO:0042759	long-chain fatty acid biosynthe	BP	11	2	562	23	19809	1.38E-01
GO:0001671	ATPase activator activity	MF	6	2	562	23	19809	1.38E-01
GO:0006342	chromatin silencing	BP	5	2	562	23	19809	1.38E-01
GO:0030742	GTP-dependent protein bindin	MF	5	2	562	23	19809	1.38E-01
GO:0043280	positive regulation of cysteine-	BP	10	3	562	46	19809	1.42E-01
GO:0050714	positive regulation of protein s	BP	10	3	562	46	19809	1.42E-01
GO:0060048	cardiac muscle contraction	BP	8	3	562	46	19809	1.42E-01
GO:0045786	negative regulation of cell cycl	BP	7	3	562	46	19809	1.42E-01
GO:0007565	female pregnancy	BP	5	5	562	97	19809	1.42E-01
GO:0120162	positive regulation of cold-ind	BP	7	5	562	97	19809	1.42E-01
GO:0007165	signal transduction	BP	4	61	562	1874	19809	1.42E-01
GO:0031490	chromatin DNA binding	MF	7	4	562	71	19809	1.43E-01
GO:0003924	GTPase activity	MF	9	13	562	328	19809	1.43E-01
GO:0005788	endoplasmic reticulum lumen	CC	7	12	562	298	19809	1.43E-01
GO:0010629	negative regulation of gene ex	BP	8	11	562	269	19809	1.45E-01
GO:0007204	positive regulation of cytosolic	BP	13	7	562	153	19809	1.45E-01
GO:1990830	cellular response to leukemia i	BP	8	5	562	98	19809	1.46E-01
GO:0008134	transcription factor binding	MF	5	14	562	360	19809	1.46E-01
GO:0034341	response to interferon-gamma	BP	7	2	562	24	19809	1.47E-01
GO:0008378	galactosyltransferase activity	MF	7	2	562	24	19809	1.47E-01
GO:0050870	positive regulation of T cell ac	BP	9	2	562	24	19809	1.47E-01
GO:0032418	lysosome localization	BP	6	2	562	24	19809	1.47E-01
GO:0008023	transcription elongation factor	CC	4	2	562	24	19809	1.47E-01
GO:0006884	cell volume homeostasis	BP	7	2	562	24	19809	1.47E-01
GO:0000137	Golgi cis cisterna	CC	8	2	562	24	19809	1.47E-01
GO:0001891	phagocytic cup	CC	5	2	562	24	19809	1.47E-01
GO:0019957	C-C chemokine binding	MF	7	2	562	24	19809	1.47E-01
GO:0007141	male meiosis I	BP	8	2	562	24	19809	1.47E-01
GO:0071556	integral component of lumenal	CC	8	2	562	24	19809	1.47E-01
GO:0045921	positive regulation of exocytos	BP	8	2	562	24	19809	1.47E-01
GO:0021766	hippocampus development	BP	5	4	562	72	19809	1.48E-01

GO:0030218	erythrocyte differentiation	BP	7	3	562	47	19809	1.48E-01
GO:0043536	positive regulation of blood ve	BP	11	3	562	47	19809	1.48E-01
GO:0007154	cell communication	BP	4	3	562	47	19809	1.48E-01
GO:0032420	stereocilium	CC	6	3	562	47	19809	1.48E-01
GO:0048538	thymus development	BP	7	3	562	47	19809	1.48E-01
GO:0008138	protein tyrosine/serine/threonin	MF	9	3	562	47	19809	1.48E-01
GO:0021915	neural tube development	BP	6	3	562	47	19809	1.48E-01
GO:0090305	nucleic acid phosphodiester bc	BP	8	6	562	126	19809	1.49E-01
GO:0005856	cytoskeleton	CC	6	44	562	1320	19809	1.50E-01
GO:0001501	skeletal system development	BP	6	7	562	155	19809	1.52E-01
GO:0001938	positive regulation of endothel	BP	9	4	562	73	19809	1.53E-01
GO:0010862	positive regulation of pathway	BP	11	3	562	48	19809	1.55E-01
GO:0006635	fatty acid beta-oxidation	BP	11	3	562	48	19809	1.55E-01
GO:0043666	regulation of phosphoprotein p	BP	11	3	562	48	19809	1.55E-01
GO:0030182	neuron differentiation	BP	6	7	562	156	19809	1.56E-01
GO:0001158	enhancer sequence-specific DN	MF	9	2	562	25	19809	1.57E-01
GO:0035307	positive regulation of protein c	BP	10	2	562	25	19809	1.57E-01
GO:2000353	positive regulation of endothel	BP	11	2	562	25	19809	1.57E-01
GO:0030099	myeloid cell differentiation	BP	6	2	562	25	19809	1.57E-01
GO:2001243	negative regulation of intrinsic	BP	10	2	562	25	19809	1.57E-01
GO:0019956	chemokine binding	MF	6	2	562	25	19809	1.57E-01
GO:0002053	positive regulation of mesench	BP	8	2	562	25	19809	1.57E-01
GO:0060135	maternal process involved in fe	BP	5	2	562	25	19809	1.57E-01
GO:0008376	acetylgalactosaminyltransferas	MF	7	2	562	25	19809	1.57E-01
GO:0043425	bHLH transcription factor binc	MF	7	2	562	25	19809	1.57E-01
GO:0045332	phospholipid translocation	BP	9	2	562	25	19809	1.57E-01
GO:0005504	fatty acid binding	MF	5	2	562	25	19809	1.57E-01
GO:0008375	acetylglucosaminyltransferase	MF	7	2	562	25	19809	1.57E-01
GO:0032868	response to insulin	BP	7	4	562	74	19809	1.58E-01
GO:0002322	B cell proliferation involved ir	BP	9	1	562	6	19809	1.59E-01
GO:0042423	catecholamine biosynthetic pro	BP	9	1	562	6	19809	1.59E-01
GO:0090238	positive regulation of arachido	BP	10	1	562	6	19809	1.59E-01
GO:0004771	sterol esterase activity	MF	7	1	562	6	19809	1.59E-01
GO:0072540	T-helper 17 cell lineage comm	BP	10	1	562	6	19809	1.59E-01
GO:0046931	pore complex assembly	BP	7	1	562	6	19809	1.59E-01
GO:0044245	polysaccharide digestion	BP	5	1	562	6	19809	1.59E-01
GO:0004551	nucleotide diphosphatase activ	MF	8	1	562	6	19809	1.59E-01
GO:0036041	long-chain fatty acid binding	MF	6	1	562	6	19809	1.59E-01
GO:0046881	positive regulation of follicle-s	BP	10	1	562	6	19809	1.59E-01
GO:1900122	positive regulation of receptor	BP	8	1	562	6	19809	1.59E-01
GO:0033278	cell proliferation in midbrain	BP	5	1	562	6	19809	1.59E-01
GO:1901018	positive regulation of potassiu	BP	8	1	562	6	19809	1.59E-01
GO:0032137	guanine/thymine mispair bindi	MF	9	1	562	6	19809	1.59E-01
GO:0047086	ketosteroid monooxygenase ac	MF	7	1	562	6	19809	1.59E-01
GO:0042976	activation of Janus kinase acti	BP	11	1	562	6	19809	1.59E-01
GO:0005587	collagen type IV trimer	CC	6	1	562	6	19809	1.59E-01
GO:0008310	single-stranded DNA 3'-5' exo	MF	10	1	562	6	19809	1.59E-01
GO:0042471	ear morphogenesis	BP	7	1	562	6	19809	1.59E-01
GO:0017040	N-acylsphingosine amidohydro	MF	7	1	562	6	19809	1.59E-01
GO:1903140	regulation of establishment of	BP	10	1	562	6	19809	1.59E-01

GO:0070309	lens fiber cell morphogenesis	BP	8	1	562	6	19809	1.59E-01
GO:0006531	aspartate metabolic process	BP	11	1	562	6	19809	1.59E-01
GO:1903862	positive regulation of oxidativ	BP	11	1	562	6	19809	1.59E-01
GO:1901727	positive regulation of histone c	BP	12	1	562	6	19809	1.59E-01
GO:0060179	male mating behavior	BP	6	1	562	6	19809	1.59E-01
GO:0014031	mesenchymal cell developmen	BP	6	1	562	6	19809	1.59E-01
GO:0047631	ADP-ribose diphosphatase acti	MF	8	1	562	6	19809	1.59E-01
GO:0010754	negative regulation of cGMP-r	BP	10	1	562	6	19809	1.59E-01
GO:0046533	negative regulation of photore	BP	11	1	562	6	19809	1.59E-01
GO:0050672	negative regulation of lympho	BP	10	1	562	6	19809	1.59E-01
GO:0042487	regulation of odontogenesis of	BP	9	1	562	6	19809	1.59E-01
GO:0072719	cellular response to cisplatin	BP	7	1	562	6	19809	1.59E-01
GO:0060478	acrosomal vesicle exocytosis	BP	10	1	562	6	19809	1.59E-01
GO:0060341	regulation of cellular localizati	BP	6	1	562	6	19809	1.59E-01
GO:0051702	interaction with symbiont	BP	5	1	562	6	19809	1.59E-01
GO:0097485	neuron projection guidance	BP	5	1	562	6	19809	1.59E-01
GO:0050917	sensory perception of umami t	BP	9	1	562	6	19809	1.59E-01
GO:0002142	stereocilia ankle link complex	CC	4	1	562	6	19809	1.59E-01
GO:0002138	retinoic acid biosynthetic proc	BP	11	1	562	6	19809	1.59E-01
GO:0006269	DNA replication, synthesis of	BP	8	1	562	6	19809	1.59E-01
GO:0032488	Cdc42 protein signal transduct	BP	9	1	562	6	19809	1.59E-01
GO:0007144	female meiosis I	BP	8	1	562	6	19809	1.59E-01
GO:0042608	T cell receptor binding	MF	6	1	562	6	19809	1.59E-01
GO:0030578	PML body organization	BP	8	1	562	6	19809	1.59E-01
GO:1900454	positive regulation of long-terr	BP	6	1	562	6	19809	1.59E-01
GO:0010040	response to iron(II) ion	BP	8	1	562	6	19809	1.59E-01
GO:0002830	positive regulation of type 2 in	BP	8	1	562	6	19809	1.59E-01
GO:0048261	negative regulation of receptor	BP	9	1	562	6	19809	1.59E-01
GO:2001141	regulation of RNA biosyntheti	BP	8	1	562	6	19809	1.59E-01
GO:0010807	regulation of synaptic vesicle p	BP	7	1	562	6	19809	1.59E-01
GO:0008131	primary amine oxidase activity	MF	7	1	562	6	19809	1.59E-01
GO:2000404	regulation of T cell migration	BP	8	1	562	6	19809	1.59E-01
GO:0003015	heart process	BP	6	1	562	6	19809	1.59E-01
GO:0007007	inner mitochondrial membrane	BP	8	1	562	6	19809	1.59E-01
GO:2000647	negative regulation of stem cel	BP	8	1	562	6	19809	1.59E-01
GO:0060022	hard palate development	BP	5	1	562	6	19809	1.59E-01
GO:0004528	phosphodiesterase I activity	MF	8	1	562	6	19809	1.59E-01
GO:0010310	regulation of hydrogen peroxic	BP	8	1	562	6	19809	1.59E-01
GO:1901164	negative regulation of trophob	BP	9	1	562	6	19809	1.59E-01
GO:0055131	C3HC4-type RING finger dom	MF	6	1	562	6	19809	1.59E-01
GO:0010659	cardiac muscle cell apoptotic p	BP	9	1	562	6	19809	1.59E-01
GO:0050916	sensory perception of sweet ta	BP	9	1	562	6	19809	1.59E-01
GO:0015288	porin activity	MF	8	1	562	6	19809	1.59E-01
GO:0050913	sensory perception of bitter tas	BP	9	1	562	6	19809	1.59E-01
GO:0018027	peptidyl-lysine dimethylation	BP	12	1	562	6	19809	1.59E-01
GO:0009143	nucleoside triphosphate catabo	BP	9	1	562	6	19809	1.59E-01
GO:0098712	L-glutamate import across plas	BP	7	1	562	6	19809	1.59E-01
GO:1904929	coreceptor activity involved in	MF	7	1	562	6	19809	1.59E-01
GO:0006552	leucine catabolic process	BP	11	1	562	6	19809	1.59E-01
GO:0055102	lipase inhibitor activity	MF	6	1	562	6	19809	1.59E-01

GO:0060706	cell differentiation involved in	BP	5	1	562	6	19809	1.59E-01
GO:0043305	negative regulation of mast cel	BP	10	1	562	6	19809	1.59E-01
GO:0019464	glycine decarboxylation via gl	BP	13	1	562	6	19809	1.59E-01
GO:1903038	negative regulation of leukocy	BP	8	1	562	6	19809	1.59E-01
GO:0060228	phosphatidylcholine-sterol O-a	MF	6	1	562	6	19809	1.59E-01
GO:0032020	ISG15-protein conjugation	BP	9	1	562	6	19809	1.59E-01
GO:0000320	re-entry into mitotic cell cycle	BP	5	1	562	6	19809	1.59E-01
GO:0061304	retinal blood vessel morphoge	BP	7	1	562	6	19809	1.59E-01
GO:0042743	hydrogen peroxide metabolic p	BP	7	1	562	6	19809	1.59E-01
GO:0097136	Bcl-2 family protein complex	CC	4	1	562	6	19809	1.59E-01
GO:0034354	'de novo' NAD biosynthetic pr	BP	11	1	562	6	19809	1.59E-01
GO:0060628	regulation of ER to Golgi vesi	BP	8	1	562	6	19809	1.59E-01
GO:0002318	myeloid progenitor cell differe	BP	7	1	562	6	19809	1.59E-01
GO:0034698	response to gonadotropin	BP	6	1	562	6	19809	1.59E-01
GO:0072675	osteoclast fusion	BP	6	1	562	6	19809	1.59E-01
GO:0061299	retina vasculature morphogene	BP	5	1	562	6	19809	1.59E-01
GO:0070257	positive regulation of mucus se	BP	9	1	562	6	19809	1.59E-01
GO:0097527	necroptotic signaling pathway	BP	5	1	562	6	19809	1.59E-01
GO:0010694	positive regulation of alkaline	BP	12	1	562	6	19809	1.59E-01
GO:2000427	positive regulation of apoptoti	BP	9	1	562	6	19809	1.59E-01
GO:0034447	very-low-density lipoprotein p	BP	5	1	562	6	19809	1.59E-01
GO:0032914	positive regulation of transforr	BP	9	1	562	6	19809	1.59E-01
GO:0055009	atrial cardiac muscle tissue mo	BP	8	1	562	6	19809	1.59E-01
GO:0030368	interleukin-17 receptor activity	MF	7	1	562	6	19809	1.59E-01
GO:0072559	NLRP3 inflammasome comple	CC	5	1	562	6	19809	1.59E-01
GO:0032405	MutLalpha complex binding	MF	6	1	562	6	19809	1.59E-01
GO:0002686	negative regulation of leukocy	BP	7	1	562	6	19809	1.59E-01
GO:0031223	auditory behavior	BP	5	1	562	6	19809	1.59E-01
GO:0008238	exopeptidase activity	MF	7	1	562	6	19809	1.59E-01
GO:0032782	bile acid secretion	BP	11	1	562	6	19809	1.59E-01
GO:0061370	testosterone biosynthetic proce	BP	8	1	562	6	19809	1.59E-01
GO:0033499	galactose catabolic process via	BP	10	1	562	6	19809	1.59E-01
GO:0032813	tumor necrosis factor receptor	MF	7	1	562	6	19809	1.59E-01
GO:0001765	membrane raft assembly	BP	7	1	562	6	19809	1.59E-01
GO:0070245	positive regulation of thymocy	BP	13	1	562	6	19809	1.59E-01
GO:0097119	postsynaptic density protein 9	BP	8	1	562	6	19809	1.59E-01
GO:0089701	U2AF	CC	4	1	562	6	19809	1.59E-01
GO:0043237	laminin-1 binding	MF	6	1	562	6	19809	1.59E-01
GO:0048729	tissue morphogenesis	BP	5	1	562	6	19809	1.59E-01
GO:0004111	creatine kinase activity	MF	7	1	562	6	19809	1.59E-01
GO:2001268	negative regulation of cysteine	BP	11	1	562	6	19809	1.59E-01
GO:1903978	regulation of microglial cell ac	BP	8	1	562	6	19809	1.59E-01
GO:0042063	gliogenesis	BP	7	1	562	6	19809	1.59E-01
GO:0015866	ADP transport	BP	9	1	562	6	19809	1.59E-01
GO:0009812	flavonoid metabolic process	BP	5	1	562	6	19809	1.59E-01
GO:1900020	positive regulation of protein k	BP	13	1	562	6	19809	1.59E-01
GO:0097443	sorting endosome	CC	8	1	562	6	19809	1.59E-01
GO:0001739	sex chromatin	CC	9	1	562	6	19809	1.59E-01
GO:0072384	organelle transport along micr	BP	9	1	562	6	19809	1.59E-01
GO:2000503	positive regulation of natural k	BP	10	1	562	6	19809	1.59E-01

GO:0030644	cellular chloride ion homeosta	BP	11	1	562	6	19809	1.59E-01
GO:0003272	endocardial cushion formation	BP	5	1	562	6	19809	1.59E-01
GO:0015677	copper ion import	BP	11	1	562	6	19809	1.59E-01
GO:0035335	peptidyl-tyrosine dephosphory	BP	10	5	562	101	19809	1.60E-01
GO:0051087	chaperone binding	MF	5	5	562	101	19809	1.60E-01
GO:0005085	guanyl-nucleotide exchange fa	MF	7	8	562	186	19809	1.60E-01
GO:0042110	T cell activation	BP	7	3	562	49	19809	1.62E-01
GO:0051209	release of sequestered calcium	BP	8	3	562	49	19809	1.62E-01
GO:0001618	virus receptor activity	MF	5	4	562	75	19809	1.64E-01
GO:0055038	recycling endosome membrane	CC	9	4	562	75	19809	1.64E-01
GO:0005509	calcium ion binding	MF	7	25	562	714	19809	1.64E-01
GO:0050768	negative regulation of neuroge	BP	9	2	562	26	19809	1.68E-01
GO:0048741	skeletal muscle fiber developm	BP	9	2	562	26	19809	1.68E-01
GO:0007026	negative regulation of microtul	BP	10	2	562	26	19809	1.68E-01
GO:0014003	oligodendrocyte development	BP	7	2	562	26	19809	1.68E-01
GO:0031032	actomyosin structure organizat	BP	8	2	562	26	19809	1.68E-01
GO:0031334	positive regulation of protein c	BP	7	2	562	26	19809	1.68E-01
GO:0048675	axon extension	BP	7	2	562	26	19809	1.68E-01
GO:0070888	E-box binding	MF	11	3	562	50	19809	1.69E-01
GO:0019902	phosphatase binding	MF	6	3	562	50	19809	1.69E-01
GO:0032720	negative regulation of tumor n	BP	9	3	562	50	19809	1.69E-01
GO:0031098	stress-activated protein kinase	BP	6	3	562	50	19809	1.69E-01
GO:0002931	response to ischemia	BP	5	3	562	50	19809	1.69E-01
GO:0006351	transcription, DNA-templated	BP	8	6	562	131	19809	1.69E-01
GO:0030512	negative regulation of transfor	BP	10	4	562	76	19809	1.69E-01
GO:0017148	negative regulation of translati	BP	10	4	562	77	19809	1.75E-01
GO:0009791	post-embryonic development	BP	4	4	562	77	19809	1.75E-01
GO:0016032	viral process	BP	6	17	562	466	19809	1.75E-01
GO:0042802	identical protein binding	MF	5	36	562	1078	19809	1.76E-01
GO:0042626	ATPase activity, coupled to tra	MF	8	3	562	51	19809	1.76E-01
GO:0007162	negative regulation of cell adh	BP	6	3	562	51	19809	1.76E-01
GO:0048167	regulation of synaptic plasticit	BP	9	3	562	51	19809	1.76E-01
GO:0032515	negative regulation of phospho	BP	12	3	562	51	19809	1.76E-01
GO:0014069	postsynaptic density	CC	5	11	562	281	19809	1.77E-01
GO:0004540	ribonuclease activity	MF	7	2	562	27	19809	1.78E-01
GO:0048870	cell motility	BP	5	2	562	27	19809	1.78E-01
GO:0005640	nuclear outer membrane	CC	6	2	562	27	19809	1.78E-01
GO:0060976	coronary vasculature developm	BP	6	2	562	27	19809	1.78E-01
GO:0042605	peptide antigen binding	MF	5	2	562	27	19809	1.78E-01
GO:0003746	translation elongation factor ac	MF	8	2	562	27	19809	1.78E-01
GO:0050690	regulation of defense response	BP	7	2	562	27	19809	1.78E-01
GO:0005184	neuropeptide hormone activity	MF	8	2	562	27	19809	1.78E-01
GO:0001954	positive regulation of cell-matı	BP	8	2	562	27	19809	1.78E-01
GO:0043687	post-translational protein modi	BP	9	13	562	343	19809	1.79E-01
GO:0051015	actin filament binding	MF	7	8	562	192	19809	1.80E-01
GO:0030183	B cell differentiation	BP	8	4	562	78	19809	1.81E-01
GO:0030628	pre-mRNA 3'-splice site bindir	MF	8	1	562	7	19809	1.82E-01
GO:0015174	basic amino acid transmembra	MF	8	1	562	7	19809	1.82E-01
GO:0070585	protein localization to mitocho	BP	8	1	562	7	19809	1.82E-01
GO:0045835	negative regulation of meiotic	BP	9	1	562	7	19809	1.82E-01

GO:0042612	MHC class I protein complex	CC	7	1	562	7	19809	1.82E-01
GO:1903243	negative regulation of cardiac :	BP	11	1	562	7	19809	1.82E-01
GO:0036089	cleavage furrow formation	BP	6	1	562	7	19809	1.82E-01
GO:1900744	regulation of p38MAPK casca	BP	12	1	562	7	19809	1.82E-01
GO:0045630	positive regulation of T-helper	BP	9	1	562	7	19809	1.82E-01
GO:0021902	commitment of neuronal cell t	BP	8	1	562	7	19809	1.82E-01
GO:0150094	amyloid-beta clearance by cell	BP	6	1	562	7	19809	1.82E-01
GO:0045428	regulation of nitric oxide biosy	BP	6	1	562	7	19809	1.82E-01
GO:0097151	positive regulation of inhibitor	BP	9	1	562	7	19809	1.82E-01
GO:0070973	protein localization to endopla	BP	9	1	562	7	19809	1.82E-01
GO:0001614	purinergic nucleotide receptor	MF	7	1	562	7	19809	1.82E-01
GO:0021861	forebrain radial glial cell differ	BP	8	1	562	7	19809	1.82E-01
GO:0051099	positive regulation of binding	BP	6	1	562	7	19809	1.82E-01
GO:0031730	CCR5 chemokine receptor bin	MF	9	1	562	7	19809	1.82E-01
GO:0050920	regulation of chemotaxis	BP	7	1	562	7	19809	1.82E-01
GO:0060536	cartilage morphogenesis	BP	6	1	562	7	19809	1.82E-01
GO:0071546	pi-body	CC	9	1	562	7	19809	1.82E-01
GO:0043496	regulation of protein homodim	BP	7	1	562	7	19809	1.82E-01
GO:2000318	positive regulation of T-helper	BP	10	1	562	7	19809	1.82E-01
GO:0048007	antigen processing and present	BP	6	1	562	7	19809	1.82E-01
GO:0097647	amylin receptor signaling path	BP	7	1	562	7	19809	1.82E-01
GO:1904798	positive regulation of core pro	BP	9	1	562	7	19809	1.82E-01
GO:0045348	positive regulation of MHC cl	BP	9	1	562	7	19809	1.82E-01
GO:0102991	myristoyl-CoA hydrolase activ	MF	7	1	562	7	19809	1.82E-01
GO:0045919	positive regulation of cytolysis	BP	7	1	562	7	19809	1.82E-01
GO:0032374	regulation of cholesterol transp	BP	9	1	562	7	19809	1.82E-01
GO:0006475	internal protein amino acid ace	BP	11	1	562	7	19809	1.82E-01
GO:0006105	succinate metabolic process	BP	6	1	562	7	19809	1.82E-01
GO:0071286	cellular response to magnesiur	BP	8	1	562	7	19809	1.82E-01
GO:0004711	ribosomal protein S6 kinase ac	MF	9	1	562	7	19809	1.82E-01
GO:0006308	DNA catabolic process	BP	8	1	562	7	19809	1.82E-01
GO:0043270	positive regulation of ion trans	BP	8	1	562	7	19809	1.82E-01
GO:0017121	plasma membrane phospholipi	BP	7	1	562	7	19809	1.82E-01
GO:0002674	negative regulation of acute in	BP	10	1	562	7	19809	1.82E-01
GO:0030497	fatty acid elongation	BP	11	1	562	7	19809	1.82E-01
GO:0006650	glycerophospholipid metabolic	BP	8	1	562	7	19809	1.82E-01
GO:2001269	positive regulation of cysteine-	BP	11	1	562	7	19809	1.82E-01
GO:0015672	monovalent inorganic cation tr	BP	8	1	562	7	19809	1.82E-01
GO:0032357	oxidized purine DNA binding	MF	9	1	562	7	19809	1.82E-01
GO:0048842	positive regulation of axon ext	BP	9	1	562	7	19809	1.82E-01
GO:0030641	regulation of cellular pH	BP	11	1	562	7	19809	1.82E-01
GO:0060117	auditory receptor cell develop	BP	8	1	562	7	19809	1.82E-01
GO:0032308	positive regulation of prostagl	BP	10	1	562	7	19809	1.82E-01
GO:1904684	negative regulation of metallo	BP	12	1	562	7	19809	1.82E-01
GO:1904781	positive regulation of protein l	BP	7	1	562	7	19809	1.82E-01
GO:0044351	macropinocytosis	BP	9	1	562	7	19809	1.82E-01
GO:0050291	sphingosine N-acyltransferase	MF	8	1	562	7	19809	1.82E-01
GO:1903288	positive regulation of potassiu	BP	12	1	562	7	19809	1.82E-01
GO:0004536	deoxyribonuclease activity	MF	7	1	562	7	19809	1.82E-01
GO:0042428	serotonin metabolic process	BP	7	1	562	7	19809	1.82E-01

GO:0048341	paraxial mesoderm formation	BP	7	1	562	7	19809	1.82E-01
GO:0072015	glomerular visceral epithelial c	BP	8	1	562	7	19809	1.82E-01
GO:0040036	regulation of fibroblast growth	BP	8	1	562	7	19809	1.82E-01
GO:0006771	riboflavin metabolic process	BP	7	1	562	7	19809	1.82E-01
GO:0032808	lacrimal gland development	BP	7	1	562	7	19809	1.82E-01
GO:0000904	cell morphogenesis involved in	BP	7	1	562	7	19809	1.82E-01
GO:0009437	carnitine metabolic process	BP	7	1	562	7	19809	1.82E-01
GO:0086008	voltage-gated potassium chann	MF	10	1	562	7	19809	1.82E-01
GO:1903997	positive regulation of non-men	BP	13	1	562	7	19809	1.82E-01
GO:0045654	positive regulation of megakar	BP	9	1	562	7	19809	1.82E-01
GO:0050996	positive regulation of lipid cat	BP	8	1	562	7	19809	1.82E-01
GO:0048732	gland development	BP	6	1	562	7	19809	1.82E-01
GO:0016264	gap junction assembly	BP	8	1	562	7	19809	1.82E-01
GO:0001553	luteinization	BP	5	1	562	7	19809	1.82E-01
GO:0010766	negative regulation of sodium	BP	10	1	562	7	19809	1.82E-01
GO:0048102	autophagic cell death	BP	6	1	562	7	19809	1.82E-01
GO:1902667	regulation of axon guidance	BP	10	1	562	7	19809	1.82E-01
GO:0030913	paranodal junction assembly	BP	8	1	562	7	19809	1.82E-01
GO:0010827	regulation of glucose transmen	BP	8	1	562	7	19809	1.82E-01
GO:0015939	pantothenate metabolic proces	BP	6	1	562	7	19809	1.82E-01
GO:0047429	nucleoside-triphosphate dipho	MF	8	1	562	7	19809	1.82E-01
GO:0045217	cell-cell junction maintenance	BP	7	1	562	7	19809	1.82E-01
GO:0001505	regulation of neurotransmitter	BP	5	1	562	7	19809	1.82E-01
GO:0005004	GPI-linked ephrin receptor act	MF	11	1	562	7	19809	1.82E-01
GO:0001517	N-acetylglucosamine 6-O-sulf	MF	7	1	562	7	19809	1.82E-01
GO:0032489	regulation of Cdc42 protein sig	BP	12	1	562	7	19809	1.82E-01
GO:0060290	transdifferentiation	BP	6	1	562	7	19809	1.82E-01
GO:0001848	complement binding	MF	5	1	562	7	19809	1.82E-01
GO:0045545	syndecan binding	MF	6	1	562	7	19809	1.82E-01
GO:0043654	recognition of apoptotic cell	BP	6	1	562	7	19809	1.82E-01
GO:0046514	ceramide catabolic process	BP	10	1	562	7	19809	1.82E-01
GO:0042167	heme catabolic process	BP	9	1	562	7	19809	1.82E-01
GO:2000553	positive regulation of T-helper	BP	12	1	562	7	19809	1.82E-01
GO:0030011	maintenance of cell polarity	BP	5	1	562	7	19809	1.82E-01
GO:0048179	activin receptor complex	CC	6	1	562	7	19809	1.82E-01
GO:0002480	antigen processing and present	BP	8	1	562	7	19809	1.82E-01
GO:0002829	negative regulation of type 2 in	BP	8	1	562	7	19809	1.82E-01
GO:0071014	post-mRNA release spliceoson	CC	7	1	562	7	19809	1.82E-01
GO:0097084	vascular smooth muscle cell de	BP	7	1	562	7	19809	1.82E-01
GO:0046578	regulation of Ras protein signa	BP	10	1	562	7	19809	1.82E-01
GO:0021559	trigeminal nerve development	BP	7	1	562	7	19809	1.82E-01
GO:0035381	ATP-gated ion channel activity	MF	9	1	562	7	19809	1.82E-01
GO:0046581	intercellular canaliculus	CC	5	1	562	7	19809	1.82E-01
GO:0008525	phosphatidylcholine transporte	MF	6	1	562	7	19809	1.82E-01
GO:0007183	SMAD protein complex assem	BP	8	1	562	7	19809	1.82E-01
GO:0038085	vascular endothelial growth fa	MF	6	1	562	7	19809	1.82E-01
GO:0035254	glutamate receptor binding	MF	6	1	562	7	19809	1.82E-01
GO:0051271	negative regulation of cellular	BP	7	1	562	7	19809	1.82E-01
GO:2000987	positive regulation of behavior	BP	9	1	562	7	19809	1.82E-01
GO:0071447	cellular response to hydropero	BP	7	1	562	7	19809	1.82E-01

GO:0120115	Lsm2-8 complex	CC	5	1	562	7	19809	1.82E-01
GO:1990247	N6-methyladenosine-containin	MF	7	1	562	7	19809	1.82E-01
GO:0004931	extracellularly ATP-gated cati	MF	10	1	562	7	19809	1.82E-01
GO:0050688	regulation of defense response	BP	7	1	562	7	19809	1.82E-01
GO:0036435	K48-linked polyubiquitin mod	MF	7	1	562	7	19809	1.82E-01
GO:0045581	negative regulation of T cell di	BP	10	1	562	7	19809	1.82E-01
GO:0036465	synaptic vesicle recycling	BP	6	1	562	7	19809	1.82E-01
GO:0032876	negative regulation of DNA en	BP	9	1	562	7	19809	1.82E-01
GO:0060740	prostate gland epithelium mor	BP	7	1	562	7	19809	1.82E-01
GO:0045409	negative regulation of interleu	BP	9	1	562	7	19809	1.82E-01
GO:0019239	deaminase activity	MF	5	1	562	7	19809	1.82E-01
GO:0035020	regulation of Rac protein signa	BP	11	1	562	7	19809	1.82E-01
GO:0045111	intermediate filament cytoskel	CC	7	3	562	52	19809	1.83E-01
GO:0003007	heart morphogenesis	BP	6	3	562	52	19809	1.83E-01
GO:0051928	positive regulation of calcium	BP	9	2	562	28	19809	1.88E-01
GO:0051453	regulation of intracellular pH	BP	12	2	562	28	19809	1.88E-01
GO:0043113	receptor clustering	BP	6	2	562	28	19809	1.88E-01
GO:0043325	phosphatidylinositol-3,4-bisph	MF	9	2	562	28	19809	1.88E-01
GO:0014047	glutamate secretion	BP	11	2	562	28	19809	1.88E-01
GO:0042744	hydrogen peroxide catabolic p	BP	8	2	562	28	19809	1.88E-01
GO:0007259	receptor signaling pathway via	BP	7	2	562	28	19809	1.88E-01
GO:0007140	male meiotic nuclear division	BP	5	2	562	28	19809	1.88E-01
GO:0005539	glycosaminoglycan binding	MF	5	2	562	28	19809	1.88E-01
GO:0035116	embryonic hindlimb morphoge	BP	8	2	562	28	19809	1.88E-01
GO:0008083	growth factor activity	MF	7	7	562	165	19809	1.89E-01
GO:0004252	serine-type endopeptidase acti	MF	8	7	562	165	19809	1.89E-01
GO:0030659	cytoplasmic vesicle membrane	CC	6	7	562	165	19809	1.89E-01
GO:0071320	cellular response to cAMP	BP	9	3	562	53	19809	1.90E-01
GO:0031295	T cell costimulation	BP	5	3	562	53	19809	1.90E-01
GO:0031252	cell leading edge	CC	4	3	562	53	19809	1.90E-01
GO:0004714	transmembrane receptor protei	MF	9	3	562	53	19809	1.90E-01
GO:0005044	scavenger receptor activity	MF	4	3	562	53	19809	1.90E-01
GO:0007584	response to nutrient	BP	7	4	562	80	19809	1.92E-01
GO:1902600	proton transmembrane transpo	BP	9	5	562	108	19809	1.93E-01
GO:0098793	presynapse	CC	4	6	562	137	19809	1.94E-01
GO:0008237	metallopeptidase activity	MF	7	7	562	167	19809	1.97E-01
GO:0015630	microtubule cytoskeleton	CC	7	7	562	167	19809	1.97E-01
GO:0005802	trans-Golgi network	CC	7	7	562	167	19809	1.97E-01
GO:1903078	positive regulation of protein l	BP	10	3	562	54	19809	1.97E-01
GO:0030838	positive regulation of actin fila	BP	9	3	562	54	19809	1.97E-01
GO:0018149	peptide cross-linking	BP	9	3	562	54	19809	1.97E-01
GO:0009898	cytoplasmic side of plasma me	CC	5	3	562	54	19809	1.97E-01
GO:0047485	protein N-terminus binding	MF	5	5	562	109	19809	1.98E-01
GO:0046875	ephrin receptor binding	MF	6	2	562	29	19809	1.98E-01
GO:0071889	14-3-3 protein binding	MF	5	2	562	29	19809	1.98E-01
GO:0006298	mismatch repair	BP	9	2	562	29	19809	1.98E-01
GO:0003785	actin monomer binding	MF	7	2	562	29	19809	1.98E-01
GO:0046579	positive regulation of Ras prot	BP	11	2	562	29	19809	1.98E-01
GO:0035035	histone acetyltransferase bindi	MF	6	2	562	29	19809	1.98E-01
GO:0048589	developmental growth	BP	4	2	562	29	19809	1.98E-01

GO:1904837	beta-catenin-TCF complex ass	BP	7	2	562	29	19809	1.98E-01
GO:0051281	positive regulation of release c	BP	15	2	562	29	19809	1.98E-01
GO:0035025	positive regulation of Rho prot	BP	12	2	562	29	19809	1.98E-01
GO:0020037	heme binding	MF	6	6	562	138	19809	1.99E-01
GO:0044877	protein-containing complex bi	MF	4	11	562	289	19809	2.00E-01
GO:0043066	negative regulation of apoptoti	BP	9	19	562	542	19809	2.02E-01
GO:0021549	cerebellum development	BP	5	3	562	55	19809	2.05E-01
GO:0006139	nucleobase-containing compou	BP	6	3	562	55	19809	2.05E-01
GO:0043434	response to peptide hormone	BP	6	3	562	55	19809	2.05E-01
GO:1904724	tertiary granule lumen	CC	7	3	562	55	19809	2.05E-01
GO:0071550	death-inducing signaling comp	BP	7	1	562	8	19809	2.06E-01
GO:0042599	lamellar body	CC	9	1	562	8	19809	2.06E-01
GO:0071141	SMAD protein complex	CC	5	1	562	8	19809	2.06E-01
GO:0072577	endothelial cell apoptotic proc	BP	8	1	562	8	19809	2.06E-01
GO:0016229	steroid dehydrogenase activity	MF	5	1	562	8	19809	2.06E-01
GO:0010518	positive regulation of phospho	BP	9	1	562	8	19809	2.06E-01
GO:0051920	peroxiredoxin activity	MF	4	1	562	8	19809	2.06E-01
GO:0060346	bone trabecula formation	BP	6	1	562	8	19809	2.06E-01
GO:0016049	cell growth	BP	4	1	562	8	19809	2.06E-01
GO:0009308	amine metabolic process	BP	6	1	562	8	19809	2.06E-01
GO:1900244	positive regulation of synaptic	BP	9	1	562	8	19809	2.06E-01
GO:0003993	acid phosphatase activity	MF	8	1	562	8	19809	2.06E-01
GO:0006379	mRNA cleavage	BP	10	1	562	8	19809	2.06E-01
GO:0004862	cAMP-dependent protein kinas	MF	9	1	562	8	19809	2.06E-01
GO:0010700	negative regulation of norepin	BP	10	1	562	8	19809	2.06E-01
GO:0051096	positive regulation of helicase	BP	8	1	562	8	19809	2.06E-01
GO:0010882	regulation of cardiac muscle co	BP	8	1	562	8	19809	2.06E-01
GO:0034382	chylomicron remnant clearanc	BP	6	1	562	8	19809	2.06E-01
GO:0006548	histidine catabolic process	BP	11	1	562	8	19809	2.06E-01
GO:0017151	DEAD/H-box RNA helicase b	MF	6	1	562	8	19809	2.06E-01
GO:0035860	glial cell-derived neurotrophic	BP	8	1	562	8	19809	2.06E-01
GO:0070878	primary miRNA binding	MF	7	1	562	8	19809	2.06E-01
GO:0061702	inflammasome complex	CC	4	1	562	8	19809	2.06E-01
GO:0098797	plasma membrane protein com	CC	5	1	562	8	19809	2.06E-01
GO:0034983	peptidyl-lysine deacetylation	BP	11	1	562	8	19809	2.06E-01
GO:0046324	regulation of glucose import	BP	9	1	562	8	19809	2.06E-01
GO:0035767	endothelial cell chemotaxis	BP	10	1	562	8	19809	2.06E-01
GO:0003254	regulation of membrane depola	BP	6	1	562	8	19809	2.06E-01
GO:0042609	CD4 receptor binding	MF	6	1	562	8	19809	2.06E-01
GO:0002115	store-operated calcium entry	BP	11	1	562	8	19809	2.06E-01
GO:0033292	T-tubule organization	BP	6	1	562	8	19809	2.06E-01
GO:0090140	regulation of mitochondrial fis	BP	9	1	562	8	19809	2.06E-01
GO:0018023	peptidyl-lysine trimethylation	BP	12	1	562	8	19809	2.06E-01
GO:0015889	cobalamin transport	BP	7	1	562	8	19809	2.06E-01
GO:1902966	positive regulation of protein l	BP	10	1	562	8	19809	2.06E-01
GO:2000766	negative regulation of cytoplas	BP	11	1	562	8	19809	2.06E-01
GO:1990712	HFE-transferrin receptor comp	CC	6	1	562	8	19809	2.06E-01
GO:0060627	regulation of vesicle-mediated	BP	6	1	562	8	19809	2.06E-01
GO:0004300	enoyl-CoA hydratase activity	MF	7	1	562	8	19809	2.06E-01
GO:0048312	intracellular distribution of mi	BP	8	1	562	8	19809	2.06E-01

GO:0034616	response to laminar fluid shear	BP	6	1	562	8	19809	2.06E-01
GO:0034618	arginine binding	MF	8	1	562	8	19809	2.06E-01
GO:0031995	insulin-like growth factor II bi	MF	7	1	562	8	19809	2.06E-01
GO:0009950	dorsal/ventral axis specificatio	BP	6	1	562	8	19809	2.06E-01
GO:0031209	SCAR complex	CC	4	1	562	8	19809	2.06E-01
GO:0061614	pri-miRNA transcription by RI	BP	10	1	562	8	19809	2.06E-01
GO:0008290	F-actin capping protein comple	CC	4	1	562	8	19809	2.06E-01
GO:1902093	positive regulation of flagellat	BP	8	1	562	8	19809	2.06E-01
GO:1901522	positive regulation of transcrip	BP	11	1	562	8	19809	2.06E-01
GO:0005225	volume-sensitive anion channe	MF	8	1	562	8	19809	2.06E-01
GO:0071499	cellular response to laminar flu	BP	7	1	562	8	19809	2.06E-01
GO:0031402	sodium ion binding	MF	8	1	562	8	19809	2.06E-01
GO:0032736	positive regulation of interleuk	BP	8	1	562	8	19809	2.06E-01
GO:1903169	regulation of calcium ion trans	BP	10	1	562	8	19809	2.06E-01
GO:0008526	phosphatidylinositol transporte	MF	6	1	562	8	19809	2.06E-01
GO:0060907	positive regulation of macroph	BP	9	1	562	8	19809	2.06E-01
GO:0060732	positive regulation of inositol p	BP	10	1	562	8	19809	2.06E-01
GO:0014732	skeletal muscle atrophy	BP	9	1	562	8	19809	2.06E-01
GO:0097400	interleukin-17-mediated signal	BP	7	1	562	8	19809	2.06E-01
GO:0001960	negative regulation of cytokine	BP	9	1	562	8	19809	2.06E-01
GO:0036396	RNA N6-methyladenosine met	CC	6	1	562	8	19809	2.06E-01
GO:2000601	positive regulation of Arp2/3 c	BP	11	1	562	8	19809	2.06E-01
GO:0005688	U6 snRNP	CC	8	1	562	8	19809	2.06E-01
GO:0071169	establishment of protein locali	BP	10	1	562	8	19809	2.06E-01
GO:0006600	creatine metabolic process	BP	6	1	562	8	19809	2.06E-01
GO:0003857	3-hydroxyacyl-CoA dehydroge	MF	7	1	562	8	19809	2.06E-01
GO:0001955	blood vessel maturation	BP	6	1	562	8	19809	2.06E-01
GO:0071420	cellular response to histamine	BP	8	1	562	8	19809	2.06E-01
GO:0048608	reproductive structure develop	BP	5	1	562	8	19809	2.06E-01
GO:0021860	pyramidal neuron developmen	BP	9	1	562	8	19809	2.06E-01
GO:0035590	purinergic nucleotide receptor	BP	7	1	562	8	19809	2.06E-01
GO:0098883	synapse pruning	BP	6	1	562	8	19809	2.06E-01
GO:0090091	positive regulation of extracell	BP	9	1	562	8	19809	2.06E-01
GO:0043576	regulation of respiratory gaseo	BP	6	1	562	8	19809	2.06E-01
GO:0035529	NADH pyrophosphatase activi	MF	9	1	562	8	19809	2.06E-01
GO:0061384	heart trabecula morphogenesis	BP	6	1	562	8	19809	2.06E-01
GO:0051683	establishment of Golgi localiza	BP	7	1	562	8	19809	2.06E-01
GO:0061202	clathrin-sculpted gamma-amin	CC	9	1	562	8	19809	2.06E-01
GO:0016655	oxidoreductase activity, acting	MF	6	1	562	8	19809	2.06E-01
GO:2001259	positive regulation of cation ch	BP	8	1	562	8	19809	2.06E-01
GO:0051646	mitochondrion localization	BP	6	1	562	8	19809	2.06E-01
GO:0010761	fibroblast migration	BP	8	1	562	8	19809	2.06E-01
GO:0003996	acyl-CoA ligase activity	MF	7	1	562	8	19809	2.06E-01
GO:0071340	skeletal muscle acetylcholine-g	BP	7	1	562	8	19809	2.06E-01
GO:0071493	cellular response to UV-B	BP	9	1	562	8	19809	2.06E-01
GO:0033010	paranodal junction	CC	5	1	562	8	19809	2.06E-01
GO:0016176	superoxide-generating NADPH	MF	6	1	562	8	19809	2.06E-01
GO:0030119	AP-type membrane coat adapto	CC	6	1	562	8	19809	2.06E-01
GO:1900246	positive regulation of RIG-I si	BP	8	1	562	8	19809	2.06E-01
GO:0032873	negative regulation of stress-ac	BP	12	1	562	8	19809	2.06E-01

GO:0072678	T cell migration	BP	6	1	562	8	19809	2.06E-01
GO:0071786	endoplasmic reticulum tubular	BP	7	1	562	8	19809	2.06E-01
GO:0035912	dorsal aorta morphogenesis	BP	9	1	562	8	19809	2.06E-01
GO:0070201	regulation of establishment of	BP	7	1	562	8	19809	2.06E-01
GO:0046930	pore complex	CC	5	1	562	8	19809	2.06E-01
GO:0042117	monocyte activation	BP	7	1	562	8	19809	2.06E-01
GO:0070997	neuron death	BP	5	1	562	8	19809	2.06E-01
GO:0006637	acyl-CoA metabolic process	BP	7	2	562	30	19809	2.09E-01
GO:0007274	neuromuscular synaptic transm	BP	10	2	562	30	19809	2.09E-01
GO:0001816	cytokine production	BP	4	2	562	30	19809	2.09E-01
GO:0032715	negative regulation of interleul	BP	8	2	562	30	19809	2.09E-01
GO:0050769	positive regulation of neuroger	BP	9	2	562	30	19809	2.09E-01
GO:0001662	behavioral fear response	BP	7	2	562	30	19809	2.09E-01
GO:2000379	positive regulation of reactive	BP	8	2	562	30	19809	2.09E-01
GO:0043029	T cell homeostasis	BP	6	2	562	30	19809	2.09E-01
GO:0034451	centriolar satellite	CC	7	2	562	30	19809	2.09E-01
GO:0006414	translational elongation	BP	8	2	562	30	19809	2.09E-01
GO:0046329	negative regulation of JNK cas	BP	13	2	562	30	19809	2.09E-01
GO:0042327	positive regulation of phospho	BP	10	2	562	30	19809	2.09E-01
GO:0050852	T cell receptor signaling pathw	BP	8	7	562	170	19809	2.09E-01
GO:0009055	electron transfer activity	MF	5	4	562	83	19809	2.10E-01
GO:0034976	response to endoplasmic reticu	BP	6	4	562	83	19809	2.10E-01
GO:0042542	response to hydrogen peroxide	BP	7	3	562	56	19809	2.12E-01
GO:0099055	integral component of postsyn	CC	8	3	562	56	19809	2.12E-01
GO:0048839	inner ear development	BP	5	3	562	56	19809	2.12E-01
GO:0043679	axon terminus	CC	6	3	562	56	19809	2.12E-01
GO:0046332	SMAD binding	MF	5	3	562	56	19809	2.12E-01
GO:0010951	negative regulation of endopep	BP	11	6	562	141	19809	2.12E-01
GO:0051091	positive regulation of DNA-bi	BP	6	5	562	112	19809	2.13E-01
GO:0016746	transferase activity, transferrin	MF	5	7	562	171	19809	2.13E-01
GO:0008017	microtubule binding	MF	7	9	562	232	19809	2.15E-01
GO:0001228	DNA-binding transcription act	MF	6	15	562	420	19809	2.16E-01
GO:0048662	negative regulation of smooth	BP	8	2	562	31	19809	2.19E-01
GO:0045880	positive regulation of smoothe	BP	9	2	562	31	19809	2.19E-01
GO:0016079	synaptic vesicle exocytosis	BP	9	2	562	31	19809	2.19E-01
GO:0051482	positive regulation of cytosolic	BP	14	2	562	31	19809	2.19E-01
GO:0050807	regulation of synapse organiza	BP	7	2	562	31	19809	2.19E-01
GO:0048856	anatomical structure developm	BP	4	2	562	31	19809	2.19E-01
GO:1990837	sequence-specific double-stran	MF	8	2	562	31	19809	2.19E-01
GO:0001819	positive regulation of cytokine	BP	7	2	562	31	19809	2.19E-01
GO:0050699	WW domain binding	MF	6	2	562	31	19809	2.19E-01
GO:0043542	endothelial cell migration	BP	9	2	562	31	19809	2.19E-01
GO:0001786	phosphatidylserine binding	MF	6	3	562	57	19809	2.19E-01
GO:0019730	antimicrobial humoral respons	BP	6	3	562	57	19809	2.19E-01
GO:0035577	azurophil granule membrane	CC	10	3	562	57	19809	2.19E-01
GO:0007188	adenylate cyclase-modulating	BP	7	3	562	57	19809	2.19E-01
GO:0031901	early endosome membrane	CC	9	6	562	143	19809	2.21E-01
GO:0042742	defense response to bacterium	BP	8	9	562	235	19809	2.25E-01
GO:0006959	humoral immune response	BP	5	3	562	58	19809	2.27E-01
GO:0001889	liver development	BP	7	4	562	86	19809	2.28E-01

GO:0033138	positive regulation of peptidyl-	BP	11	4	562	86	19809	2.28E-01
GO:0010873	positive regulation of choleste	BP	10	1	562	9	19809	2.28E-01
GO:0090650	cellular response to oxygen-gl	BP	8	1	562	9	19809	2.28E-01
GO:0005861	troponin complex	CC	4	1	562	9	19809	2.28E-01
GO:1900004	negative regulation of serine-ty	BP	12	1	562	9	19809	2.28E-01
GO:0086011	membrane repolarization durin	BP	9	1	562	9	19809	2.28E-01
GO:0097091	synaptic vesicle clustering	BP	8	1	562	9	19809	2.28E-01
GO:0048387	negative regulation of retinoic	BP	9	1	562	9	19809	2.28E-01
GO:0032395	MHC class II receptor activity	MF	6	1	562	9	19809	2.28E-01
GO:0051292	nuclear pore complex assembl	BP	8	1	562	9	19809	2.28E-01
GO:2000177	regulation of neural precursor	BP	7	1	562	9	19809	2.28E-01
GO:0004022	alcohol dehydrogenase (NAD)	MF	7	1	562	9	19809	2.28E-01
GO:0048806	genitalia development	BP	6	1	562	9	19809	2.28E-01
GO:0016868	intramolecular transferase acti	MF	6	1	562	9	19809	2.28E-01
GO:0097011	cellular response to granulocyt	BP	8	1	562	9	19809	2.28E-01
GO:0005501	retinoid binding	MF	6	1	562	9	19809	2.28E-01
GO:0033622	integrin activation	BP	7	1	562	9	19809	2.28E-01
GO:0032592	integral component of mitocho	CC	7	1	562	9	19809	2.28E-01
GO:0038113	interleukin-9-mediated signali	BP	7	1	562	9	19809	2.28E-01
GO:0003416	endochondral bone growth	BP	6	1	562	9	19809	2.28E-01
GO:0022417	protein maturation by protein f	BP	5	1	562	9	19809	2.28E-01
GO:0070493	thrombin-activated receptor si	BP	6	1	562	9	19809	2.28E-01
GO:0001574	ganglioside biosynthetic proce	BP	11	1	562	9	19809	2.28E-01
GO:0048048	embryonic eye morphogenesis	BP	7	1	562	9	19809	2.28E-01
GO:0071374	cellular response to parathyroi	BP	7	1	562	9	19809	2.28E-01
GO:0006621	protein retention in ER lumen	BP	9	1	562	9	19809	2.28E-01
GO:0048712	negative regulation of astrocyt	BP	12	1	562	9	19809	2.28E-01
GO:0034405	response to fluid shear stress	BP	5	1	562	9	19809	2.28E-01
GO:0032300	mismatch repair complex	CC	5	1	562	9	19809	2.28E-01
GO:0010226	response to lithium ion	BP	7	1	562	9	19809	2.28E-01
GO:0032059	bleb	CC	6	1	562	9	19809	2.28E-01
GO:0038092	nodal signaling pathway	BP	9	1	562	9	19809	2.28E-01
GO:0010888	negative regulation of lipid sto	BP	6	1	562	9	19809	2.28E-01
GO:0001973	adenosine receptor signaling p	BP	7	1	562	9	19809	2.28E-01
GO:2000304	positive regulation of ceramide	BP	9	1	562	9	19809	2.28E-01
GO:1900027	regulation of ruffle assembly	BP	10	1	562	9	19809	2.28E-01
GO:0045656	negative regulation of monocy	BP	10	1	562	9	19809	2.28E-01
GO:2000650	negative regulation of sodium	BP	8	1	562	9	19809	2.28E-01
GO:0023052	signaling	BP	3	1	562	9	19809	2.28E-01
GO:0099527	postsynapse to nucleus signali	BP	6	1	562	9	19809	2.28E-01
GO:0008354	germ cell migration	BP	7	1	562	9	19809	2.28E-01
GO:1903202	negative regulation of oxidativ	BP	8	1	562	9	19809	2.28E-01
GO:0010594	regulation of endothelial cell n	BP	9	1	562	9	19809	2.28E-01
GO:0070613	regulation of protein processin	BP	9	1	562	9	19809	2.28E-01
GO:0071636	positive regulation of transforr	BP	8	1	562	9	19809	2.28E-01
GO:0051880	G-quadruplex DNA binding	MF	7	1	562	9	19809	2.28E-01
GO:0016045	detection of bacterium	BP	8	1	562	9	19809	2.28E-01
GO:0072089	stem cell proliferation	BP	4	1	562	9	19809	2.28E-01
GO:0040012	regulation of locomotion	BP	5	1	562	9	19809	2.28E-01
GO:0018026	peptidyl-lysine monomethylati	BP	12	1	562	9	19809	2.28E-01

GO:0034702	ion channel complex	CC	6	1	562	9	19809	2.28E-01
GO:0014009	glial cell proliferation	BP	4	1	562	9	19809	2.28E-01
GO:0060429	epithelium development	BP	6	1	562	9	19809	2.28E-01
GO:0035456	response to interferon-beta	BP	7	1	562	9	19809	2.28E-01
GO:0042491	inner ear auditory receptor cell	BP	9	1	562	9	19809	2.28E-01
GO:0000774	adenyl-nucleotide exchange fa	MF	8	1	562	9	19809	2.28E-01
GO:0021984	adenohypophysis development	BP	5	1	562	9	19809	2.28E-01
GO:0006782	protoporphyrinogen IX biosyn	BP	9	1	562	9	19809	2.28E-01
GO:0060539	diaphragm development	BP	8	1	562	9	19809	2.28E-01
GO:0044754	autolysosome	CC	10	1	562	9	19809	2.28E-01
GO:0051782	negative regulation of cell divi	BP	7	1	562	9	19809	2.28E-01
GO:0060087	relaxation of vascular smooth r	BP	8	1	562	9	19809	2.28E-01
GO:0004000	adenosine deaminase activity	MF	7	1	562	9	19809	2.28E-01
GO:0032330	regulation of chondrocyte diffe	BP	7	1	562	9	19809	2.28E-01
GO:0015810	aspartate transmembrane trans	BP	12	1	562	9	19809	2.28E-01
GO:0043567	regulation of insulin-like grow	BP	8	1	562	9	19809	2.28E-01
GO:0045124	regulation of bone resorption	BP	8	1	562	9	19809	2.28E-01
GO:0046620	regulation of organ growth	BP	7	1	562	9	19809	2.28E-01
GO:0050810	regulation of steroid biosynthe	BP	9	1	562	9	19809	2.28E-01
GO:0031985	Golgi cisterna	CC	7	1	562	9	19809	2.28E-01
GO:0035065	regulation of histone acetylatic	BP	10	1	562	9	19809	2.28E-01
GO:0014037	Schwann cell differentiation	BP	7	1	562	9	19809	2.28E-01
GO:0060039	pericardium development	BP	7	1	562	9	19809	2.28E-01
GO:0010544	negative regulation of platelet	BP	9	1	562	9	19809	2.28E-01
GO:0071312	cellular response to alkaloid	BP	8	1	562	9	19809	2.28E-01
GO:0043306	positive regulation of mast cell	BP	10	1	562	9	19809	2.28E-01
GO:0061371	determination of heart left/righ	BP	8	1	562	9	19809	2.28E-01
GO:0048667	cell morphogenesis involved in	BP	8	1	562	9	19809	2.28E-01
GO:0016043	cellular component organizatic	BP	4	1	562	9	19809	2.28E-01
GO:0017162	aryl hydrocarbon receptor binc	MF	6	1	562	9	19809	2.28E-01
GO:0036353	histone H2A-K119 monoubiqu	BP	15	1	562	9	19809	2.28E-01
GO:0043304	regulation of mast cell degranu	BP	9	1	562	9	19809	2.28E-01
GO:0031095	platelet dense tubular network	CC	5	1	562	9	19809	2.28E-01
GO:0003417	growth plate cartilage develop	BP	8	1	562	9	19809	2.28E-01
GO:0097094	craniofacial suture morphogen	BP	5	1	562	9	19809	2.28E-01
GO:0009266	response to temperature stimul	BP	5	1	562	9	19809	2.28E-01
GO:0034774	secretory granule lumen	CC	7	5	562	115	19809	2.28E-01
GO:0046777	protein autophosphorylation	BP	10	7	562	175	19809	2.30E-01
GO:0030041	actin filament polymerization	BP	8	2	562	32	19809	2.30E-01
GO:0048147	negative regulation of fibrobla	BP	8	2	562	32	19809	2.30E-01
GO:0043392	negative regulation of DNA bi	BP	7	2	562	32	19809	2.30E-01
GO:0005921	gap junction	CC	5	2	562	32	19809	2.30E-01
GO:1901223	negative regulation of NIK/NF	BP	10	2	562	32	19809	2.30E-01
GO:0048018	receptor ligand activity	MF	6	2	562	32	19809	2.30E-01
GO:0043278	response to morphine	BP	6	2	562	32	19809	2.30E-01
GO:0038083	peptidyl-tyrosine autophospho	BP	11	2	562	32	19809	2.30E-01
GO:0007528	neuromuscular junction develc	BP	6	2	562	32	19809	2.30E-01
GO:0030336	negative regulation of cell mig	BP	8	6	562	145	19809	2.30E-01
GO:0007507	heart development	BP	6	9	562	237	19809	2.32E-01
GO:0044297	cell body	CC	4	4	562	87	19809	2.34E-01

GO:0046718	viral entry into host cell	BP	9	4	562	87	19809	2.34E-01
GO:0060395	SMAD protein signal transduc	BP	5	3	562	59	19809	2.34E-01
GO:0015631	tubulin binding	MF	6	3	562	59	19809	2.34E-01
GO:0006879	cellular iron ion homeostasis	BP	12	3	562	59	19809	2.34E-01
GO:0032755	positive regulation of interleuk	BP	8	3	562	59	19809	2.34E-01
GO:0006816	calcium ion transport	BP	10	6	562	146	19809	2.35E-01
GO:0018105	peptidyl-serine phosphorylatio	BP	10	6	562	146	19809	2.35E-01
GO:0043085	positive regulation of catalytic	BP	6	4	562	88	19809	2.40E-01
GO:0017157	regulation of exocytosis	BP	7	2	562	33	19809	2.40E-01
GO:0035115	embryonic forelimb morphoge	BP	8	2	562	33	19809	2.40E-01
GO:0030534	adult behavior	BP	4	2	562	33	19809	2.40E-01
GO:0004553	hydrolase activity, hydrolyzing	MF	6	2	562	33	19809	2.40E-01
GO:0022008	neurogenesis	BP	6	3	562	60	19809	2.42E-01
GO:0031234	extrinsic component of cytopla	CC	6	3	562	60	19809	2.42E-01
GO:0005801	cis-Golgi network	CC	6	3	562	60	19809	2.42E-01
GO:0007611	learning or memory	BP	4	3	562	60	19809	2.42E-01
GO:0005096	GTPase activator activity	MF	6	10	562	271	19809	2.42E-01
GO:0030027	lamellipodium	CC	6	7	562	178	19809	2.42E-01
GO:0005911	cell-cell junction	CC	4	7	562	178	19809	2.42E-01
GO:0031902	late endosome membrane	CC	9	5	562	119	19809	2.49E-01
GO:0016525	negative regulation of angioge	BP	8	5	562	119	19809	2.49E-01
GO:1901215	negative regulation of neuron (	BP	8	3	562	61	19809	2.50E-01
GO:0005903	brush border	CC	5	3	562	61	19809	2.50E-01
GO:0016529	sarcoplasmic reticulum	CC	7	3	562	61	19809	2.50E-01
GO:0090084	negative regulation of inclusio	BP	8	1	562	10	19809	2.50E-01
GO:0032041	NAD-dependent histone deace	MF	9	1	562	10	19809	2.50E-01
GO:2001056	positive regulation of cysteine-	BP	12	1	562	10	19809	2.50E-01
GO:0032940	secretion by cell	BP	4	1	562	10	19809	2.50E-01
GO:0071437	invadopodium	CC	6	1	562	10	19809	2.50E-01
GO:0008347	glial cell migration	BP	7	1	562	10	19809	2.50E-01
GO:0060287	epithelial cilium movement inv	BP	8	1	562	10	19809	2.50E-01
GO:0051901	positive regulation of mitochoi	BP	8	1	562	10	19809	2.50E-01
GO:0046621	negative regulation of organ gi	BP	8	1	562	10	19809	2.50E-01
GO:0006569	tryptophan catabolic process	BP	11	1	562	10	19809	2.50E-01
GO:0045793	positive regulation of cell size	BP	7	1	562	10	19809	2.50E-01
GO:0051272	positive regulation of cellular (	BP	7	1	562	10	19809	2.50E-01
GO:0003149	membranous septum morphoge	BP	5	1	562	10	19809	2.50E-01
GO:0031419	cobalamin binding	MF	5	1	562	10	19809	2.50E-01
GO:0042271	susceptibility to natural killer (	BP	9	1	562	10	19809	2.50E-01
GO:0007494	midgut development	BP	7	1	562	10	19809	2.50E-01
GO:0035721	intraciliary retrograde transpor	BP	8	1	562	10	19809	2.50E-01
GO:1990907	beta-catenin-TCF complex	CC	7	1	562	10	19809	2.50E-01
GO:0003810	protein-glutamine gamma-glut	MF	7	1	562	10	19809	2.50E-01
GO:0071723	lipopeptide binding	MF	5	1	562	10	19809	2.50E-01
GO:0021879	forebrain neuron differentiatio	BP	8	1	562	10	19809	2.50E-01
GO:0033539	fatty acid beta-oxidation using	BP	12	1	562	10	19809	2.50E-01
GO:0051918	negative regulation of fibrinoly	BP	9	1	562	10	19809	2.50E-01
GO:0009651	response to salt stress	BP	6	1	562	10	19809	2.50E-01
GO:0033179	proton-transporting V-type AT	CC	6	1	562	10	19809	2.50E-01
GO:0038063	collagen-activated tyrosine kin	BP	8	1	562	10	19809	2.50E-01

GO:0019911	structural constituent of myelir	MF	4	1	562	10	19809	2.50E-01
GO:0097197	tetraspanin-enriched microdon	CC	5	1	562	10	19809	2.50E-01
GO:0034138	toll-like receptor 3 signaling pa	BP	9	1	562	10	19809	2.50E-01
GO:0042053	regulation of dopamine metabo	BP	9	1	562	10	19809	2.50E-01
GO:0016504	peptidase activator activity	MF	6	1	562	10	19809	2.50E-01
GO:0002456	T cell mediated immunity	BP	7	1	562	10	19809	2.50E-01
GO:0038007	netrin-activated signaling path	BP	6	1	562	10	19809	2.50E-01
GO:0006171	cAMP biosynthetic process	BP	12	1	562	10	19809	2.50E-01
GO:0006012	galactose metabolic process	BP	8	1	562	10	19809	2.50E-01
GO:0005832	chaperonin-containing T-comp	CC	7	1	562	10	19809	2.50E-01
GO:0001706	endoderm formation	BP	6	1	562	10	19809	2.50E-01
GO:2000480	negative regulation of cAMP-c	BP	13	1	562	10	19809	2.50E-01
GO:0010907	positive regulation of glucose r	BP	10	1	562	10	19809	2.50E-01
GO:0006972	hyperosmotic response	BP	6	1	562	10	19809	2.50E-01
GO:0090394	negative regulation of excitato	BP	9	1	562	10	19809	2.50E-01
GO:0051428	peptide hormone receptor bind	MF	7	1	562	10	19809	2.50E-01
GO:0046887	positive regulation of hormone	BP	8	1	562	10	19809	2.50E-01
GO:0004859	phospholipase inhibitor activit	MF	7	1	562	10	19809	2.50E-01
GO:0071313	cellular response to caffeine	BP	7	1	562	10	19809	2.50E-01
GO:0001162	RNA polymerase II intronic tra	MF	10	1	562	10	19809	2.50E-01
GO:0045741	positive regulation of epiderma	BP	10	1	562	10	19809	2.50E-01
GO:1990416	cellular response to brain-deriv	BP	9	1	562	10	19809	2.50E-01
GO:0008190	eukaryotic initiation factor 4E	MF	6	1	562	10	19809	2.50E-01
GO:0033619	membrane protein proteolysis	BP	8	1	562	10	19809	2.50E-01
GO:0050663	cytokine secretion	BP	11	1	562	10	19809	2.50E-01
GO:0030510	regulation of BMP signaling pa	BP	10	1	562	10	19809	2.50E-01
GO:0031053	primary miRNA processing	BP	11	1	562	10	19809	2.50E-01
GO:0030432	peristalsis	BP	9	1	562	10	19809	2.50E-01
GO:0050795	regulation of behavior	BP	5	1	562	10	19809	2.50E-01
GO:0150077	regulation of neuroinflammato	BP	9	1	562	10	19809	2.50E-01
GO:0097062	dendritic spine maintenance	BP	6	1	562	10	19809	2.50E-01
GO:0097484	dendrite extension	BP	7	1	562	10	19809	2.50E-01
GO:0032525	somite rostral/caudal axis spec	BP	7	1	562	10	19809	2.50E-01
GO:0033690	positive regulation of osteobla	BP	8	1	562	10	19809	2.50E-01
GO:0009101	glycoprotein biosynthetic proc	BP	8	1	562	10	19809	2.50E-01
GO:0045416	positive regulation of interleuk	BP	9	1	562	10	19809	2.50E-01
GO:1990124	messenger ribonucleoprotein c	CC	5	1	562	10	19809	2.50E-01
GO:0071470	cellular response to osmotic st	BP	6	1	562	10	19809	2.50E-01
GO:0002827	positive regulation of T-helper	BP	10	1	562	10	19809	2.50E-01
GO:0016188	synaptic vesicle maturation	BP	7	1	562	10	19809	2.50E-01
GO:0046425	regulation of receptor signaling	BP	9	1	562	10	19809	2.50E-01
GO:0046888	negative regulation of hormone	BP	8	1	562	10	19809	2.50E-01
GO:0097066	response to thyroid hormone	BP	6	1	562	10	19809	2.50E-01
GO:0046641	positive regulation of alpha-be	BP	12	1	562	10	19809	2.50E-01
GO:0070723	response to cholesterol	BP	8	1	562	10	19809	2.50E-01
GO:0042118	endothelial cell activation	BP	5	1	562	10	19809	2.50E-01
GO:0016175	superoxide-generating NADPH	MF	7	1	562	10	19809	2.50E-01
GO:0004966	galanin receptor activity	MF	8	1	562	10	19809	2.50E-01
GO:0005883	neurofilament	CC	8	1	562	10	19809	2.50E-01
GO:0002082	regulation of oxidative phosph	BP	10	1	562	10	19809	2.50E-01

GO:0097449	astrocyte projection	CC	7	1	562	10	19809	2.50E-01
GO:0060272	embryonic skeletal joint morpl	BP	8	1	562	10	19809	2.50E-01
GO:0003091	renal water homeostasis	BP	6	2	562	34	19809	2.51E-01
GO:0014065	phosphatidylinositol 3-kinase s	BP	8	2	562	34	19809	2.51E-01
GO:0051973	positive regulation of telomera	BP	8	2	562	34	19809	2.51E-01
GO:0001530	lipopolysaccharide binding	MF	5	2	562	34	19809	2.51E-01
GO:0051930	regulation of sensory perceptic	BP	9	2	562	34	19809	2.51E-01
GO:0010975	regulation of neuron projectior	BP	10	2	562	34	19809	2.51E-01
GO:0007029	endoplasmic reticulum organiz	BP	6	2	562	34	19809	2.51E-01
GO:0048536	spleen development	BP	7	2	562	34	19809	2.51E-01
GO:0021510	spinal cord development	BP	6	2	562	34	19809	2.51E-01
GO:0048701	embryonic cranial skeleton mo	BP	8	2	562	34	19809	2.51E-01
GO:0035579	specific granule membrane	CC	8	4	562	90	19809	2.52E-01
GO:0046982	protein heterodimerization acti	MF	6	18	562	532	19809	2.54E-01
GO:0001822	kidney development	BP	6	5	562	120	19809	2.55E-01
GO:0001523	retinoid metabolic process	BP	10	3	562	62	19809	2.57E-01
GO:1901224	positive regulation of NIK/NF-	BP	10	3	562	62	19809	2.57E-01
GO:0045177	apical part of cell	CC	4	4	562	91	19809	2.58E-01
GO:0008092	cytoskeletal protein binding	MF	5	4	562	91	19809	2.58E-01
GO:0030032	lamellipodium assembly	BP	8	2	562	35	19809	2.62E-01
GO:0043200	response to amino acid	BP	6	2	562	35	19809	2.62E-01
GO:0042059	negative regulation of epiderm	BP	10	2	562	35	19809	2.62E-01
GO:0035924	cellular response to vascular e	BP	8	2	562	35	19809	2.62E-01
GO:1900026	positive regulation of substrate	BP	10	2	562	35	19809	2.62E-01
GO:0003700	DNA-binding transcription fac	MF	4	29	562	899	19809	2.63E-01
GO:0006915	apoptotic process	BP	6	23	562	700	19809	2.63E-01
GO:0060071	Wnt signaling pathway, planar	BP	9	4	562	92	19809	2.65E-01
GO:0018107	peptidyl-threonine phosphoryl:	BP	10	3	562	63	19809	2.65E-01
GO:0043627	response to estrogen	BP	4	3	562	63	19809	2.65E-01
GO:0034605	cellular response to heat	BP	6	3	562	63	19809	2.65E-01
GO:0005777	peroxisome	CC	7	5	562	122	19809	2.65E-01
GO:0001867	complement activation, lectin j	BP	6	1	562	11	19809	2.71E-01
GO:0045899	positive regulation of RNA po	BP	8	1	562	11	19809	2.71E-01
GO:0003995	acyl-CoA dehydrogenase activ	MF	6	1	562	11	19809	2.71E-01
GO:0015643	toxic substance binding	MF	4	1	562	11	19809	2.71E-01
GO:0006751	glutathione catabolic process	BP	9	1	562	11	19809	2.71E-01
GO:0035137	hindlimb morphogenesis	BP	7	1	562	11	19809	2.71E-01
GO:0001054	RNA polymerase I activity	MF	10	1	562	11	19809	2.71E-01
GO:0048143	astrocyte activation	BP	6	1	562	11	19809	2.71E-01
GO:0002221	pattern recognition receptor si	BP	7	1	562	11	19809	2.71E-01
GO:0060339	negative regulation of type I in	BP	10	1	562	11	19809	2.71E-01
GO:0070933	histone H4 deacetylation	BP	12	1	562	11	19809	2.71E-01
GO:0090557	establishment of endothelial in	BP	9	1	562	11	19809	2.71E-01
GO:0060055	angiogenesis involved in wour	BP	6	1	562	11	19809	2.71E-01
GO:0005391	sodium:potassium-exchanging	MF	11	1	562	11	19809	2.71E-01
GO:0048935	peripheral nervous system neu	BP	7	1	562	11	19809	2.71E-01
GO:0051974	negative regulation of telomer:	BP	8	1	562	11	19809	2.71E-01
GO:0033691	sialic acid binding	MF	7	1	562	11	19809	2.71E-01
GO:0097386	glial cell projection	CC	6	1	562	11	19809	2.71E-01
GO:0035457	cellular response to interferon-	BP	8	1	562	11	19809	2.71E-01

GO:0043030	regulation of macrophage activ	BP	7	1	562	11	19809	2.71E-01
GO:0030955	potassium ion binding	MF	8	1	562	11	19809	2.71E-01
GO:0042116	macrophage activation	BP	7	1	562	11	19809	2.71E-01
GO:0015279	store-operated calcium channe	MF	9	1	562	11	19809	2.71E-01
GO:0031253	cell projection membrane	CC	5	1	562	11	19809	2.71E-01
GO:2000637	positive regulation of gene sile	BP	8	1	562	11	19809	2.71E-01
GO:0060100	positive regulation of phagocy	BP	9	1	562	11	19809	2.71E-01
GO:0045176	apical protein localization	BP	6	1	562	11	19809	2.71E-01
GO:0048038	quinone binding	MF	5	1	562	11	19809	2.71E-01
GO:0007168	receptor guanylyl cyclase sign	BP	7	1	562	11	19809	2.71E-01
GO:0150078	positive regulation of neuroinf	BP	10	1	562	11	19809	2.71E-01
GO:0048681	negative regulation of axon re	BP	8	1	562	11	19809	2.71E-01
GO:0046135	pyrimidine nucleoside cataboli	BP	10	1	562	11	19809	2.71E-01
GO:0005742	mitochondrial outer membrane	CC	6	1	562	11	19809	2.71E-01
GO:0030033	microvillus assembly	BP	8	1	562	11	19809	2.71E-01
GO:0006048	UDP-N-acetylglucosamine bio	BP	8	1	562	11	19809	2.71E-01
GO:2000273	positive regulation of signaling	BP	9	1	562	11	19809	2.71E-01
GO:0003016	respiratory system process	BP	5	1	562	11	19809	2.71E-01
GO:0007256	activation of JNKK activity	BP	14	1	562	11	19809	2.71E-01
GO:0031665	negative regulation of lipopoly	BP	8	1	562	11	19809	2.71E-01
GO:0008199	ferric iron binding	MF	9	1	562	11	19809	2.71E-01
GO:0045723	positive regulation of fatty aci	BP	9	1	562	11	19809	2.71E-01
GO:0004016	adenylate cyclase activity	MF	5	1	562	11	19809	2.71E-01
GO:0003094	glomerular filtration	BP	7	1	562	11	19809	2.71E-01
GO:0010738	regulation of protein kinase A	BP	9	1	562	11	19809	2.71E-01
GO:0072425	signal transduction involved in	BP	9	1	562	11	19809	2.71E-01
GO:0046512	sphingosine biosynthetic proce	BP	9	1	562	11	19809	2.71E-01
GO:0001012	RNA polymerase II regulatory	MF	8	1	562	11	19809	2.71E-01
GO:0019371	cyclooxygenase pathway	BP	12	1	562	11	19809	2.71E-01
GO:0071203	WASH complex	CC	4	1	562	11	19809	2.71E-01
GO:0043129	surfactant homeostasis	BP	9	1	562	11	19809	2.71E-01
GO:0060306	regulation of membrane repola	BP	9	1	562	11	19809	2.71E-01
GO:0000050	urea cycle	BP	8	1	562	11	19809	2.71E-01
GO:0090150	establishment of protein locali	BP	7	1	562	11	19809	2.71E-01
GO:0002281	macrophage activation involve	BP	8	1	562	11	19809	2.71E-01
GO:1901380	negative regulation of potassiu	BP	11	1	562	11	19809	2.71E-01
GO:1901612	cardiolipin binding	MF	7	1	562	11	19809	2.71E-01
GO:0001893	maternal placenta developmen	BP	5	1	562	11	19809	2.71E-01
GO:0043616	keratinocyte proliferation	BP	5	1	562	11	19809	2.71E-01
GO:0060253	negative regulation of glial cel	BP	8	1	562	11	19809	2.71E-01
GO:0099173	postsynapse organization	BP	5	1	562	11	19809	2.71E-01
GO:0010737	protein kinase A signaling	BP	6	1	562	11	19809	2.71E-01
GO:0060009	Sertoli cell development	BP	7	1	562	11	19809	2.71E-01
GO:0045577	regulation of B cell differentia	BP	9	1	562	11	19809	2.71E-01
GO:0030296	protein tyrosine kinase activat	MF	8	1	562	11	19809	2.71E-01
GO:0016918	retinal binding	MF	7	1	562	11	19809	2.71E-01
GO:0034379	very-low-density lipoprotein p	BP	9	1	562	11	19809	2.71E-01
GO:0045637	regulation of myeloid cell diff	BP	7	1	562	11	19809	2.71E-01
GO:0009048	dosage compensation by inacti	BP	10	1	562	11	19809	2.71E-01
GO:0060452	positive regulation of cardiac r	BP	10	1	562	11	19809	2.71E-01

GO:0022407	regulation of cell-cell adhesior	BP	6	1	562	11	19809	2.71E-01
GO:0033327	Leydig cell differentiation	BP	5	1	562	11	19809	2.71E-01
GO:0009314	response to radiation	BP	5	2	562	36	19809	2.72E-01
GO:0007369	gastrulation	BP	6	2	562	36	19809	2.72E-01
GO:0030173	integral component of Golgi m	CC	7	3	562	64	19809	2.73E-01
GO:0045165	cell fate commitment	BP	5	3	562	64	19809	2.73E-01
GO:0051082	unfolded protein binding	MF	5	5	562	124	19809	2.76E-01
GO:0000902	cell morphogenesis	BP	6	4	562	94	19809	2.77E-01
GO:0045596	negative regulation of cell diff	BP	7	3	562	65	19809	2.81E-01
GO:0019898	extrinsic component of membr	CC	4	3	562	65	19809	2.81E-01
GO:0030374	nuclear receptor transcription	MF	6	3	562	65	19809	2.81E-01
GO:0001937	negative regulation of endothe	BP	9	2	562	37	19809	2.83E-01
GO:0007223	Wnt signaling pathway, calciu	BP	9	2	562	37	19809	2.83E-01
GO:1901998	toxin transport	BP	6	2	562	37	19809	2.83E-01
GO:0000987	proximal promoter sequence-s	MF	9	2	562	37	19809	2.83E-01
GO:0010667	negative regulation of cardiac	BP	12	2	562	37	19809	2.83E-01
GO:0072593	reactive oxygen species metab	BP	5	2	562	37	19809	2.83E-01
GO:0010634	positive regulation of epithelia	BP	9	2	562	37	19809	2.83E-01
GO:0019888	protein phosphatase regulator	MF	6	2	562	37	19809	2.83E-01
GO:0031214	biomineral tissue development	BP	6	2	562	37	19809	2.83E-01
GO:0007005	mitochondrion organization	BP	6	4	562	95	19809	2.84E-01
GO:0000122	negative regulation of transcrip	BP	10	26	562	811	19809	2.88E-01
GO:0050679	positive regulation of epithelia	BP	8	3	562	66	19809	2.88E-01
GO:0060079	excitatory postsynaptic potenti	BP	7	3	562	66	19809	2.88E-01
GO:0043005	neuron projection	CC	5	14	562	414	19809	2.89E-01
GO:0032869	cellular response to insulin stir	BP	8	4	562	96	19809	2.90E-01
GO:0002237	response to molecule of bacter	BP	6	1	562	12	19809	2.92E-01
GO:1904668	positive regulation of ubiquitir	BP	13	1	562	12	19809	2.92E-01
GO:0070410	co-SMAD binding	MF	6	1	562	12	19809	2.92E-01
GO:0040015	negative regulation of multicel	BP	8	1	562	12	19809	2.92E-01
GO:0004126	cytidine deaminase activity	MF	7	1	562	12	19809	2.92E-01
GO:0043586	tongue development	BP	7	1	562	12	19809	2.92E-01
GO:0032026	response to magnesium ion	BP	7	1	562	12	19809	2.92E-01
GO:2001171	positive regulation of ATP bio	BP	12	1	562	12	19809	2.92E-01
GO:0031994	insulin-like growth factor I bin	MF	7	1	562	12	19809	2.92E-01
GO:0016790	thiolester hydrolase activity	MF	6	1	562	12	19809	2.92E-01
GO:0043031	negative regulation of macropl	BP	8	1	562	12	19809	2.92E-01
GO:0051481	negative regulation of cytosoli	BP	13	1	562	12	19809	2.92E-01
GO:0030212	hyaluronan metabolic process	BP	8	1	562	12	19809	2.92E-01
GO:0050927	positive regulation of positive	BP	9	1	562	12	19809	2.92E-01
GO:0004033	aldo-keto reductase (NADP) a	MF	7	1	562	12	19809	2.92E-01
GO:0005845	mRNA cap binding complex	CC	5	1	562	12	19809	2.92E-01
GO:0001945	lymph vessel development	BP	5	1	562	12	19809	2.92E-01
GO:0090161	Golgi ribbon formation	BP	7	1	562	12	19809	2.92E-01
GO:0030208	dermatan sulfate biosynthetic	BP	9	1	562	12	19809	2.92E-01
GO:0019363	pyridine nucleotide biosynthet	BP	8	1	562	12	19809	2.92E-01
GO:0032434	regulation of proteasomal ubiq	BP	11	1	562	12	19809	2.92E-01
GO:0003181	atrioventricular valve morphog	BP	6	1	562	12	19809	2.92E-01
GO:0005751	mitochondrial respiratory chai	CC	6	1	562	12	19809	2.92E-01
GO:0007096	regulation of exit from mitosis	BP	9	1	562	12	19809	2.92E-01

GO:0010818	T cell chemotaxis	BP	7	1	562	12	19809	2.92E-01
GO:0060391	positive regulation of SMAD p	BP	10	1	562	12	19809	2.92E-01
GO:1902282	voltage-gated potassium chann	MF	11	1	562	12	19809	2.92E-01
GO:0042592	homeostatic process	BP	5	1	562	12	19809	2.92E-01
GO:0048016	inositol phosphate-mediated si	BP	7	1	562	12	19809	2.92E-01
GO:0003215	cardiac right ventricle morpho	BP	7	1	562	12	19809	2.92E-01
GO:0048407	platelet-derived growth factor	MF	6	1	562	12	19809	2.92E-01
GO:0071679	commissural neuron axon guid	BP	7	1	562	12	19809	2.92E-01
GO:0031953	negative regulation of protein a	BP	11	1	562	12	19809	2.92E-01
GO:0071372	cellular response to follicle-sti	BP	8	1	562	12	19809	2.92E-01
GO:0097153	cysteine-type endopeptidase ac	MF	9	1	562	12	19809	2.92E-01
GO:0006596	polyamine biosynthetic proces	BP	10	1	562	12	19809	2.92E-01
GO:0016810	hydrolase activity, acting on ca	MF	5	1	562	12	19809	2.92E-01
GO:0046785	microtubule polymerization	BP	9	1	562	12	19809	2.92E-01
GO:0043229	intracellular organelle	CC	4	1	562	12	19809	2.92E-01
GO:0016554	cytidine to uridine editing	BP	11	1	562	12	19809	2.92E-01
GO:0051549	positive regulation of keratino	BP	10	1	562	12	19809	2.92E-01
GO:0045410	positive regulation of interleuk	BP	9	1	562	12	19809	2.92E-01
GO:2001022	positive regulation of response	BP	7	1	562	12	19809	2.92E-01
GO:0015867	ATP transport	BP	9	1	562	12	19809	2.92E-01
GO:1900242	regulation of synaptic vesicle e	BP	8	1	562	12	19809	2.92E-01
GO:0016818	hydrolase activity, acting on ac	MF	6	1	562	12	19809	2.92E-01
GO:0060394	negative regulation of pathway	BP	11	1	562	12	19809	2.92E-01
GO:0030203	glycosaminoglycan metabolic	BP	7	1	562	12	19809	2.92E-01
GO:0045830	positive regulation of isotype s	BP	10	1	562	12	19809	2.92E-01
GO:0072687	meiotic spindle	CC	7	1	562	12	19809	2.92E-01
GO:0016290	palmitoyl-CoA hydrolase activ	MF	9	1	562	12	19809	2.92E-01
GO:0014075	response to amine	BP	7	1	562	12	19809	2.92E-01
GO:0033558	protein deacetylase activity	MF	6	1	562	12	19809	2.92E-01
GO:0004115	3',5'-cyclic-AMP phosphodiest	MF	10	1	562	12	19809	2.92E-01
GO:0043922	negative regulation by host of	BP	10	1	562	12	19809	2.92E-01
GO:0033700	phospholipid efflux	BP	9	1	562	12	19809	2.92E-01
GO:0043220	Schmidt-Lanterman incisure	CC	4	1	562	12	19809	2.92E-01
GO:0005890	sodium:potassium-exchanging	CC	8	1	562	12	19809	2.92E-01
GO:0038180	nerve growth factor signaling p	BP	7	1	562	12	19809	2.92E-01
GO:0004861	cyclin-dependent protein serin	MF	8	1	562	12	19809	2.92E-01
GO:0009972	cytidine deamination	BP	13	1	562	12	19809	2.92E-01
GO:0071564	npBAF complex	CC	8	1	562	12	19809	2.92E-01
GO:0098915	membrane repolarization durin	BP	11	1	562	12	19809	2.92E-01
GO:0005736	RNA polymerase I complex	CC	7	1	562	12	19809	2.92E-01
GO:1904385	cellular response to angiotensi	BP	8	1	562	12	19809	2.92E-01
GO:0042770	signal transduction in response	BP	7	1	562	12	19809	2.92E-01
GO:0009058	biosynthetic process	BP	4	2	562	38	19809	2.93E-01
GO:0006493	protein O-linked glycosylation	BP	10	2	562	38	19809	2.93E-01
GO:0004601	peroxidase activity	MF	4	2	562	38	19809	2.93E-01
GO:0043531	ADP binding	MF	9	2	562	38	19809	2.93E-01
GO:0048487	beta-tubulin binding	MF	7	2	562	38	19809	2.93E-01
GO:0032467	positive regulation of cytokine	BP	9	2	562	38	19809	2.93E-01
GO:0030049	muscle filament sliding	BP	8	2	562	38	19809	2.93E-01
GO:0071333	cellular response to glucose sti	BP	10	3	562	67	19809	2.96E-01

GO:0005791	rough endoplasmic reticulum	CC	7	3	562	67	19809	2.96E-01
GO:0051384	response to glucocorticoid	BP	8	3	562	67	19809	2.96E-01
GO:0016791	phosphatase activity	MF	7	5	562	128	19809	2.98E-01
GO:0001726	ruffle	CC	6	4	562	98	19809	3.03E-01
GO:1902036	regulation of hematopoietic ste	BP	8	3	562	68	19809	3.04E-01
GO:0006487	protein N-linked glycosylation	BP	10	2	562	39	19809	3.04E-01
GO:0031226	intrinsic component of plasma	CC	5	2	562	39	19809	3.04E-01
GO:0046034	ATP metabolic process	BP	4	2	562	39	19809	3.04E-01
GO:0043491	protein kinase B signaling	BP	6	2	562	39	19809	3.04E-01
GO:0070555	response to interleukin-1	BP	7	2	562	39	19809	3.04E-01
GO:0005109	frizzled binding	MF	7	2	562	39	19809	3.04E-01
GO:0051260	protein homooligomerization	BP	8	8	562	225	19809	3.08E-01
GO:0000082	G1/S transition of mitotic cell	BP	7	4	562	99	19809	3.09E-01
GO:0018108	peptidyl-tyrosine phosphorylat	BP	10	5	562	130	19809	3.09E-01
GO:0070588	calcium ion transmembrane tra	BP	11	5	562	130	19809	3.09E-01
GO:0035690	cellular response to drug	BP	6	3	562	69	19809	3.12E-01
GO:0006986	response to unfolded protein	BP	6	3	562	69	19809	3.12E-01
GO:0043020	NADPH oxidase complex	CC	6	1	562	13	19809	3.12E-01
GO:0004629	phospholipase C activity	MF	8	1	562	13	19809	3.12E-01
GO:0060389	pathway-restricted SMAD pro	BP	10	1	562	13	19809	3.12E-01
GO:0045086	positive regulation of interleuk	BP	9	1	562	13	19809	3.12E-01
GO:0047617	acyl-CoA hydrolase activity	MF	8	1	562	13	19809	3.12E-01
GO:0045744	negative regulation of G protei	BP	9	1	562	13	19809	3.12E-01
GO:0060674	placenta blood vessel developr	BP	6	1	562	13	19809	3.12E-01
GO:0034214	protein hexamerization	BP	8	1	562	13	19809	3.12E-01
GO:0021756	striatum development	BP	5	1	562	13	19809	3.12E-01
GO:0008093	cytoskeletal adaptor activity	MF	6	1	562	13	19809	3.12E-01
GO:0060993	kidney morphogenesis	BP	6	1	562	13	19809	3.12E-01
GO:0071425	hematopoietic stem cell prolife	BP	5	1	562	13	19809	3.12E-01
GO:0070306	lens fiber cell differentiation	BP	7	1	562	13	19809	3.12E-01
GO:0060397	growth hormone receptor signa	BP	9	1	562	13	19809	3.12E-01
GO:0032479	regulation of type I interferon p	BP	7	1	562	13	19809	3.12E-01
GO:0045806	negative regulation of endocyt	BP	8	1	562	13	19809	3.12E-01
GO:0097067	cellular response to thyroid ho	BP	7	1	562	13	19809	3.12E-01
GO:0061029	eyelid development in camera-	BP	5	1	562	13	19809	3.12E-01
GO:0070679	inositol 1,4,5 trisphosphate bir	MF	6	1	562	13	19809	3.12E-01
GO:0014902	myotube differentiation	BP	8	1	562	13	19809	3.12E-01
GO:0044237	cellular metabolic process	BP	4	1	562	13	19809	3.12E-01
GO:0010524	positive regulation of calcium	BP	14	1	562	13	19809	3.12E-01
GO:0008645	hexose transmembrane transpo	BP	10	1	562	13	19809	3.12E-01
GO:2000146	negative regulation of cell mot	BP	7	1	562	13	19809	3.12E-01
GO:0070528	protein kinase C signaling	BP	6	1	562	13	19809	3.12E-01
GO:1900271	regulation of long-term synapt	BP	10	1	562	13	19809	3.12E-01
GO:0030516	regulation of axon extension	BP	7	1	562	13	19809	3.12E-01
GO:0007171	activation of transmembrane re	BP	13	1	562	13	19809	3.12E-01
GO:0031529	ruffle organization	BP	7	1	562	13	19809	3.12E-01
GO:0008553	proton-exporting ATPase activ	MF	10	1	562	13	19809	3.12E-01
GO:0019430	removal of superoxide radicals	BP	7	1	562	13	19809	3.12E-01
GO:0030007	cellular potassium ion homeos	BP	11	1	562	13	19809	3.12E-01
GO:0042581	specific granule	CC	9	1	562	13	19809	3.12E-01

GO:0045779	negative regulation of bone res	BP	9	1	562	13	19809	3.12E-01
GO:0036376	sodium ion export across plasr	BP	11	1	562	13	19809	3.12E-01
GO:0001967	suckling behavior	BP	5	1	562	13	19809	3.12E-01
GO:0050708	regulation of protein secretion	BP	9	1	562	13	19809	3.12E-01
GO:0051489	regulation of filopodium assen	BP	10	1	562	13	19809	3.12E-01
GO:2000772	regulation of cellular senescen	BP	7	1	562	13	19809	3.12E-01
GO:0042641	actomyosin	CC	6	1	562	13	19809	3.12E-01
GO:0005885	Arp2/3 protein complex	CC	4	1	562	13	19809	3.12E-01
GO:0003323	type B pancreatic cell developı	BP	9	1	562	13	19809	3.12E-01
GO:0032230	positive regulation of synaptic	BP	10	1	562	13	19809	3.12E-01
GO:0060074	synapse maturation	BP	5	1	562	13	19809	3.12E-01
GO:0016024	CDP-diacylglycerol biosynthei	BP	11	1	562	13	19809	3.12E-01
GO:0014912	negative regulation of smooth	BP	9	1	562	13	19809	3.12E-01
GO:0050713	negative regulation of interleul	BP	10	1	562	13	19809	3.12E-01
GO:0042761	very long-chain fatty acid bios	BP	11	1	562	13	19809	3.12E-01
GO:0035308	negative regulation of protein (	BP	10	1	562	13	19809	3.12E-01
GO:0048246	macrophage chemotaxis	BP	6	1	562	13	19809	3.12E-01
GO:0045078	positive regulation of interfero	BP	9	1	562	13	19809	3.12E-01
GO:2001256	regulation of store-operated ca	BP	10	1	562	13	19809	3.12E-01
GO:0006809	nitric oxide biosynthetic proce	BP	8	1	562	13	19809	3.12E-01
GO:0017017	MAP kinase tyrosine/serine/th	MF	10	1	562	13	19809	3.12E-01
GO:2000678	negative regulation of transcrij	BP	8	1	562	13	19809	3.12E-01
GO:0008210	estrogen metabolic process	BP	7	1	562	13	19809	3.12E-01
GO:0048333	mesodermal cell differentiatioı	BP	6	1	562	13	19809	3.12E-01
GO:0090399	replicative senescence	BP	6	1	562	13	19809	3.12E-01
GO:2000009	negative regulation of protein l	BP	7	1	562	13	19809	3.12E-01
GO:0048484	enteric nervous system develoj	BP	6	1	562	13	19809	3.12E-01
GO:0031076	embryonic camera-type eye de	BP	9	1	562	13	19809	3.12E-01
GO:1900272	negative regulation of long-ter	BP	6	1	562	13	19809	3.12E-01
GO:0009303	rRNA transcription	BP	10	1	562	13	19809	3.12E-01
GO:0061052	negative regulation of cell grov	BP	11	1	562	13	19809	3.12E-01
GO:0070986	left/right axis specification	BP	6	1	562	13	19809	3.12E-01
GO:1902894	negative regulation of pri-miR	BP	11	1	562	13	19809	3.12E-01
GO:0032233	positive regulation of actin fila	BP	7	1	562	13	19809	3.12E-01
GO:0010839	negative regulation of keratino	BP	9	1	562	13	19809	3.12E-01
GO:0008154	actin polymerization or depoly	BP	7	1	562	13	19809	3.12E-01
GO:0043281	regulation of cysteine-type enc	BP	9	1	562	13	19809	3.12E-01
GO:0071257	cellular response to electrical s	BP	6	1	562	13	19809	3.12E-01
GO:1901216	positive regulation of neuron d	BP	8	2	562	40	19809	3.14E-01
GO:0007346	regulation of mitotic cell cycle	BP	7	2	562	40	19809	3.14E-01
GO:0031623	receptor internalization	BP	9	2	562	40	19809	3.14E-01
GO:2001244	positive regulation of intrinsic	BP	10	2	562	40	19809	3.14E-01
GO:0045071	negative regulation of viral geı	BP	10	2	562	40	19809	3.14E-01
GO:0007420	brain development	BP	6	9	562	259	19809	3.15E-01
GO:0004672	protein kinase activity	MF	7	18	562	556	19809	3.16E-01
GO:0070301	cellular response to hydrogen ן	BP	8	3	562	70	19809	3.19E-01
GO:0016323	basolateral plasma membrane	CC	5	7	562	196	19809	3.22E-01
GO:0000981	DNA-binding transcription fac	MF	5	47	562	1541	19809	3.22E-01
GO:0005783	endoplasmic reticulum	CC	6	46	562	1508	19809	3.24E-01
GO:0007190	activation of adenylate cyclase	BP	6	2	562	41	19809	3.25E-01

GO:1904646	cellular response to amyloid-b	BP	9	2	562	41	19809	3.25E-01
GO:0009566	fertilization	BP	4	2	562	41	19809	3.25E-01
GO:0008528	G protein-coupled peptide rece	MF	6	2	562	41	19809	3.25E-01
GO:0030020	extracellular matrix structural	MF	5	2	562	41	19809	3.25E-01
GO:0004518	nuclease activity	MF	6	5	562	133	19809	3.26E-01
GO:0016853	isomerase activity	MF	4	5	562	133	19809	3.26E-01
GO:0043231	intracellular membrane-bound	CC	5	25	562	795	19809	3.26E-01
GO:0035725	sodium ion transmembrane tra	BP	10	3	562	71	19809	3.27E-01
GO:0036464	cytoplasmic ribonucleoprotein	CC	7	3	562	71	19809	3.27E-01
GO:0032956	regulation of actin cytoskeleto	BP	7	3	562	71	19809	3.27E-01
GO:0004725	protein tyrosine phosphatase a	MF	9	4	562	102	19809	3.29E-01
GO:0030141	secretory granule	CC	8	4	562	102	19809	3.29E-01
GO:0005622	intracellular	CC	4	23	562	729	19809	3.30E-01
GO:0060416	response to growth hormone	BP	7	1	562	14	19809	3.32E-01
GO:0070403	NAD+ binding	MF	6	1	562	14	19809	3.32E-01
GO:0043149	stress fiber assembly	BP	8	1	562	14	19809	3.32E-01
GO:0043023	ribosomal large subunit bindin	MF	6	1	562	14	19809	3.32E-01
GO:0043274	phospholipase binding	MF	6	1	562	14	19809	3.32E-01
GO:1902043	positive regulation of extrinsic	BP	11	1	562	14	19809	3.32E-01
GO:0060411	cardiac septum morphogenesis	BP	5	1	562	14	19809	3.32E-01
GO:0042551	neuron maturation	BP	6	1	562	14	19809	3.32E-01
GO:0035925	mRNA 3'-UTR AU-rich regior	MF	9	1	562	14	19809	3.32E-01
GO:0010952	positive regulation of peptidas	BP	10	1	562	14	19809	3.32E-01
GO:0005095	GTPase inhibitor activity	MF	6	1	562	14	19809	3.32E-01
GO:0033162	melanosome membrane	CC	7	1	562	14	19809	3.32E-01
GO:0042574	retinal metabolic process	BP	11	1	562	14	19809	3.32E-01
GO:0009312	oligosaccharide biosynthetic p	BP	7	1	562	14	19809	3.32E-01
GO:0003085	negative regulation of systemic	BP	7	1	562	14	19809	3.32E-01
GO:0061157	mRNA destabilization	BP	11	1	562	14	19809	3.32E-01
GO:0002687	positive regulation of leukocyt	BP	7	1	562	14	19809	3.32E-01
GO:0031089	platelet dense granule lumen	CC	8	1	562	14	19809	3.32E-01
GO:0019841	retinol binding	MF	7	1	562	14	19809	3.32E-01
GO:0086064	cell communication by electric	BP	6	1	562	14	19809	3.32E-01
GO:0046039	GTP metabolic process	BP	11	1	562	14	19809	3.32E-01
GO:0007035	vacuolar acidification	BP	13	1	562	14	19809	3.32E-01
GO:0031936	negative regulation of chromat	BP	10	1	562	14	19809	3.32E-01
GO:0021954	central nervous system neuron	BP	7	1	562	14	19809	3.32E-01
GO:0031573	intra-S DNA damage checkpoi	BP	9	1	562	14	19809	3.32E-01
GO:1903351	cellular response to dopamine	BP	7	1	562	14	19809	3.32E-01
GO:0005243	gap junction channel activity	MF	8	1	562	14	19809	3.32E-01
GO:0006071	glycerol metabolic process	BP	8	1	562	14	19809	3.32E-01
GO:0031432	titin binding	MF	6	1	562	14	19809	3.32E-01
GO:0060402	calcium ion transport into cyto	BP	14	1	562	14	19809	3.32E-01
GO:0051279	regulation of release of seques	BP	7	1	562	14	19809	3.32E-01
GO:0050673	epithelial cell proliferation	BP	4	1	562	14	19809	3.32E-01
GO:0016772	transferase activity, transferrin	MF	5	1	562	14	19809	3.32E-01
GO:0048009	insulin-like growth factor rece	BP	8	1	562	14	19809	3.32E-01
GO:0005003	ephrin receptor activity	MF	10	1	562	14	19809	3.32E-01
GO:0060038	cardiac muscle cell proliferatic	BP	6	1	562	14	19809	3.32E-01
GO:0010248	establishment or maintenance	BP	8	1	562	14	19809	3.32E-01

GO:0002504	antigen processing and present	BP	5	1	562	14	19809	3.32E-01
GO:0043249	erythrocyte maturation	BP	6	1	562	14	19809	3.32E-01
GO:0010759	positive regulation of macroph	BP	9	1	562	14	19809	3.32E-01
GO:0009435	NAD biosynthetic process	BP	10	1	562	14	19809	3.32E-01
GO:0010954	positive regulation of protein p	BP	10	1	562	14	19809	3.32E-01
GO:0016811	hydrolase activity, acting on ca	MF	6	1	562	14	19809	3.32E-01
GO:0071305	cellular response to vitamin D	BP	8	1	562	14	19809	3.32E-01
GO:0031669	cellular response to nutrient le	BP	7	1	562	14	19809	3.32E-01
GO:1990380	Lys48-specific deubiquitinase	MF	6	1	562	14	19809	3.32E-01
GO:0045651	positive regulation of macroph	BP	10	1	562	14	19809	3.32E-01
GO:0004029	aldehyde dehydrogenase (NAI	MF	7	1	562	14	19809	3.32E-01
GO:0038024	cargo receptor activity	MF	3	1	562	14	19809	3.32E-01
GO:0032693	negative regulation of interleul	BP	8	1	562	14	19809	3.32E-01
GO:0048172	regulation of short-term neuroı	BP	11	1	562	14	19809	3.32E-01
GO:0050892	intestinal absorption	BP	6	1	562	14	19809	3.32E-01
GO:0019725	cellular homeostasis	BP	4	1	562	14	19809	3.32E-01
GO:0015693	magnesium ion transport	BP	10	1	562	14	19809	3.32E-01
GO:0032743	positive regulation of interleuk	BP	8	1	562	14	19809	3.32E-01
GO:1903428	positive regulation of reactive	BP	8	1	562	14	19809	3.32E-01
GO:0015278	calcium-release channel activit	MF	9	1	562	14	19809	3.32E-01
GO:0016471	vacuolar proton-transporting V	CC	7	1	562	14	19809	3.32E-01
GO:1900747	negative regulation of vasculaı	BP	9	1	562	14	19809	3.32E-01
GO:0071805	potassium ion transmembrane	BP	10	5	562	134	19809	3.32E-01
GO:0002244	hematopoietic progenitor cell d	BP	6	3	562	72	19809	3.35E-01
GO:0051017	actin filament bundle assembly	BP	6	2	562	42	19809	3.35E-01
GO:0001102	RNA polymerase II activating	MF	8	2	562	42	19809	3.35E-01
GO:0090501	RNA phosphodiester bond hyd	BP	9	2	562	42	19809	3.35E-01
GO:0051225	spindle assembly	BP	9	2	562	42	19809	3.35E-01
GO:0010811	positive regulation of cell-subs	BP	7	2	562	42	19809	3.35E-01
GO:0019882	antigen processing and present	BP	4	2	562	42	19809	3.35E-01
GO:0005160	transforming growth factor bet	MF	7	2	562	42	19809	3.35E-01
GO:0098655	cation transmembrane transpoı	BP	8	2	562	42	19809	3.35E-01
GO:0007156	homophilic cell adhesion via p	BP	7	6	562	167	19809	3.37E-01
GO:0006355	regulation of transcription, DN	BP	8	47	562	1551	19809	3.39E-01
GO:0005516	calmodulin binding	MF	5	7	562	200	19809	3.40E-01
GO:0032091	negative regulation of protein l	BP	7	3	562	73	19809	3.43E-01
GO:0050767	regulation of neurogenesis	BP	8	2	562	43	19809	3.46E-01
GO:0006352	DNA-templated transcription,	BP	8	2	562	43	19809	3.46E-01
GO:0007368	determination of left/right sym	BP	7	3	562	74	19809	3.51E-01
GO:0043433	negative regulation of DNA-bi	BP	6	3	562	74	19809	3.51E-01
GO:0006878	cellular copper ion homeostasi	BP	12	1	562	15	19809	3.51E-01
GO:0016540	protein autoprocessing	BP	9	1	562	15	19809	3.51E-01
GO:0034587	piRNA metabolic process	BP	10	1	562	15	19809	3.51E-01
GO:1903830	magnesium ion transmembrane	BP	11	1	562	15	19809	3.51E-01
GO:0035909	aorta morphogenesis	BP	8	1	562	15	19809	3.51E-01
GO:0042554	superoxide anion generation	BP	7	1	562	15	19809	3.51E-01
GO:0005639	integral component of nuclear	CC	7	1	562	15	19809	3.51E-01
GO:0033689	negative regulation of osteobla	BP	8	1	562	15	19809	3.51E-01
GO:0051016	barbed-end actin filament capp	BP	11	1	562	15	19809	3.51E-01
GO:0032703	negative regulation of interleul	BP	8	1	562	15	19809	3.51E-01

GO:0099059	integral component of presyna	CC	7	1	562	15	19809	3.51E-01
GO:0031462	Cul2-RING ubiquitin ligase co	CC	7	1	562	15	19809	3.51E-01
GO:0045667	regulation of osteoblast differe	BP	7	1	562	15	19809	3.51E-01
GO:1900181	negative regulation of protein l	BP	9	1	562	15	19809	3.51E-01
GO:0016584	nucleosome positioning	BP	8	1	562	15	19809	3.51E-01
GO:0045178	basal part of cell	CC	4	1	562	15	19809	3.51E-01
GO:0030195	negative regulation of blood co	BP	8	1	562	15	19809	3.51E-01
GO:0060047	heart contraction	BP	7	1	562	15	19809	3.51E-01
GO:0045580	regulation of T cell differentia	BP	9	1	562	15	19809	3.51E-01
GO:0006518	peptide metabolic process	BP	7	1	562	15	19809	3.51E-01
GO:0031045	dense core granule	CC	9	1	562	15	19809	3.51E-01
GO:2000737	negative regulation of stem cel	BP	8	1	562	15	19809	3.51E-01
GO:0090303	positive regulation of wound h	BP	9	1	562	15	19809	3.51E-01
GO:0005161	platelet-derived growth factor	MF	7	1	562	15	19809	3.51E-01
GO:0043508	negative regulation of JUN kir	BP	13	1	562	15	19809	3.51E-01
GO:0042622	photoreceptor outer segment m	CC	7	1	562	15	19809	3.51E-01
GO:0035326	enhancer binding	MF	8	1	562	15	19809	3.51E-01
GO:0006112	energy reserve metabolic proc	BP	7	1	562	15	19809	3.51E-01
GO:0032516	positive regulation of phospho	BP	12	1	562	15	19809	3.51E-01
GO:1903206	negative regulation of hydroge	BP	10	1	562	15	19809	3.51E-01
GO:0032794	GTPase activating protein binc	MF	5	1	562	15	19809	3.51E-01
GO:1901381	positive regulation of potassiu	BP	11	1	562	15	19809	3.51E-01
GO:0050910	detection of mechanical stimul	BP	6	1	562	15	19809	3.51E-01
GO:0007158	neuron cell-cell adhesion	BP	6	1	562	15	19809	3.51E-01
GO:0015908	fatty acid transport	BP	8	1	562	15	19809	3.51E-01
GO:0006750	glutathione biosynthetic proce	BP	9	1	562	15	19809	3.51E-01
GO:0001510	RNA methylation	BP	10	1	562	15	19809	3.51E-01
GO:0008527	taste receptor activity	MF	6	1	562	15	19809	3.51E-01
GO:2000484	positive regulation of interleuk	BP	9	1	562	15	19809	3.51E-01
GO:0097623	potassium ion export across pl	BP	11	1	562	15	19809	3.51E-01
GO:0060996	dendritic spine development	BP	5	1	562	15	19809	3.51E-01
GO:0046935	1-phosphatidylinositol-3-kinas	MF	7	1	562	15	19809	3.51E-01
GO:0010820	positive regulation of T cell ch	BP	10	1	562	15	19809	3.51E-01
GO:0098993	anchored component of synapt	CC	6	1	562	15	19809	3.51E-01
GO:0043951	negative regulation of cAMP-r	BP	10	1	562	15	19809	3.51E-01
GO:0035259	glucocorticoid receptor bindin	MF	9	1	562	15	19809	3.51E-01
GO:0005088	Ras guanyl-nucleotide exchang	MF	8	1	562	15	19809	3.51E-01
GO:0045670	regulation of osteoclast differe	BP	9	1	562	15	19809	3.51E-01
GO:0043083	synaptic cleft	CC	4	1	562	15	19809	3.51E-01
GO:0003841	1-acylglycerol-3-phosphate O-	MF	9	1	562	15	19809	3.51E-01
GO:0006914	autophagy	BP	6	6	562	170	19809	3.52E-01
GO:0007417	central nervous system develo	BP	6	5	562	138	19809	3.54E-01
GO:0002224	toll-like receptor signaling patl	BP	8	2	562	44	19809	3.56E-01
GO:0070527	platelet aggregation	BP	7	2	562	44	19809	3.56E-01
GO:0030282	bone mineralization	BP	7	2	562	44	19809	3.56E-01
GO:0008307	structural constituent of muscl	MF	4	2	562	44	19809	3.56E-01
GO:0008652	cellular amino acid biosyntheti	BP	9	2	562	44	19809	3.56E-01
GO:0004857	enzyme inhibitor activity	MF	5	2	562	44	19809	3.56E-01
GO:0021762	substantia nigra development	BP	6	2	562	44	19809	3.56E-01
GO:0007266	Rho protein signal transductio	BP	8	3	562	75	19809	3.58E-01

GO:0016757	transferase activity, transferrin	MF	5	8	562	237	19809	3.59E-01
GO:0016787	hydrolase activity	MF	4	50	562	1667	19809	3.60E-01
GO:0030016	myofibril	CC	7	2	562	45	19809	3.66E-01
GO:0042562	hormone binding	MF	4	1	562	16	19809	3.69E-01
GO:0005005	transmembrane-ephrin recepto	MF	11	1	562	16	19809	3.69E-01
GO:0015671	oxygen transport	BP	7	1	562	16	19809	3.69E-01
GO:0010633	negative regulation of epithelia	BP	9	1	562	16	19809	3.69E-01
GO:0008328	ionotropic glutamate receptor c	CC	7	1	562	16	19809	3.69E-01
GO:0001516	prostaglandin biosynthetic pro	BP	11	1	562	16	19809	3.69E-01
GO:0014067	negative regulation of phospha	BP	10	1	562	16	19809	3.69E-01
GO:0097746	regulation of blood vessel diam	BP	8	1	562	16	19809	3.69E-01
GO:2001224	positive regulation of neuron m	BP	9	1	562	16	19809	3.69E-01
GO:0035162	embryonic hemopoiesis	BP	8	1	562	16	19809	3.69E-01
GO:0042974	retinoic acid receptor binding	MF	8	1	562	16	19809	3.69E-01
GO:0042613	MHC class II protein complex	CC	7	1	562	16	19809	3.69E-01
GO:0001736	establishment of planar polarit	BP	6	1	562	16	19809	3.69E-01
GO:0050868	negative regulation of T cell ac	BP	9	1	562	16	19809	3.69E-01
GO:0004708	MAP kinase kinase activity	MF	9	1	562	16	19809	3.69E-01
GO:0008066	glutamate receptor activity	MF	6	1	562	16	19809	3.69E-01
GO:2000406	positive regulation of T cell m	BP	9	1	562	16	19809	3.69E-01
GO:0061053	somite development	BP	7	1	562	16	19809	3.69E-01
GO:0048384	retinoic acid receptor signaling	BP	6	1	562	16	19809	3.69E-01
GO:0003756	protein disulfide isomerase act	MF	7	1	562	16	19809	3.69E-01
GO:0045616	regulation of keratinocyte diffe	BP	9	1	562	16	19809	3.69E-01
GO:0006790	sulfur compound metabolic pro	BP	5	1	562	16	19809	3.69E-01
GO:0032959	inositol trisphosphate biosynth	BP	9	1	562	16	19809	3.69E-01
GO:0000400	four-way junction DNA bindin	MF	8	1	562	16	19809	3.69E-01
GO:0015095	magnesium ion transmembrane	MF	9	1	562	16	19809	3.69E-01
GO:0035970	peptidyl-threonine dephosphor	BP	10	1	562	16	19809	3.69E-01
GO:0000028	ribosomal small subunit assem	BP	9	1	562	16	19809	3.69E-01
GO:0035640	exploration behavior	BP	4	1	562	16	19809	3.69E-01
GO:0019372	lipoxygenase pathway	BP	10	1	562	16	19809	3.69E-01
GO:0071391	cellular response to estrogen st	BP	5	1	562	16	19809	3.69E-01
GO:0035196	production of miRNAs involve	BP	12	1	562	16	19809	3.69E-01
GO:0086010	membrane depolarization durin	BP	7	1	562	16	19809	3.69E-01
GO:0006895	Golgi to endosome transport	BP	9	1	562	16	19809	3.69E-01
GO:0015813	L-glutamate transmembrane tra	BP	11	1	562	16	19809	3.69E-01
GO:0097320	plasma membrane tubulation	BP	7	1	562	16	19809	3.69E-01
GO:0032963	collagen metabolic process	BP	4	1	562	16	19809	3.69E-01
GO:0008656	cysteine-type endopeptidase ac	MF	8	1	562	16	19809	3.69E-01
GO:0043395	heparan sulfate proteoglycan b	MF	6	1	562	16	19809	3.69E-01
GO:0006941	striated muscle contraction	BP	7	1	562	16	19809	3.69E-01
GO:0003382	epithelial cell morphogenesis	BP	8	1	562	16	19809	3.69E-01
GO:0050921	positive regulation of chemota	BP	8	1	562	16	19809	3.69E-01
GO:2001222	regulation of neuron migration	BP	8	1	562	16	19809	3.69E-01
GO:0005662	DNA replication factor A com	CC	4	1	562	16	19809	3.69E-01
GO:0061158	3'-UTR-mediated mRNA desta	BP	12	1	562	16	19809	3.69E-01
GO:0003222	ventricular trabecula myocardi	BP	9	1	562	16	19809	3.69E-01
GO:0071850	mitotic cell cycle arrest	BP	6	1	562	16	19809	3.69E-01
GO:0060216	definitive hemopoiesis	BP	8	1	562	16	19809	3.69E-01

GO:0030021	extracellular matrix structural	MF	5	1	562	16	19809	3.69E-01
GO:0090280	positive regulation of calcium	BP	10	1	562	16	19809	3.69E-01
GO:0001935	endothelial cell proliferation	BP	5	1	562	16	19809	3.69E-01
GO:0034975	protein folding in endoplasmic	BP	5	1	562	16	19809	3.69E-01
GO:0070593	dendrite self-avoidance	BP	6	1	562	16	19809	3.69E-01
GO:0045589	regulation of regulatory T cell	BP	10	1	562	16	19809	3.69E-01
GO:0045190	isotype switching	BP	8	1	562	16	19809	3.69E-01
GO:0035102	PRC1 complex	CC	7	1	562	16	19809	3.69E-01
GO:0045202	synapse	CC	3	19	562	610	19809	3.71E-01
GO:0042383	sarcolemma	CC	5	4	562	109	19809	3.74E-01
GO:0016363	nuclear matrix	CC	6	4	562	109	19809	3.74E-01
GO:0008584	male gonad development	BP	7	4	562	109	19809	3.74E-01
GO:0052689	carboxylic ester hydrolase acti	MF	6	2	562	46	19809	3.77E-01
GO:0007595	lactation	BP	8	2	562	46	19809	3.77E-01
GO:0010508	positive regulation of autophag	BP	9	2	562	46	19809	3.77E-01
GO:0050766	positive regulation of phagocy	BP	8	2	562	46	19809	3.77E-01
GO:0032993	protein-DNA complex	CC	4	2	562	46	19809	3.77E-01
GO:0030968	endoplasmic reticulum unfolde	BP	5	2	562	46	19809	3.77E-01
GO:0016311	dephosphorylation	BP	7	6	562	175	19809	3.78E-01
GO:0000786	nucleosome	CC	5	4	562	110	19809	3.80E-01
GO:0006334	nucleosome assembly	BP	7	4	562	110	19809	3.80E-01
GO:0005793	endoplasmic reticulum-Golgi i	CC	6	3	562	78	19809	3.82E-01
GO:0016197	endosomal transport	BP	7	3	562	78	19809	3.82E-01
GO:0031594	neuromuscular junction	CC	4	3	562	78	19809	3.82E-01
GO:0045454	cell redox homeostasis	BP	5	3	562	78	19809	3.82E-01
GO:0031965	nuclear membrane	CC	5	8	562	243	19809	3.85E-01
GO:0043647	inositol phosphate metabolic p	BP	7	2	562	47	19809	3.87E-01
GO:0030155	regulation of cell adhesion	BP	5	2	562	47	19809	3.87E-01
GO:0014704	intercalated disc	CC	6	2	562	47	19809	3.87E-01
GO:0035914	skeletal muscle cell differentia	BP	6	2	562	47	19809	3.87E-01
GO:0032270	positive regulation of cellular	BP	8	1	562	17	19809	3.87E-01
GO:0060285	cilium-dependent cell motility	BP	7	1	562	17	19809	3.87E-01
GO:0030247	polysaccharide binding	MF	5	1	562	17	19809	3.87E-01
GO:0042813	Wnt-activated receptor activity	MF	6	1	562	17	19809	3.87E-01
GO:0006883	cellular sodium ion homeostas	BP	11	1	562	17	19809	3.87E-01
GO:0043292	contractile fiber	CC	6	1	562	17	19809	3.87E-01
GO:0051131	chaperone-mediated protein co	BP	8	1	562	17	19809	3.87E-01
GO:0031143	pseudopodium	CC	6	1	562	17	19809	3.87E-01
GO:0008324	cation transmembrane transpo	MF	6	1	562	17	19809	3.87E-01
GO:1904355	positive regulation of telomere	BP	11	1	562	17	19809	3.87E-01
GO:0002407	dendritic cell chemotaxis	BP	6	1	562	17	19809	3.87E-01
GO:0034122	negative regulation of toll-like	BP	7	1	562	17	19809	3.87E-01
GO:0046849	bone remodeling	BP	5	1	562	17	19809	3.87E-01
GO:0070063	RNA polymerase binding	MF	6	1	562	17	19809	3.87E-01
GO:0031904	endosome lumen	CC	8	1	562	17	19809	3.87E-01
GO:0060324	face development	BP	5	1	562	17	19809	3.87E-01
GO:0042834	peptidoglycan binding	MF	6	1	562	17	19809	3.87E-01
GO:0098688	parallel fiber to Purkinje cell s	CC	5	1	562	17	19809	3.87E-01
GO:0043024	ribosomal small subunit bindir	MF	6	1	562	17	19809	3.87E-01
GO:0047555	3',5'-cyclic-GMP phosphodiest	MF	10	1	562	17	19809	3.87E-01

GO:0035036	sperm-egg recognition	BP	6	1	562	17	19809	3.87E-01
GO:0032966	negative regulation of collager	BP	8	1	562	17	19809	3.87E-01
GO:0033198	response to ATP	BP	8	1	562	17	19809	3.87E-01
GO:0055075	potassium ion homeostasis	BP	10	1	562	17	19809	3.87E-01
GO:0048535	lymph node development	BP	7	1	562	17	19809	3.87E-01
GO:0045717	negative regulation of fatty aci	BP	9	1	562	17	19809	3.87E-01
GO:0004198	calcium-dependent cysteine-ty	MF	9	1	562	17	19809	3.87E-01
GO:0010586	miRNA metabolic process	BP	10	1	562	17	19809	3.87E-01
GO:0042310	vasoconstriction	BP	10	1	562	17	19809	3.87E-01
GO:0034375	high-density lipoprotein partic	BP	9	1	562	17	19809	3.87E-01
GO:1901653	cellular response to peptide	BP	8	1	562	17	19809	3.87E-01
GO:0031489	myosin V binding	MF	7	1	562	17	19809	3.87E-01
GO:0050850	positive regulation of calcium-	BP	10	1	562	17	19809	3.87E-01
GO:0030837	negative regulation of actin fil	BP	9	1	562	17	19809	3.87E-01
GO:0060441	epithelial tube branching invol	BP	7	1	562	17	19809	3.87E-01
GO:0070886	positive regulation of calcineu	BP	12	1	562	17	19809	3.87E-01
GO:0007009	plasma membrane organizatioı	BP	6	1	562	17	19809	3.87E-01
GO:0051216	cartilage development	BP	6	3	562	79	19809	3.89E-01
GO:0001933	negative regulation of protein	BP	10	3	562	79	19809	3.89E-01
GO:0040008	regulation of growth	BP	5	3	562	79	19809	3.89E-01
GO:0006813	potassium ion transport	BP	9	5	562	145	19809	3.94E-01
GO:0060291	long-term synaptic potentiatioı	BP	10	2	562	48	19809	3.97E-01
GO:0000186	activation of MAPKK activity	BP	13	2	562	48	19809	3.97E-01
GO:0006520	cellular amino acid metabolic	BP	8	2	562	48	19809	3.97E-01
GO:0007157	heterophilic cell-cell adhesion	BP	7	2	562	48	19809	3.97E-01
GO:0048015	phosphatidylinositol-mediated	BP	7	2	562	48	19809	3.97E-01
GO:0050808	synapse organization	BP	5	2	562	48	19809	3.97E-01
GO:0051117	ATPase binding	MF	6	3	562	80	19809	3.97E-01
GO:0043123	positive regulation of I-kappaE	BP	10	6	562	179	19809	3.98E-01
GO:0008289	lipid binding	MF	4	10	562	314	19809	4.00E-01
GO:0045211	postsynaptic membrane	CC	5	8	562	247	19809	4.02E-01
GO:0005525	GTP binding	MF	8	12	562	383	19809	4.04E-01
GO:0019432	triglyceride biosynthetic proce	BP	10	1	562	18	19809	4.04E-01
GO:0001709	cell fate determination	BP	5	1	562	18	19809	4.04E-01
GO:0001702	gastrulation with mouth formiı	BP	7	1	562	18	19809	4.04E-01
GO:0000712	resolution of meiotic recombin	BP	7	1	562	18	19809	4.04E-01
GO:0043011	myeloid dendritic cell differen	BP	8	1	562	18	19809	4.04E-01
GO:0048566	embryonic digestive tract deve	BP	7	1	562	18	19809	4.04E-01
GO:0090026	positive regulation of monocyt	BP	9	1	562	18	19809	4.04E-01
GO:0046847	filopodium assembly	BP	8	1	562	18	19809	4.04E-01
GO:0048646	anatomical structure formation	BP	4	1	562	18	19809	4.04E-01
GO:1901379	regulation of potassium ion tra	BP	10	1	562	18	19809	4.04E-01
GO:0007095	mitotic G2 DNA damage checl	BP	9	1	562	18	19809	4.04E-01
GO:0042288	MHC class I protein binding	MF	7	1	562	18	19809	4.04E-01
GO:0009190	cyclic nucleotide biosynthetic	BP	10	1	562	18	19809	4.04E-01
GO:0032433	filopodium tip	CC	6	1	562	18	19809	4.04E-01
GO:0097602	cullin family protein binding	MF	5	1	562	18	19809	4.04E-01
GO:0004970	ionotropic glutamate receptor a	MF	7	1	562	18	19809	4.04E-01
GO:0032331	negative regulation of chondro	BP	8	1	562	18	19809	4.04E-01
GO:0008301	DNA binding, bending	MF	7	1	562	18	19809	4.04E-01

GO:0022011	myelination in peripheral nerv	BP	7	1	562	18	19809	4.04E-01
GO:0099524	postsynaptic cytosol	CC	4	1	562	18	19809	4.04E-01
GO:0010666	positive regulation of cardiac r	BP	12	1	562	18	19809	4.04E-01
GO:0051895	negative regulation of focal ad	BP	9	1	562	18	19809	4.04E-01
GO:0008569	ATP-dependent microtubule m	MF	12	1	562	18	19809	4.04E-01
GO:0070064	proline-rich region binding	MF	5	1	562	18	19809	4.04E-01
GO:0007250	activation of NF-kappaB-induc	BP	13	1	562	18	19809	4.04E-01
GO:2000042	negative regulation of double-s	BP	11	1	562	18	19809	4.04E-01
GO:2000251	positive regulation of actin cyt	BP	10	1	562	18	19809	4.04E-01
GO:0080111	DNA demethylation	BP	10	1	562	18	19809	4.04E-01
GO:0001964	startle response	BP	5	1	562	18	19809	4.04E-01
GO:0000038	very long-chain fatty acid meta	BP	10	1	562	18	19809	4.04E-01
GO:0060037	pharyngeal system developmen	BP	6	1	562	18	19809	4.04E-01
GO:0019752	carboxylic acid metabolic proc	BP	7	1	562	18	19809	4.04E-01
GO:0032426	stereocilium tip	CC	4	1	562	18	19809	4.04E-01
GO:0007221	positive regulation of transcrip	BP	11	1	562	18	19809	4.04E-01
GO:0050880	regulation of blood vessel size	BP	7	1	562	18	19809	4.04E-01
GO:0008320	protein transmembrane transpo	MF	6	1	562	18	19809	4.04E-01
GO:0003429	growth plate cartilage chondro	BP	10	1	562	18	19809	4.04E-01
GO:0043551	regulation of phosphatidylinos	BP	9	1	562	18	19809	4.04E-01
GO:0007194	negative regulation of adenylat	BP	8	1	562	18	19809	4.04E-01
GO:0086005	ventricular cardiac muscle cell	BP	9	1	562	18	19809	4.04E-01
GO:0034199	activation of protein kinase A a	BP	13	1	562	18	19809	4.04E-01
GO:1990126	retrograde transport, endosome	BP	8	1	562	18	19809	4.04E-01
GO:0035269	protein O-linked mannosylatio	BP	11	1	562	18	19809	4.04E-01
GO:0005798	Golgi-associated vesicle	CC	7	1	562	18	19809	4.04E-01
GO:0061178	regulation of insulin secretion	BP	6	1	562	18	19809	4.04E-01
GO:0038191	neuropilin binding	MF	6	1	562	18	19809	4.04E-01
GO:0006936	muscle contraction	BP	6	4	562	114	19809	4.06E-01
GO:0005179	hormone activity	MF	7	4	562	114	19809	4.06E-01
GO:0034260	negative regulation of GTPase	BP	8	2	562	49	19809	4.07E-01
GO:1990841	promoter-specific chromatin b	MF	5	2	562	49	19809	4.07E-01
GO:0023014	signal transduction by protein	BP	10	2	562	49	19809	4.07E-01
GO:0016491	oxidoreductase activity	MF	4	18	562	589	19809	4.07E-01
GO:0060271	cilium assembly	BP	7	6	562	181	19809	4.08E-01
GO:0001764	neuron migration	BP	7	4	562	115	19809	4.12E-01
GO:0009887	animal organ morphogenesis	BP	5	4	562	115	19809	4.12E-01
GO:0046983	protein dimerization activity	MF	5	7	562	216	19809	4.15E-01
GO:0030514	negative regulation of BMP sig	BP	11	2	562	50	19809	4.17E-01
GO:0007173	epidermal growth factor recept	BP	9	2	562	50	19809	4.17E-01
GO:0016301	kinase activity	MF	6	21	562	696	19809	4.18E-01
GO:0000724	double-strand break repair via	BP	10	3	562	83	19809	4.20E-01
GO:0045088	regulation of innate immune re	BP	7	1	562	19	19809	4.21E-01
GO:0001223	transcription coactivator bindi	MF	7	1	562	19	19809	4.21E-01
GO:2000649	regulation of sodium ion trans	BP	8	1	562	19	19809	4.21E-01
GO:0010614	negative regulation of cardiac	BP	10	1	562	19	19809	4.21E-01
GO:2000774	positive regulation of cellular s	BP	7	1	562	19	19809	4.21E-01
GO:0098641	cadherin binding involved in c	MF	7	1	562	19	19809	4.21E-01
GO:0000146	microfilament motor activity	MF	10	1	562	19	19809	4.21E-01
GO:0006937	regulation of muscle contractio	BP	8	1	562	19	19809	4.21E-01

GO:1902236	negative regulation of endopla	BP	9	1	562	19	19809	4.21E-01
GO:0090201	negative regulation of release (	BP	10	1	562	19	19809	4.21E-01
GO:0035338	long-chain fatty-acyl-CoA bios	BP	10	1	562	19	19809	4.21E-01
GO:0046655	folic acid metabolic process	BP	7	1	562	19	19809	4.21E-01
GO:0048265	response to pain	BP	6	1	562	19	19809	4.21E-01
GO:1900017	positive regulation of cytokine	BP	8	1	562	19	19809	4.21E-01
GO:0071354	cellular response to interleukin	BP	8	1	562	19	19809	4.21E-01
GO:0045582	positive regulation of T cell di	BP	10	1	562	19	19809	4.21E-01
GO:0060546	negative regulation of necropto	BP	10	1	562	19	19809	4.21E-01
GO:0048821	erythrocyte development	BP	7	1	562	19	19809	4.21E-01
GO:0070971	endoplasmic reticulum exit site	CC	7	1	562	19	19809	4.21E-01
GO:0006939	smooth muscle contraction	BP	7	1	562	19	19809	4.21E-01
GO:0071397	cellular response to cholestero	BP	9	1	562	19	19809	4.21E-01
GO:0090190	positive regulation of branchin	BP	10	1	562	19	19809	4.21E-01
GO:0030539	male genitalia development	BP	7	1	562	19	19809	4.21E-01
GO:0006998	nuclear envelope organization	BP	6	1	562	19	19809	4.21E-01
GO:0043034	costamere	CC	5	1	562	19	19809	4.21E-01
GO:0042311	vasodilation	BP	10	1	562	19	19809	4.21E-01
GO:0030279	negative regulation of ossificat	BP	7	1	562	19	19809	4.21E-01
GO:0022904	respiratory electron transport c	BP	7	1	562	19	19809	4.21E-01
GO:0070932	histone H3 deacetylation	BP	12	1	562	19	19809	4.21E-01
GO:0030220	platelet formation	BP	7	1	562	19	19809	4.21E-01
GO:0034314	Arp2/3 complex-mediated acti	BP	8	1	562	19	19809	4.21E-01
GO:0010812	negative regulation of cell-sub	BP	7	1	562	19	19809	4.21E-01
GO:0046597	negative regulation of viral ent	BP	10	1	562	19	19809	4.21E-01
GO:0031290	retinal ganglion cell axon guid	BP	7	1	562	19	19809	4.21E-01
GO:0006337	nucleosome disassembly	BP	8	1	562	19	19809	4.21E-01
GO:0008284	positive regulation of cell popu	BP	7	17	562	561	19809	4.25E-01
GO:0009755	hormone-mediated signaling p	BP	5	2	562	51	19809	4.27E-01
GO:0004402	histone acetyltransferase activi	MF	11	2	562	51	19809	4.27E-01
GO:0048146	positive regulation of fibroblas	BP	8	2	562	51	19809	4.27E-01
GO:0006091	generation of precursor metabo	BP	5	2	562	51	19809	4.27E-01
GO:0008047	enzyme activator activity	MF	5	2	562	51	19809	4.27E-01
GO:0045785	positive regulation of cell adhe	BP	6	2	562	51	19809	4.27E-01
GO:0008013	beta-catenin binding	MF	5	3	562	84	19809	4.28E-01
GO:0000976	transcription regulatory region	MF	8	3	562	84	19809	4.28E-01
GO:0042995	cell projection	CC	4	29	562	978	19809	4.30E-01
GO:0000978	RNA polymerase II proximal p	MF	10	15	562	494	19809	4.31E-01
GO:0004713	protein tyrosine kinase activity	MF	8	4	562	118	19809	4.31E-01
GO:0005737	cytoplasm	CC	5	189	562	6581	19809	4.34E-01
GO:0098685	Schaffer collateral - CA1 syna	CC	4	3	562	85	19809	4.35E-01
GO:0034644	cellular response to UV	BP	8	2	562	52	19809	4.36E-01
GO:0007269	neurotransmitter secretion	BP	6	2	562	52	19809	4.36E-01
GO:0030148	sphingolipid biosynthetic proc	BP	9	2	562	52	19809	4.36E-01
GO:0010942	positive regulation of cell deat	BP	7	2	562	52	19809	4.36E-01
GO:0040037	negative regulation of fibrobla	BP	9	1	562	20	19809	4.38E-01
GO:0032722	positive regulation of chemoki	BP	8	1	562	20	19809	4.38E-01
GO:0048011	neurotrophin TRK receptor sig	BP	8	1	562	20	19809	4.38E-01
GO:0001502	cartilage condensation	BP	4	1	562	20	19809	4.38E-01
GO:0060716	labyrinthine layer blood vessel	BP	7	1	562	20	19809	4.38E-01

GO:0001774	microglial cell activation	BP	7	1	562	20	19809	4.38E-01
GO:0045197	establishment or maintenance (	BP	7	1	562	20	19809	4.38E-01
GO:0045019	negative regulation of nitric ox	BP	9	1	562	20	19809	4.38E-01
GO:0007413	axonal fasciculation	BP	6	1	562	20	19809	4.38E-01
GO:0045954	positive regulation of natural k	BP	8	1	562	20	19809	4.38E-01
GO:0016849	phosphorus-oxygen lyase activ	MF	5	1	562	20	19809	4.38E-01
GO:0002028	regulation of sodium ion transp	BP	9	1	562	20	19809	4.38E-01
GO:0048168	regulation of neuronal synaptic	BP	10	1	562	20	19809	4.38E-01
GO:0070542	response to fatty acid	BP	6	1	562	20	19809	4.38E-01
GO:0032006	regulation of TOR signaling	BP	9	1	562	20	19809	4.38E-01
GO:0000062	fatty-acyl-CoA binding	MF	9	1	562	20	19809	4.38E-01
GO:0006536	glutamate metabolic process	BP	11	1	562	20	19809	4.38E-01
GO:0006826	iron ion transport	BP	10	1	562	20	19809	4.38E-01
GO:0042474	middle ear morphogenesis	BP	6	1	562	20	19809	4.38E-01
GO:0098632	cell-cell adhesion mediator act	MF	5	1	562	20	19809	4.38E-01
GO:0004806	triglyceride lipase activity	MF	7	1	562	20	19809	4.38E-01
GO:0003678	DNA helicase activity	MF	10	1	562	20	19809	4.38E-01
GO:0030048	actin filament-based movemen	BP	5	1	562	20	19809	4.38E-01
GO:0010592	positive regulation of lamellip	BP	11	1	562	20	19809	4.38E-01
GO:0043046	DNA methylation involved in	BP	11	1	562	20	19809	4.38E-01
GO:0016234	inclusion body	CC	5	1	562	20	19809	4.38E-01
GO:0030517	negative regulation of axon ex	BP	8	1	562	20	19809	4.38E-01
GO:0042623	ATPase activity, coupled	MF	10	1	562	20	19809	4.38E-01
GO:0042832	defense response to protozoan	BP	8	1	562	20	19809	4.38E-01
GO:0009083	branched-chain amino acid cat	BP	10	1	562	20	19809	4.38E-01
GO:0032211	negative regulation of telomere	BP	12	1	562	20	19809	4.38E-01
GO:1901214	regulation of neuron death	BP	7	1	562	20	19809	4.38E-01
GO:0008408	3'-5' exonuclease activity	MF	8	1	562	20	19809	4.38E-01
GO:0031641	regulation of myelination	BP	6	1	562	20	19809	4.38E-01
GO:0005868	cytoplasmic dynein complex	CC	6	1	562	20	19809	4.38E-01
GO:0034361	very-low-density lipoprotein p	CC	6	1	562	20	19809	4.38E-01
GO:0016922	nuclear receptor binding	MF	8	1	562	20	19809	4.38E-01
GO:0035050	embryonic heart tube developn	BP	6	1	562	20	19809	4.38E-01
GO:0032088	negative regulation of NF-kap	BP	7	3	562	86	19809	4.43E-01
GO:0000226	microtubule cytoskeleton orga	BP	7	4	562	120	19809	4.44E-01
GO:0007254	JNK cascade	BP	13	2	562	53	19809	4.46E-01
GO:0043087	regulation of GTPase activity	BP	7	3	562	87	19809	4.50E-01
GO:0005720	nuclear heterochromatin	CC	8	1	562	21	19809	4.54E-01
GO:0071375	cellular response to peptide ho	BP	7	1	562	21	19809	4.54E-01
GO:0060425	lung morphogenesis	BP	6	1	562	21	19809	4.54E-01
GO:0035458	cellular response to interferon-	BP	8	1	562	21	19809	4.54E-01
GO:0070530	K63-linked polyubiquitin mod	MF	7	1	562	21	19809	4.54E-01
GO:0045742	positive regulation of epiderma	BP	10	1	562	21	19809	4.54E-01
GO:0044306	neuron projection terminus	CC	5	1	562	21	19809	4.54E-01
GO:0033141	positive regulation of peptidyl-	BP	12	1	562	21	19809	4.54E-01
GO:0030904	retromer complex	CC	4	1	562	21	19809	4.54E-01
GO:0045055	regulated exocytosis	BP	8	1	562	21	19809	4.54E-01
GO:0045182	translation regulator activity	MF	3	1	562	21	19809	4.54E-01
GO:0060044	negative regulation of cardiac	BP	8	1	562	21	19809	4.54E-01
GO:0002042	cell migration involved in spro	BP	11	1	562	21	19809	4.54E-01

GO:0016597	amino acid binding	MF	7	1	562	21	19809	4.54E-01
GO:0071404	cellular response to low-densit	BP	6	1	562	21	19809	4.54E-01
GO:0007276	gamete generation	BP	5	1	562	21	19809	4.54E-01
GO:0006801	superoxide metabolic process	BP	6	1	562	21	19809	4.54E-01
GO:0070584	mitochondrion morphogenesis	BP	7	1	562	21	19809	4.54E-01
GO:0034067	protein localization to Golgi ap	BP	8	1	562	21	19809	4.54E-01
GO:0035024	negative regulation of Rho pro	BP	12	1	562	21	19809	4.54E-01
GO:0017015	regulation of transforming gro	BP	9	1	562	21	19809	4.54E-01
GO:0001659	temperature homeostasis	BP	5	1	562	21	19809	4.54E-01
GO:0009235	cobalamin metabolic process	BP	7	1	562	21	19809	4.54E-01
GO:0005942	phosphatidylinositol 3-kinase c	CC	7	1	562	21	19809	4.54E-01
GO:0006796	phosphate-containing compou	BP	6	1	562	21	19809	4.54E-01
GO:0021522	spinal cord motor neuron diffe	BP	7	1	562	21	19809	4.54E-01
GO:0016601	Rac protein signal transductio	BP	8	1	562	21	19809	4.54E-01
GO:0045672	positive regulation of osteocla	BP	10	1	562	21	19809	4.54E-01
GO:0031410	cytoplasmic vesicle	CC	6	21	562	710	19809	4.54E-01
GO:0008344	adult locomotory behavior	BP	5	2	562	54	19809	4.56E-01
GO:0005070	SH3/SH2 adaptor activity	MF	7	2	562	54	19809	4.56E-01
GO:0001085	RNA polymerase II transcripti	MF	6	2	562	54	19809	4.56E-01
GO:1903779	regulation of cardiac conducti	BP	9	2	562	54	19809	4.56E-01
GO:0006468	protein phosphorylation	BP	9	19	562	642	19809	4.58E-01
GO:0008022	protein C-terminus binding	MF	5	6	562	191	19809	4.58E-01
GO:0016829	lyase activity	MF	4	5	562	157	19809	4.61E-01
GO:0012507	ER to Golgi transport vesicle r	CC	8	2	562	55	19809	4.65E-01
GO:0035176	social behavior	BP	5	2	562	55	19809	4.65E-01
GO:0042552	myelination	BP	6	2	562	55	19809	4.65E-01
GO:0016573	histone acetylation	BP	10	2	562	55	19809	4.65E-01
GO:0031267	small GTPase binding	MF	7	1	562	22	19809	4.69E-01
GO:0017075	syntaxin-1 binding	MF	7	1	562	22	19809	4.69E-01
GO:0002052	positive regulation of neurobla	BP	10	1	562	22	19809	4.69E-01
GO:0045216	cell-cell junction organization	BP	6	1	562	22	19809	4.69E-01
GO:0030325	adrenal gland development	BP	7	1	562	22	19809	4.69E-01
GO:0045663	positive regulation of myoblas	BP	8	1	562	22	19809	4.69E-01
GO:0051899	membrane depolarization	BP	6	1	562	22	19809	4.69E-01
GO:0001894	tissue homeostasis	BP	7	1	562	22	19809	4.69E-01
GO:1900016	negative regulation of cytokin	BP	8	1	562	22	19809	4.69E-01
GO:1900273	positive regulation of long-ter	BP	9	1	562	22	19809	4.69E-01
GO:0006541	glutamine metabolic process	BP	11	1	562	22	19809	4.69E-01
GO:0043548	phosphatidylinositol 3-kinase l	MF	5	1	562	22	19809	4.69E-01
GO:0097150	neuronal stem cell population	BP	5	1	562	22	19809	4.69E-01
GO:1903861	positive regulation of dendrite	BP	12	1	562	22	19809	4.69E-01
GO:0001953	negative regulation of cell-mat	BP	8	1	562	22	19809	4.69E-01
GO:0042629	mast cell granule	CC	9	1	562	22	19809	4.69E-01
GO:0050321	tau-protein kinase activity	MF	8	1	562	22	19809	4.69E-01
GO:0005112	Notch binding	MF	6	1	562	22	19809	4.69E-01
GO:0014066	regulation of phosphatidylinos	BP	9	1	562	22	19809	4.69E-01
GO:1990138	neuron projection extension	BP	6	1	562	22	19809	4.69E-01
GO:0005158	insulin receptor binding	MF	6	1	562	22	19809	4.69E-01
GO:0016279	protein-lysine N-methyltransfe	MF	8	1	562	22	19809	4.69E-01
GO:0030215	semaphorin receptor binding	MF	6	1	562	22	19809	4.69E-01

GO:0032588	trans-Golgi network membrane	CC	8	3	562	90	19809	4.72E-01
GO:0048468	cell development	BP	5	2	562	56	19809	4.74E-01
GO:0032508	DNA duplex unwinding	BP	9	2	562	56	19809	4.74E-01
GO:0008081	phosphoric diester hydrolase a	MF	7	2	562	56	19809	4.74E-01
GO:0007623	circadian rhythm	BP	4	3	562	91	19809	4.79E-01
GO:0017124	SH3 domain binding	MF	6	4	562	126	19809	4.82E-01
GO:0031047	gene silencing by RNA	BP	5	2	562	57	19809	4.84E-01
GO:0060348	bone development	BP	6	2	562	57	19809	4.84E-01
GO:0017053	transcriptional repressor comp	CC	4	2	562	57	19809	4.84E-01
GO:0006865	amino acid transport	BP	10	2	562	57	19809	4.84E-01
GO:0009792	embryo development ending in	BP	6	1	562	23	19809	4.84E-01
GO:0030687	preribosome, large subunit pre	CC	6	1	562	23	19809	4.84E-01
GO:0060122	inner ear receptor cell stereoci	BP	8	1	562	23	19809	4.84E-01
GO:0001676	long-chain fatty acid metabolic	BP	10	1	562	23	19809	4.84E-01
GO:0005680	anaphase-promoting complex	CC	7	1	562	23	19809	4.84E-01
GO:0035904	aorta development	BP	7	1	562	23	19809	4.84E-01
GO:0030500	regulation of bone mineralizati	BP	7	1	562	23	19809	4.84E-01
GO:0035235	ionotropic glutamate receptor s	BP	7	1	562	23	19809	4.84E-01
GO:0060307	regulation of ventricular cardia	BP	11	1	562	23	19809	4.84E-01
GO:0090023	positive regulation of neutroph	BP	10	1	562	23	19809	4.84E-01
GO:0008198	ferrous iron binding	MF	9	1	562	23	19809	4.84E-01
GO:0031954	positive regulation of protein a	BP	11	1	562	23	19809	4.84E-01
GO:0097228	sperm principal piece	CC	5	1	562	23	19809	4.84E-01
GO:0017134	fibroblast growth factor bindin	MF	6	1	562	23	19809	4.84E-01
GO:0045104	intermediate filament cytoskel	BP	7	1	562	23	19809	4.84E-01
GO:0033344	cholesterol efflux	BP	10	1	562	23	19809	4.84E-01
GO:0046961	proton-transporting ATPase ac	MF	10	1	562	23	19809	4.84E-01
GO:0009880	embryonic pattern specificatio	BP	5	1	562	23	19809	4.84E-01
GO:0005548	phospholipid transporter activi	MF	5	1	562	23	19809	4.84E-01
GO:0001824	blastocyst development	BP	5	1	562	23	19809	4.84E-01
GO:0006672	ceramide metabolic process	BP	9	1	562	23	19809	4.84E-01
GO:1905564	positive regulation of vascular	BP	10	1	562	23	19809	4.84E-01
GO:0070412	R-SMAD binding	MF	6	1	562	23	19809	4.84E-01
GO:0097225	sperm midpiece	CC	5	1	562	23	19809	4.84E-01
GO:0044389	ubiquitin-like protein ligase bi	MF	6	1	562	23	19809	4.84E-01
GO:0006457	protein folding	BP	4	5	562	162	19809	4.88E-01
GO:0006629	lipid metabolic process	BP	5	16	562	548	19809	4.89E-01
GO:0006357	regulation of transcription by I	BP	9	36	562	1252	19809	4.92E-01
GO:0019827	stem cell population maintenan	BP	4	2	562	58	19809	4.93E-01
GO:0005507	copper ion binding	MF	8	2	562	58	19809	4.93E-01
GO:0055114	oxidation-reduction process	BP	4	21	562	725	19809	4.93E-01
GO:0032587	ruffle membrane	CC	6	3	562	93	19809	4.94E-01
GO:0010008	endosome membrane	CC	8	7	562	234	19809	4.97E-01
GO:2000378	negative regulation of reactive	BP	8	1	562	24	19809	4.99E-01
GO:0008585	female gonad development	BP	7	1	562	24	19809	4.99E-01
GO:0030595	leukocyte chemotaxis	BP	5	1	562	24	19809	4.99E-01
GO:0034383	low-density lipoprotein particl	BP	5	1	562	24	19809	4.99E-01
GO:0012501	programmed cell death	BP	5	1	562	24	19809	4.99E-01
GO:0034364	high-density lipoprotein partic	CC	5	1	562	24	19809	4.99E-01
GO:0060999	positive regulation of dendritic	BP	13	1	562	24	19809	4.99E-01

GO:0090398	cellular senescence	BP	6	1	562	24	19809	4.99E-01
GO:0010881	regulation of cardiac muscle co	BP	9	1	562	24	19809	4.99E-01
GO:0032733	positive regulation of interleuk	BP	8	1	562	24	19809	4.99E-01
GO:0003009	skeletal muscle contraction	BP	8	1	562	24	19809	4.99E-01
GO:0016831	carboxy-lyase activity	MF	6	1	562	24	19809	4.99E-01
GO:0015012	heparan sulfate proteoglycan b	BP	10	1	562	24	19809	4.99E-01
GO:0035567	non-canonical Wnt signaling p	BP	8	1	562	24	19809	4.99E-01
GO:0099560	synaptic membrane adhesion	BP	7	1	562	24	19809	4.99E-01
GO:0045453	bone resorption	BP	8	1	562	24	19809	4.99E-01
GO:0045662	negative regulation of myoblas	BP	8	1	562	24	19809	4.99E-01
GO:0048814	regulation of dendrite morpho;	BP	9	1	562	24	19809	4.99E-01
GO:0009954	proximal/distal pattern formati	BP	6	1	562	24	19809	4.99E-01
GO:0007626	locomotory behavior	BP	4	3	562	94	19809	5.01E-01
GO:0001725	stress fiber	CC	7	2	562	59	19809	5.02E-01
GO:0015297	antiporter activity	MF	7	2	562	59	19809	5.02E-01
GO:0035861	site of double-strand break	CC	7	2	562	59	19809	5.02E-01
GO:0007409	axonogenesis	BP	9	3	562	95	19809	5.08E-01
GO:0034097	response to cytokine	BP	6	2	562	60	19809	5.11E-01
GO:0009725	response to hormone	BP	5	2	562	60	19809	5.11E-01
GO:0001947	heart looping	BP	7	2	562	60	19809	5.11E-01
GO:0016485	protein processing	BP	8	2	562	60	19809	5.11E-01
GO:0045171	intercellular bridge	CC	4	2	562	60	19809	5.11E-01
GO:0004674	protein serine/threonine kinase	MF	8	12	562	414	19809	5.11E-01
GO:0042981	regulation of apoptotic process	BP	8	8	562	273	19809	5.13E-01
GO:0005657	replication fork	CC	6	1	562	25	19809	5.13E-01
GO:0002227	innate immune response in mu	BP	7	1	562	25	19809	5.13E-01
GO:0010613	positive regulation of cardiac r	BP	10	1	562	25	19809	5.13E-01
GO:0055074	calcium ion homeostasis	BP	10	1	562	25	19809	5.13E-01
GO:0097755	positive regulation of blood ve	BP	9	1	562	25	19809	5.13E-01
GO:0071901	negative regulation of protein :	BP	12	1	562	25	19809	5.13E-01
GO:0030866	cortical actin cytoskeleton orga	BP	8	1	562	25	19809	5.13E-01
GO:0055010	ventricular cardiac muscle tiss	BP	8	1	562	25	19809	5.13E-01
GO:0090103	cochlea morphogenesis	BP	6	1	562	25	19809	5.13E-01
GO:2000377	regulation of reactive oxygen s	BP	7	1	562	25	19809	5.13E-01
GO:0045503	dynein light chain binding	MF	5	1	562	25	19809	5.13E-01
GO:0034394	protein localization to cell surf	BP	7	1	562	25	19809	5.13E-01
GO:0048019	receptor antagonist activity	MF	6	1	562	25	19809	5.13E-01
GO:0035902	response to immobilization str	BP	5	1	562	25	19809	5.13E-01
GO:0032735	positive regulation of interleuk	BP	8	1	562	25	19809	5.13E-01
GO:0051233	spindle midzone	CC	6	1	562	25	19809	5.13E-01
GO:0002474	antigen processing and present	BP	6	1	562	25	19809	5.13E-01
GO:0030878	thyroid gland development	BP	7	1	562	25	19809	5.13E-01
GO:0008135	translation factor activity, RN/	MF	7	1	562	25	19809	5.13E-01
GO:0031116	positive regulation of microtut	BP	10	1	562	25	19809	5.13E-01
GO:0010499	proteasomal ubiquitin-indepen	BP	9	1	562	25	19809	5.13E-01
GO:0015035	protein disulfide oxidoreducta:	MF	7	1	562	25	19809	5.13E-01
GO:0051018	protein kinase A binding	MF	5	1	562	25	19809	5.13E-01
GO:0001944	vasculature development	BP	6	1	562	25	19809	5.13E-01
GO:1900182	positive regulation of protein l	BP	9	1	562	25	19809	5.13E-01
GO:0045666	positive regulation of neuron d	BP	10	3	562	96	19809	5.15E-01

GO:0016310	phosphorylation	BP	7	20	562	700	19809	5.20E-01
GO:2000134	negative regulation of G1/S tra	BP	10	2	562	61	19809	5.20E-01
GO:0099056	integral component of presyna	CC	6	2	562	61	19809	5.20E-01
GO:0035064	methylated histone binding	MF	6	2	562	61	19809	5.20E-01
GO:0000079	regulation of cyclin-dependent	BP	12	2	562	61	19809	5.20E-01
GO:0009636	response to toxic substance	BP	5	3	562	97	19809	5.22E-01
GO:0005874	microtubule	CC	7	10	562	347	19809	5.25E-01
GO:0048041	focal adhesion assembly	BP	9	1	562	26	19809	5.27E-01
GO:1990782	protein tyrosine kinase binding	MF	8	1	562	26	19809	5.27E-01
GO:0043015	gamma-tubulin binding	MF	7	1	562	26	19809	5.27E-01
GO:0042162	telomeric DNA binding	MF	8	1	562	26	19809	5.27E-01
GO:0030206	chondroitin sulfate biosyntheti	BP	9	1	562	26	19809	5.27E-01
GO:0000188	inactivation of MAPK activity	BP	13	1	562	26	19809	5.27E-01
GO:0045499	chemorepellent activity	MF	7	1	562	26	19809	5.27E-01
GO:1903588	negative regulation of blood ve	BP	10	1	562	26	19809	5.27E-01
GO:0043403	skeletal muscle tissue regenera	BP	7	1	562	26	19809	5.27E-01
GO:0005719	nuclear euchromatin	CC	8	1	562	26	19809	5.27E-01
GO:0035257	nuclear hormone receptor bind	MF	7	1	562	26	19809	5.27E-01
GO:0004407	histone deacetylase activity	MF	7	1	562	26	19809	5.27E-01
GO:0004435	phosphatidylinositol phospholi	MF	9	1	562	26	19809	5.27E-01
GO:0070372	regulation of ERK1 and ERK2	BP	11	1	562	26	19809	5.27E-01
GO:0016627	oxidoreductase activity, acting	MF	5	1	562	26	19809	5.27E-01
GO:0031018	endocrine pancreas developme	BP	5	1	562	26	19809	5.27E-01
GO:0030261	chromosome condensation	BP	9	1	562	26	19809	5.27E-01
GO:0042755	eating behavior	BP	5	1	562	26	19809	5.27E-01
GO:0051090	regulation of DNA-binding tra	BP	9	1	562	26	19809	5.27E-01
GO:0045861	negative regulation of proteoly	BP	9	1	562	26	19809	5.27E-01
GO:0022400	regulation of rhodopsin mediat	BP	9	1	562	26	19809	5.27E-01
GO:0098586	cellular response to virus	BP	8	1	562	26	19809	5.27E-01
GO:0045334	clathrin-coated endocytic vesic	CC	9	1	562	26	19809	5.27E-01
GO:0009967	positive regulation of signal tra	BP	8	2	562	62	19809	5.29E-01
GO:0035580	specific granule lumen	CC	8	2	562	62	19809	5.29E-01
GO:0016605	PML body	CC	8	3	562	98	19809	5.29E-01
GO:0019900	kinase binding	MF	6	3	562	98	19809	5.29E-01
GO:0005770	late endosome	CC	8	4	562	135	19809	5.36E-01
GO:0030133	transport vesicle	CC	7	3	562	99	19809	5.36E-01
GO:0048208	COPII vesicle coating	BP	8	2	562	63	19809	5.37E-01
GO:0005902	microvillus	CC	7	2	562	63	19809	5.37E-01
GO:0043086	negative regulation of catalytic	BP	6	2	562	63	19809	5.37E-01
GO:0015721	bile acid and bile salt transport	BP	8	1	562	27	19809	5.40E-01
GO:0043409	negative regulation of MAPK	BP	11	1	562	27	19809	5.40E-01
GO:0032809	neuronal cell body membrane	CC	6	1	562	27	19809	5.40E-01
GO:0019897	extrinsic component of plasma	CC	5	1	562	27	19809	5.40E-01
GO:0006261	DNA-dependent DNA replicat	BP	9	1	562	27	19809	5.40E-01
GO:0004004	ATP-dependent RNA helicase	MF	11	1	562	27	19809	5.40E-01
GO:0031519	PcG protein complex	CC	4	1	562	27	19809	5.40E-01
GO:0070371	ERK1 and ERK2 cascade	BP	12	1	562	27	19809	5.40E-01
GO:0004114	3',5'-cyclic-nucleotide phospho	MF	9	1	562	27	19809	5.40E-01
GO:0001968	fibronectin binding	MF	5	1	562	27	19809	5.40E-01
GO:0022604	regulation of cell morphogenes	BP	7	1	562	27	19809	5.40E-01

GO:0031210	phosphatidylcholine binding	MF	6	1	562	27	19809	5.40E-01
GO:0005540	hyaluronic acid binding	MF	6	1	562	27	19809	5.40E-01
GO:0032689	negative regulation of interfer	BP	8	1	562	27	19809	5.40E-01
GO:1903672	positive regulation of sproutin	BP	9	1	562	27	19809	5.40E-01
GO:0000027	ribosomal large subunit assem	BP	9	1	562	27	19809	5.40E-01
GO:0033137	negative regulation of peptidyl	BP	11	1	562	27	19809	5.40E-01
GO:0008021	synaptic vesicle	CC	4	4	562	136	19809	5.41E-01
GO:0007249	I-kappaB kinase/NF-kappaB si	BP	6	2	562	64	19809	5.46E-01
GO:0043408	regulation of MAPK cascade	BP	10	2	562	64	19809	5.46E-01
GO:0097190	apoptotic signaling pathway	BP	5	2	562	64	19809	5.46E-01
GO:0003697	single-stranded DNA binding	MF	7	3	562	101	19809	5.49E-01
GO:0007218	neuropeptide signaling pathwa	BP	6	3	562	101	19809	5.49E-01
GO:0000278	mitotic cell cycle	BP	5	5	562	174	19809	5.51E-01
GO:0010332	response to gamma radiation	BP	7	1	562	28	19809	5.54E-01
GO:0090383	phagosome acidification	BP	13	1	562	28	19809	5.54E-01
GO:0001835	blastocyst hatching	BP	6	1	562	28	19809	5.54E-01
GO:0007422	peripheral nervous system dev	BP	6	1	562	28	19809	5.54E-01
GO:0030507	spectrin binding	MF	6	1	562	28	19809	5.54E-01
GO:0032757	positive regulation of interleuk	BP	8	1	562	28	19809	5.54E-01
GO:0044295	axonal growth cone	CC	6	1	562	28	19809	5.54E-01
GO:0004181	metallocarboxypeptidase activ	MF	9	1	562	28	19809	5.54E-01
GO:0007159	leukocyte cell-cell adhesion	BP	6	1	562	28	19809	5.54E-01
GO:0045776	negative regulation of blood pı	BP	6	1	562	28	19809	5.54E-01
GO:0010971	positive regulation of G2/M tr	BP	10	1	562	28	19809	5.54E-01
GO:0050770	regulation of axonogenesis	BP	9	1	562	28	19809	5.54E-01
GO:0005790	smooth endoplasmic reticulum	CC	7	1	562	28	19809	5.54E-01
GO:0030136	clathrin-coated vesicle	CC	8	2	562	65	19809	5.54E-01
GO:0007612	learning	BP	5	2	562	65	19809	5.54E-01
GO:0050680	negative regulation of epitheli	BP	8	2	562	65	19809	5.54E-01
GO:0005524	ATP binding	MF	5	42	562	1499	19809	5.58E-01
GO:0050821	protein stabilization	BP	6	5	562	176	19809	5.62E-01
GO:0051865	protein autoubiquitination	BP	12	2	562	66	19809	5.62E-01
GO:0005776	autophagosome	CC	7	2	562	66	19809	5.62E-01
GO:0005768	endosome	CC	7	18	562	645	19809	5.63E-01
GO:0005198	structural molecule activity	MF	3	7	562	249	19809	5.63E-01
GO:0048514	blood vessel morphogenesis	BP	6	1	562	29	19809	5.66E-01
GO:0050873	brown fat cell differentiation	BP	7	1	562	29	19809	5.66E-01
GO:0043001	Golgi to plasma membrane pro	BP	10	1	562	29	19809	5.66E-01
GO:0015267	channel activity	MF	6	1	562	29	19809	5.66E-01
GO:0004712	protein serine/threonine/tyrosi	MF	8	1	562	29	19809	5.66E-01
GO:0004129	cytochrome-c oxidase activity	MF	6	1	562	29	19809	5.66E-01
GO:0030001	metal ion transport	BP	8	1	562	29	19809	5.66E-01
GO:0007569	cell aging	BP	5	1	562	29	19809	5.66E-01
GO:0048663	neuron fate commitment	BP	6	1	562	29	19809	5.66E-01
GO:0051959	dynein light intermediate chair	MF	5	1	562	29	19809	5.66E-01
GO:0010803	regulation of tumor necrosis fa	BP	9	1	562	29	19809	5.66E-01
GO:0007129	synapsis	BP	8	1	562	29	19809	5.66E-01
GO:0051085	chaperone cofactor-dependent	BP	7	1	562	29	19809	5.66E-01
GO:0035249	synaptic transmission, glutama	BP	10	1	562	29	19809	5.66E-01
GO:0061512	protein localization to cilium	BP	8	1	562	29	19809	5.66E-01

GO:0019933	cAMP-mediated signaling	BP	8	1	562	29	19809	5.66E-01
GO:0005261	cation channel activity	MF	7	1	562	29	19809	5.66E-01
GO:0021537	telencephalon development	BP	5	1	562	29	19809	5.66E-01
GO:0043539	protein serine/threonine kinase	MF	8	1	562	29	19809	5.66E-01
GO:0006378	mRNA polyadenylation	BP	12	1	562	29	19809	5.66E-01
GO:0031016	pancreas development	BP	6	1	562	29	19809	5.66E-01
GO:0035198	miRNA binding	MF	8	1	562	29	19809	5.66E-01
GO:0030449	regulation of complement activ	BP	7	3	562	104	19809	5.69E-01
GO:0008565	protein transporter activity	MF		2	562	67	19809	5.71E-01
GO:0001932	regulation of protein phosphor	BP	9	2	562	67	19809	5.71E-01
GO:0043565	sequence-specific DNA bindin	MF	7	18	562	648	19809	5.71E-01
GO:0003676	nucleic acid binding	MF	5	40	562	1435	19809	5.71E-01
GO:0000287	magnesium ion binding	MF	7	6	562	215	19809	5.74E-01
GO:0004386	helicase activity	MF	9	4	562	142	19809	5.76E-01
GO:0009952	anterior/posterior pattern speci	BP	6	3	562	105	19809	5.76E-01
GO:0006362	transcription elongation from I	BP	9	1	562	30	19809	5.79E-01
GO:0005774	vacuolar membrane	CC	7	1	562	30	19809	5.79E-01
GO:0045505	dynein intermediate chain binc	MF	5	1	562	30	19809	5.79E-01
GO:0015991	ATP hydrolysis coupled proto	BP	11	1	562	30	19809	5.79E-01
GO:0070979	protein K11-linked ubiquitinat	BP	13	1	562	30	19809	5.79E-01
GO:0090314	positive regulation of protein t	BP	11	1	562	30	19809	5.79E-01
GO:0090200	positive regulation of release c	BP	10	1	562	30	19809	5.79E-01
GO:0015698	inorganic anion transport	BP	8	1	562	30	19809	5.79E-01
GO:0051276	chromosome organization	BP	6	1	562	30	19809	5.79E-01
GO:0007202	activation of phospholipase C	BP	11	1	562	30	19809	5.79E-01
GO:0038111	interleukin-7-mediated signalii	BP	7	1	562	30	19809	5.79E-01
GO:0033116	endoplasmic reticulum-Golgi i	CC	6	2	562	68	19809	5.79E-01
GO:0005741	mitochondrial outer membrane	CC	6	5	562	181	19809	5.87E-01
GO:0009267	cellular response to starvation	BP	8	2	562	69	19809	5.87E-01
GO:0031398	positive regulation of protein u	BP	11	2	562	69	19809	5.87E-01
GO:0003725	double-stranded RNA binding	MF	7	2	562	69	19809	5.87E-01
GO:0001558	regulation of cell growth	BP	6	2	562	69	19809	5.87E-01
GO:0006417	regulation of translation	BP	9	4	562	144	19809	5.87E-01
GO:0004003	ATP-dependent DNA helicase	MF	11	1	562	31	19809	5.91E-01
GO:0038128	ERBB2 signaling pathway	BP	9	1	562	31	19809	5.91E-01
GO:0098664	G protein-coupled serotonin re	BP	6	1	562	31	19809	5.91E-01
GO:2000145	regulation of cell motility	BP	6	1	562	31	19809	5.91E-01
GO:0005771	multivesicular body	CC	9	1	562	31	19809	5.91E-01
GO:0060076	excitatory synapse	CC	4	1	562	31	19809	5.91E-01
GO:0017080	sodium channel regulator activ	MF	5	1	562	31	19809	5.91E-01
GO:0017156	calcium ion regulated exocyto	BP	9	1	562	31	19809	5.91E-01
GO:0044853	plasma membrane raft	CC	7	1	562	31	19809	5.91E-01
GO:0007586	digestion	BP	4	1	562	31	19809	5.91E-01
GO:0045664	regulation of neuron differenti	BP	9	1	562	31	19809	5.91E-01
GO:0006363	termination of RNA polymeras	BP	9	1	562	31	19809	5.91E-01
GO:2000310	regulation of NMDA receptor	BP	10	1	562	31	19809	5.91E-01
GO:0097193	intrinsic apoptotic signaling pa	BP	6	1	562	31	19809	5.91E-01
GO:0098609	cell-cell adhesion	BP	5	4	562	145	19809	5.92E-01
GO:0030900	forebrain development	BP	5	2	562	70	19809	5.95E-01
GO:0006633	fatty acid biosynthetic process	BP	10	2	562	70	19809	5.95E-01

GO:0015078	proton transmembrane transpo	MF	9	1	562	32	19809	6.02E-01
GO:0048863	stem cell differentiation	BP	6	1	562	32	19809	6.02E-01
GO:0034612	response to tumor necrosis fac	BP	7	1	562	32	19809	6.02E-01
GO:0046540	U4/U6 x U5 tri-snRNP comple	CC	9	1	562	32	19809	6.02E-01
GO:0045736	negative regulation of cyclin-d	BP	13	1	562	32	19809	6.02E-01
GO:0016239	positive regulation of macroau	BP	10	1	562	32	19809	6.02E-01
GO:0006361	transcription initiation from Rl	BP	9	1	562	32	19809	6.02E-01
GO:0045907	positive regulation of vasocons	BP	10	1	562	32	19809	6.02E-01
GO:0097192	extrinsic apoptotic signaling pa	BP	6	1	562	32	19809	6.02E-01
GO:0043552	positive regulation of phospha	BP	10	1	562	32	19809	6.02E-01
GO:0071108	protein K48-linked deubiquitir	BP	10	1	562	32	19809	6.02E-01
GO:0016788	hydrolase activity, acting on es	MF	5	1	562	32	19809	6.02E-01
GO:0008089	anterograde axonal transport	BP	11	1	562	32	19809	6.02E-01
GO:0004993	G protein-coupled serotonin re	MF	8	1	562	32	19809	6.02E-01
GO:0044344	cellular response to fibroblast	BP	8	1	562	32	19809	6.02E-01
GO:0000123	histone acetyltransferase comp	CC	6	1	562	32	19809	6.02E-01
GO:0032480	negative regulation of type I in	BP	8	1	562	32	19809	6.02E-01
GO:0030117	membrane coat	CC	6	1	562	32	19809	6.02E-01
GO:0016616	oxidoreductase activity, acting	MF	6	1	562	32	19809	6.02E-01
GO:0030670	phagocytic vesicle membrane	CC	8	2	562	71	19809	6.02E-01
GO:0043154	negative regulation of cysteine	BP	10	2	562	71	19809	6.02E-01
GO:0006464	cellular protein modification p	BP	8	3	562	110	19809	6.07E-01
GO:0010506	regulation of autophagy	BP	8	2	562	72	19809	6.10E-01
GO:0032436	positive regulation of proteaso	BP	12	2	562	72	19809	6.10E-01
GO:0005251	delayed rectifier potassium cha	MF	10	1	562	33	19809	6.13E-01
GO:0048678	response to axon injury	BP	6	1	562	33	19809	6.13E-01
GO:0030073	insulin secretion	BP	11	1	562	33	19809	6.13E-01
GO:0030513	positive regulation of BMP sig	BP	11	1	562	33	19809	6.13E-01
GO:0071949	FAD binding	MF	7	1	562	33	19809	6.13E-01
GO:0008625	extrinsic apoptotic signaling pa	BP	7	1	562	33	19809	6.13E-01
GO:0043967	histone H4 acetylation	BP	11	1	562	33	19809	6.13E-01
GO:0006370	7-methylguanosine mRNA cap	BP	11	1	562	33	19809	6.13E-01
GO:0018279	protein N-linked glycosylation	BP	11	1	562	33	19809	6.13E-01
GO:0070059	intrinsic apoptotic signaling pa	BP	7	1	562	33	19809	6.13E-01
GO:0045815	positive regulation of gene exp	BP	9	1	562	33	19809	6.13E-01
GO:0006904	vesicle docking involved in ex	BP	7	1	562	33	19809	6.13E-01
GO:0033574	response to testosterone	BP	6	1	562	33	19809	6.13E-01
GO:0032839	dendrite cytoplasm	CC	9	1	562	33	19809	6.13E-01
GO:0043928	exonucleolytic nuclear-transcr	BP	13	1	562	33	19809	6.13E-01
GO:0007264	small GTPase mediated signal	BP	6	3	562	111	19809	6.14E-01
GO:0070268	cornification	BP	6	3	562	111	19809	6.14E-01
GO:0043197	dendritic spine	CC	7	4	562	149	19809	6.14E-01
GO:0042391	regulation of membrane potent	BP	5	3	562	112	19809	6.20E-01
GO:0004721	phosphoprotein phosphatase ac	MF	8	4	562	151	19809	6.24E-01
GO:0008236	serine-type peptidase activity	MF	6	4	562	151	19809	6.24E-01
GO:0032148	activation of protein kinase B a	BP	13	1	562	34	19809	6.24E-01
GO:0032212	positive regulation of telomere	BP	12	1	562	34	19809	6.24E-01
GO:0070830	bicellular tight junction assemb	BP	9	1	562	34	19809	6.24E-01
GO:0003281	ventricular septum developmen	BP	6	1	562	34	19809	6.24E-01
GO:0030594	neurotransmitter receptor activ	MF	5	1	562	34	19809	6.24E-01

GO:1903671	negative regulation of sproutin	BP	9	1	562	34	19809	6.24E-01
GO:0002027	regulation of heart rate	BP	9	1	562	34	19809	6.24E-01
GO:0006479	protein methylation	BP	10	1	562	34	19809	6.24E-01
GO:0000077	DNA damage checkpoint	BP	7	1	562	34	19809	6.24E-01
GO:0098686	hippocampal mossy fiber to C/	CC	5	1	562	34	19809	6.24E-01
GO:0007616	long-term memory	BP	6	1	562	34	19809	6.24E-01
GO:0030879	mammary gland development	BP	7	1	562	34	19809	6.24E-01
GO:0007281	germ cell development	BP	5	1	562	34	19809	6.24E-01
GO:0030855	epithelial cell differentiation	BP	6	2	562	74	19809	6.25E-01
GO:0002479	antigen processing and present	BP	8	2	562	74	19809	6.25E-01
GO:0045444	fat cell differentiation	BP	6	2	562	74	19809	6.25E-01
GO:0003677	DNA binding	MF	6	63	562	2294	19809	6.30E-01
GO:0070373	negative regulation of ERK1 a	BP	12	2	562	75	19809	6.32E-01
GO:0045727	positive regulation of translatio	BP	10	2	562	75	19809	6.32E-01
GO:0006661	phosphatidylinositol biosynthe	BP	10	2	562	75	19809	6.32E-01
GO:0051291	protein heterooligomerization	BP	8	2	562	75	19809	6.32E-01
GO:0006811	ion transport	BP	6	18	562	672	19809	6.33E-01
GO:0019731	antibacterial humoral response	BP	7	1	562	35	19809	6.35E-01
GO:0030521	androgen receptor signaling pa	BP	7	1	562	35	19809	6.35E-01
GO:0005875	microtubule associated comple	CC	4	1	562	35	19809	6.35E-01
GO:0005319	lipid transporter activity	MF	4	1	562	35	19809	6.35E-01
GO:0098656	anion transmembrane transpor	BP	8	1	562	35	19809	6.35E-01
GO:0042169	SH2 domain binding	MF	6	1	562	35	19809	6.35E-01
GO:0000118	histone deacetylase complex	CC	7	1	562	35	19809	6.35E-01
GO:0048568	embryonic organ development	BP	6	1	562	35	19809	6.35E-01
GO:0046627	negative regulation of insulin r	BP	9	1	562	35	19809	6.35E-01
GO:0071479	cellular response to ionizing ra	BP	7	1	562	35	19809	6.35E-01
GO:0006099	tricarboxylic acid cycle	BP	5	1	562	35	19809	6.35E-01
GO:0031305	integral component of mitocho	CC	8	1	562	35	19809	6.35E-01
GO:0006259	DNA metabolic process	BP	7	1	562	35	19809	6.35E-01
GO:0033017	sarcoplasmic reticulum membr	CC	6	1	562	35	19809	6.35E-01
GO:0016358	dendrite development	BP	8	1	562	35	19809	6.35E-01
GO:0004860	protein kinase inhibitor activity	MF	7	1	562	35	19809	6.35E-01
GO:0006897	endocytosis	BP	7	5	562	193	19809	6.43E-01
GO:0009653	anatomical structure morphoge	BP	4	3	562	116	19809	6.43E-01
GO:0015171	amino acid transmembrane trai	MF	7	1	562	36	19809	6.46E-01
GO:0006641	triglyceride metabolic process	BP	9	1	562	36	19809	6.46E-01
GO:0048477	oogenesis	BP	6	1	562	36	19809	6.46E-01
GO:2000272	negative regulation of signalin	BP	9	1	562	36	19809	6.46E-01
GO:0043198	dendritic shaft	CC	5	1	562	36	19809	6.46E-01
GO:0050772	positive regulation of axonoge	BP	10	1	562	36	19809	6.46E-01
GO:0030010	establishment of cell polarity	BP	5	1	562	36	19809	6.46E-01
GO:0033572	transferrin transport	BP	11	1	562	36	19809	6.46E-01
GO:0042026	protein refolding	BP	5	1	562	36	19809	6.46E-01
GO:0007093	mitotic cell cycle checkpoint	BP	5	1	562	36	19809	6.46E-01
GO:0001540	amyloid-beta binding	MF	6	2	562	77	19809	6.46E-01
GO:0003777	microtubule motor activity	MF	10	2	562	77	19809	6.46E-01
GO:0005216	ion channel activity	MF	6	5	562	194	19809	6.48E-01
GO:0003730	mRNA 3'-UTR binding	MF	8	2	562	78	19809	6.53E-01
GO:0051259	protein complex oligomerizatio	BP	7	2	562	78	19809	6.53E-01

GO:0005623	cell	CC	3	6	562	234	19809	6.55E-01
GO:0030177	positive regulation of Wnt sigr	BP	9	1	562	37	19809	6.56E-01
GO:0048786	presynaptic active zone	CC	4	1	562	37	19809	6.56E-01
GO:0007588	excretion	BP	5	1	562	37	19809	6.56E-01
GO:0042476	odontogenesis	BP	6	1	562	37	19809	6.56E-01
GO:0001654	eye development	BP	7	1	562	37	19809	6.56E-01
GO:0048286	lung alveolus development	BP	5	1	562	37	19809	6.56E-01
GO:0032991	protein-containing complex	CC	3	18	562	682	19809	6.57E-01
GO:0005764	lysosome	CC	8	10	562	386	19809	6.59E-01
GO:0005811	lipid droplet	CC	6	2	562	79	19809	6.60E-01
GO:0006096	glycolytic process	BP	10	1	562	38	19809	6.65E-01
GO:0031463	Cul3-RING ubiquitin ligase co	CC	7	1	562	38	19809	6.65E-01
GO:0055088	lipid homeostasis	BP	7	1	562	38	19809	6.65E-01
GO:0017112	Rab guanyl-nucleotide exchan	MF	9	1	562	38	19809	6.65E-01
GO:0006024	glycosaminoglycan biosynthet	BP	8	1	562	38	19809	6.65E-01
GO:0060045	positive regulation of cardiac r	BP	8	1	562	38	19809	6.65E-01
GO:0051287	NAD binding	MF	6	1	562	38	19809	6.65E-01
GO:1990090	cellular response to nerve grov	BP	8	1	562	38	19809	6.65E-01
GO:0086091	regulation of heart rate by card	BP	10	1	562	38	19809	6.65E-01
GO:0006469	negative regulation of protein l	BP	11	2	562	80	19809	6.67E-01
GO:0000209	protein polyubiquitination	BP	12	6	562	237	19809	6.67E-01
GO:0030308	negative regulation of cell grov	BP	7	3	562	121	19809	6.71E-01
GO:0042734	presynaptic membrane	CC	5	2	562	81	19809	6.74E-01
GO:0006606	protein import into nucleus	BP	11	2	562	81	19809	6.74E-01
GO:0007040	lysosome organization	BP	8	1	562	39	19809	6.75E-01
GO:0007257	activation of JUN kinase activi	BP	14	1	562	39	19809	6.75E-01
GO:0004180	carboxypeptidase activity	MF	8	1	562	39	19809	6.75E-01
GO:1904315	transmitter-gated ion channel a	MF	10	1	562	39	19809	6.75E-01
GO:0048813	dendrite morphogenesis	BP	9	1	562	39	19809	6.75E-01
GO:0043537	negative regulation of blood ve	BP	11	1	562	39	19809	6.75E-01
GO:0032266	phosphatidylinositol-3-phosph	MF	8	1	562	39	19809	6.75E-01
GO:0008094	DNA-dependent ATPase activ	MF	11	1	562	39	19809	6.75E-01
GO:0042307	positive regulation of protein i	BP	10	1	562	39	19809	6.75E-01
GO:0061098	positive regulation of protein t	BP	12	1	562	39	19809	6.75E-01
GO:1904706	negative regulation of vasculai	BP	9	1	562	39	19809	6.75E-01
GO:0006687	glycosphingolipid metabolic p	BP	9	1	562	39	19809	6.75E-01
GO:0009409	response to cold	BP	5	1	562	39	19809	6.75E-01
GO:0010468	regulation of gene expression	BP	7	6	562	240	19809	6.79E-01
GO:0007613	memory	BP	5	2	562	82	19809	6.80E-01
GO:0006910	phagocytosis, recognition	BP	5	2	562	82	19809	6.80E-01
GO:0008150	biological_process	BP	2	15	562	581	19809	6.82E-01
GO:0035735	intraciliary transport involved	BP	8	1	562	40	19809	6.84E-01
GO:0007017	microtubule-based process	BP	4	1	562	40	19809	6.84E-01
GO:0016575	histone deacetylation	BP	11	1	562	40	19809	6.84E-01
GO:0031640	killing of cells of other organis	BP	4	1	562	40	19809	6.84E-01
GO:0001784	phosphotyrosine residue bindi	MF	7	1	562	40	19809	6.84E-01
GO:0007032	endosome organization	BP	7	1	562	40	19809	6.84E-01
GO:0005267	potassium channel activity	MF	8	2	562	83	19809	6.87E-01
GO:0030307	positive regulation of cell grov	BP	7	2	562	83	19809	6.87E-01
GO:0016042	lipid catabolic process	BP	6	3	562	124	19809	6.87E-01

GO:0006260	DNA replication	BP	8	4	562	165	19809	6.92E-01
GO:0000932	P-body	CC	8	2	562	84	19809	6.93E-01
GO:0071277	cellular response to calcium io	BP	8	2	562	84	19809	6.93E-01
GO:0030276	clathrin binding	MF	5	1	562	41	19809	6.93E-01
GO:0006749	glutathione metabolic process	BP	8	1	562	41	19809	6.93E-01
GO:0070317	negative regulation of G0 to G	BP	9	1	562	41	19809	6.93E-01
GO:0043407	negative regulation of MAP ki	BP	12	1	562	41	19809	6.93E-01
GO:0030286	dynein complex	CC	5	1	562	41	19809	6.93E-01
GO:0030018	Z disc	CC	5	3	562	126	19809	6.98E-01
GO:0005923	bicellular tight junction	CC	6	3	562	126	19809	6.98E-01
GO:0016779	nucleotidyltransferase activity	MF	6	2	562	85	19809	6.99E-01
GO:0007059	chromosome segregation	BP	4	2	562	85	19809	6.99E-01
GO:0042220	response to cocaine	BP	6	1	562	42	19809	7.02E-01
GO:0050909	sensory perception of taste	BP	8	1	562	42	19809	7.02E-01
GO:0030331	estrogen receptor binding	MF	9	1	562	42	19809	7.02E-01
GO:0035097	histone methyltransferase com	CC	6	1	562	42	19809	7.02E-01
GO:0032391	photoreceptor connecting ciliu	CC	6	1	562	42	19809	7.02E-01
GO:0006654	phosphatidic acid biosynthetic	BP	10	1	562	42	19809	7.02E-01
GO:0007098	centrosome cycle	BP	5	1	562	42	19809	7.02E-01
GO:0045214	sarcomere organization	BP	9	1	562	42	19809	7.02E-01
GO:0051539	4 iron, 4 sulfur cluster binding	MF	6	1	562	42	19809	7.02E-01
GO:0003713	transcription coactivator activi	MF	5	7	562	285	19809	7.02E-01
GO:0044325	ion channel binding	MF	5	3	562	127	19809	7.03E-01
GO:0046330	positive regulation of JNK cas	BP	13	2	562	86	19809	7.05E-01
GO:0006956	complement activation	BP	5	2	562	86	19809	7.05E-01
GO:0014070	response to organic cyclic con	BP	6	3	562	128	19809	7.08E-01
GO:0019894	kinesin binding	MF	6	1	562	43	19809	7.10E-01
GO:0005245	voltage-gated calcium channel	MF	9	1	562	43	19809	7.10E-01
GO:0034332	adherens junction organization	BP	6	1	562	43	19809	7.10E-01
GO:0016235	aggresome	CC	6	1	562	43	19809	7.10E-01
GO:0003899	DNA-directed 5'-3' RNA polyr	MF	9	1	562	43	19809	7.10E-01
GO:0016798	hydrolase activity, acting on gl	MF	5	2	562	87	19809	7.11E-01
GO:0032580	Golgi cisterna membrane	CC	8	2	562	87	19809	7.11E-01
GO:0034220	ion transmembrane transport	BP	7	7	562	289	19809	7.16E-01
GO:0035264	multicellular organism growth	BP	4	2	562	88	19809	7.17E-01
GO:0004519	endonuclease activity	MF	7	2	562	88	19809	7.17E-01
GO:0001658	branching involved in ureteric	BP	7	1	562	44	19809	7.19E-01
GO:0007043	cell-cell junction assembly	BP	7	1	562	44	19809	7.19E-01
GO:0006665	sphingolipid metabolic process	BP	8	1	562	44	19809	7.19E-01
GO:0071005	U2-type precatalytic spliceoso	CC	8	1	562	44	19809	7.19E-01
GO:0050681	androgen receptor binding	MF	9	1	562	44	19809	7.19E-01
GO:0042645	mitochondrial nucleoid	CC	4	1	562	44	19809	7.19E-01
GO:0006814	sodium ion transport	BP	9	3	562	131	19809	7.22E-01
GO:0008333	endosome to lysosome transpo	BP	8	1	562	45	19809	7.27E-01
GO:0030199	collagen fibril organization	BP	7	1	562	45	19809	7.27E-01
GO:0030658	transport vesicle membrane	CC	7	1	562	45	19809	7.27E-01
GO:0032024	positive regulation of insulin s	BP	11	1	562	45	19809	7.27E-01
GO:0015914	phospholipid transport	BP	8	1	562	45	19809	7.27E-01
GO:0014823	response to activity	BP	4	1	562	45	19809	7.27E-01
GO:0048156	tau protein binding	MF	6	1	562	45	19809	7.27E-01

GO:0090051	negative regulation of cell mig	BP	12	1	562	45	19809	7.27E-01
GO:0097542	ciliary tip	CC	5	1	562	45	19809	7.27E-01
GO:0030315	T-tubule	CC	5	1	562	45	19809	7.27E-01
GO:0005829	cytosol	CC	6	137	562	5029	19809	7.27E-01
GO:0004715	non-membrane spanning prote	MF	9	1	562	46	19809	7.34E-01
GO:0001890	placenta development	BP	6	1	562	46	19809	7.34E-01
GO:0022627	cytosolic small ribosomal subu	CC	8	1	562	46	19809	7.34E-01
GO:0010212	response to ionizing radiation	BP	6	1	562	46	19809	7.34E-01
GO:0031100	animal organ regeneration	BP	6	1	562	46	19809	7.34E-01
GO:0005262	calcium channel activity	MF	8	2	562	92	19809	7.40E-01
GO:0032147	activation of protein kinase act	BP	12	2	562	92	19809	7.40E-01
GO:0001669	acrosomal vesicle	CC	9	2	562	92	19809	7.40E-01
GO:0006605	protein targeting	BP	11	1	562	47	19809	7.42E-01
GO:0120163	negative regulation of cold-ind	BP	7	1	562	47	19809	7.42E-01
GO:0030864	cortical actin cytoskeleton	CC	8	1	562	47	19809	7.42E-01
GO:0003727	single-stranded RNA binding	MF	7	1	562	47	19809	7.42E-01
GO:0004879	nuclear receptor activity	MF	6	1	562	47	19809	7.42E-01
GO:0043124	negative regulation of I-kappaI	BP	10	1	562	47	19809	7.42E-01
GO:0051781	positive regulation of cell divis	BP	7	1	562	47	19809	7.42E-01
GO:0004175	endopeptidase activity	MF	7	2	562	93	19809	7.45E-01
GO:1905515	non-motile cilium assembly	BP	8	1	562	48	19809	7.49E-01
GO:0000723	telomere maintenance	BP	8	1	562	48	19809	7.49E-01
GO:0071902	positive regulation of protein s	BP	12	1	562	48	19809	7.49E-01
GO:0006612	protein targeting to membrane	BP	12	1	562	48	19809	7.49E-01
GO:0031175	neuron projection developmen	BP	7	3	562	138	19809	7.54E-01
GO:0042127	regulation of cell population p	BP	6	4	562	180	19809	7.55E-01
GO:0019886	antigen processing and present	BP	7	2	562	95	19809	7.56E-01
GO:0061337	cardiac conduction	BP	9	1	562	49	19809	7.56E-01
GO:0050885	neuromuscular process control	BP	7	1	562	49	19809	7.56E-01
GO:0009411	response to UV	BP	7	1	562	49	19809	7.56E-01
GO:0045747	positive regulation of Notch si	BP	9	1	562	49	19809	7.56E-01
GO:0008542	visual learning	BP	5	1	562	49	19809	7.56E-01
GO:0045597	positive regulation of cell diffe	BP	7	1	562	49	19809	7.56E-01
GO:0006508	proteolysis	BP	7	16	562	651	19809	7.57E-01
GO:0005769	early endosome	CC	8	6	562	262	19809	7.57E-01
GO:0055085	transmembrane transport	BP	6	16	562	653	19809	7.61E-01
GO:0034613	cellular protein localization	BP	6	1	562	50	19809	7.63E-01
GO:0010507	negative regulation of autopha	BP	9	1	562	50	19809	7.63E-01
GO:0005637	nuclear inner membrane	CC	6	1	562	50	19809	7.63E-01
GO:0075733	intracellular transport of virus	BP	8	1	562	50	19809	7.63E-01
GO:0008146	sulfotransferase activity	MF	6	1	562	50	19809	7.63E-01
GO:0001917	photoreceptor inner segment	CC	5	1	562	50	19809	7.63E-01
GO:0046854	phosphatidylinositol phosphor	BP	10	1	562	50	19809	7.63E-01
GO:0036297	interstrand cross-link repair	BP	9	1	562	50	19809	7.63E-01
GO:0000281	mitotic cytokinesis	BP	7	1	562	51	19809	7.70E-01
GO:0004527	exonuclease activity	MF	7	1	562	51	19809	7.70E-01
GO:0051603	proteolysis involved in cellulaı	BP	8	1	562	51	19809	7.70E-01
GO:0070498	interleukin-1-mediated signaliı	BP	7	2	562	98	19809	7.71E-01
GO:0006911	phagocytosis, engulfment	BP	8	2	562	98	19809	7.71E-01
GO:0006958	complement activation, classic	BP	6	3	562	142	19809	7.71E-01

GO:0045095	keratin filament	CC	8	2	562	99	19809	7.76E-01
GO:0030163	protein catabolic process	BP	7	1	562	52	19809	7.77E-01
GO:0030178	negative regulation of Wnt sig	BP	9	1	562	52	19809	7.77E-01
GO:0032760	positive regulation of tumor ne	BP	9	1	562	52	19809	7.77E-01
GO:0005544	calcium-dependent phospholip	MF	6	1	562	52	19809	7.77E-01
GO:0009306	protein secretion	BP	10	1	562	52	19809	7.77E-01
GO:0019901	protein kinase binding	MF	7	11	562	468	19809	7.78E-01
GO:0005913	cell-cell adherens junction	CC	5	2	562	100	19809	7.80E-01
GO:0000980	RNA polymerase II distal enha	MF	10	2	562	100	19809	7.80E-01
GO:0005635	nuclear envelope	CC	6	4	562	187	19809	7.81E-01
GO:0016887	ATPase activity	MF	9	5	562	229	19809	7.82E-01
GO:0051290	protein heterotetramerization	BP	9	1	562	53	19809	7.83E-01
GO:0005881	cytoplasmic microtubule	CC	8	1	562	53	19809	7.83E-01
GO:0006888	endoplasmic reticulum to Golg	BP	7	4	562	188	19809	7.84E-01
GO:0030030	cell projection organization	BP	5	4	562	188	19809	7.84E-01
GO:0019904	protein domain specific bindin	MF	5	6	562	271	19809	7.84E-01
GO:1903508	positive regulation of nucleic a	BP	10	2	562	101	19809	7.85E-01
GO:0005975	carbohydrate metabolic proces	BP	5	5	562	231	19809	7.88E-01
GO:0001227	DNA-binding transcription rep	MF	6	5	562	231	19809	7.88E-01
GO:0003707	steroid hormone receptor activ	MF	5	1	562	54	19809	7.89E-01
GO:0048704	embryonic skeletal system mo	BP	7	1	562	54	19809	7.89E-01
GO:0042277	peptide binding	MF	5	1	562	54	19809	7.89E-01
GO:0008152	metabolic process	BP	3	4	562	190	19809	7.91E-01
GO:0045814	negative regulation of gene ex	BP	9	1	562	55	19809	7.95E-01
GO:1904115	axon cytoplasm	CC	6	1	562	55	19809	7.95E-01
GO:0043401	steroid hormone mediated sign	BP	6	1	562	55	19809	7.95E-01
GO:0005871	kinesin complex	CC	5	1	562	55	19809	7.95E-01
GO:0043657	host cell	CC	5	1	562	55	19809	7.95E-01
GO:0016459	myosin complex	CC	4	1	562	55	19809	7.95E-01
GO:0003674	MF	MF	2	17	562	711	19809	7.98E-01
GO:0005654	nucleoplasm	CC	6	84	562	3200	19809	8.01E-01
GO:0048365	Rac GTPase binding	MF	10	1	562	56	19809	8.01E-01
GO:0030665	clathrin-coated vesicle membra	CC	8	1	562	56	19809	8.01E-01
GO:0045787	positive regulation of cell cycl	BP	7	1	562	56	19809	8.01E-01
GO:0043022	ribosome binding	MF	6	1	562	56	19809	8.01E-01
GO:0031492	nucleosomal DNA binding	MF	8	1	562	56	19809	8.01E-01
GO:0006366	transcription by RNA polymer	BP	9	6	562	277	19809	8.01E-01
GO:0043621	protein self-association	MF	5	1	562	57	19809	8.07E-01
GO:0030326	embryonic limb morphogenesi	BP	7	1	562	57	19809	8.07E-01
GO:0006338	chromatin remodeling	BP	6	2	562	106	19809	8.07E-01
GO:0006470	protein dephosphorylation	BP	9	4	562	195	19809	8.07E-01
GO:0031966	mitochondrial membrane	CC	5	3	562	152	19809	8.09E-01
GO:0030176	integral component of endopla	CC	7	2	562	107	19809	8.11E-01
GO:0008654	phospholipid biosynthetic proc	BP	8	1	562	58	19809	8.12E-01
GO:0000166	nucleotide binding	MF	5	46	562	1817	19809	8.14E-01
GO:0000045	autophagosome assembly	BP	7	1	562	59	19809	8.17E-01
GO:0019003	GDP binding	MF	8	1	562	59	19809	8.17E-01
GO:0045860	positive regulation of protein k	BP	11	1	562	59	19809	8.17E-01
GO:0005759	mitochondrial matrix	CC	7	8	562	366	19809	8.19E-01
GO:0008544	epidermis development	BP	6	2	562	109	19809	8.19E-01

GO:0050853	B cell receptor signaling pathw	BP	8	2	562	109	19809	8.19E-01
GO:0001568	blood vessel development	BP	5	1	562	60	19809	8.23E-01
GO:0016328	lateral plasma membrane	CC	5	1	562	60	19809	8.23E-01
GO:0002223	stimulatory C-type lectin recep	BP	8	2	562	110	19809	8.23E-01
GO:0016740	transferase activity	MF	4	46	562	1826	19809	8.24E-01
GO:0001570	vasculogenesis	BP	6	1	562	61	19809	8.28E-01
GO:0030139	endocytic vesicle	CC	7	1	562	61	19809	8.28E-01
GO:0000151	ubiquitin ligase complex	CC	5	2	562	112	19809	8.31E-01
GO:0031072	heat shock protein binding	MF	5	1	562	62	19809	8.33E-01
GO:0051898	negative regulation of protein l	BP	10	1	562	62	19809	8.33E-01
GO:0007601	visual perception	BP	8	4	562	204	19809	8.34E-01
GO:0005819	spindle	CC	6	4	562	204	19809	8.34E-01
GO:0003774	motor activity	MF	9	2	562	113	19809	8.34E-01
GO:0051536	iron-sulfur cluster binding	MF	5	1	562	63	19809	8.37E-01
GO:0007187	G protein-coupled receptor sig	BP	6	1	562	63	19809	8.37E-01
GO:0048666	neuron development	BP	6	1	562	63	19809	8.37E-01
GO:0006694	steroid biosynthetic process	BP	7	1	562	63	19809	8.37E-01
GO:0045665	negative regulation of neuron c	BP	10	1	562	63	19809	8.37E-01
GO:0032259	methylation	BP	4	4	562	206	19809	8.40E-01
GO:0004722	protein serine/threonine phosp	MF	9	1	562	64	19809	8.42E-01
GO:0031667	response to nutrient levels	BP	6	1	562	64	19809	8.42E-01
GO:0030433	ubiquitin-dependent ERAD pa	BP	8	1	562	64	19809	8.42E-01
GO:0007416	synapse assembly	BP	6	1	562	64	19809	8.42E-01
GO:0005905	clathrin-coated pit	CC	4	1	562	64	19809	8.42E-01
GO:0003682	chromatin binding	MF	4	9	562	418	19809	8.42E-01
GO:0007268	chemical synaptic transmissior	BP	9	5	562	251	19809	8.43E-01
GO:0005765	lysosomal membrane	CC	9	6	562	294	19809	8.44E-01
GO:0002250	adaptive immune response	BP	5	8	562	379	19809	8.46E-01
GO:0003684	damaged DNA binding	MF	7	1	562	65	19809	8.46E-01
GO:0050660	flavin adenine dinucleotide bir	MF	6	1	562	65	19809	8.46E-01
GO:0008217	regulation of blood pressure	BP	5	1	562	65	19809	8.46E-01
GO:0030017	sarcomere	CC	5	1	562	65	19809	8.46E-01
GO:0006281	DNA repair	BP	8	8	562	380	19809	8.48E-01
GO:0007283	spermatogenesis	BP	7	10	562	463	19809	8.50E-01
GO:0043010	camera-type eye development	BP	8	1	562	66	19809	8.51E-01
GO:0002039	p53 binding	MF	5	1	562	66	19809	8.51E-01
GO:0000381	regulation of alternative mRNA	BP	10	1	562	66	19809	8.51E-01
GO:0008233	peptidase activity	MF	5	12	562	545	19809	8.51E-01
GO:0051321	meiotic cell cycle	BP	5	2	562	118	19809	8.52E-01
GO:0005789	endoplasmic reticulum membr	CC	5	23	562	979	19809	8.52E-01
GO:0003729	mRNA binding	MF	7	3	562	166	19809	8.54E-01
GO:0010977	negative regulation of neuron p	BP	11	1	562	67	19809	8.55E-01
GO:0000149	SNARE binding	MF	5	1	562	67	19809	8.55E-01
GO:0005249	voltage-gated potassium chann	MF	9	1	562	68	19809	8.59E-01
GO:0006887	exocytosis	BP	7	2	562	121	19809	8.62E-01
GO:0022857	transmembrane transporter act	MF	4	3	562	170	19809	8.65E-01
GO:0000784	nuclear chromosome, telomeri	CC	8	2	562	122	19809	8.65E-01
GO:0060041	retina development in camera-	BP	5	1	562	70	19809	8.67E-01
GO:0016241	regulation of macroautophagy	BP	9	1	562	71	19809	8.71E-01
GO:0045335	phagocytic vesicle	CC	8	1	562	71	19809	8.71E-01

GO:0032482	Rab protein signal transductioı	BP	8	1	562	71	19809	8.71E-01
GO:0007265	Ras protein signal transductior	BP	7	1	562	71	19809	8.71E-01
GO:0061418	regulation of transcription fror	BP	11	1	562	72	19809	8.75E-01
GO:0090575	RNA polymerase II transcripti	CC	6	1	562	72	19809	8.75E-01
GO:0005546	phosphatidylinositol-4,5-bisph	MF	9	1	562	72	19809	8.75E-01
GO:0042393	histone binding	MF	5	2	562	126	19809	8.76E-01
GO:0009749	response to glucose	BP	9	1	562	73	19809	8.78E-01
GO:0045732	positive regulation of protein c	BP	8	1	562	73	19809	8.78E-01
GO:0060548	negative regulation of cell dea	BP	7	1	562	73	19809	8.78E-01
GO:0036064	ciliary basal body	CC	7	2	562	127	19809	8.79E-01
GO:0004930	G protein-coupled receptor act	MF	6	18	562	806	19809	8.80E-01
GO:0000785	chromatin	CC	6	2	562	128	19809	8.82E-01
GO:0038096	Fc-gamma receptor signaling p	BP	8	2	562	128	19809	8.82E-01
GO:0021987	cerebral cortex development	BP	5	1	562	75	19809	8.85E-01
GO:0072686	mitotic spindle	CC	7	1	562	75	19809	8.85E-01
GO:0006644	phospholipid metabolic proces	BP	7	1	562	75	19809	8.85E-01
GO:0016567	protein ubiquitination	BP	11	12	562	568	19809	8.86E-01
GO:0043130	ubiquitin binding	MF	6	1	562	76	19809	8.88E-01
GO:0000086	G2/M transition of mitotic cell	BP	7	2	562	131	19809	8.90E-01
GO:1901796	regulation of signal transductic	BP	9	2	562	131	19809	8.90E-01
GO:0051301	cell division	BP	4	8	562	404	19809	8.90E-01
GO:0030216	keratinocyte differentiation	BP	8	1	562	77	19809	8.91E-01
GO:0004842	ubiquitin-protein transferase a	MF	6	5	562	274	19809	8.92E-01
GO:0005884	actin filament	CC	7	1	562	78	19809	8.95E-01
GO:0050790	regulation of catalytic activity	BP	5	1	562	78	19809	8.95E-01
GO:0016705	oxidoreductase activity, acting	MF	5	1	562	78	19809	8.95E-01
GO:0001650	fibrillar center	CC	7	2	562	133	19809	8.95E-01
GO:0045892	negative regulation of transcri	BP	9	12	562	577	19809	8.97E-01
GO:0008543	fibroblast growth factor recept	BP	8	1	562	79	19809	8.98E-01
GO:0007338	single fertilization	BP	5	1	562	79	19809	8.98E-01
GO:0071260	cellular response to mechanica	BP	6	1	562	79	19809	8.98E-01
GO:0030672	synaptic vesicle membrane	CC	4	1	562	79	19809	8.98E-01
GO:0061024	membrane organization	BP	5	2	562	135	19809	8.99E-01
GO:0007018	microtubule-based movement	BP	5	1	562	80	19809	9.00E-01
GO:0005758	mitochondrial intermembrane	CC	6	1	562	80	19809	9.00E-01
GO:0000922	spindle pole	CC	6	2	562	138	19809	9.06E-01
GO:0060964	regulation of gene silencing by	BP	10	1	562	82	19809	9.06E-01
GO:0008104	protein localization	BP	5	1	562	82	19809	9.06E-01
GO:0031647	regulation of protein stability	BP	5	1	562	83	19809	9.09E-01
GO:0008286	insulin receptor signaling path	BP	8	1	562	83	19809	9.09E-01
GO:0008168	methyltransferase activity	MF	6	3	562	190	19809	9.09E-01
GO:0072562	blood microparticle	CC	4	2	562	140	19809	9.10E-01
GO:0003712	transcription coregulator activi	MF	4	1	562	84	19809	9.11E-01
GO:0016874	ligase activity	MF	4	2	562	141	19809	9.12E-01
GO:0030674	protein binding, bridging	MF	5	1	562	85	19809	9.14E-01
GO:0031625	ubiquitin protein ligase bindin	MF	7	5	562	288	19809	9.14E-01
GO:0050829	defense response to Gram-neg	BP	9	1	562	86	19809	9.16E-01
GO:0019903	protein phosphatase binding	MF	7	1	562	87	19809	9.19E-01
GO:0004497	monooxygenase activity	MF	5	1	562	87	19809	9.19E-01
GO:0008076	voltage-gated potassium chann	CC	9	1	562	89	19809	9.23E-01

GO:0006898	receptor-mediated endocytosis	BP	8	3	562	199	19809	9.24E-01
GO:0003714	transcription corepressor activ	MF	5	4	562	248	19809	9.24E-01
GO:0005739	mitochondrion	CC	6	35	562	1526	19809	9.24E-01
GO:0035278	miRNA mediated inhibition of	BP	12	1	562	91	19809	9.28E-01
GO:0035578	azurophil granule lumen	CC	8	1	562	91	19809	9.28E-01
GO:0005694	chromosome	CC	6	9	562	477	19809	9.28E-01
GO:0007049	cell cycle	BP	4	13	562	649	19809	9.29E-01
GO:0005244	voltage-gated ion channel acti	MF	9	2	562	150	19809	9.29E-01
GO:0006805	xenobiotic metabolic process	BP	5	1	562	93	19809	9.32E-01
GO:0090630	activation of GTPase activity	BP	9	1	562	94	19809	9.34E-01
GO:0005930	axoneme	CC	6	1	562	94	19809	9.34E-01
GO:0008203	cholesterol metabolic process	BP	8	1	562	94	19809	9.34E-01
GO:0042632	cholesterol homeostasis	BP	9	1	562	96	19809	9.37E-01
GO:0034765	regulation of ion transmembra	BP	8	2	562	156	19809	9.38E-01
GO:0006886	intracellular protein transport	BP	10	6	562	354	19809	9.39E-01
GO:0005681	spliceosomal complex	CC	6	2	562	157	19809	9.40E-01
GO:0008234	cysteine-type peptidase activit	MF	7	2	562	158	19809	9.41E-01
GO:1990904	ribonucleoprotein complex	CC	4	2	562	160	19809	9.44E-01
GO:0005200	structural constituent of cytosk	MF	4	1	562	100	19809	9.44E-01
GO:0003823	antigen binding	MF	4	2	562	161	19809	9.45E-01
GO:0006325	chromatin organization	BP	5	6	562	362	19809	9.46E-01
GO:0031090	organelle membrane	CC	4	1	562	102	19809	9.47E-01
GO:0006310	DNA recombination	BP	8	1	562	102	19809	9.47E-01
GO:0005575	CC	CC	2	7	562	411	19809	9.49E-01
GO:0031982	vesicle	CC	5	2	562	164	19809	9.49E-01
GO:0038095	Fc-epsilon receptor signaling p	BP	8	2	562	166	19809	9.51E-01
GO:0002377	immunoglobulin production	BP	5	1	562	106	19809	9.53E-01
GO:0061630	ubiquitin protein ligase activity	MF	7	3	562	222	19809	9.53E-01
GO:0007015	actin filament organization	BP	6	1	562	108	19809	9.56E-01
GO:0004843	thiol-dependent ubiquitin-spec	MF	10	1	562	112	19809	9.61E-01
GO:0005815	microtubule organizing center	CC	6	7	562	427	19809	9.61E-01
GO:0005543	phospholipid binding	MF	5	1	562	113	19809	9.62E-01
GO:0036459	thiol-dependent ubiquitinyl hy	MF	9	1	562	113	19809	9.62E-01
GO:0043488	regulation of mRNA stability	BP	10	1	562	114	19809	9.63E-01
GO:0006367	transcription initiation from R	BP	9	2	562	178	19809	9.64E-01
GO:0042593	glucose homeostasis	BP	8	1	562	115	19809	9.64E-01
GO:0000184	nuclear-transcribed mRNA cat	BP	12	1	562	119	19809	9.68E-01
GO:0008202	steroid metabolic process	BP	6	1	562	126	19809	9.74E-01
GO:0006974	cellular response to DNA dam	BP	6	8	562	499	19809	9.74E-01
GO:0005634	nucleus	CC	6	166	562	6586	19809	9.75E-01
GO:0045296	cadherin binding	MF	6	4	562	306	19809	9.76E-01
GO:0031424	keratinization	BP	4	2	562	198	19809	9.78E-01
GO:0005882	intermediate filament	CC	7	2	562	205	19809	9.81E-01
GO:0005929	cilium	CC	4	5	562	373	19809	9.82E-01
GO:0031514	motile cilium	CC	5	1	562	141	19809	9.83E-01
GO:0005506	iron ion binding	MF	8	1	562	141	19809	9.83E-01
GO:0016579	protein deubiquitination	BP	9	3	562	269	19809	9.83E-01
GO:0051726	regulation of cell cycle	BP	6	1	562	149	19809	9.86E-01
GO:0016607	nuclear speck	CC	8	5	562	397	19809	9.89E-01
GO:0030496	midbody	CC	4	1	562	157	19809	9.89E-01

GO:0016604	nuclear body	CC	7	3	562	289	19809	9.89E-01
GO:0005730	nucleolus	CC	6	14	562	834	19809	9.90E-01
GO:0005938	cell cortex	CC	7	1	562	161	19809	9.90E-01
GO:0015031	protein transport	BP	9	10	562	657	19809	9.91E-01
GO:0000398	mRNA splicing, via spliceoso	BP	13	2	562	237	19809	9.92E-01
GO:0003735	structural constituent of riboso	MF	4	1	562	170	19809	9.93E-01
GO:0003723	RNA binding	MF	6	29	562	1525	19809	9.94E-01
GO:0005743	mitochondrial inner membrane	CC	6	5	562	442	19809	9.96E-01
GO:0043161	proteasome-mediated ubiquitin	BP	11	1	562	189	19809	9.96E-01
GO:0005840	ribosome	CC	6	1	562	205	19809	9.97E-01
GO:0006511	ubiquitin-dependent protein ca	BP	10	2	562	314	19809	9.99E-01
GO:0006412	translation	BP	8	2	562	318	19809	9.99E-01
GO:0005813	centrosome	CC	7	4	562	485	19809	1.00E+00
GO:0016192	vesicle-mediated transport	BP	6	1	562	278	19809	1.00E+00
GO:0008380	RNA splicing	BP	10	1	562	291	19809	1.00E+00
GO:0006397	mRNA processing	BP	10	2	562	379	19809	1.00E+00
GO:0050896	response to stimulus	BP	3	1	562	569	19809	1.00E+00

Supplementary Table 5. KEGG enrichment analysis of differentially expressed genes.

pathway_id	pathway_name	S.gene.number	TS.gene.number	B.gene.number	TB.gene.number	pvalue
ko04640	Hematopoietic cell lineage	15	259	94	7612	5.29E-07
ko04060	Cytokine-cytokine receptor interaction	24	259	292	7612	5.16E-05
ko04350	TGF-beta signaling pathway	10	259	91	7612	9.91E-04
ko04512	ECM-receptor interaction	8	259	82	7612	6.53E-03
ko05418	Fluid shear stress and atherosclerosis	11	259	137	7612	6.87E-03
ko04371	Apelin signaling pathway	11	259	137	7612	6.87E-03
ko04978	Mineral absorption	6	259	51	7612	7.30E-03
ko04510	Focal adhesion	14	259	199	7612	7.82E-03
ko04151	PI3K-Akt signaling pathway	21	259	353	7612	8.79E-03
ko04380	Osteoclast differentiation	10	259	125	7612	1.00E-02
ko05310	Asthma	4	259	28	7612	1.41E-02
ko00512	Mucin type O-glycan biosynthesis	4	259	31	7612	2.00E-02
ko00052	Galactose metabolism	4	259	31	7612	2.00E-02
ko04750	Inflammatory mediator regulation of TRP cha	8	259	100	7612	2.03E-02
ko05144	Malaria	5	259	48	7612	2.28E-02
ko00770	Pantothenate and CoA biosynthesis	3	259	19	7612	2.52E-02
ko04062	Chemokine signaling pathway	12	259	188	7612	2.65E-02
ko00740	Riboflavin metabolism	2	259	8	7612	2.82E-02
ko05323	Rheumatoid arthritis	7	259	87	7612	2.82E-02
ko04145	Phagosome	10	259	148	7612	2.92E-02
ko05206	MicroRNAs in cancer	10	259	150	7612	3.17E-02
ko05166	Human T-cell leukemia virus 1 infection	13	259	217	7612	3.37E-02
ko05146	Amoebiasis	7	259	95	7612	4.26E-02
ko04360	Axon guidance	11	259	181	7612	4.45E-02
ko05202	Transcriptional misregulation in cancer	11	259	185	7612	5.06E-02
ko04610	Complement and coagulation cascades	6	259	79	7612	5.17E-02
ko04933	AGE-RAGE signaling pathway in diabetic co	7	259	100	7612	5.36E-02
ko04514	Cell adhesion molecules (CAMs)	9	259	144	7612	5.67E-02
ko05200	Pathways in cancer	25	259	528	7612	5.70E-02
ko00380	Tryptophan metabolism	4	259	43	7612	5.73E-02
ko04022	cGMP-PKG signaling pathway	10	259	167	7612	5.84E-02
ko04611	Platelet activation	8	259	124	7612	6.04E-02
ko04926	Relaxin signaling pathway	8	259	130	7612	7.51E-02
ko05205	Proteoglycans in cancer	11	259	199	7612	7.68E-02
ko05163	Human cytomegalovirus infection	12	259	223	7612	7.78E-02
ko00330	Arginine and proline metabolism	4	259	49	7612	8.43E-02
ko05414	Dilated cardiomyopathy (DCM)	6	259	90	7612	8.56E-02
ko04725	Cholinergic synapse	7	259	112	7612	8.68E-02
ko00410	beta-Alanine metabolism	3	259	31	7612	8.70E-02
ko05140	Leishmaniasis	5	259	70	7612	8.92E-02
ko04218	Cellular senescence	9	259	158	7612	8.96E-02
ko04657	IL-17 signaling pathway	6	259	93	7612	9.65E-02
ko04550	Signaling pathways regulating pluripotency of	8	259	139	7612	1.01E-01
ko04071	Sphingolipid signaling pathway	7	259	119	7612	1.11E-01
ko04971	Gastric acid secretion	5	259	75	7612	1.11E-01
ko05330	Allograft rejection	3	259	35	7612	1.15E-01
ko05134	Legionellosis	4	259	55	7612	1.16E-01

ko05416	Viral myocarditis	4	259	56	7612	1.22E-01
ko05340	Primary immunodeficiency	3	259	36	7612	1.23E-01
ko00500	Starch and sucrose metabolism	3	259	36	7612	1.23E-01
ko05143	African trypanosomiasis	3	259	36	7612	1.23E-01
ko05216	Thyroid cancer	3	259	37	7612	1.30E-01
ko05332	Graft-versus-host disease	3	259	37	7612	1.30E-01
ko04621	NOD-like receptor signaling pathway	9	259	177	7612	1.49E-01
ko04928	Parathyroid hormone synthesis, secretion and a	6	259	106	7612	1.52E-01
ko05165	Human papillomavirus infection	15	259	330	7612	1.55E-01
ko05132	Salmonella infection	5	259	84	7612	1.57E-01
ko05219	Bladder cancer	3	259	41	7612	1.62E-01
ko04940	Type I diabetes mellitus	3	259	41	7612	1.62E-01
ko04270	Vascular smooth muscle contraction	7	259	132	7612	1.63E-01
ko05410	Hypertrophic cardiomyopathy (HCM)	5	259	85	7612	1.63E-01
ko04015	Rap1 signaling pathway	10	259	206	7612	1.64E-01
ko04911	Insulin secretion	5	259	86	7612	1.68E-01
ko04310	Wnt signaling pathway	8	259	158	7612	1.70E-01
ko05150	Staphylococcus aureus infection	4	259	64	7612	1.73E-01
ko05167	Kaposi sarcoma-associated herpesvirus infecti	9	259	186	7612	1.83E-01
ko00340	Histidine metabolism	2	259	23	7612	1.83E-01
ko04614	Renin-angiotensin system	2	259	23	7612	1.83E-01
ko04210	Apoptosis	7	259	137	7612	1.85E-01
ko05230	Central carbon metabolism in cancer	4	259	66	7612	1.86E-01
ko04020	Calcium signaling pathway	9	259	187	7612	1.87E-01
ko00750	Vitamin B6 metabolism	1	259	6	7612	1.88E-01
ko04973	Carbohydrate digestion and absorption	3	259	44	7612	1.88E-01
ko04974	Protein digestion and absorption	5	259	90	7612	1.91E-01
ko04724	Glutamatergic synapse	6	259	114	7612	1.92E-01
ko05222	Small cell lung cancer	5	259	92	7612	2.03E-01
ko04612	Antigen processing and presentation	4	259	69	7612	2.07E-01
ko04064	NF-kappa B signaling pathway	5	259	93	7612	2.10E-01
ko00790	Folate biosynthesis	2	259	26	7612	2.21E-01
ko05412	Arrhythmogenic right ventricular cardiomyopa	4	259	72	7612	2.29E-01
ko04913	Ovarian steroidogenesis	3	259	49	7612	2.32E-01
ko05169	Epstein-Barr virus infection	9	259	198	7612	2.32E-01
ko00601	Glycosphingolipid biosynthesis - lacto and neo	2	259	27	7612	2.34E-01
ko01040	Biosynthesis of unsaturated fatty acids	2	259	27	7612	2.34E-01
ko00062	Fatty acid elongation	2	259	27	7612	2.34E-01
ko04713	Circadian entrainment	5	259	97	7612	2.34E-01
ko04972	Pancreatic secretion	5	259	98	7612	2.41E-01
ko04925	Aldosterone synthesis and secretion	5	259	98	7612	2.41E-01
ko04918	Thyroid hormone synthesis	4	259	74	7612	2.44E-01
ko00650	Butanoate metabolism	2	259	28	7612	2.46E-01
ko05133	Pertussis	4	259	76	7612	2.59E-01
ko05142	Chagas disease (American trypanosomiasis)	5	259	102	7612	2.67E-01
ko05322	Systemic lupus erythematosus	6	259	130	7612	2.81E-01
ko05130	Pathogenic Escherichia coli infection	3	259	55	7612	2.88E-01
ko04742	Taste transduction	4	259	82	7612	3.05E-01
ko04810	Regulation of actin cytoskeleton	9	259	214	7612	3.05E-01
ko04931	Insulin resistance	5	259	108	7612	3.06E-01

ko05145	Toxoplasmosis	5	259	110	7612	3.20E-01
ko04915	Estrogen signaling pathway	6	259	137	7612	3.23E-01
ko04670	Leukocyte transendothelial migration	5	259	112	7612	3.33E-01
ko00250	Alanine, aspartate and glutamate metabolism	2	259	35	7612	3.35E-01
ko04146	Peroxisome	4	259	86	7612	3.36E-01
ko04726	Serotonergic synapse	5	259	113	7612	3.40E-01
ko05225	Hepatocellular carcinoma	7	259	167	7612	3.41E-01
ko04540	Gap junction	4	259	88	7612	3.51E-01
ko04960	Aldosterone-regulated sodium reabsorption	2	259	37	7612	3.60E-01
ko04919	Thyroid hormone signaling pathway	5	259	116	7612	3.60E-01
ko05321	Inflammatory bowel disease (IBD)	3	259	63	7612	3.63E-01
ko04666	Fc gamma R-mediated phagocytosis	4	259	90	7612	3.67E-01
ko04970	Salivary secretion	4	259	91	7612	3.75E-01
ko00533	Glycosaminoglycan biosynthesis - keratan sulf	1	259	14	7612	3.84E-01
ko05032	Morphine addiction	4	259	93	7612	3.90E-01
ko04723	Retrograde endocannabinoid signaling	6	259	148	7612	3.90E-01
ko05226	Gastric cancer	6	259	148	7612	3.90E-01
ko04927	Cortisol synthesis and secretion	3	259	66	7612	3.91E-01
ko04720	Long-term potentiation	3	259	67	7612	4.00E-01
ko04664	Fc epsilon RI signaling pathway	3	259	67	7612	4.00E-01
ko00604	Glycosphingolipid biosynthesis - ganglio serie	1	259	15	7612	4.05E-01
ko00603	Glycosphingolipid biosynthesis - globo and iso	1	259	15	7612	4.05E-01
ko04110	Cell cycle	5	259	124	7612	4.15E-01
ko04924	Renin secretion	3	259	69	7612	4.19E-01
ko05211	Renal cell carcinoma	3	259	69	7612	4.19E-01
ko04114	Oocyte meiosis	5	259	125	7612	4.22E-01
ko04390	Hippo signaling pathway	6	259	154	7612	4.27E-01
ko04662	B cell receptor signaling pathway	3	259	70	7612	4.28E-01
ko05170	Human immunodeficiency virus 1 infection	8	259	211	7612	4.29E-01
ko04914	Progesterone-mediated oocyte maturation	4	259	99	7612	4.37E-01
ko00071	Fatty acid degradation	2	259	44	7612	4.45E-01
ko04976	Bile secretion	3	259	72	7612	4.46E-01
ko04520	Adherens junction	3	259	72	7612	4.46E-01
ko04934	Cushing syndrome	6	259	158	7612	4.51E-01
ko04660	T cell receptor signaling pathway	4	259	101	7612	4.52E-01
ko04916	Melanogenesis	4	259	101	7612	4.52E-01
ko02010	ABC transporters	2	259	45	7612	4.56E-01
ko04620	Toll-like receptor signaling pathway	4	259	102	7612	4.59E-01
ko03320	PPAR signaling pathway	3	259	74	7612	4.64E-01
ko00450	Selenocompound metabolism	1	259	18	7612	4.64E-01
ko04672	Intestinal immune network for IgA production	2	259	46	7612	4.67E-01
ko04625	C-type lectin receptor signaling pathway	4	259	104	7612	4.74E-01
ko04630	Jak-STAT signaling pathway	6	259	162	7612	4.76E-01
ko04217	Necroptosis	6	259	162	7612	4.76E-01
ko04930	Type II diabetes mellitus	2	259	47	7612	4.79E-01
ko00600	Sphingolipid metabolism	2	259	47	7612	4.79E-01
ko04659	Th17 cell differentiation	4	259	105	7612	4.82E-01
ko04961	Endocrine and other factor-regulated calcium r	2	259	48	7612	4.90E-01
ko04330	Notch signaling pathway	2	259	48	7612	4.90E-01
ko00270	Cysteine and methionine metabolism	2	259	48	7612	4.90E-01

ko00280	Valine, leucine and isoleucine degradation	2	259	48	7612	4.90E-01
ko00532	Glycosaminoglycan biosynthesis - chondroitin	1	259	20	7612	5.00E-01
ko00670	One carbon pool by folate	1	259	20	7612	5.00E-01
ko05320	Autoimmune thyroid disease	2	259	50	7612	5.12E-01
ko05164	Influenza A	6	259	169	7612	5.17E-01
ko00220	Arginine biosynthesis	1	259	21	7612	5.17E-01
ko04668	TNF signaling pathway	4	259	110	7612	5.18E-01
ko05014	Amyotrophic lateral sclerosis (ALS)	2	259	51	7612	5.22E-01
ko05010	Alzheimer disease	6	259	171	7612	5.29E-01
ko04014	Ras signaling pathway	8	259	233	7612	5.41E-01
ko03430	Mismatch repair	1	259	23	7612	5.49E-01
ko04964	Proximal tubule bicarbonate reclamation	1	259	23	7612	5.49E-01
ko00515	Mannose type O-glycan biosynthesis	1	259	23	7612	5.49E-01
ko05152	Tuberculosis	6	259	175	7612	5.51E-01
ko05224	Breast cancer	5	259	147	7612	5.65E-01
ko05210	Colorectal cancer	3	259	86	7612	5.65E-01
ko05034	Alcoholism	6	259	180	7612	5.79E-01
ko00240	Pyrimidine metabolism	2	259	57	7612	5.83E-01
ko04727	GABAergic synapse	3	259	89	7612	5.88E-01
ko05213	Endometrial cancer	2	259	58	7612	5.92E-01
ko04950	Maturity onset diabetes of the young	1	259	26	7612	5.94E-01
ko04150	mTOR signaling pathway	5	259	152	7612	5.95E-01
ko04921	Oxytocin signaling pathway	5	259	153	7612	6.00E-01
ko04966	Collecting duct acid secretion	1	259	27	7612	6.08E-01
ko04080	Neuroactive ligand-receptor interaction	11	259	340	7612	6.13E-01
ko04912	GnRH signaling pathway	3	259	93	7612	6.18E-01
ko00561	Glycerolipid metabolism	2	259	61	7612	6.20E-01
ko04730	Long-term depression	2	259	61	7612	6.20E-01
ko04650	Natural killer cell mediated cytotoxicity	4	259	126	7612	6.26E-01
ko05217	Basal cell carcinoma	2	259	63	7612	6.38E-01
ko00590	Arachidonic acid metabolism	2	259	64	7612	6.46E-01
ko00760	Nicotinate and nicotinamide metabolism	1	259	30	7612	6.47E-01
ko05215	Prostate cancer	3	259	97	7612	6.47E-01
ko04728	Dopaminergic synapse	4	259	131	7612	6.57E-01
ko01523	Antifolate resistance	1	259	31	7612	6.59E-01
ko03020	RNA polymerase	1	259	31	7612	6.59E-01
ko00230	Purine metabolism	4	259	132	7612	6.63E-01
ko04068	FoxO signaling pathway	4	259	133	7612	6.69E-01
ko00640	Propanoate metabolism	1	259	32	7612	6.70E-01
ko00830	Retinol metabolism	2	259	68	7612	6.79E-01
ko00051	Fructose and mannose metabolism	1	259	33	7612	6.82E-01
ko04010	MAPK signaling pathway	9	259	296	7612	6.84E-01
ko00010	Glycolysis / Gluconeogenesis	2	259	69	7612	6.87E-01
ko04922	Glucagon signaling pathway	3	259	103	7612	6.87E-01
ko00040	Pentose and glucuronate interconversions	1	259	34	7612	6.93E-01
ko04920	Adipocytokine signaling pathway	2	259	70	7612	6.94E-01
ko05168	Herpes simplex virus 1 infection	15	259	489	7612	7.01E-01
ko05020	Prion diseases	1	259	35	7612	7.03E-01
ko01524	Platinum drug resistance	2	259	72	7612	7.09E-01
ko04115	p53 signaling pathway	2	259	72	7612	7.09E-01

ko05012	Parkinson disease	4	259	142	7612	7.18E-01
ko05100	Bacterial invasion of epithelial cells	2	259	74	7612	7.23E-01
ko04261	Adrenergic signaling in cardiomyocytes	4	259	145	7612	7.33E-01
ko05220	Chronic myeloid leukemia	2	259	76	7612	7.37E-01
ko04072	Phospholipase D signaling pathway	4	259	147	7612	7.43E-01
ko04260	Cardiac muscle contraction	2	259	78	7612	7.50E-01
ko04721	Synaptic vesicle cycle	2	259	78	7612	7.50E-01
ko00260	Glycine, serine and threonine metabolism	1	259	40	7612	7.51E-01
ko04216	Ferroptosis	1	259	40	7612	7.51E-01
ko00860	Porphyrin and chlorophyll metabolism	1	259	42	7612	7.67E-01
ko04722	Neurotrophin signaling pathway	3	259	119	7612	7.76E-01
ko04962	Vasopressin-regulated water reabsorption	1	259	44	7612	7.83E-01
ko04012	ErbB signaling pathway	2	259	85	7612	7.91E-01
ko00565	Ether lipid metabolism	1	259	47	7612	8.04E-01
ko00520	Amino sugar and nucleotide sugar metabolism	1	259	48	7612	8.11E-01
ko05161	Hepatitis B	4	259	163	7612	8.11E-01
ko04658	Th1 and Th2 cell differentiation	2	259	90	7612	8.17E-01
ko05110	Vibrio cholerae infection	1	259	50	7612	8.24E-01
ko00510	N-Glycan biosynthesis	1	259	50	7612	8.24E-01
ko04979	Cholesterol metabolism	1	259	50	7612	8.24E-01
ko01522	Endocrine resistance	2	259	96	7612	8.44E-01
ko03460	Fanconi anemia pathway	1	259	54	7612	8.47E-01
ko00564	Glycerophospholipid metabolism	2	259	97	7612	8.48E-01
ko05231	Choline metabolism in cancer	2	259	99	7612	8.56E-01
ko04923	Regulation of lipolysis in adipocytes	1	259	56	7612	8.57E-01
ko00480	Glutathione metabolism	1	259	56	7612	8.57E-01
ko04070	Phosphatidylinositol signaling system	2	259	100	7612	8.60E-01
ko04066	HIF-1 signaling pathway	2	259	100	7612	8.60E-01
ko00310	Lysine degradation	1	259	59	7612	8.71E-01
ko00140	Steroid hormone biosynthesis	1	259	60	7612	8.76E-01
ko04213	Longevity regulating pathway - multiple speci	1	259	64	7612	8.92E-01
ko05131	Shigellosis	1	259	65	7612	8.96E-01
ko05223	Non-small cell lung cancer	1	259	66	7612	8.99E-01
ko05221	Acute myeloid leukemia	1	259	66	7612	8.99E-01
ko05160	Hepatitis C	3	259	155	7612	9.03E-01
ko05120	Epithelial cell signaling in Helicobacter pylori	1	259	68	7612	9.06E-01
ko04917	Prolactin signaling pathway	1	259	70	7612	9.12E-01
ko04622	RIG-I-like receptor signaling pathway	1	259	70	7612	9.12E-01
ko05203	Viral carcinogenesis	4	259	200	7612	9.14E-01
ko05218	Melanoma	1	259	72	7612	9.18E-01
ko04141	Protein processing in endoplasmic reticulum	3	259	165	7612	9.24E-01
ko04142	Lysosome	2	259	123	7612	9.26E-01
ko05212	Pancreatic cancer	1	259	75	7612	9.26E-01
ko00562	Inositol phosphate metabolism	1	259	75	7612	9.26E-01
ko03008	Ribosome biogenesis in eukaryotes	1	259	78	7612	9.34E-01
ko00983	Drug metabolism - other enzymes	1	259	78	7612	9.34E-01
ko04530	Tight junction	3	259	171	7612	9.35E-01
ko03018	RNA degradation	1	259	79	7612	9.36E-01
ko01521	EGFR tyrosine kinase inhibitor resistance	1	259	79	7612	9.36E-01
ko04140	Autophagy - animal	2	259	129	7612	9.38E-01

ko05204	Chemical carcinogenesis	1	259	82	7612	9.42E-01
ko00190	Oxidative phosphorylation	2	259	133	7612	9.45E-01
ko03015	mRNA surveillance pathway	1	259	91	7612	9.58E-01
ko04211	Longevity regulating pathway	1	259	91	7612	9.58E-01
ko05016	Huntington disease	3	259	194	7612	9.64E-01
ko04932	Non-alcoholic fatty liver disease (NAFLD)	2	259	149	7612	9.65E-01
ko04024	cAMP signaling pathway	3	259	212	7612	9.78E-01
ko04152	AMPK signaling pathway	1	259	121	7612	9.85E-01
ko04714	Thermogenesis	3	259	231	7612	9.87E-01
ko03040	Spliceosome	1	259	133	7612	9.90E-01
ko03010	Ribosome	1	259	134	7612	9.91E-01
ko04144	Endocytosis	3	259	244	7612	9.91E-01
ko04120	Ubiquitin mediated proteolysis	1	259	136	7612	9.91E-01
ko05162	Measles	1	259	138	7612	9.92E-01
ko04910	Insulin signaling pathway	1	259	138	7612	9.92E-01
ko04740	Olfactory transduction	3	259	442	7612	1.00E+00

Supplementary Table 6. GSEA plots showing the pathways of DEGs altered by IGF2BP3 were involved in acute myeloid leukemia cells.

ID	setSize	enrichmentScore	NES	pvalue	p.adjust	qvalues	rank	leading_edge
KEGG_ALLOGRAFT_REJECTION	30	-0.8316	-1.8057	0.0001	0.0049	0.0024	3429	tags=37%, list=6%, signal=34%
KEGG_GLYOXYLATE_AND_DICARBOXYLATE_METABOLISM	16	0.6513	2.3934	0.0003	0.0049	0.0024	7757	tags=62%, list=15%, signal=53%
KEGG_STEROID_BIOSYNTHESIS	17	0.7294	2.7401	0.0003	0.0049	0.0024	5646	tags=71%, list=11%, signal=63%
KEGG_MISMATCH_REPAIR	23	0.6863	2.8717	0.0003	0.0049	0.0024	7417	tags=70%, list=14%, signal=60%
KEGG_GLYCOSYLPHOSPHATIDYLINOSITOL_GPI_ANCHOR_BI(	25	0.6466	2.7761	0.0004	0.0049	0.0024	7931	tags=56%, list=15%, signal=48%
KEGG_HOMOLOGOUS_RECOMBINATION	28	0.6119	2.7337	0.0004	0.0049	0.0024	8033	tags=57%, list=15%, signal=48%
KEGG_RNA_POLYMERASE	28	0.6471	2.8911	0.0004	0.0049	0.0024	7381	tags=59%, list=14%, signal=50%
KEGG_CITRATE_CYCLE_TCA_CYCLE	29	0.5994	2.7114	0.0004	0.0049	0.0024	8542	tags=62%, list=16%, signal=52%
KEGG_PROPANOATE_METABOLISM	29	0.5847	2.6452	0.0004	0.0049	0.0024	8370	tags=55%, list=16%, signal=46%
KEGG_AUTOIMMUNE_THYROID_DISEASE	43	-0.7697	-1.7677	0.0005	0.0049	0.0024	2378	tags=23%, list=5%, signal=22%
KEGG_TERPENOID_BACKBONE_BIOSYNTHESIS	14	0.6786	2.3818	0.0005	0.0049	0.0024	3840	tags=50%, list=7%, signal=46%
KEGG_BASAL_TRANSCRIPTION_FACTORS	35	0.4965	2.3958	0.0005	0.0049	0.0024	8381	tags=43%, list=16%, signal=36%
KEGG_BASE_EXCISION_REPAIR	35	0.7240	3.4934	0.0005	0.0049	0.0024	7577	tags=66%, list=14%, signal=56%
KEGG_DNA_REPLICATION	36	0.7687	3.7527	0.0005	0.0049	0.0024	7566	tags=81%, list=14%, signal=69%
KEGG_SNARE_INTERACTIONS_IN_VESICULAR_TRANSPORT	36	0.5471	2.6708	0.0005	0.0049	0.0024	7973	tags=44%, list=15%, signal=38%
KEGG_FATTY_ACID_METABOLISM	38	0.5604	2.7884	0.0006	0.0049	0.0024	7222	tags=58%, list=14%, signal=50%
KEGG_PYRUVATE_METABOLISM	38	0.4529	2.2534	0.0006	0.0049	0.0024	8370	tags=45%, list=16%, signal=38%
KEGG_LYSINE_DEGRADATION	39	0.6041	3.0359	0.0006	0.0049	0.0024	8542	tags=59%, list=16%, signal=49%

KEGG_VASOPRESSIN_REGULATED_WATER_REABSORPTION	40	0.5616	2.8503	0.0006	0.0049	0.0024	7925	tags=52%, list=15%, signal=45%
KEGG_AMINO_SUGAR_AND_NUCLEOTIDE_SUGAR_METABOLI	41	0.6666	3.4035	0.0006	0.0049	0.0024	8474	tags=66%, list=16%, signal=55%
KEGG_SULFUR_METABOLISM	10	0.6705	2.0755	0.0007	0.0049	0.0024	8439	tags=60%, list=16%, signal=50%
KEGG_NUCLEOTIDE_EXCISION_REPAIR	44	0.6954	3.6393	0.0007	0.0049	0.0024	7744	tags=61%, list=15%, signal=52%
KEGG_PROTEASOME	44	0.5817	3.0442	0.0007	0.0049	0.0024	8483	tags=55%, list=16%, signal=46%
KEGG_GALACTOSE_METABOLISM	24	0.5612	2.3723	0.0007	0.0049	0.0024	8166	tags=58%, list=15%, signal=49%
KEGG_ENDOMETRIAL_CANCER	45	0.5482	2.8855	0.0007	0.0049	0.0024	8247	tags=62%, list=16%, signal=53%
KEGG_GLUTATHIONE_METABOLISM	45	0.5686	2.9927	0.0007	0.0049	0.0024	6741	tags=49%, list=13%, signal=43%
KEGG_N_GLYCAN_BIOSYNTHESIS	45	0.5637	2.9668	0.0007	0.0049	0.0024	7262	tags=44%, list=14%, signal=38%
KEGG_ARACHIDONIC_ACID_METABOLISM	47	0.4279	2.2956	0.0008	0.0049	0.0024	3508	tags=26%, list=7%, signal=24%
KEGG_PATHOGENIC_ESCHERICHIA_COLI_INFECTION	48	0.4890	2.6376	0.0008	0.0049	0.0024	6795	tags=54%, list=13%, signal=47%
KEGG_VIBRIO_CHOLERAE_INFECTION	48	0.5890	3.1767	0.0008	0.0049	0.0024	8489	tags=58%, list=16%, signal=49%
KEGG_INOSITOL_PHOSPHATE_METABOLISM	51	0.5465	2.9763	0.0009	0.0051	0.0025	8131	tags=59%, list=15%, signal=50%
KEGG_NON_SMALL_CELL_LUNG_CANCER	52	0.5741	3.1430	0.0009	0.0051	0.0025	8282	tags=62%, list=16%, signal=52%
KEGG_GLYCOSAMINOGLYCAN_DEGRADATION	20	0.5692	2.2633	0.0009	0.0051	0.0025	8174	tags=50%, list=15%, signal=42%
KEGG_ACUTE_MYELOID_LEUKEMIA	53	0.6215	3.4415	0.0010	0.0051	0.0025	8247	tags=62%, list=16%, signal=53%
KEGG_INTESTINAL_IMMUNE_NETWORK_FOR_IGA_PRODUCTI	37	-0.7817	-1.7543	0.0010	0.0051	0.0025	4124	tags=35%, list=8%, signal=32%
KEGG_BLADDER_CANCER	35	0.4343	2.0953	0.0010	0.0052	0.0025	8282	tags=57%, list=16%, signal=48%
KEGG_COLORECTAL_CANCER	56	0.5533	3.1161	0.0011	0.0052	0.0025	8247	tags=57%, list=16%, signal=48%

KEGG_NOD_LIKE_RECEPTOR_SIGNALING_PATHWAY	58	0.4266	2.4331	0.0011	0.0052	0.0025	8446	tags=40%, list=16%, signal=33%
KEGG_RNA_DEGRADATION	59	0.4268	2.4362	0.0012	0.0052	0.0025	8306	tags=39%, list=16%, signal=33%
KEGG_ADHERENS_JUNCTION	60	0.4803	2.7434	0.0012	0.0052	0.0025	9075	tags=58%, list=17%, signal=48%
KEGG_EPITHELIAL_CELL_SIGNALING_IN_HELICOBACTER_PY	60	0.4609	2.6325	0.0012	0.0052	0.0025	8212	tags=45%, list=16%, signal=38%
KEGG_P53_SIGNALING_PATHWAY	60	0.5516	3.1506	0.0012	0.0052	0.0025	7964	tags=50%, list=15%, signal=43%
KEGG_GLIOMA	61	0.4914	2.8218	0.0012	0.0052	0.0025	8282	tags=54%, list=16%, signal=46%
KEGG_BUTANOATE_METABOLISM	29	0.4909	2.2208	0.0013	0.0052	0.0025	8370	tags=52%, list=16%, signal=44%
KEGG_GLYCEROPHOSPHOLIPID_METABOLISM	63	0.3977	2.3157	0.0013	0.0052	0.0025	8385	tags=43%, list=16%, signal=36%
KEGG_RENAL_CELL_CARCINOMA	63	0.3853	2.2439	0.0013	0.0052	0.0025	8247	tags=52%, list=16%, signal=44%
KEGG_PANCREATIC_CANCER	65	0.5012	2.9553	0.0014	0.0054	0.0026	8282	tags=60%, list=16%, signal=51%
KEGG_ARGININE_AND_PROLINE_METABOLISM	45	0.4097	2.1564	0.0015	0.0055	0.0027	7942	tags=60%, list=15%, signal=51%
KEGG_SPHINGOLIPID_METABOLISM	34	0.4454	2.1304	0.0015	0.0055	0.0027	8538	tags=65%, list=16%, signal=54%
KEGG_B_CELL_RECEPTOR_SIGNALING_PATHWAY	68	0.4116	2.4653	0.0015	0.0056	0.0027	8384	tags=50%, list=16%, signal=42%
KEGG_CHRONIC_MYELOID_LEUKEMIA	69	0.5063	3.0420	0.0016	0.0057	0.0027	8371	tags=52%, list=16%, signal=44%
KEGG_SMALL_CELL_LUNG_CANCER	71	0.5027	3.0182	0.0016	0.0058	0.0028	8282	tags=49%, list=16%, signal=42%
KEGG_PHOSPHATIDYLINOSITOL_SIGNALING_SYSTEM	72	0.4405	2.6330	0.0017	0.0058	0.0028	8247	tags=51%, list=16%, signal=43%
KEGG_VEGF_SIGNALING_PATHWAY	72	0.4566	2.7289	0.0017	0.0058	0.0028	8247	tags=42%, list=16%, signal=35%
KEGG_CIRCADIAN_RHYTHM_MAMMAL	11	0.6271	2.0118	0.0018	0.0061	0.0029	8169	tags=55%, list=15%, signal=46%
KEGG_ASTHMA	26	-0.8006	-1.7016	0.0019	0.0062	0.0030	3111	tags=35%, list=6%, signal=33%

KEGG_PROGESTERONE_MEDIATED_OOCYTE_MATURATION	79	0.3893	2.3717	0.0021	0.0066	0.0032	8555	tags=41%, list=16%, signal=34%
KEGG_ERBB_SIGNALING_PATHWAY	81	0.3838	2.3644	0.0022	0.0067	0.0033	8247	tags=44%, list=16%, signal=38%
KEGG_PROSTATE_CANCER	81	0.5034	3.1017	0.0022	0.0067	0.0033	8282	tags=53%, list=16%, signal=45%
KEGG_TYPE_I_DIABETES_MELLITUS	34	-0.7673	-1.6979	0.0023	0.0069	0.0033	3429	tags=29%, list=6%, signal=28%
KEGG_APOPTOSIS	84	0.5183	3.2353	0.0023	0.0070	0.0034	8452	tags=60%, list=16%, signal=50%
KEGG_FC_GAMMA_R_MEDIATED_PHAGOCYTOSIS	89	0.5588	3.5433	0.0027	0.0081	0.0039	8371	tags=53%, list=16%, signal=45%
KEGG_GNRH_SIGNALING_PATHWAY	90	0.3103	1.9704	0.0028	0.0081	0.0039	8141	tags=38%, list=15%, signal=32%
KEGG_PYRIMIDINE_METABOLISM	93	0.5993	3.8197	0.0029	0.0084	0.0041	8088	tags=56%, list=15%, signal=48%
KEGG_AMINOACYL_TRNA_BIOSYNTHESIS	22	0.4927	2.0228	0.0030	0.0085	0.0041	8149	tags=45%, list=15%, signal=38%
KEGG_OOCYTE_MEIOSIS	105	0.4456	2.9022	0.0044	0.0123	0.0060	8555	tags=45%, list=16%, signal=38%
KEGG_LYSOSOME	115	0.5287	3.5590	0.0052	0.0141	0.0068	8152	tags=59%, list=15%, signal=50%
KEGG_CELL_CYCLE	116	0.5063	3.4187	0.0053	0.0142	0.0068	8555	tags=52%, list=16%, signal=43%
KEGG_VIRAL_MYOCARDITIS	56	-0.7017	-1.6604	0.0054	0.0144	0.0069	2378	tags=21%, list=5%, signal=20%
KEGG_NEUROTROPHIN_SIGNALING_PATHWAY	117	0.4145	2.8059	0.0055	0.0144	0.0069	8426	tags=46%, list=16%, signal=39%
KEGG_RIG_I_LIKE_RECEPTOR_SIGNALING_PATHWAY	65	0.3142	1.8525	0.0056	0.0144	0.0070	8157	tags=31%, list=15%, signal=26%
KEGG_PROTEIN_EXPORT	24	0.4533	1.9164	0.0058	0.0147	0.0071	8489	tags=42%, list=16%, signal=35%
KEGG_PARKINSONS_DISEASE	121	0.5383	3.6038	0.0063	0.0157	0.0076	8300	tags=65%, list=16%, signal=55%
KEGG_GLYCOSAMINOGLYCAN_BIOSYNTHESIS_CHONDROITI[illegible]	19	0.4765	1.8626	0.0064	0.0157	0.0076	10913	tags=53%, list=21%, signal=42%
KEGG_CYTOSOLIC_DNA_SENSING_PATHWAY	50	0.3559	1.9340	0.0069	0.0168	0.0081	7781	tags=30%, list=15%, signal=26%

KEGG_SPLICEOSOME	125	0.5357	3.6195	0.0071	0.0170	0.0082	8359	tags=50%, list=16%, signal=42%
KEGG_CELL_ADHESION_MOLECULES_CAMS	107	-0.6324	-1.5537	0.0072	0.0170	0.0082	4124	tags=22%, list=8%, signal=21%
KEGG_INSULIN_SIGNALING_PATHWAY	128	0.3810	2.5907	0.0076	0.0177	0.0085	8247	tags=45%, list=16%, signal=38%
KEGG_OXIDATIVE_PHOSPHORYLATION	128	0.5571	3.7887	0.0076	0.0177	0.0085	8212	tags=64%, list=16%, signal=54%
KEGG_UBIQUITIN_MEDIATED_PROTEOLYSIS	132	0.5180	3.5767	0.0084	0.0190	0.0092	8555	tags=52%, list=16%, signal=44%
KEGG_PANTOTHENATE_AND_COA_BIOSYNTHESIS	12	0.5467	1.8107	0.0085	0.0190	0.0092	8342	tags=50%, list=16%, signal=42%
KEGG_GRAFT_VERSUS_HOST_DISEASE	32	-0.7416	-1.6278	0.0085	0.0190	0.0092	3429	tags=25%, list=6%, signal=23%
KEGG_TOLL_LIKE_RECEPTOR_SIGNALING_PATHWAY	93	0.2749	1.7520	0.0088	0.0193	0.0093	8446	tags=43%, list=16%, signal=36%
KEGG_GLYCEROLIPID_METABOLISM	43	0.3634	1.8821	0.0089	0.0193	0.0093	8614	tags=49%, list=16%, signal=41%
KEGG_WNT_SIGNALING_PATHWAY	136	0.3287	2.2792	0.0090	0.0194	0.0094	8238	tags=46%, list=16%, signal=39%
KEGG_GAP_JUNCTION	74	0.2821	1.7028	0.0091	0.0195	0.0094	7361	tags=36%, list=14%, signal=31%
KEGG_PURINE_METABOLISM	141	0.3783	2.6500	0.0105	0.0221	0.0107	7828	tags=41%, list=15%, signal=35%
KEGG_VALINE_LEUCINE_AND_ISOLEUCINE_DEGRADATION	40	0.3585	1.8195	0.0129	0.0267	0.0129	8370	tags=62%, list=16%, signal=53%
KEGG_NATURAL_KILLER_CELL_MEDIATED_CYTOTOXICITY	123	0.2792	1.8813	0.0137	0.0282	0.0136	8452	tags=37%, list=16%, signal=31%
KEGG_ALZHEIMERS_DISEASE	154	0.4750	3.3632	0.0143	0.0290	0.0140	8438	tags=61%, list=16%, signal=51%
KEGG_NEUROACTIVE_LIGAND_RECEPTOR_INTERACTION	231	-0.5471	-1.3486	0.0207	0.0417	0.0201	3406	tags=13%, list=6%, signal=13%
KEGG_HUNTINGTONS_DISEASE	173	0.4547	3.3628	0.0222	0.0442	0.0214	8074	tags=59%, list=15%, signal=51%
KEGG_SYSTEMIC_LUPUS_ERYTHEMATOSUS	52	-0.6642	-1.5635	0.0241	0.0473	0.0229	3111	tags=21%, list=6%, signal=20%
KEGG_RIBOFLAVIN_METABOLISM	12	0.5000	1.6559	0.0269	0.0523	0.0253	8373	tags=42%, list=16%, signal=35%

KEGG_CYTOKINE_CYTOKINE_RECEPTOR_INTERACTION	219	-0.5428	-1.3380	0.0284	0.0548	0.0265	4445	tags=21%, list=8%, signal=19%
KEGG_ETHER_LIPID_METABOLISM	28	0.3659	1.6346	0.0345	0.0658	0.0318	6284	tags=39%, list=12%, signal=35%
KEGG_CYSTEINE_AND_METHIONINE_METABOLISM	31	0.3511	1.6247	0.0351	0.0663	0.0320	6663	tags=29%, list=13%, signal=25%
KEGG_THYROID_CANCER	25	0.3699	1.5883	0.0399	0.0745	0.0360	7443	tags=40%, list=14%, signal=34%
KEGG_NON_HOMOLOGOUS_END_JOINING	12	0.4784	1.5847	0.0417	0.0771	0.0372	6989	tags=33%, list=13%, signal=29%
KEGG_DORSO_VENTRAL_AXIS_FORMATION	18	0.4024	1.5419	0.0528	0.0967	0.0467	6483	tags=33%, list=12%, signal=29%
KEGG_TASTE_TRANSDUCTION	47	-0.6105	-1.4158	0.0869	0.1575	0.0761	4161	tags=21%, list=8%, signal=20%
KEGG_AMYOTROPHIC_LATERAL_SCLEROSIS_ALS	49	0.2667	1.4408	0.0892	0.1601	0.0774	8438	tags=43%, list=16%, signal=36%
KEGG_HEMATOPOIETIC_CELL_LINEAGE	58	-0.5917	-1.4048	0.0906	0.1610	0.0778	3111	tags=16%, list=6%, signal=15%
KEGG_OTHER_GLYCAN_DEGRADATION	16	0.3835	1.4091	0.1043	0.1819	0.0879	7206	tags=69%, list=14%, signal=59%
KEGG_GLYCOSPHINGOLIPID_BIOSYNTHESIS_GLOBO_SERIES	13	-0.7386	-1.4265	0.1044	0.1819	0.0879	1509	tags=23%, list=3%, signal=22%
KEGG_GLYCINE_SERINE_AND_THREONINE_METABOLISM	28	-0.6538	-1.4038	0.1059	0.1829	0.0884	4387	tags=39%, list=8%, signal=36%
KEGG_PORPHYRIN_AND_CHLOROPHYLL_METABOLISM	36	0.2888	1.4099	0.1092	0.1868	0.0903	9184	tags=33%, list=17%, signal=28%
KEGG_ONE_CARBON_POOL_BY_FOLATE	16	0.3763	1.3828	0.1186	0.2009	0.0971	6712	tags=31%, list=13%, signal=27%
KEGG_ADIPOCYTOKINE_SIGNALING_PATHWAY	62	0.2299	1.3272	0.1310	0.2200	0.1063	8015	tags=31%, list=15%, signal=26%
KEGG_LINOLEIC_ACID_METABOLISM	25	0.3137	1.3468	0.1410	0.2346	0.1133	19295	tags=60%, list=37%, signal=38%
KEGG_PRIMARY_BILE_ACID_BIOSYNTHESIS	13	-0.7121	-1.3752	0.1446	0.2365	0.1143	2970	tags=23%, list=6%, signal=22%
KEGG_ALPHA_LINOLENIC_ACID_METABOLISM	17	0.3563	1.3383	0.1447	0.2365	0.1143	6063	tags=29%, list=11%, signal=26%
KEGG_TYROSINE_METABOLISM	35	-0.6074	-1.3497	0.1489	0.2394	0.1157	4502	tags=23%, list=9%, signal=21%

KEGG_PHENYLALANINE_METABOLISM	14	-0.6976	-1.3596	0.1491	0.2394	0.1157	1075	tags=21%, list=2%, signal=21%
KEGG_PROXIMAL_TUBULE_BICARBONATE_RECLAMATION	21	0.3250	1.3151	0.1565	0.2474	0.1196	9466	tags=43%, list=18%, signal=35%
KEGG_CALCIUM_SIGNALING_PATHWAY	152	-0.5025	-1.2391	0.1568	0.2474	0.1196	3397	tags=14%, list=6%, signal=13%
KEGG_HYPERTROPHIC_CARDIOMYOPATHY_HCM	69	-0.5389	-1.2979	0.1682	0.2630	0.1271	4818	tags=19%, list=9%, signal=17%
KEGG_ARRHYTHMOGENIC_RIGHT_VENTRICULAR_CARDIOMY	59	-0.5456	-1.2963	0.1768	0.2743	0.1325	2791	tags=15%, list=5%, signal=14%
KEGG_FOLATE_BIOSYNTHESIS	11	0.3987	1.2789	0.1848	0.2841	0.1373	4004	tags=27%, list=8%, signal=25%
KEGG_TRYPTOPHAN_METABOLISM	33	-0.5928	-1.3076	0.1906	0.2906	0.1404	1975	tags=15%, list=4%, signal=15%
KEGG_PRIMARY_IMMUNODEFICIENCY	29	-0.6001	-1.2960	0.2027	0.3066	0.1481	3422	tags=21%, list=6%, signal=19%
KEGG_REGULATION_OF_AUTOPHAGY	33	0.2608	1.2303	0.2165	0.3247	0.1569	7823	tags=27%, list=15%, signal=23%
KEGG_COMPLEMENT_AND_COAGULATION_CASCADES	54	-0.5345	-1.2608	0.2201	0.3274	0.1582	2162	tags=19%, list=4%, signal=18%
KEGG_HISTIDINE_METABOLISM	21	-0.6134	-1.2607	0.2318	0.3389	0.1637	5390	tags=33%, list=10%, signal=30%
KEGG_MATURITY_ONSET_DIABETES_OF_THE_YOUNG	21	-0.6131	-1.2603	0.2323	0.3389	0.1637	1696	tags=14%, list=3%, signal=14%
KEGG_BIOSYNTHESIS_OF_UNSATURATED_FATTY_ACIDS	19	-0.6214	-1.2630	0.2333	0.3389	0.1637	1908	tags=21%, list=4%, signal=20%
KEGG_GLYCOSPHINGOLIPID_BIOSYNTHESIS_LACTO_AND_NE	24	-0.5967	-1.2519	0.2434	0.3502	0.1692	1275	tags=12%, list=2%, signal=12%
KEGG_ASCORBATE_AND_ALDARATE_METABOLISM	22	0.2925	1.2009	0.2450	0.3502	0.1692	27494	tags=77%, list=52%, signal=37%
KEGG_TGF_BETA_SIGNALING_PATHWAY	69	-0.5028	-1.2110	0.2605	0.3659	0.1768	4527	tags=19%, list=9%, signal=17%
KEGG_ECM_RECEPTOR_INTERACTION	51	-0.5211	-1.2222	0.2611	0.3659	0.1768	4818	tags=33%, list=9%, signal=30%
KEGG_ANTIGEN_PROCESSING_AND_PRESENTATION	73	-0.4990	-1.2060	0.2619	0.3659	0.1768	3429	tags=10%, list=6%, signal=9%
KEGG_DILATED_CARDIOMYOPATHY	76	-0.4935	-1.1970	0.2775	0.3837	0.1854	3111	tags=17%, list=6%, signal=16%

KEGG_GLYCOSPHINGOLIPID_BIOSYNTHESIS_GANGLIO_SERIE	13	-0.6618	-1.2781	0.2789	0.3837	0.1854	3171	tags=31%, list=6%, signal=29%
KEGG_SELENOAMINO_ACID_METABOLISM	23	0.2748	1.1498	0.2918	0.3985	0.1926	8439	tags=26%, list=16%, signal=22%
KEGG_PATHWAYS_IN_CANCER	278	0.3070	2.2755	0.3333	0.4519	0.2183	8282	tags=45%, list=16%, signal=38%
KEGG_PEROXISOME	73	-0.4653	-1.1246	0.3672	0.4941	0.2387	3037	tags=14%, list=6%, signal=13%
KEGG_ABC_TRANSPORTERS	36	-0.5038	-1.1237	0.3798	0.5073	0.2451	2016	tags=11%, list=4%, signal=11%
KEGG_NITROGEN_METABOLISM	21	0.2587	1.0465	0.3965	0.5255	0.2539	7765	tags=29%, list=15%, signal=24%
KEGG_CARDIAC_MUSCLE_CONTRACTION	73	-0.4558	-1.1016	0.3992	0.5255	0.2539	2330	tags=8%, list=4%, signal=8%
KEGG_RIBOSOME	87	-0.4426	-1.0785	0.4240	0.5490	0.2653	12152	tags=87%, list=23%, signal=67%
KEGG_NICOTINATE_AND_NICOTINAMIDE_METABOLISM	20	-0.5450	-1.1154	0.4257	0.5490	0.2653	1825	tags=25%, list=3%, signal=24%
KEGG_RENIN_ANGIOTENSIN_SYSTEM	15	-0.5885	-1.1553	0.4260	0.5490	0.2653	1836	tags=13%, list=3%, signal=13%
KEGG_BASAL_CELL_CARCINOMA	42	-0.4736	-1.0805	0.4336	0.5549	0.2681	2548	tags=14%, list=5%, signal=14%
KEGG_STARCH_AND_SUCROSE_METABOLISM	46	-0.4493	-1.0398	0.4787	0.6083	0.2939	1133	tags=9%, list=2%, signal=9%
KEGG_HEDGEHOG_SIGNALING_PATHWAY	44	-0.4483	-1.0322	0.4866	0.6101	0.2948	2548	tags=14%, list=5%, signal=13%
KEGG_ALDOSTERONE_REGULATED_SODIUM_REABSORPTION	34	-0.4573	-1.0119	0.4867	0.6101	0.2948	2411	tags=12%, list=5%, signal=11%
KEGG_ALANINE_ASPARTATE_AND_GLUTAMATE_METABOLIS	28	-0.4763	-1.0227	0.4929	0.6136	0.2965	3656	tags=18%, list=7%, signal=17%
KEGG_LEISHMANIA_INFECTION	61	-0.4254	-1.0145	0.5001	0.6184	0.2988	3111	tags=20%, list=6%, signal=19%
KEGG_GLYCOLYSIS_GLUCONEOGENESIS	56	-0.4296	-1.0167	0.5059	0.6213	0.3002	1609	tags=9%, list=3%, signal=9%
KEGG_DRUG_METABOLISM_CYTOCHROME_P450	63	-0.4215	-1.0068	0.5106	0.6229	0.3010	1496	tags=10%, list=3%, signal=9%
KEGG_JAK_STAT_SIGNALING_PATHWAY	142	-0.4076	-1.0046	0.5278	0.6397	0.3091	4470	tags=12%, list=8%, signal=11%

KEGG_BETA_ALANINE_METABOLISM	18	-0.4960	-1.0007	0.5873	0.7071	0.3417	3421	tags=17%, list=6%, signal=16%
KEGG_STEROID_HORMONE_BIOSYNTHESIS	45	-0.3999	-0.9235	0.5951	0.7100	0.3431	1530	tags=11%, list=3%, signal=11%
KEGG_AXON_GUIDANCE	105	-0.3870	-0.9507	0.5975	0.7100	0.3431	3830	tags=11%, list=7%, signal=11%
KEGG_O_GLYCAN_BIOSYNTHESIS	23	-0.4569	-0.9509	0.6206	0.7301	0.3528	3854	tags=22%, list=7%, signal=20%
KEGG_TYPE_II_DIABETES_MELLITUS	43	-0.3891	-0.8935	0.6224	0.7301	0.3528	3610	tags=16%, list=7%, signal=15%
KEGG_MELANOMA	63	-0.3801	-0.9079	0.6330	0.7379	0.3565	2497	tags=8%, list=5%, signal=8%
KEGG_PENTOSE_PHOSPHATE_PATHWAY	26	-0.4349	-0.9243	0.6382	0.7392	0.3571	2498	tags=15%, list=5%, signal=15%
KEGG_PPAR_SIGNALING_PATHWAY	60	-0.3734	-0.8886	0.6499	0.7480	0.3614	4131	tags=18%, list=8%, signal=17%
KEGG_NOTCH_SIGNALING_PATHWAY	42	-0.3733	-0.8517	0.6680	0.7635	0.3689	1502	tags=7%, list=3%, signal=7%
KEGG_RETINOL_METABOLISM	59	-0.3626	-0.8616	0.6717	0.7635	0.3689	2757	tags=5%, list=5%, signal=5%
KEGG_LONG_TERM_DEPRESSION	64	-0.3507	-0.8388	0.6998	0.7905	0.3819	2104	tags=9%, list=4%, signal=9%
KEGG_FC_EPSILON_RI_SIGNALING_PATHWAY	76	-0.3399	-0.8245	0.7231	0.8118	0.3922	5099	tags=14%, list=10%, signal=13%
KEGG_GLYCOSAMINOGLYCAN_BIOSYNTHESIS_KERATAN_SU	14	-0.3374	-0.6576	0.7486	0.8354	0.4036	12479	tags=64%, list=24%, signal=49%
KEGG_GLYCOSAMINOGLYCAN_BIOSYNTHESIS_HEPARAN_SU	21	-0.3759	-0.7726	0.7580	0.8366	0.4042	1434	tags=24%, list=3%, signal=23%
KEGG_TIGHT_JUNCTION	110	-0.3301	-0.8115	0.7589	0.8366	0.4042	3213	tags=8%, list=6%, signal=8%
KEGG_LEUKOCYTE_TRANSENDOTHELIAL_MIGRATION	99	-0.3192	-0.7822	0.7815	0.8564	0.4138	5087	tags=14%, list=10%, signal=13%
KEGG_T_CELL_RECEPTOR_SIGNALING_PATHWAY	96	-0.3137	-0.7674	0.7955	0.8665	0.4187	5099	tags=16%, list=10%, signal=14%
KEGG_FRUCTOSE_AND_MANNOSE_METABOLISM	31	-0.3334	-0.7275	0.8071	0.8740	0.4223	852	tags=13%, list=2%, signal=13%
KEGG_MELANOGENESIS	90	-0.2977	-0.7272	0.8194	0.8820	0.4262	5397	tags=18%, list=10%, signal=16%

KEGG_PRION_DISEASES	31	-0.3162	-0.6899	0.8283	0.8864	0.4283	4351	tags=13%, list=8%, signal=12%
KEGG_LONG_TERM_POTENTIATION	64	-0.2818	-0.6740	0.8336	0.8869	0.4285	4315	tags=9%, list=8%, signal=9%
KEGG_VASCULAR_SMOOTH_MUSCLE_CONTRACTION	97	-0.2908	-0.7122	0.8386	0.8871	0.4286	2694	tags=7%, list=5%, signal=7%
KEGG_ENDOCYTOSIS	157	-0.3082	-0.7596	0.8440	0.8877	0.4289	4586	tags=11%, list=9%, signal=10%
KEGG_MTOR_SIGNALING_PATHWAY	50	-0.2762	-0.6455	0.8609	0.9002	0.4350	2411	tags=18%, list=5%, signal=17%
KEGG_FOCAL_ADHESION	157	-0.2902	-0.7152	0.8783	0.9087	0.4391	5099	tags=16%, list=10%, signal=14%
KEGG_REGULATION_OF_ACTIN_CYTOSKELETON	183	-0.2979	-0.7350	0.8789	0.9087	0.4391	2791	tags=5%, list=5%, signal=5%
KEGG_CHEMOKINE_SIGNALING_PATHWAY	167	-0.2713	-0.6690	0.9129	0.9385	0.4535	5424	tags=18%, list=10%, signal=16%
KEGG_DRUG_METABOLISM_OTHER_ENZYMES	47	-0.2121	-0.4919	0.9380	0.9589	0.4633	1496	tags=6%, list=3%, signal=6%
KEGG_OLFACTORY_TRANSDUCTION	368	-0.2968	-0.7297	0.9509	0.9627	0.4652	2713	tags=4%, list=5%, signal=3%
KEGG_MAPK_SIGNALING_PATHWAY	236	-0.2611	-0.6438	0.9576	0.9627	0.4652	3243	tags=5%, list=6%, signal=5%
KEGG_METABOLISM_OF_XENOBIOTICS_BY_CYTOCHROME_P[illegible]	56	-0.1844	-0.4365	0.9611	0.9627	0.4652	1530	tags=7%, list=3%, signal=7%
KEGG_PENTOSE_AND_GLUCURONATE_INTERCONVERSIONS	25	-0.1591	-0.3361	0.9627	0.9627	0.4652	3247	tags=12%, list=6%, signal=11%
